# Safety, Feasibility, and Effectiveness of Ketogenic Diet in Pediatric Patients With Brain Tumors: A Systematic Review

**DOI:** 10.1155/jnme/7935879

**Published:** 2025-03-18

**Authors:** Hanan AlMutairi, Fiona Mccullough, Khawar Siddiqui, Ibrahim Ghemlas, Manal AlHarbi, Richard Grundy, Madhumita Dandapani

**Affiliations:** ^1^Clinical Nutrition Department, King Faisal Specialist Hospital and Research Center, Riyadh, Saudi Arabia; ^2^Department of Bioscience, University of Nottingham, Nottingham NG7 2RD, UK; ^3^Department of Pediatric Hematology/Oncology, King Faisal Specialist Hospital and Research Center, Riyadh, Saudi Arabia; ^4^Children's Brain Tumour Research Center, Biodiscovery Institute, University of Nottingham, Nottingham NG7 2RD, UK

**Keywords:** astrocytoma, brain, classic ketogenic diet, ependymoma, glioma, low-carbohydrate, medulloblastoma, modified Atkins, pediatric, tumor

## Abstract

**Background:** Evidence suggests the positive effects of ketogenic diet (KD) on cancers by limiting glucose availability to cancer cells. This systematic review aimed to explore the safety, feasibility, and effectiveness of KD in children with brain tumors including diet side effects, patient tolerance and compliance, tumor response, quality of life, and nutritional status.

**Methods:** Six databases were searched for relevant publications between 1995 and 2022; non-English language publications were excluded to avoid misinterpretation. The Joanna Briggs Institute assessment scale for observational studies was used to measure study methodology quality and evaluate the extent to which the bias possibility in study design, conduct, and analysis has been stated. The study was registered in PROSPERO under registration number (CRD42021281620).

**Results:** Ultimately, eight eligible publications involving a total of 11 children with brain tumors following KD were included. Nine patients followed classic KD with medium-chain triglyceride oil, whereas others followed a modified Atkin or low-carbohydrate diet. KD was well-tolerated, having nonsevere side effects. Six patients showed positive tumor response, five improved neurological skills, and four reported growth improvement. Six patients reported a median overall survival of 17.6 months. Lastly, statistical analyses could not be performed; hence, a meta-analysis was not possible.

**Conclusion:** KD may be a safe and feasible dietary intervention for children with brain tumors. However, the effects on tumors remain unclear and require further study. The study limitation included the lack of high-quality and appropriately controlled trials with large samples. Moreover, heterogeneity was observed, and quality-of-life assessments were self-reported, which might have resulted in bias or inaccuracy.

## 1. Introduction

Cancer is a leading cause of death in children in developed countries. In 2017 in the USA, an estimated 15,270 children and adolescents were diagnosed with cancer and 1,790 died from it. Brain tumor was the second most common pediatric cancer after leukemia [1]. Various studies support the hypothesis that cancer cell growth can be decelerated by nutrient deprivation, which was first discovered by Warburg, Wind, and Negelein in 1924 in vitro studies [2]. To enact the Warburg effect against tumor cells, patients should follow a ketogenic diet (KD) [3]. KD is a high-fat, moderate-to-low-portion, and low-carbohydrate diet therapy that increases the number of ketone bodies in the bloodstream above the normal level (> 5 mmol/L) and reduces blood glucose level to 3.6–4.4 mmol/L, to enact a state of ketosis [4, 5]. The common ratio is 4 g of fat for every 1 g of combined protein and carbohydrate, where 90% of total energy comes from fat intake and 10% from the combined protein and carbohydrate intake. Several KDs have been formulated to enhance palatability: classic KD, medium-chain triglyceride (TG) KD, modified Atkin diet, and low-glycemic index ketogenic [6].

Unlike normal cells, cancer cells often depend on substrate-level phosphorylation to meet their energy demands. Normal cells derive most of their usable energy from two sources: The primary source is oxidative phosphorylation of the glucose and/or lactate inside the mitochondria, and the secondary cellular fuel is fatty acid. TGs in the body's fat tissues can release free fatty acid to be used as an energy source, and it can also be broken down by the liver into KBs, including acetoacetate, acetone, and βHB. To proliferate, cancer cells need a glycolytic rate 8 to 200 times higher than that of normal cells to provide 10% more adenosine triphosphate. The dysfunctional mitochondria in cancer cells cannot use KBs to provide energy. Thus, ketones use the apoptotic mechanism to kill cancer cells [7]. Previous trials have predominantly been conducted in adult patient populations [8, 9]. Therefore, in this systematic review, we evaluated KD feasibility, safety, and effectiveness in children with brain tumors according to the level of available evidence upon which a potential association could be based.

## 2. Methods

Priori protocol was developed for this systematic review and registered in PROSPERO (CRD42021281620) since there was no published protocol available to follow. A Population Intervention Comparator Outcomes Study (PICO) framework was used to identify eligible studies with human participants, aged ≤ 18 years, and with brain tumors. Preclinical adult studies and studies involving other medical conditions such as epilepsy, obesity, and diabetes were excluded along with those involving other nutrition regimens rather than KD. All possible control groups and patients on KD were included in the dataset. Outcomes like nutritional status, quality of life, mortality, morbidity, and adverse events along with tumor changes, tolerability, ketones, and blood glucose levels were recorded. All relevant publications between 1995 and 2022 were reviewed to ensure sufficient evidence collection, and to avoid misinterpretation, we decided to exclude all non-English language publications from the list.

### 2.1. Search Strategies

A systematic search using Google Scholar, PubMed, Wiley Online Library, Cochrane, Scopus, and Ovid databases was initiated in August 2020 for primary quantitative observational and interventional studies including case studies, systematic reviews, retrospective or prospective cohort studies, posters, and books. All other studies such as preclinical trials (animal and in vitro), studies related to other medical conditions except brain tumors, nonpediatric (age group > 18 years), and non-English language publications were excluded.

Eligible references were manually searched for any articles missed by the electronic search. Preferred Reporting Items for Systematic Reviews and Meta-Analyses (PRISMA) statement was followed [10]. Two reviewers independently (H.A. and F.M.) assessed whether the studies met the inclusion criteria. Authors were contacted for additional information where required.

### 2.2. Data Extraction

Patient information was extracted from studies or obtained from authors. The relevant data from the included studies were systematically and independently recorded by two investigators. The extracted information included study design, participant characteristics, dietary regimen, and its modifications and follow-ups. Outcomes extracted were as follows: types of adverse events, therapy adherence rate, tumor response, nutrition status, and quality-of-life assessment. Considering that the authors would not have addressed outcomes consistently, we summarized the results using text and tables.

Primary outcomes included feasibility: assessing KD tolerance, patient compliance, duration of diet adherence, and reasons for stopping nutrition therapy or adjusting the diet; safety: assessing adverse events developed during nutrition therapy and determining if the adverse events were related to KD or other factors such as disease progression or treatment; and effectiveness: reporting tumor response in radiology tests or metastatic status and measuring the survival rate in months or weeks at the end of the study. For secondary outcomes, we recorded ketosis: measurement of ketosis levels in the blood and urine, assessment of different diets that induce ketosis, and diet therapy modifications in hyperketosis or hypoglycemic incidents and nutrition: assessment of patients' nutrition status and how this might affect patient growth parameters, using weight, height, and body mass index for age and quality-of-life assessment of patients' mental and physical changes by using gross motor functioning classification system.

### 2.3. Risk of Bias Assessment

Case reports and case series are subject to publication bias; however, they were included owing to limited published data. Two authors (H.A. and F.M.) individually evaluated the risk of bias and level of evidence. The Joanna Briggs Institute (JBI) assessment scale for observational studies was used to measure the quality of study methodology and evaluate the extent to which a study has stated the bias possibility in the study design, conduct, and analysis. Additionally, results were compared with multiple review authors to observe any inconsistencies. Disagreements were resolved by discussion. Furthermore, the process of Grading of Recommendations Assessment Development and Evaluations (GRADE) was performed to evaluate the level of certainty for seven arbitrarily chosen outcome parameters related to safety, feasibility, quality of life, and survival benefits.

### 2.4. Heterogeneity

In the absence of statistical heterogeneity, clinical heterogeneity was used to assess differences in patients' characteristics, types and timing of intervention, and outcomes by reporting the measures as similar or comparable.

### 2.5. Data Synthesis

Due to heterogeneity in study designs and methodologies, outcomes could not be quantitatively pooled to perform subgroup investigations. Therefore, outcomes were presented narratively by describing participants' characteristics, intervention duration, KD modifications, and monitoring. Moreover, the results included hypoglycemia/hyperketosis induced by the therapy, diet tolerance and compliance, the number of side effects, improvements in quality of life, patients' growth, tumor response, and survival rate.

### 2.6. Missing Information

Missing values were deleted in some cases, and where needed, the original authors were contacted for additional information or clarification.

## 3. Results

**Figure 1:**
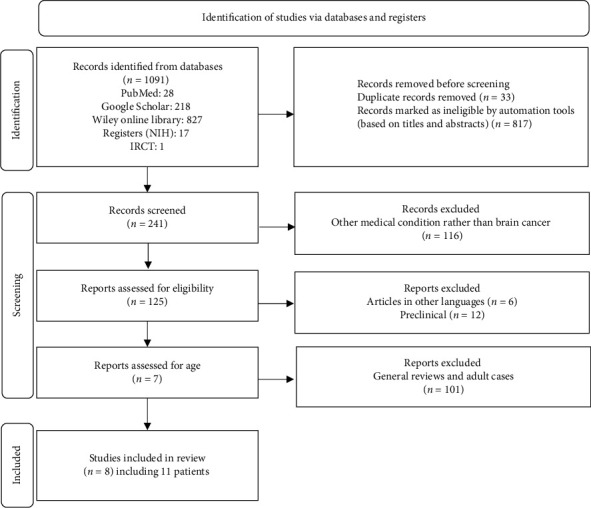
PRISMA 2020 flow diagram.

An electronic search identified 1091 publications. Once duplicates and noncancer cases were removed, trials that failed to meet the inclusion criteria were excluded (*n* = 1083, Supporting Table S4). Overall, 117 full-text studies were retrieved: Six were written in non-English languages, 12 were preclinical trials, and 99 included adult populations and secondary research. Finally, only eight publications involving 11 patients fulfilled the inclusion criteria (Figure 1).

### 3.1. Evidence

The eight publications included the following: two case series, four conference abstracts and posters, and two personal experience accounts, all aiming to evaluate the safety and feasibility of KD in children with brain tumors and its effectiveness in cancer treatment. Nebeling et al. [11] assessed nutritional status and quality of life, Nathan et al. [12] investigated whether fat improved KD efficiency, and van der Louw et al. [13] evaluated KD in patients with no remaining treatment options. Both retrospective and prospective methods were used in this review. Three trials were conducted in university hospitals and two in community hospitals. The remaining trials were personal experiences (details of publications characteristics can be found in Table 1).

The JBI tool was used to assess the risk of bias [18], as shown in Table 2. Patients' history was not reported in detail in the studies by Shen and Brown [15], Nathan et al. [12], and Perez et al. [16]. Assessment methods or diagnostic tests and the outcomes were not clear for both studies by Shen et al. [15] and Schwartz et al. [17] No postintervention clinical conditions were documented for Patients 9 and 16, and Shen and Brown [15] and Seyfried [2] missed reporting information on adverse events.

GRADE evaluation alluded to a “very low” level of certainty for tumor response, neurological skills, and quality-of-life matrices and nutrition-related laboratory profile, whereas for safety, feasibility, growth parameters, and survival, the same was at “low” level in term of parameters of outcome assessment. The reasons for such a lower level of certainty were attributed to the nature of the studies reported, small sample size, and nonhomogeneity of the measurement tools (Table 3).

### 3.2. Patient Characteristics

Among the 11 patients, six were boys, and median age was 5.3 years (2.5–15.5). Four patients were treated in Europe [13, 14], four in the USA [11, 17], and two in Asia [12, 16]. Pathological diagnoses in six patients were diffuse intrinsic pontine glioma, astrocytoma in three patients, and glioblastoma in two patients. All diagnoses were confirmed using magnetic resonance imaging. All patients were treated with chemotherapy, radiotherapy, or surgery (except for case number 8, who was treated with KD only).

Intervention in Cases 4 and 7 required enteral feeding. Nine patients followed KD during disease relapse/progression with neurological deteriorations, while Case 6 started KD at diagnosis with no neurological deterioration and Patient 10 was in remission and in a stable state. Nutritional status showed that Patients 2 and 4 were severely underweight, with −3 standard deviation (SD), and Patients 1 and 5 were normal weight. Patients 3 and 11 were classified as overweight with +2SD and +3SD, respectively. No documented assessments were available for Patients 6–10. Patient characteristics are presented in Supporting Table S1.

### 3.3. Intervention Characteristics

At baseline, Patients 1 to 3 initiated KD as a full liquid diet for 2 weeks, Case 6 followed a low-carbohydrate diet earlier, and the remaining patients were on a regular diet, especially Patient 4, who used PediaSure through a nasogastric tube. All patients followed KD for > 3 months except Patient 5, who followed it for 8 weeks. Moreover, all patients followed classic KD with a maximum ratio of 3.5, except for Patients 6 and 11, who followed the modified Atkin diet and classic Atkins diets, respectively. KD was gradually introduced orally except for Patients 3, 4, and 7, who used enteral feeding.

Regarding energy requirements, Patients 4 and 5 received 120% of recommended dietary allowance, Patients 8 and 9 received 75%, and Patients 1, 3, and 11 received 80%–90%. Carbohydrate intake was varied among patients between 5.6% and 10%, and the Ketocal formula was used with a 4:1 ratio from Patients 1–7. Additionally, medium-chain TG oil was added to the diet with a maximum dose of 150 mL per day excluding Cases 6, 10, and 11. Nebeling et al. [11] and Shen et al. [15] used vitamin and mineral supplements.

KD was frequently modified, and authors reduced the ratio and medium-chain TG oil dosage in a step-wise manner. van der Louw et al. [13] reduced the KD ratio from 1.6:1 to 2.1:1 with carbs intake ranging from 35 to 48 g per day to eliminate vomiting and prevent hyperketosis. Eventually, the formula servings were decreased along with an increase in solid food intake with medium-chain TG oil due to patients' refusal of Ketocal. Cases 8 and 9's diets were fine-tuned to maintain weight and +4 ketones, and Case 11 changed his regimen to energy-restricted KD.

All patients visited their physicians routinely for monitoring and close follow-up, assessing laboratory parameters, neurological skills, and KD toxicity. Nine of 11 patients were followed up by dietitians weekly and five were contacted telephonically or via email if additional support was required. van der Louw et al. [13] reported dietitian home visits. Following diet therapy, three patients continued with KD, and one patient returned to a regular diet (Patient 1) (Supporting Table S2).

### 3.4. KD Safety

Hypoglycemia and hyperketosis were documented in three patients: Patient 1 experienced hypoglycemia and hyperketosis on Day 3; Patient 3 had a decrease in ketone levels below the adequate range, which normalized after discontinuing steroids; and Patient 7 had a moderately elevated KB level. Additionally, Patients 4 and 6 showed slightly elevated serum lipid profiles. No serious gastrointestinal symptoms were reported in children with brain tumors. Four patients reported vomiting and constipation which resolved after regimen adjustment. Moreover, Patient 3 developed swallowing difficulties. In contrast, Patients 8 and 9 reported improvement in symptoms (Supporting Table S3).

### 3.5. KD Feasibility

KD was well tolerated in children with brain tumors without major side effects in both oral and enteral feeding. Moreover, Nebeling et al. [11] reported that parents were motivated during therapy. Patients 1, 2, and 6 refused foods, particularly KD formula, due to additional medical conditions, which was resolved by adding medium-chain TG oil with KD snacks such as shakes, scrambled eggs, tuna, and salad.

Patients who discontinued the diet did not stop because of adverse events; Patients 2 and 6 discontinued because of disease progression, and Patient 6 discontinued after 2 years of being tolerated. Additionally, the parents of Patient 1 decided to discontinue therapy after the study. However, Patients 3, 4, and 10 continued the therapy after the study (Supporting Table S3).

### 3.6. KD Effectiveness

First, tumor changes were assessed by MRI for all the patients. Additionally, Nebeling et al. [11] used positron-emission tomography (PET) scans to look for tumor responses to KD in Cases 4 and 5. Six patients showed a positive tumor response, and Patient 2 reported metastasis. PET scans in patients 4 and 5 showed a decrease in fluorodeoxyglucose uptake to 21%. MRI indicated tumor improvement in Patients 6, 7, 10, and 11, with almost 15% reduction in tumor size in the last two patients.

Secondly, neurological skills and quality of life were assessed. Four patients had neurological deterioration which negatively affected their quality of life [13, 14]. Patient 1 had a gross motor functioning classification system level of 5, and Patient 2 had seizures 3 weeks after the intervention. Patient 3 had nasogastric tube feeding initiated in the eighth week due to swallowing difficulties. A deteriorating clinical condition was noted in Patient 6. Alternatively, five patients experienced an improvement in their neurological condition and overall quality of life and three patients returned to school. Patient 2 showed recovery of skill development, particularly in gait, mobility, speech, and hand coordination. Patients 8, 9, and 10 stated improvement with the diet, with substantial recovery of numerous symptoms. Patient 11 showed improvement in vision, stamina, pituitary function, and a decline in hypothalamic obesity.

Finally, growth was assessed. Four patients reported stabilization in growth SD except for Patient 11, who had a reduction in BMI from −3SD to −2SD. Overall survival (OS) was difficult to determine since not all publications reported the survival duration from the commencement of initial therapy. However, Perez et al. [16] and van der Louw et al. [13] reported OS for six patients and the median was 17.6 months. No death was reported due to KD therapy, as death was caused by disease progression in Patients 2 and 6. Similarly, Patients 1 and 3 died several months after the study (Supporting Table S3).

## 4. Discussion

This review aimed to explore current evidence regarding the safety, feasibility, and effectiveness of KD in children with brain tumors. The different aspects considered included KD adverse events, tolerance, compliance to nutritional therapy, tumor response to the diet, improvements in quality of life, and children's nutritional status. Due to limited evidence, this review discussed publications involving 11 patients in case reports and case series with no comparison, resulting in an Evidence Level 4 (Oxford Center for Evidence-Based Medicine 2011). This is similar to the review by Perez et al. [16] who included six cases in his report. Geographically, cases were equally distributed from Europe and North America (four each), whereas only two cases were from Asia.

KDs have been reported as safe interventions for pediatric patients with brain tumors. First, KD usually increases the serum levels of KBs, acetoacetate, β-hydroxybutyrate, and acetone above the reference range (5 mmol/L) [19], thus developing hyperketosis. However, this review showed that children with brain cancer do not exhibit significant incidents of hyperketosis, possibly due to effective monitoring. Similarly, in pediatric patients with epilepsy and adult cancers, only a few incidents of hyperketosis have been reported [20, 21]. Secondly, while patients undergoing radiotherapy are usually prescribed glucocorticoids to lower brain swelling and tumor edema, this medication has the side effect of significantly increasing blood glucose levels [22]. Importantly, such an increase was not observed in patients following KD. This indicates the potential role of KD in preventing glucocorticoid-induced hyperglycemic episodes. Third, side effects were mainly GI symptoms including vomiting, constipation, and fatigue (Cases 1, 2, 3, and 6), mostly in classic KD and modified Atkin diet. However, for oncology patients, these side effects may result from anticancer treatments or disease progression. Moreover, these adverse events can be resolved by adjusting the diet. Our observations are consistent with those of other studies that reported gastrointestinal symptoms most common among pediatric epilepsy patients [23, 24] and among adult patients [25]. Dyslipidemia is a common adverse event associated with KD. However, only one study by Nebeling et al. [11] observed an increase in cholesterol profile. In contrast, pediatric patients with epilepsy report dyslipidemia as the second most common adverse event [26, 27]. Our results are similar to Rieger et al. [28], where reduced cholesterol levels were reported throughout the diet in adults. Moreover, other studies have observed improvement or normalization of the same [29, 30]. Finally, nonsevere side effects have been reported in pediatric cases of brain tumors, possibly due to the short trial durations. Nevertheless, renal complications, infections, and other major side effects can result from KDs in patients with epilepsy [31]. Similar to children with brain tumors, adult patients did not experience this side effect despite a long therapy duration [32, 33]. Thus, close monitoring and follow-ups could prevent these side effects.

Concerning feasibility, this review reported good adherence, in agreement with Perez et al. [16], and may be related to the short period of nutrition treatment compared with other pediatric trials. The highest KD ratio reached was 3.5:1 for both Patients 7 and 11. However, lower than what epileptic children could tolerate (4:1). Compliance was higher with an oral diet of solid food than with oral nutrition supplements (Patients 1, 2, and 3). The researchers added MCT oil to reach the needed ketosis level, which can cause taste problems in pediatric patients on KD [31]. Hallböök et al. [31] conducted a retrospective study on children with seizures and found a high dietary adherence rate. The main reason for poor adherence was parents finding the therapeutic diet too challenging to follow. Kang et al. [34] found that noncompliance was higher among patients on the diet for longer than 2 years compared with 8 months of therapy with 100% compliance. Likewise, most studies suggested that KD was well tolerated in pediatric cancer patients [35]. Rieger et al. [28] reported that KD has an 85% adherence rate with no serious adverse event.

Regarding tumor prognosis and survival, six out of 11 patients experienced a reduction in tumor size mainly with diffuse intrinsic pontine gliomas patients (approximately 55%), while three cases showed negative outcome. Survival rate improved in both classic KD and modified Atkin diet, and no death was reported due to KD therapy. Evaluation of tumor status and survival outcomes were not clear in many studies owing to small sample sizes and short therapy durations. Although the findings from the included studies might be inconclusive, the evidence from the current literature appears promising. Moreover, adult studies showed that brain MRI indicated a significant reduction in the hyperintensity of tumor regions [36–38]. However, a study by Schwartz et al. [17] showed mixed MRI results. Additionally, Voss et al. [39] found an improvement in the median survival days of 331 for patients who underwent KD therapy compared with 291 days for those on a standard diet.

All patients reported overall improved or stable conditions in this review regarding quality of life, some even went back to school, except for four patients, possibly related to tumor progression. This provides hope for children by lowering social stress during anticancer therapy. Similarly, performance improvement was found in adult patients with brain tumors in trials conducted by Zuccoli et al. [38] and Martin-McGill et al. [40], while negative quality of life was reported by Rieger et al. [28].

Mineral and vitamin deficiencies can occur in children following KD; Nebeling et al. [11] and Shen et al. [15] used vitamin and mineral supplements for their patients (Cases 4, 5, and 7). In an adult population with brain tumors, Bergqvist [41] and Christodoulides et al. [42] observed changes in vitamins A and E, and a substantial decline in magnesium status after 1 year of KD. Hence, dietary micronutrient supplementation in combination with KD therapy was recommended by the international KD study group [43]. A KD guideline for regulating renal complications among patients is the use of potassium citrate [43]. Although these precautions were not observed in the studies reviewed yet, no cases reported kidney stones, possibly due to the short trial durations. Regarding body composition, four patients reported stabilization and no significant changes in growth SD were seen. Patient 11, who was overweight, used an energy-restricted diet to help him reach the normal body mass. However, in adult studies, body weight was reduced following KD treatment compared with baseline [17, 36–38]. The dietitian's role in the management and follow-up of patients is crucial because compliance with such restrictive diets can be challenging, and potential adverse events of the KD may require dietary modifications. Klement and Sweeney [4] encouraged close monitoring and regular follow-ups with the dietitian to improve compliance and ketosis.

The primary limitation of this review is the lack of high-quality, appropriately controlled trials with large samples. Moreover, heterogeneity was observed in the study participants' characteristics including brain tumor diagnosis and treatments, and the use of various KDs. Finally, quality-of-life assessments were self-reported which might have resulted in bias or inaccuracy. Furthermore, statistical analyses could not be performed; hence, a meta-analysis of the data was not possible.

In conclusion, KD can be a safe, feasible, and promising dietary intervention for pediatric patients with brain tumors when monitored by a professional dietitian. This review demonstrated that most adverse events associated with KDs can be resolved by fine-tuning the diet. However, the effects on tumors prognosis and survival rate remain unclear requiring further robust studies.

## Figures and Tables

**Table 1:** Characteristics of reviewed publications.

Included publications	Study type	Level of evidence	Study location	Patient number
1. van der Louw et al. [13]	Case series	Level 4.c	Erasmus MC-Sophia Children's Hospital University Medical Centre, Rotterdam, Netherlands	1, 2, 3
2. Nebeling et al. [11]	Case series	Level 4.c	University Hospital of Cleveland, USA	4, 5
3. Perez et al. [14]	Case report	Level 4.d	University Hospital of Geneva, Geneva, Switzerland	6
4. Shen and Brown [15]	Case report	Level 4.d	Children's Hospital of Orange County, Orange, CA, USA	7
5. Nathan et al. [12]	Case report	Level 4.d	Shushrusha Hospital, Mumbai	8
6. Perez et al. [16]	Case report	Level 4.d	Shushrusha Hospital, Mumbai	9
7. Seyfried [2]	Case report	Level 4.d	Personal experience	10
8. Schwartz et al. [17]	Case report	Level 4.d	Barrows Neurological Institute, Arizona, USA	11

**Table 2:** Joanna Briggs Institute appraisal.

	van der Louw et al. [13]	Nebeling et al. [11]	Perez et al. [14]	Shen and Brown [15]	Nathan et al. [12]	Perez et al. [16]	Seyfried [2]	Schwartz [17]
1. Were patient's demographic characteristics clearly described?	Yes	Yes	Yes	Yes	Yes	Yes	Yes	Yes
2. Was the patient's history clearly described and presented as a timeline?	Yes	Yes	Unclear	No	No	No	Yes	Yes
3. Was the current clinical condition of the patient on presentation clearly described?	Yes	Yes	Yes	Yes	Yes	Yes	Yes	Yes
4. Were diagnostic tests or assessment methods and the results clearly described?	Yes	Yes	Yes	No	Unclear	Yes	Yes	No
5. Was the intervention(s) or treatment procedure(s) clearly described?	Yes	Yes	Yes	Yes	Yes	Yes	Yes	Yes
6. Was the postintervention clinical condition clearly described?	Yes	Yes	Yes	Unclear	Unclear	No	Unclear	Yes
7. Were adverse events (harms) or unanticipated events identified and described?	Yes	Unclear	Yes	No	Yes	Yes	No	Unclear
8. Does the case report provide takeaway lessons?	Yes	Yes	Yes	Yes	Yes	Yes	Yes	Yes

**Table 3:** GRADE assessment for the study evaluation.

Certainty assessment							Impact	Certainty	Importance
No. of studies	Study design	Risk of bias	Inconsistency	Indirectness	Imprecision	Other considerations			
*Safety (follow-up: range 3 months to 2 years; assessed with side effect events)*									
4	Nonrandomized studies	Serious^a^	Not serious	Not serious	Serious^b^	None	Four patients reported vomiting and constipation which resolved after regimen adjustment. One patient developed swallowing difficulties.	⨁⨁◯◯ Low	Critical

*Feasibility (follow-up: range 8 weeks to 2 years; assessed with: diet compliance)*									
7	Nonrandomized studies	Serious^a^	Not serious	Not serious	Serious^b^	None	Three patients refused foods, particularly KD formula, which was resolved by adding MCT oil. Other three patients continued the therapy after the study	⨁⨁◯◯ Low	Critical

*Tumor response (follow-up: range 8 weeks to 2 years; assessed with images)*									
5	Nonrandomized studies	Serious^a^	Serious^c^	Not serious	Serious^b^	None	Six patients showed a positive tumor response and one reported tumor metastasis.	⨁◯◯◯ Very low	Important

*Neurological skills and quality-of-life matrices (follow-up: range 8 weeks to 2 years)*									
7	Nonrandomized studies	Serious^a^	Serious^c^	Not serious	Serious^b^	None	Four patients had neurological deterioration, and five patients experienced an improvement in their neurological condition and overall QoL.	⨁◯◯◯ Very low	Important

*Growth parameters (follow-up: range 3 months to 2 years; assessed with SD and BMI)*									
3	Nonrandomized studies	Serious^a^	Not serious	Not serious	Serious^b^	None	Four patients reported stabilization in growth, and one patient had a reduction in BMI from −3SD to −2SD.	⨁⨁◯◯ Low	Critical

*Nutrition-related laboratory profile (follow-up: range 8 weeks to 2 years)*									
7	Nonrandomized studies	Serious^a^	Not serious	Serious^d^	Serious^b^	None	Hypoglycemia and hyperketosis were documented in three patients, and two patients showed slightly elevated serum lipid profiles.	⨁◯◯◯ Very low	Important

*Survival rate (follow-up: range 3 months to 2 years; assessed with months)*									
4	Nonrandomized studies	Serious^a^	Not serious	Not serious	Serious^b^	None	Two studies reported overall survival for six patients, and the median was 17.6 months. No death was reported due to KD therapy.	⨁⨁◯◯ Low	Important

Abbreviations: BMI, body mass index; SD, standard deviation.

^a^Not all the patients completed the studies.

^b^Observational studies with small sample size.

^c^Nonhomogeneity in measurement tools.

^d^Six out of seven studies reported hypoglycemia and hyperketosis.

## Data Availability

All data are available upon reasonable request from the corresponding author.

## Supporting Information

Supporting Information 1 Table S1: Patient characteristics.

Supporting Information 2 Table S2: Intervention characteristics.

Supporting Information 3 Table S3: Study outcomes.

Supporting Information 4 Table S4: List of studies excluded from the review.

## References

[1] Institute N. C. (2019). Comprehensive Cancer Information.

[2] Seyfried T. (2012). *Cancer as a MetabolicDisease: On the Origin, Management, and Prevention of Cancer*.

[3] Warburg O., Wind F., Negelein E. (1927). The Metabolism of Tumors in the Body. *Journal of General Physiology*.

[4] Klement R. J., Sweeney R. A. (2016). Impact of a Ketogenic Diet Intervention During Radiotherapy on Body Composition: I. Initial Clinical Experience With Six Prospectively Studied Patients. *BMC Research Notes*.

[5] Fine E. J., Segal-Isaacson C., Feinman R. D. (2012). Targeting Insulin Inhibition as a Metabolic Therapy in Advanced Cancer: A Pilot Safety and Feasibility Dietary Trial in 10 Patients. *Nutrition*.

[6] Martin K., Jackson C. F., Levy R. G., Cooper P. N. (2016). Ketogenic Diet and Other Dietary Treatments for Epilepsy. *Cochrane Database of Systematic Reviews*.

[7] Vander Heiden M. G., Cantley L. C., Thompson C. B. (2009). Understanding the Warburg Effect: Themetabolicrequirements of Cellproliferation. *Science*.

[8] Woolf E. C., Syed N., Scheck A. C. (2016). Tumor Metabolism, the Ketogenic Diet and β-Hydroxybutyrate: Novel Approaches to Adjuvant Brain Tumor Therapy. *Frontiers in Molecular Neuroscience*.

[9] Chung H. Y., Park Y. K. (2017). Rationale, Feasibility and Acceptability of Ketogenicdiet for Cancertreatment. *Journal of Cancer*.

[10] Moher D., Liberati A., Tetzlaff J., Altman D. G. (2009). Preferred Reporting Items for Systematic Reviews and Meta-Analyses: The PRISMA Statement. *PLoS Medicine*.

[11] Nebeling L. C., Miraldi F., Shurin S. B., Lerner E. (1995). Effects of a Ketogenic Diet on Tumor Metabolism and Nutritional Status in Pediatric Oncology Patients: Two Case Reports. *Journal of the American College of Nutrition*.

[12] Nathan J., Nathan S., Chadha B., Khedekar D. (2011). Pushing the Frontier-Easier, Safer and More Efficacious Ketogenic Diet. *Epilepsia*.

[13] van der Louw E. J. T. M., Reddingius R. E., Olieman J. F., Neuteboom R. F., Catsman-Berrevoets C. E. (2019). Ketogenic Diet Treatment in Recurrent Diffuse Intrinsic Pontine Glioma in Children: A Safety and Feasibility Study. *Pediatric Blood and Cancer*.

[14] Perez A., Merlini L., El‐Ayadi M., Korff C., Ansari M., von Bueren A. O. (2019). Comment on: Ketogenic Diet Treatment in Recurrent Diffuse Intrinsic Pontine Glioma in Children: A Safety and Feasibility Study. *Pediatric Blood and Cancer*.

[15] Shen T. L., Brown J. (2016). HG-32 Use of ketogenic Diet as a Complimentary Metabolic Therapy During Chemo-Radiation Therapy in A 7 Year Old Female With Glioblastoma. *Neuro-Oncology*.

[16] Perez A., van der Louw E., Nathan J. (2021). Ketogenic Diet Treatment in Diffuse Intrinsic Pontine Glioma in Children: Retrospective Analysis of Feasibility, Safety, and Survival Data. *Cancer Reports*.

[17] Schwartz K., Chang H. T., Nikolai M. (2015). Treatment of Glioma Patients With Ketogenic Diets: Report of Two Cases Treated With an IRB-Approved Energy-Restricted Ketogenic Diet Protocol and Review of the Literature. *Cancer & Metabolism*.

[18] JBI Global Critical Appraisal Tools for Use in JBI Systematic Reviews. https://jbi.global/critical-appraisal-tools.AccessedSeptember62022.

[19] Gupta L., Khandelwal D., Kalra S., Gupta P., Dutta D., Aggarwal S. (2017). Ketogenic Diet in Endocrine Disorders: Current Perspectives. *Journal of Postgraduate Medicine*.

[20] Neal E. G., Chaffe H., Schwartz R. H. (2009). A Randomized Trial of Classical and Medium‐Chain Triglyceride Ketogenic Diets in the Treatment of Childhood Epilepsy. *Epilepsia*.

[21] Erickson N., Boscheri A., Linke B., Huebner J. (2017). Systematic Review: Isocaloric Ketogenic Dietary Regimes for Cancer Patients. *Medical Oncology*.

[22] Kargiotis O., Geka A., Rao J. S., Kyritsis A. P. (2010). Effects of Irradiation on Tumor Cell Survival, Invasion and Angiogenesis. *Journal of Neuro-Oncology*.

[23] Lambrechts D. A. J. E., de Kinderen R. J. A., Vles H. S. H., de Louw A. J., Aldenkamp A. P., Majoie M. J. M. (2015). The MCT-Ketogenic Diet as a Treatment Option in Refractory Childhood Epilepsy: A Prospective Study With 2-Year Follow-Up. *Epilepsy and Behavior*.

[24] Lambrechts D. A. J. E., de Kinderen R. J. A., Vles J. S. H., de Louw A. J. A., Aldenkamp A. P., Majoie H. J. M. (2017). A Randomized Controlled Trial of the Ketogenic Diet in Refractory Childhood Epilepsy. *Acta Neurologica Scandinavica*.

[25] van der Louw E. J. T. M., Olieman J. F., van den Bemt P. M. L. A. (2019). Ketogenic Diet Treatment as Adjuvant to Standard Treatment of Glioblastoma Multiforme: A Feasibility and Safety Study. *Therapeutic Advances in Medical Oncology*.

[26] Kim J. A., Yoon J., Lee E. J. (2016). Efficacy of the Classic Ketogenic and the Modified Atkins Diets in Refractory Childhood Epilepsy. *Epilepsia*.

[27] El‐Rashidy O. F., Nassar M. F., Abdel-Hamid I. A. (2013). Modified Atkins Diet vs Classic Ketogenic Formula in Intractable Epilepsy. *Acta Neurologica Scandinavica*.

[28] Rieger J., Bähr O., Maurer G. D. (2014). Ergo: A Pilot Study of Ketogenic Diet in Recurrent Glioblastoma. *International Journal of Oncology*.

[29] SchmidtM P. N., Schwab M., Strauss I., Kämmerer U. (2011). Effects of a Ketogenic Diet on the Quality of Life in 16 Patients With Advanced Cancer: A Pilot Trial. *Nutrition and Metabolism*.

[30] Abdelwahab M. G., Fenton K. E., Preul M. C. (2012). The Ketogenic Diet Is an Effective Adjuvant to Radiation Therapy for the Treatment of Malignant Glioma. *PLoS One*.

[31] Hallböök T., Sjölander A., Åmark P., Miranda M., Bjurulf B., Dahlin M. (2015). Effectiveness of the Ketogenic Diet Used to Treat Resistant Childhood Epilepsy in Scandinavia. *European Journal of Paediatric Neurology*.

[32] Champ C. E., Palmer J. D., Volek J. S. (2014). Targeting Metabolism With a Ketogenic Diet During the Treatment of Glioblastoma Multiforme. *Journal of Neuro-Oncology*.

[33] Artzi M., Liberman G., Vaisman N. (2017). Changes in Cerebral Metabolism During Ketogenic Diet in Patients With Primary Brain Tumors: 1H-MRS Study. *Journal of Neuro-Oncology*.

[34] Kang H., Lee Y. J., Lee J. S. (2011). Comparison of Short‐Versus Long‐Term Ketogenic Diet for Intractable Infantile Spasms. *Epilepsia*.

[35] Winter S. F., Loebel F., Dietrich J. (2017). Role of Ketogenic Metabolic Therapy in Malignant Glioma: A Systematic Review. *Critical Reviews in Oncology/Hematology*.

[36] Elsakka A. M. A., Bary M. A., Abdelzaher E. (2018). Management of Glioblastoma Multiforme in a Patient Treated With Ketogenic Metabolic Therapy and Modified Standard of Care: A 24-Month Follow-Up. *Frontiers in Nutrition*.

[37] Panhans C. M., Gresham G., Amaral L. J., Hu J. (2020). Exploring the Feasibility and Effects of a Ketogenic Diet in Patients With CNS Malignancies: A Retrospective Case Series. *Frontiers in Neuroscience*.

[38] Zuccoli G., Marcello N., Pisanello A. (2010). Metabolic Management of Glioblastoma Multiforme Using Standard Therapy Together With a Restricted Ketogenic Diet: Case Report. *Nutrition and Metabolism*.

[39] Voss M., Wagner M., von Mettenheim N. (2020). ERGO2: A Prospective, Randomized Trial of Calorie-Restricted Ketogenic Diet and Fasting in Addition to Reirradiation for Malignant Glioma. *International Journal of Radiation Oncology, Biology, Physics*.

[40] Martin-McGill K. J., Marson A. G., Tudur Smith C. (2020). Ketogenic Diets as an Adjuvant Therapy for Glioblastoma (KEATING): A Randomized, Mixed Methods, Feasibility Study. *Journal of Neuro-Oncology*.

[41] Bergqvist A. C. (2012). Long-Term Monitoring of the Ketogenic Diet: Do’s and Don’ts. *Epilepsy Research*.

[42] Christodoulides S. S., Neal E. G., Fitzsimmons G. (2012). The Effect of the Classical and Medium Chain Triglyceride Ketogenic Diet on Vitamin and Mineral Levels. *Journal of Human Nutrition and Dietetics*.

[43] Bodensteiner J. (2009). Commentary on “Optimal Clinical Management of Children Receiving the Ketogenic Diet: Recommendations of the International Ketogenic Diet Study Group. *Epilepsia*.

[44] Almutairi H., Mccullough F., Alharbi M., Dandapani M., Grundy R. Safety, Feasibility, and Effectiveness of Ketogenic Diet in Paediatric Patients With Brain Tumours: A Systematic Review. https://www.authorea.com/users/631801/articles/650918-safety-feasibility-and-effectiveness-of-ketogenic-diet-in-paediatric-patients-with-brain-tumours-a-systematic-review?commit=4db98f903bc2d053c737c61e6e0c45c74d12c294.

