# Supplementary material for: Safety, Feasibility, and Effectiveness of Ketogenic Diet in Pediatric Patients With Brain Tumors: A Systematic Review
Source: J Nutr Metab. 2025 Mar 18;2025:7935879. doi: 10.1155/jnme/7935879 (PMC11936527; doi:10.1155/jnme/7935879)
Supplement: Supporting Information 1 — Table S1: Patient characteristics. [file 7935879.f1.docx]

**TABLE S1 Patient characteristics**

| Case | 1 | 2 | 3 | 4 | 5 | 6 | 7 | 8 | 9 | 10 | 11 |
| --- | --- | --- | --- | --- | --- | --- | --- | --- | --- | --- | --- |
| Age (in years) | 5.3 | 11.6 | 15.5 | 3 | 8.5 | 2.5 | 7 | 15 | 3.7 | 4 | 5 |
| Sex | Boy | Boy | Boy | Girl | Girl | Boy | Girl | Girl | Girl | Boy | Boy |
| Tumour type | DIPG | DIPG | DIPG | Anaplastic astrocytoma stage IV | Recurrent Cerebellar Astrocytoma (grade III) | DIPG | GBM | DIPG | DIPG | Low-grade Glioma | Juvenile pilocytic astrocytoma |
| Treatments | RT within 3 weeks (44.8 Gy in  16 fractions),  three 5-days of TMZ  100 mg/m  2  /day each month | RT combined with gemcitabine | RT (54 Gy in 30 fractions.  bi-weekly),bevacizumab and irinotecan, erlotinib.  week 6 had fractions of 4 Gy | CCG-945 chemo protocol | Surgery  Chemotherapy (CCG-945)  Radiotherapy, 5400 cGys | Three cycles without intraventricular methotrexate of HIT-SKK chemotherapy, followed by proton therapy | Chemoradiation therapy, dexamethasone | Observation only | RT | Chemotherapy stopped after 3 months | Chemo-TX, surgery |
| Medical conditions | Neurological condition  deteriorated again after five cycles of TMZ, and tumor  progression | Neuro-  logical symptoms were noted, with tumour progression  and an ependymal metastasis in the right ventricle.  At the start of the study, neurological examination showed dysfunction of the left trigeminal, abducens, and facial nerves, a mild ataxia on the left side, right hyperreflexia, and a pathological plantar reflex on the right side. The GMFCS level was level 2. | Neurological symptoms and tumour progressed  , a right lateral gaze palsy and central facial paresis, and left pyramidal paresis grade 3 at the arm and grade 4 at the leg with left hyperreflexia and left pathological plantar reflex. The GMFCS grade was level 3 | Increases infection, toxicity, and deteriorating motor skills, | Hearing loss due to chemotherapy and low magnesium level on supplementation | Was clinically well (Lansky performance status of 90%-100%) and steroid-independent at diagnosis. No neurological symptoms and tumour improved.  14 months after treatment, the patient experienced a progression accompanied by a deteriorating clinical condition and steroid dependence. | Requiring tracheostomy and a gastrostomy tube. Mean serum glucose was 119 (98-163) mg/dL | Disease relapse/  progression | Disease relapse/  progression | Huge size and poor prognosis. After the failure of standard protocol, vision, language, cognition, motor skills, and endocrine function deteriorated | Thalamic and hypothalamic mass, ↓Vision, hypothalamic obesity, ↓stamina, ↓pituitary function |
| Nutritional status | WT/HT  +0.05 SD  Ht/age +2.00 SD | Wt/Ht had declined to −3.09 SD | Wt/Ht +2.16 SD, Ht/ age −0.31 SD | WT and HT for age -2 to 3 SD | WT and HT for age are median SD | - | - | - | - | - | BMI for age +3 SD |
