# Supplementary material for: Safety, Feasibility, and Effectiveness of Ketogenic Diet in Pediatric Patients With Brain Tumors: A Systematic Review
Source: J Nutr Metab. 2025 Mar 18;2025:7935879. doi: 10.1155/jnme/7935879 (PMC11936527; doi:10.1155/jnme/7935879)
Supplement: Supporting Information 2 — Table S2: Intervention characteristics. [file 7935879.f2.docx]

**TABLE S2 Intervention characteristics**

| Case | Duration | Nutritional intervention | | | Diet approaches | Special modifications | Diet monitoring/ follow-up |
| --- | --- | --- | --- | --- | --- | --- | --- |
|  |  | Pre-diet | KD | Post-diet |  |  |  |
| 1 | 3 months | KD was initiated as a full liquid (LQ) KD formula with a 4:1 diet ratio, used for a maximum of 2 weeks. | CKD supplemented with MCT emulsion, KD ratio (4:1→1.5:1-2:1). | Normal diet after 6 weeks by NGT. | LQ was offered in different recipes of 100 mL and 200 calories with similar nutritional composition, which could be used interchangeably. When ketone levels of 3 mmol/L were reached, the KD LQ formula was modified into a KD (range diet ratio 1.5:1–2.0:1) in a step-wise manner with strictly calculated and prepared meals. MCT emulsion mixed with low fat milk was used several times daily in addition to the meals. | LQ formula was introduced to a maximum of seven exchanges of 100 mL Ketocal LQ 4:1 ratio and 200 kcal in a step-wise manner. Because the patient vomited and refused the LQ formula, the diet was changed into a KD of 1.6:1 diet ratio with solid food consisting of 48 grams of carbohydrates and MCT (using total of 100 mL MCT fat) in a step-wise manner. | - Regularly visited the outpatient clinic or were visited at home by the dietician for monitoring  - Required additional support (by phone and/or email).  -Feasibility was defined as the number of patients who could follow the KD for 3 months  -Safety was defined as the occurrence of adverse effects ≥2 grade, based on the common terminology criteria of adverse events (CTCAE).  -Adverse effects (e.g., gastrointestinal complaints and fatigue) were all noted in a study diary and evaluated weekly by the dietician.  -Ketone and glucose levels, growth parameters, and the patients gross motor functioning was scored using the Gross Motor Functioning Classification System (GMFCS) a self-designed, not validated, questionnaire of 15 questions administered at baseline and at the end of the study. We explored if and how parents and patients could cope with the KD. |
| 2 | 3 months | KD was initiated as a full liquid (LQ) KD formula with a 4:1 diet ratio used for a maximum of 2 weeks. | CKD supplemented with MCT emulsion, KD ratio (4:1→1.5:1-2:1). | - |  | LQ formula was carefully introduced with a maximum of four exchanges of 100 mL Ketocal LQ 4:1 ratio and 200 kcal and some snacks with total diet ratio of 3.5:1. When adequate ketosis was reached, the diet was modified into a KD of 1.75:1 diet ratio with solid foods and 35 g of carbohydrates and MCT in a step-wise manner. The MCT emulsion (at study termination, 75% of the calculated goal of 100 mL/day had been achieved) was well tolerated and he stopped vomiting. |  |
| 3 | 3 months | KD was initiated as a full liquid (LQ) KD formula with a 4:1 diet ratio used for a maximum of 2 weeks. | CKD supplemented with MCT emulsion, KD ratio (4:1→1.5:1-2:1). | Continued KD tube feeding |  | LQ formula was introduced to a maximum of 8.5 exchanges of 100 mL Ketocal LQ 4:1 ratio and 200 kcal and a little snack with an overall diet ratio of 3.8:1 in a step-wise manner. When adequate ketosis was reached, the diet was modified into a KD of 2.1:1 diet ratio with solid food consisting of 38 g of carbohydrates and MCT in a step-wise manner (using 10 mL MCT fat emulsion/day). |  |
| 4 | 12 months | 600 kcal (13% protein, 52% CHO, and 35% fat), in 50/50 TF of PediaSure and Enrich | CKD (MCT oil-based), KD ratio (1:1→ 2.5:1) supplemented with B-complex, Vit D, calcium, Iron, and other minerals. Energy requirements calculated as 120% RDA | Continued the diet for additional 12 months | 70 kcal/kg, 4 TSB of MCT oil, increased gradually to 85 kcal/kg and 5 TSB of MCT oil Formulas (Portagen and Pro-Mod) | MCT oil base KD (60% MCT oil, 20% protein, 10% CHO, and 10% dietary fat) Energy is 120% of the RDA to prevent weight lost. Supplements: B-complex, Vit D, calcium, Iron, and other minerals | -Diet introduced as an inpatient for 5 days.  - Patient discharged to continue the diet at home for 8 weeks.  -Weekly clinic visits.  -Patient used dietary manual containing recipes and food exchange lists were developed for each patient  -Families contacted by telephone to provide assistant |
| 5 | 8 weeks | 1900 kcal (17% protein, 43% CHO, and 40% fat). | CKD (MCT oil-based), KD ratio (1:1→ 2.5:1) supplemented with B-complex, Vit D, calcium, Iron, and other minerals. Energy requirements calculated as 120% RDA | - | KD provided 2200 kcal/ (88 kcal/kg) and 11.5 tsp MCT oil plus KD snacks such as shacks. Oil was added to scrambled eggs, tuna, and salad. |  |  |
| 6 | 2 years | Low-carbohydrate diet before diagnosis | Modified Atkin diet (<30‒35 g of carbohydrates with solid food and Ketocal | - | Modified Atkins diet at home. The main strategy of the diet was <30‒35 g of carbohydrates with solid food and Ketocal LQ 4:1 ratio. | - | -Followed-up by the hospital’s paediatric neurology/epilepsy team.  - Assessing ketone levels, blood glucose, serum lipids, serum uric acid, pH, renal stone, infections, liver enzymes, and weight change. |
| 7 | - | - | CKD ratio (1:1 →3.5:1) (whey protein and MCT emulsion with electrolyte supplementation during and after chemoradiation therapy). | -- | Ketogenic formula via a gastrostomy tube. KD was initiated at a 1:1 ratio, and increased to 3.5:1 ratio in a stepwise manner using KetoCal 4:1, whey protein and medium chain triglyceride emulsion with electrolyte supplementation during and after chemoradiation therapy. | - | Serum glucose, ketone levels, urine ketones, electrolytes and serum lipid profile, and serum carnitine |
| 8 | 3 months | - | CKD ratio (2.5:1) supplemented with MCT and 75% RDA | - | Started on 75% RDA calories 1800 Kcal and 2.5:1 keto ratio. Her calories and keto ratio were fine-tuned at each follow-up to maintain her weight and 4+ ketones. She was on low GI and consuming natural glutamine antagonist food products. She was on MCT oil 20 ml four times a day. | - | The clinical condition was assessed by the treating physician, and reported to be better under the diet, with substantial improvement of several symptoms  Routine tests for ketone levels, blood glucose, serum lipids, serum uric acid, pH, renal stone, infections, liver enzymes, and weight change. |
| 9 | 6months |  | CKD ratio (2.5:1) supplemented with MCT and 75% RDA | - | Ketogenic diet and started on 75% RDA calories 1200 Kcal and 2.5:1 keto ratio. Her calories and keto ratio were fine-tuned at each follow-up to maintain her weight and 4+ ketones | - |  |
| 10 | 3 months | - | CKD | Classic KD | Classic KD | After consulting Beth Zupec-Kania, a KD specialist, she corrected the calculation errors. |  |
| 11 | 12 months | - | Classic Atkins diet ratio (3.5:1) → changed to ERKD with a ratio of (3.5:1) and 80‒90% of energy. | - | Classical Atkin Diet | Changed to an ERKD with a ratio of 3.5:1 grams of fat to combined grams of protein and carbohydrates. 80 to 90% est. energy needed. |  |
