# Supplementary material for: Safety, Feasibility, and Effectiveness of Ketogenic Diet in Pediatric Patients With Brain Tumors: A Systematic Review
Source: J Nutr Metab. 2025 Mar 18;2025:7935879. doi: 10.1155/jnme/7935879 (PMC11936527; doi:10.1155/jnme/7935879)
Supplement: Supporting Information 3 — Table S3: Study outcomes. [file 7935879.f3.docx]

**TABLE S3 Study outcomes**

| Case | 1 | 2 | 3 | 4 | 5 | 6 | 7 | 8 | 9 | 10 | 11 |
| --- | --- | --- | --- | --- | --- | --- | --- | --- | --- | --- | --- |
| Ketosis and other lab parameters | On day 3, he showed hypoglycaemia (glucose level, 2.4 mmol/L) and hyperketosis (ketone level, 7.2 mmol/L). | Ketone and glucose levels were within normal range. | During the period of re-irradiation combined with steroids, the ketone levels two times decreased below the normal range of 2.3 mmol/L and 1.8 mmol/L, respectively. However, it normalized after the steroid was discontinued. | Increase in TG and FFA, slightly elevated in HDL-C, LDL-C, VLDL-C, but within normal limit | Decline in TG, slightly elevated in HDL-C, and decreased in LDL-C and VLDL-C | No hyperketosis or hypoglycaemia. Triglycerides were high. High total cholesterol: 12.40 mmol/L (range: 2.91‒5.36). Increased serum uric acid: slightly elevated, max 307 μmol/L. Chronic nephrocalcinosis since the start of the KD. Elevated ALAT at 22 unit/L once, probably related to KD.  One metabolic acidosis of pH 7.29 | Normal ketosis level.  Mean serum glucose before KD was 119 (98‒163) mg/dL and 79 (66‒95) mg/dL during KD. Mean serum ketone levels on KD was 31 (16‒59) mg/dL with moderate-to-large urine ketones. Electrolytes and serum lipid profile remained within normal limits with mild decrease in serum carnitine | No Hyperketosis or hypoglycaemia | No Hyperketosis or hypoglycaemia | - | Atkins 77.2. Keto-diet 60 to 85 mg/dl.  Serum ketone levels were above 2 or 3 mm |
| Side-effects | Fatigue, Vomiting, and Food refusal. | Fatigue, Vomiting, and Food refusal. | Fatigue, Constipation, and Inability to swallow | - | - | Fatigue, vomiting, food refusal, and constipation | - | Did not experience any documented adverse events while taking the diet (no hospitalizations were necessary). | Did not experience any documented adverse events while taking the diet (no hospitalizations were necessary). | - | - |
| Compliance | -KD with MCT was well tolerated without side effects.  -Questionnaire concerning diet application and daily monitoring (scored 2)  -use of LQ formula was difficult (scored 4)  -Parents stayed motivated during the study.  -Parents decided to discontinue the diet after the study. | -Parents expected no difficulties applying the diet in daily practice.  -Would be difficult for (score 5) them and their son to cope with the high level of monitoring and the use of LQ formula (score 4) because of his medical condition.  -Parents were motivated to start the study | -Questionnaire concerning diet application and daily monitoring (scored 1)  -Parents stayed motivated during the study | - KD established without difficulties  -Owing to sinus infection, the dietary compliance was affected. In week 4. | - KD established without difficulties  -towing to sinus infection, the dietary compliance was affected. In week 4. | Tolerable (stopped after 2 years of being well tolerated). | Ketogenic formula (KetoCal 4:1) via a gastrostomy tube (GTF), Tolerable | Tolerable | Tolerable | Tolerable | - |
| Tumor response | - | - | - | PET scan indicated decrease in FDG uptake by 21.77% and no changes in MRI | PET scan indicated decrease in FDG uptake by 21.84% | MRI appearance of the tumour improved. | MRI obtained at 10 and 16 weeks post KD initiation and following chemoradiation showed decreased tumour size. | - | - | 15% shrink in tumour size | 15% ↓tumour by MRI, |
| Neurological skills and quality of life | The GMFCS was level 3. Rapid neurological deterioration  follow-up he had bilateral abducens, facial and glossopharyngeal paresis, as well as a tetraparesis grade 2 on the right side and grade 1 on the left side. The GMFCS level  at 3 months was 5  -patient died 3.5 months after end of the KD study and 7 months after end of regular therapy | 3 weeks after the start of the KD study, he was admitted to the hospital with a generalized tonic–clonic seizure that easily responded to antiepileptic treatment.  MRI also showed extensive leptomeningeal metastases but no hydrocephalus. Within 24h, the level of consciousness decreased and he did not regain consciousness. His parents opted for a non-intervention policy, and the patient died 2 days later. | After 3 months of KD, after the study, the patient had a good language comprehension but could neither speak nor swallow and had a complete ocular paresis. He had left limb paralysis and limited use of the right hand, but just enough to use a communication device. At this time, the GMFCS grade was level 5.  Ketogenic nasogastric tube feeding was started in week 8 because of swallowing difficulty.  The patient died 3.5 months after the study | Progress in skill development (gait, mobility, speech, and hand coordination) and improvement in patient mood.  She enrolled in handicap school. Overall QoL improved. |  | 14 months after the end of treatment, the patient experienced a progression accompanied by a deteriorating clinical condition and steroid dependence. |  | Patient improved her clinical condition. She managed to walk without support, write, read, and dress. Speech improved (could return to school) | Patient improved her clinical condition. Asymptomatic (could return to kindergarten) while taking KD | Chemotherapy was discontinued by his oncologist after 3 months of implementing KD, and KD was continued. After several years, general health and neurological conditions improved with better QOL | ↑Vision, ↓hypothalamic obesity, ↑stamina, and ↑pituitary function |
| Nutrition growth | He showed no significant change in SD scores for weight for height (−0.35) or height for  age (+1.83) at the end of the study | Weight gain was not achieved; the SD score for weight for height remained at −3.0 | The SD score for weight for height stabilized at +2 and height for age was −0.41 | - | - | No change for body weight | - | - | - | - | BMI changed from 21 to 18 |
| Survival rate | (OSR of 16.5 months) | (OSR of 6.4 months). | (OSR of 18.7 months) | - | - | (OSR: 30 months). | - | (OSR: 9 months) | (OSR: 22 months) | - | - |
