# Supplementary material for: Safety, Feasibility, and Effectiveness of Ketogenic Diet in Pediatric Patients With Brain Tumors: A Systematic Review
Source: J Nutr Metab. 2025 Mar 18;2025:7935879. doi: 10.1155/jnme/7935879 (PMC11936527; doi:10.1155/jnme/7935879)
Supplement: Supporting Information 4 — Table S4: List of studies excluded from the review. [file 7935879.f4.pdf]

Feasibility of a modified Atkins diet in glioma patients during radiation and its effect on radiation sensitization

Ketogenic diets as an adjuvant therapy in glioblastoma (the KEATING trial): study protocol for a randomised pilot study

ERGO: A pilot study of ketogenic diet in recurrent glioblastoma

The Modified Ketogenic Diet in Adults with Glioblastoma: An Evaluation of Feasibility and Deliverability within the National Health Service

Targeting metabolism with a ketogenic diet during the treatment of glioblastoma multiforme

Metabolic management of glioblastoma multiforme using standard therapy together with a restricted ketogenic diet: Case Report

Isocaloric Ketogenic Diet in Adults with High-Grade Gliomas: A Prospective Metabolic Study

Efficacy of a ketogenic diet with concomitant intranasal perillyl alcohol as a novel strategy for the therapy of recurrent glioblastoma

ERGO2: A Prospective, Randomized Trial of Calorie-Restricted Ketogenic Diet and Fasting in Addition to Reirradiation for Malignant Glioma

Ketogenic diets as an adjuvant therapy for glioblastoma (KEATING): a randomized, mixed methods, feasibility study

A ketogenic diet exerts beneficial effects on body composition of cancer patients during radiotherapy: An interim analysis of the KETOCOMP study

Impact of a ketogenic diet intervention during radiotherapy on body composition: I. Initial clinical experience with six prospectively studied patients

Efficacy of Metabolically Supported Chemotherapy Combined with Ketogenic Diet, Hyperthermia, and Hyperbaric Oxygen Therapy for Stage IV Triple-Negative Breast Cancer

Management of Glioblastoma Multiforme in a Patient Treated With Ketogenic Metabolic Therapy and Modified Standard of Care: A 24-Month Follow-Up

Favorable Effects of a Ketogenic Diet on Physical Function, Perceived Energy, and Food Cravings in Women with Ovarian or Endometrial Cancer: A Randomized, Controlled Trial

Impact of modified short-term fasting and its combination with a fasting supportive diet during chemotherapy on the incidence and severity of chemotherapy-induced toxicities in cancer patients - a controlled cross-over pilot study

Modified Atkins diet in advanced malignancies - final results of a safety and feasibility trial within the Veterans Affairs Pittsburgh Healthcare System

A Phase I clinical trial of dose-escalated metabolic therapy combined with concomitant radiation therapy in high-grade glioma

Ketogenic Metabolic Therapy, Without Chemo or Radiation, for the Long-Term Management of IDH1-Mutant Glioblastoma: An 80-Month Follow-Up Case Report

Application of Bayesian evidence synthesis to modelling the effect of ketogenic therapy on survival of high grade glioma patients

Exploring the Feasibility and Effects of a Ketogenic Diet in Patients With CNS Malignancies: A Retrospective Case Series

4-year long progression-free and symptom-free survival of a patient with recurrent glioblastoma multiforme: A case report of the Paleolithic Ketogenic Diet (PKD) used as a stand-alone treatment after failed standard oncotherapy

Treatment of glioma patients with ketogenic diets: report of two cases treated with an IRB-approved energy-restricted ketogenic diet protocol and review of the literature

Short-term fasting in glioma patients: analysis of diet diaries and metabolic parameters of the ERGO2 trial

Cancer as a metabolic disease: on the origin, management, and prevention of cancer

Changes in cerebral metabolism during ketogenic diet in patients with primary brain tumors: 1H-MRS study

Glycemic modulation in neuro-oncology: experience and future directions using a modified Atkins diet for high-grade brain tumors

Does Metabolic management of gliomas using restricted Ketogenic diet combined with hyperbaric oxygen therapy (HBOT) improve clinical outcome and reduce epileptic risk? (Poster)

Ketogenic Diet With Radiation and Chemotherapy for Newly Diagnosed Glioblastoma

Feasibility and Biological Activity of a Ketogenic/Intermittent-Fasting Diet in Patients With Glioma

Ketogenic Diet as Adjunctive Treatment in Refractory/End-stage Glioblastoma Multiforme: a Pilot Study

Patient with recurrent glioblastoma responding favorably to ketogenic diet combined with intranasal delivery of perillyl alcohol: a case report and literature review

Tumor Metabolism, the Ketogenic Diet and β-Hydroxybutyrate: Novel Approaches to Adjuvant Brain Tumor Therapy

Fasting, Fats, and Physics: Combining Ketogenic and Radiation Therapy against Cancer

Investigating the ketogenic diet as treatment for primary Aggressive Brain cancer: challenges and Lessons Learned

Ketogenic Diet for Malignant Gliomas: a Review

Glycemic modulation in neuro-oncology: experience and future directions using a modified Atkins diet for high-grade brain tumors

Neutropenia in Children Treated With Ketogenic Diet Therapy

Impact of a ketogenic diet intervention during radiotherapy on body composition: I. Initial clinical experience with six prospectively studied patients

Effects of a ketogenic diet on the quality of life in 16 patients with advanced cancer: Apilot trial

Targeting insulin inhibition as a metabolic therapy in advanced cancer: A pilot safety and feasibility dietary trial in 10 patients

The development of tumours under a ketogenic diet in association with the novel tumour marker TKTL1: A case series in general practice

A Nutritional Perspective of Ketogenic Diet in Cancer: A Narrative Review

Impact of a ketogenic diet intervention during radiotherapy on body composition: I. Initial clinical experience with six prospectively studied patients

A Ketogenic Diet Reduces Central Obesity and Serum Insulin in Women with Ovarian or Endometrial Cancer

Consuming a Ketogenic Diet while Receiving Radiation and Chemotherapy for Locally Advanced Lung Cancer and Pancreatic Cancer: The University of Iowa Experience of Two Phase 1 Clinical Trials

Ketogenic Diets Enhance Oxidative Stress and Radio-Chemo-Therapy Responses in Lung Cancer Xenografts

Growth of human gastric cancer cells in nude mice is delayed by a ketogenic diet supplemented with omega-3 fatty acids and medium-chain triglycerides

A Ketogenic Formula Prevents Tumor Progression and Cancer Cachexia by Attenuating Systemic Inflammation in Colon 26 Tumor-Bearing Mice

Metabolic reprogramming induced by ketone bodies diminishes pancreatic cancer cachexia

Optimal Control Analysis of a Mathematical Model for Breast Cancer

Carbohydrate restriction, prostate cancer growth, and the insulin-like growth factor axis

The Effects of Varying Dietary Carbohydrate and Fat Content on Survival in a Murine LNCaP Prostate Cancer Xenograft Model

Beta-hydroxybutyrate (3-OHB) can influence the energetic phenotype of breast cancer cells, but does not impact their proliferation and the response to chemotherapy or radiation

Aldo–keto reductase family 1 B10 gene silencing results in growth inhibition of colorectal cancer cells: Implication for cancer intervention

Ketogenic *HMGCS2* Is a c-Myc Target Gene Expressed in Differentiated Cells of Human Colonic Epithelium and Down-Regulated in Colon Cancer

Mathematical modeling for optimal control of breast cancer

A Ketogenic Diet Is Acceptable in Women with Ovarian and Endometrial Cancer and Has No Adverse Effects on Blood Lipids: A Randomized, Controlled Trial

The Potential Use of a Ketogenic Diet in Pancreatobiliary Cancer Patients After Pancreatectomy

Effects of Ketogenic metabolic therapy on patients with breast cancer: A randomized controlled clinical trial

A ketogenic diet exerts beneficial effects on body composition of cancer patients during radiotherapy: An interim analysis of the KETOCOMP study

The Impact of a Ketogenic Dietary Intervention on the Quality of Life ofStage II and III Cancer Patients: A Randomized Controlled Trial inthe Caribbean

A ketogenic diet consumed during radiotherapy improves several aspects of quality of life and metabolic health in women with breast cancer

Impact of a ketogenic diet intervention during radiotherapy on body composition: III-final results of the KETOCOMP study for breast cancer patients

Low Carb and Ketogenic Diets Increase Quality of Life, Physical Performance, Body Composition, and Metabolic Health of Women with Breast Cancer

Ketogenic diets consumed during radio-chemotherapy have beneficial effects on quality of life and metabolic health in patients with rectal cancer

Cancer cachexia: influence of systemic ketosis on substrate levels and nitrogen metabolism

Total nutritional manipulation in humans: report of acancer patient

Effects of Pre-surgical Vitamin D Supplementation and Ketogenic Diet in a Patient with Recurrent Breast Cancer

Effect of energy substrate manipulation on tumour cell proliferation in parenterally fed cancer patients

Glucose-based total parenteral nutrition does not stimulate glucose uptake by humans tumours

Decline of Lactate in Tumor Tissue After Ketogenic Diet: In Vivo Microdialysis Study in Patients with Head and Neck Cancer

Reduction of weight loss and tumour size in a cachexia model by a high fat diet

Halted Progression of Soft Palate Cancer in a Patient Treated with the Paleolithic Ketogenic Diet Alone: A 20-months Follow-up

Clinical observation of a novel, complementary, immunotherapeutic approach based on ketogenic diet, chondroitin sulfate, vitamin D 3, oleic acid and a fermented milk and colostrum product

Halted progression of soft palate cancer in a patient treated with the paleolithic ketogenic diet alone

Efficacy of metabolically supported chemotherapy combined with ketogenic diet, hyperthermia, and hyperbaric oxygen therapy for stage IV triple-negative breast cancer

Complete cessation of recurrent cervical intraepithelial neoplasia (CIN) by the paleolithic ketogenic diet: a case report

Effects of a high-fat diet on body composition in cancer patients receiving chemotherapy: a randomized controlled study.

Metabolic Treatment of Cancer: Intermediate Results of a Prospective Case Series

The effects of short-term fasting on tolerance to (neo) adjuvant chemotherapy in HER2-negative breast cancer patients: a randomized pilot study

Differential effects of patient-related factors on the outcome of radiation therapy for rectal cancer

Favorable Effects of a Ketogenic Diet on Physical Function, Perceived Energy, and Food Cravings in Women with Ovarian or Endometrial Cancer: A Randomized, Controlled Trial

Feasibility study of metabolically supported chemotherapy with weekly carboplatin/paclitaxel combined with ketogenic diet, hyperthermia and hyperbaric oxygen therapy in metastatic non-small cell lung cancer

Ketogenic diets: from cancer to mitochondrial diseasesand beyond

Beneficial effects of ketogenic diets for cancer patients: a realist review with focus on evidence and confirmation

When less may be more: calorie restriction and response to cancer therapy

The infuence of diet on anti-cancer immune responsiveness

Selectively starving cancer cells through dietary manipulation: methods and clinical implications

The Pros and Cons of Low Carbohydrate and Ketogenic Diets in the Prevention and Treatment of Cancer

Perspective: Do Fasting, Caloric Restriction, and Diets Increase Sensitivity to Radiotherapy? ALiterature Review

The use of ketogenic diets in cancer patients: a systematic review

Restricted Calorie Ketogenic Diet for the Treatment of Glioblastoma Multiforme

SIGNIFICANCE OF LOW-CARBOHYDRATE DIETS AND FASTING IN PATIENTS WITH CANCER

Ketogenic Diet in Advanced Cancer: A Pilot Feasibility and Safety Trial in the Veterans Affairs Cancer Patient Population

Knowledge Translation of Low Carbohydrate Diet Intervention in Cancer Survivorship: From Basic Science to Clinical Practice and Policy Making

Ketogenic Diet in Cancer Prevention and Therapy: Molecular Targets and Therapeutic Opportunities

Wilhelm Brünings' forgotten contribution to the metabolic treatment of cancer utilizing hypoglycemia and a very low carbohydrate (ketogenic) diet

Complete Cessation of Recurrent Cervical Intraepithelial Neoplasia (CIN) by the Paleolithic Ketogenic Diet: A Case Report

Mitteilung: Klinische Anwendungen der dia¨tetisch-hormonalen Krebsbeeinflussung (‘‘Entzuckerungsmethode’’)

Insulin in der Krebsbehandlung

Cerebral Ketones Detected by 3T MR Spectroscopy in Patients with High-Grade Glioma on an Atkins-Based Diet

The Ketogenic Diet Is an Effective Adjuvant to Radiation Therapy for theTreatment of Malignant Glioma

Enhanced immunity in a mouse model of malignant glioma is mediated by a therapeutic ketogenic diet

The ketogenic diet reverses gene expression patterns and reduces reactive oxygen species levels when used as an adjuvant therapy for glioma

Differential utilization of ketone bodies by neurons and glioma cell lines: a rationale for ketogenic diet as experimental glioma therapy

The Ketogenic Diet Alters the Hypoxic Response and Affects Expression of Proteins Associated with Angiogenesis, Invasive Potential and Vascular Permeability in a Mouse Glioma Model

A Supplemented High-Fat Low-Carbohydrate Diet for the Treatment of Glioblastoma

Ketolytic and glycolytic enzymatic expression profiles in malignant gliomas: implication for ketogenic diet therapy

The Ketogenic Diet as an Adjuvant Therapy for Brain Tumors and Other Cancers

Drug/diet synergy for managing malignant astrocytoma in mice: 2-deoxy-D-glucose and the restricted ketogenic diet

The Ketogenic Diet Does Not Affect Growth of Hedgehog Pathway Medulloblastoma in Mice

The contribution of ketone bodies to glycolytic inhibition for the treatment of adult and pediatric glioblastoma

Ketogenic Diet in Refractory Childhood Epilepsy: Starting With a Liquid Formulation in an Outpatient Setting

Safety and Eectiveness of the Prolonged Treatment of Children with a Ketogenic Diet

Incidence of potential adverse events during hospital-based ketogenic diet initiation among children with drug-resistant epilepsy

The efficacy comparison of classic ketogenic diet and modified Atkins diet in children with refractory epilepsy: a clinical trial

Cognitive benefits of the ketogenic diet in patients with epilepsy: A systematic overview

Use of ketogenic diet therapy in infants with epilepsy: A systematic review and meta-analysis

Ketogenic diet effects on 52 children with pharmacoresistant epileptic encephalopathy: A clinical prospective study

A Prospective Study on Changes in Nutritional Status and Growth Following Two Years of Ketogenic Diet (KD) Therapy in Children with Refractory Epilepsy

Impact of the Ketogenic Diet on Linear Growth in Children: A Single-Center Retrospective Analysis of 34 Cases

Efficacy and Safety of a Ketogenic Diet in Children and Adolescents with Refractory Epilepsy—A Review

Ketogenic diet treatment of children in the intensive care unit: Safety, tolerability, and effectiveness

Short-term and long-term efficacy of classical ketogenic diet and modified Atkins diet in children and adolescents with epilepsy: A systematic review and meta-analysis

The ketogenic diet influences taxonomic and functional composition of the gut microbiota in children with severe epilepsy

A Systematic Review of the Quality of Life for Families Supporting a Child Consuming the Ketogenic Diet for Seizure Reduction

Efficacy of KetogenicDiet,ModifiedAtkinsDiet,andLowGlycemicIndex TherapyDietAmongChildrenWithDrug-ResistantEpilepsy ARandomizedClinical Trial

The Effect of Ketogenic Diet Treatment in Drug-resistant Epilepsies of Childhood

Ketogenic diet and other dietary treatments for epilepsy (reviw)

Cognitive and behavioral impact of the ketogenic diet in children and adolescents with refractory epilepsy: A randomized controlled trial

Can an early 24-hour EEG predict the response to the ketogenic diet? A prospective study in 34 children and adults with refractory epilepsy treated with the ketogenic diet

Valproate effect on ketosis in children under ketogenic diet

Growth of children on the ketogenic diet

The ketogenic diet for the treatment of childhood epilepsy: a randomised controlled trial

The development of tumours under a ketogenic diet in association with the novel tumour marker TKTL1: A case series in general practice

Ketogenic Diet Therapy in Infants: Efficacy and Tolerability

Ten-year single-center experience of the ketogenic diet: factors influencing efficacy, tolerability, and compliance

Use of the modified Atkins diet for treatment of refractory childhood epilepsy: a randomized controlled trial

Seizure control and biochemical profile on the ketogenic diet in young children with refractory epilepsy--Indian experience

Efficacy of 4:1 (classic) versus 2.5:1 ketogenic ratio diets in refractory epilepsy in young children: a randomized open labeled study

A randomized trial of classical and medium-chain triglyceride ketogenic diets in the treatment of childhood epilepsy

A randomized controlled trial of the ketogenic diet in refractory childhood epilepsy

The MCT-ketogenic diet as a treatment option in refractory childhood epilepsy: A prospective study with 2-year follow-up

Efficacy of the classic ketogenic and the modified Atkins diets in refractory childhood epilepsy

Ketogenic diet efficacy in the treatment of intractable epileptic spasms

Comparison of short- versus long-term ketogenic diet for intractable infantile spasms

Effectiveness of the ketogenic diet used to treat resistant childhood epilepsy in Scandinavia

Modified Atkins diet vs classic ketogenic formula in intractable epilepsy

The effect of the classical and medium chain triglyceride ketogenic diet on vitamin and mineral levels

Long-term follow-up of the ketogenic diet for refractory epilepsy: multicenter Argentinean experience in 216 pediatric patients

Long-term effects of a ketogenic diet in obese patients

The Ketogenic Diet and Sport A Possible Marriage?

Beneficial effects of ketogenic diet in obese diabetic subjects

Interest in the Ketogenic Diet Grows for Weight Loss and Type 2 Diabetes

Obesity treatment by very low-calorie-ketogenic diet at two years: reduction in visceral fat and on the burden of disease

Low-carb and ketogenic diets in type 1 and type 2 diabetes

A Low-Carbohydrate, Ketogenic Diet versus a Low-Fat Diet To Treat Obesity and Hyperlipidemia: A Randomized, Controlled Trial

Efficacy of ketogenic diet on body composition during resistance training in trained men: a randomized controlled trial

Impact of a 6-week non-energy-restricted ketogenic diet on physical fitness, body composition and biochemical parameters in healthy adults

Spanish Ketogenic Mediterranean diet: a healthy cardiovascular diet for weight loss

Application of a Ketogenic Diet in Children With Autistic Behavior: Pilot Study

Distinct Circadian Signatures in Liver and Gut Clocks Revealed by Ketogenic Diet

Effects of a ketogenic diet in overweight women with polycystic ovary syndrome

The Ketogenic Diet: Seizure Control Correlates Better With Serum β-Hydroxybutyrate Than With Urine Ketones

Dietary Fat, Ketosis, and Seizure Resistance in Rats on the Ketogenic Diet

Hepatic steatosis, inflammation, and ER stress in mice maintained long term on a very low-carbohydrate ketogenic diet

Middle and Long-Term Impact of a Very Low-Carbohydrate Ketogenic Diet on Cardiometabolic Factors: A Multi-Center, Cross-Sectional, Clinical Study

Comparison of a very low-calorie-ketogenic diet with a standard low-calorie diet in the treatment of obesity

A Ketogenic Diet Improves Mitochondrial Biogenesis and Bioenergetics via the PGC1α-SIRT3-UCP2 Axis

A high-fat, ketogenic diet induces a unique metabolic state in mice

Ketogenic Diets Alter the Gut Microbiome Resulting in Decreased Intestinal Th17 Cells

Selenium Deficiency Associated with Cardiomyopathy: A Complication of the Ketogenic Diet

The effect of a low-carbohydrate, ketogenic diet versus a low-glycemic index diet on glycemic control in type 2 diabetes mellitus

Dietary-Induced Ketogenesis: Adults Are Not Children

An Israeli tuberous sclerosis cohort: the efficacy of different anti-epileptic strategies

Tumor growth in patients with tuberous sclerosis complex on the ketogenic diet

Ketogenic diet treatment in adults with refractory epilepsy

Tuberous Sclerosis Complex and the Ketogenic Diet

Long-term outcomes of ketogenic diet in patients with tuberous sclerosis complex-derived epilepsy

The effects of the ketogenic diet in refractory partial seizures with reference to tuberous sclerosis

Ketogenic Diet for the Management of Epilepsy Associated with Tuberous Sclerosis Complex in Children

Long-term High Fat Ketogenic Diet Promotes Renal Tumor Growth in a Rat Model of Tuberous Sclerosis

Glucose deprivation in tuberous sclerosis complex-related tumors

Comparison of a very low-calorie-ketogenic diet with a standard low-calorie diet in the treatment of obesity

Very Low-Calorie Ketogenic Diet: A Safe and Effective Tool for Weight Loss in Patients with Obesity and Mild Kidney Failure

Short-Term Ketogenic Diet Improves Abdominal Obesity in Overweight/Obese Chinese Young Females

Metabolic impact of a ketogenic diet compared to a hypocaloric diet in obese children and adolescents

Effect of DHA supplementation in a very low-calorie ketogenic diet in the treatment of obesity: a randomized clinical trial

Effect of a Very-Low-Calorie Ketogenic Diet on Circulating Myokine Levels Compared with the Effect of Bariatric Surgery or a Low-Calorie Diet in Patients with Obesity

Body Composition Changes After Very-Low-Calorie Ketogenic Diet in Obesity Evaluated by 3 Standardized Methods

Very Low-Carbohydrate Ketogenic Diet for the Treatment of Severe Obesity and Associated Non-Alcoholic Fatty Liver Disease: The Role of Sex Differences

Ketogenic diet modifies the risk factors of heart disease in obese patients

The Effect of a Low-Carbohydrate, Ketogenic Diet on Nonalcoholic Fatty Liver Disease: A Pilot Study

Biochemical effect of a ketogenic diet on the brains of obese adult rats

Resting metabolic rate of obese patients under very low calorie ketogenic diet

Ketogenic diets as treatment of obesity and type 2 diabetes mellitus

A very low carbohydrate ketogenic diet improves glucose tolerance in ob/ob mice independently of weight loss

Effectiveness of a Very Low Calorie Ketogenic Diet on Testicular Function in Overweight/Obese Men

Effects of Very Low Calorie Ketogenic Diet on the Orexinergic System, Visceral Adipose Tissue, and ROS Production

MRI estimated changes in visceral adipose tissue and liver fat fraction in patients with obesity during a very low-calorie-ketogenic diet compared to a standard low-calorie diet

Energy expenditure and body composition changes after an isocaloric ketogenic diet in overweight and obese men

Very Low-Carbohydrate Ketogenic Diet Before Bariatric Surgery: Prospective Evaluation of a Sequential Diet

Comparing the Efficacy of Ketogenic Diet with Low-Fat Diet for Weight Loss in Obesity Patients: Evidenc-Based Case Report

Effects of a high-protein ketogenic diet on hunger, appetite, and weight loss in obese men feeding ad libitum1,

The effects of a low-carbohydrate, ketogenic diet on the polycystic ovary syndrome: A pilot study

Acid–base safety during the course of a very low-calorie-ketogenic diet

Efficacy of a 2-Month Very Low-Calorie Ketogenic Diet (VLCKD) Compared to a Standard Low-Calorie Diet in Reducing Visceral and Liver Fat Accumulation in Patients With Obesity

Effect of A Very Low-Calorie Ketogenic Diet on Food and Alcohol Cravings, Physical and Sexual Activity, Sleep Disturbances, and Quality of Life in Obese Patients

Weight loss, improved physical performance, cognitive function, eating behavior, and metabolic profile in a 12-week ketogenic diet in obese adults

Ketogenic Diet-Induced Weight Loss is Associated with an Increase in Vitamin D Levels in Obese Adults

The Effects of a Low Calorie Ketogenic Diet on Glycaemic Control Variables in Hyperinsulinemic Overweight/Obese Females

Timeline of changes in appetite during weight loss with a ketogenic diet

An observational study of sequential protein-sparing, very low-calorie ketogenic diet (Oloproteic diet) and hypocaloric Mediterranean-like diet for the treatment of obesity

Short-term feeding of a ketogenic diet induces more severe hepatic insulin resistance than an obesogenic high-fat diet

Resistance training in overweight women on a ketogenic diet conserved lean body mass while reducing body fat

Therapeutic role of low-carbohydrate ketogenic diet in diabetes

Energy expenditure and body composition changes after an isocaloric ketogenic diet in overweight and obese men: A secondary analysis of energy expenditure and physical activity

Induced and controlled dietary ketosis as a regulator of obesity and metabolic syndrome pathologies

Ketogenic Diet Acts on Body Remodeling and MicroRNAs Expression Profile

Ketogenic Diet Impairs FGF21 Signaling and Promotes Differential Inflammatory Responses in the Liver and White Adipose Tissue

Glucose and Lipid Homeostasis and Inflammation in Humans Following an Isocaloric Ketogenic Diet

Pre-operative Very Low Calorie Ketogenic Diet (VLCKD) vs. Very Low Calorie Diet (VLCD): Surgical Impact

Successful treatment of obesity and insulin resistance via ketogenic diet status post Roux-en-Y

Low glycemic index treatment for epilepsy in tuberous sclerosis complex

Metabolic impact of a ketogenic diet compared to a hypocaloric diet in obese children and adolescents
Reconciling Diabetes Management and the Ketogenic Diet in a Child with Pyruvate Dehydrogenase Deficiency
Successful Treatment of Type 1 Diabetes and Seizures With Combined Ketogenic Diet and Insulin
Interest in the Ketogenic Diet Grows for Weight Loss and Type 2 Diabetes

Epi	
Epi	
Epi	
Epi	
Epi	
Epi	
Epi	
Epi	
Epi	
Epi	
Epi	
Epi	
Epi	
Epi	
Epi	
Epi	
Epi	
Epi	
Epi	
Epi	
Epi	
Epi	
Cancer	non-brain
Epi	
Epi	
Epi	
Epi	
Epi	
Epi	
Epi	
Epi	
Epi	
Epi	
Epi	
Epi	
Epi	
Epi	
Epi	
Obesity	
Obesity	
DM and Obesity	
DM and Obesity	
CVS	
DM	
Obesity	
Sport	
Sport	
CVS and Obesity	
Autisim	
Liver	
Ovary	
Epi	
Epi	
Inflamation	
CVS	
Obesity	

Epi
genetic
Gut
CVS
DM
Mixed
TSC
TSC
Epi
TSC
TSC
TSC
TSC
TSC
TSC
Obesity
Obesity
Obesity
Obesity
Obesity
Obesity
Obesity
Liver
Obesity
Liver
Obesity
Obesity
DM and Obesity
Obesity
Obesity
Obesity
Obesity
Obesity
Obesity
Obesity
Obesity
Ovary
Obesity
Obesity
Obesity
Obesity
Obesity
Obesity
Obesity
Obesity
Obesity
Obesity
DM
Obesity
Obesity
Obesity
Obesity
Inflamation
Surgical
Obesity
TSC

DM
DM
DM
DM

Ketogenic diets as anadjuvant cancer therapy: History and potential mechanism	mechanisime and review human BC
Implementing a ketogenic diet based on medium-chain triglyceride oil in pediatric patients with cancer	Peds cancer Reivew
Calorieor Carbohydrate Restriction? The Ketogenic Dietas Another Option for Supportive Cancer Treatment	Letter
THE KETOGENIC DIET FOR PATIENTS WITH BRAIN TUMOURS: TWO PARALLEL RANDOMISED TRIALS	Meeting
The ketogenic diet for the treatment of malignant glioma	Review in glioma
Toward a cancer-specific diet	Review
Rationale, Feasibility and Acceptability of Ketogenic Diet for Cancer Treatment	SR(cancer)
Role of ketogenic metabolic therapy in malignant glioma: A systematic review	SR(cancer)
Systematic review: isocaloric ketogenic dietary regimes for cancer patients	SR(cancer)
Clinical Aspects of the Ketogenic Diet	Reivew (non cancer)
Ketogenic diet guidelines for infants with refractory epilepsy	guidelines (Epi)
Optimal clinical management of children receiving the ketogenic diet: Recommendations of the international ketogenic diet study group	recommendations (Epi)
THE EFFECT OF KETOGENIC DIET ON SURVIVAL AND QUALITY OF LIFE IN PATIENTS WITH MALIGNANT BRAIN TUMORS IN PALLIATIVE CARE	Abstract Peds BC
Safety and tumor inhibitory effect of ketogenic diet for pediatric patients with malignant brain tumors	Abstract Peds BC
HG-32.USEOFKETOGENICDIETASACOMPLIMENTARY METABOLICTHERAPYDURINGCHEMO-RADIATION THERAPYINA7YEAROLDFEMALEWITHGLIOBLASTOMA	Abstract Peds BC
Targeting energy metabolism in brain cancer through calorie restriction and the ketogenic diet	mechanisime and review human BC
Effect of Ketogenic Diets on Body Composition and Metabolic Parameters of Cancer Patients: A Systematic Review and Meta-Analysis	SR(cancer)
History of the ketogenic diet	Review in Epi
The Neuropharmacology of the Ketogenic Diet	Review in Epi
Ketogenic diet in cancer therapy	lab Review in cancer
Mitochondria: The ketogenic diet—A metabolism-based therapy	Review in general
Ketogenic Diet for Obesity: Friend or Foe?	Review in Obesity
The Ketogenic Diet: Evidence for Optimism but High-Quality Research Needed	Review in general
Ketogenic Diet: A New Light Shining on Old but Gold Biochemistry	Review in general
The Ketogenic Diet for Obesity and Diabetes—Enthusiasm Outpaces Evidence	Review in DM and Obesity
The ketogenic diet: Uses in epilepsy and other neurologic illnesses	Review in general
Is there a role for carbohydrate restriction in the treatment and prevention of cancer?	lab Review in cancer
Metabolic management of brain cancer	Lab Review in BC
The ketogenic diet: Pros and cons	Review in general
Dietary modifications for enhanced cancer therapy	Review in cancer
The management of very low-calorie ketogenic diet in obesity outpatient clinic: a practical guide	Review in Obesity
Dietary patterns and cancer risk	Review in cancer
How Does the Ketogenic Diet Work? Four Potential Mechanisms	Review in general
Mechanisms of action for the medium-chain triglyceride ketogenic diet in neurological and metabolic disorders	Review in general
Modulation of Cellular Biochemistry, Epigenetics and Metabolomics by Ketone Bodies. Implications of the Ketogenic Diet in the Physiology of the Organism and Pathological States	lab Review
Ketogenic diet in the treatment of cancer e Where do we stand?	lab Review
When less may be more: calorie restriction and response to cancer therapy	lab Review
Tumor Cells Growth and Survival Time with the Ketogenic Diet in Animal Models: A Systematic Review	lab Review
Anti-Tumor Effects of Ketogenic Diets in Mice: A Meta-Analysis	lab Review
Obesity and Cancer Mechanisms: Cancer Metabolism	Review
Ketogenic diets Boon or bane?	Review in general
Metabolic therapy: A new paradigm for managing malignant brain cancer	lab Review in BC
Beneficial effects of the ketogenic diet on nonalcoholic fatty liver disease: A comprehensive review of the literature	Review for non cancer
Ketosis and the Ketogenic Diet, 2010: Advances in Treating Epilepsy and Other Disorders	Review in Epi
Ketosis, ketogenic diet and food intake control: a complex relationship	Review in general
Ketogenic Diet in Alzheimer's Disease	Review in Alzheimer
The Ketogenic Diet as a Treatment Paradigm for Diverse Neurological Disorders	Review in Nuro
Calories, carbohydrates, and cancer therapy with radiation: exploiting the five R's through dietary manipulation	Review in cancer
Ketogenic diets for weight loss: A review of their principles, safety and efficacy	Review in Obesity
Ketogenic Diets and Pain	Review
Ketogenic Diets for Adult Neurological Disorders	Review in Nuro
Mechanisms of Ketogenic Diet Action	Review
Ketogenic Diet and Microbiota: Friends or Enemies?	lab Review
Impact of Different Types of Diet on Gut Microbiota Profiles and Cancer Prevention and Treatment	Review in diets
Impact of a Ketogenic Diet on Metabolic Parameters in Patients with Obesity or Overweight and with or without Type 2 Diabetes: A Meta-Analysis of Randomized Controlled Trials	Reivew in DM and Obesity
Tumor-derived lactate and myeloid-derived suppressor cells	lab Review

Efficacy and safety of very low calorie ketogenic diet (VLCKD) in patients with overweight and obesity: A systematic review and meta-analysis	Review in Obesity
Starvation, Stress Resistance, and Cancer	lab Review
Out of Warburg effect: An effective cancer treatment targeting the tumor specific metabolism and dysregulated pH	lab Review
Efficacy of the Ketogenic Diet as a Treatment Option for Epilepsy: Meta-analysis	Review in Epi
New insights into the mechanisms of the ketogenic diet	Review in Epi
Review of current evidence and clinical recommendations on the effects of low-carbohydrate and very-low-carbohydrate (including ketogenic) diets for the management of body weight and other cardiometabolic risk factors: A scientific statement from the National L	Review in Obesity
Dietary Approaches to Cancer Therapy	Review in cancer
Nutritional Ketosis and Mitohormesis: Potential Implications for Mitochondrial Function and Human Health	Review
Dietary fat: From foe to friend?	Review in general
Insulin–PI3K signalling: an evolutionarily insulated metabolic driver of cancer	Review in Endo
Ketogenic Diets: An Update for Child Neurologists	Review in Epi
Nutrition, inflammation and cancer	Reivew
Fasting and cancer: molecular mechanisms and clinical application	Lab Reivew in cancer
Effects of Ketogenic Diets on Cardiovascular Risk Factors: Evidence from Animal and Human Studies	Review in CVS
Implementing a low-carbohydrate, ketogenic diet to manage type 2 diabetes mellitus	Review in DM
Safety of low-carbohydrate diets	Reivew
Ketogenic diets new advances for metabolism-based therapies	Review in Epi
Low-carbohydrate diets: what are the potential short-and long-term health implications?	Reivew
The Ketogenic Diet: One Decade Later	Reivew
Cancer as a metabolic disease: implications for novel therapeutics	Lab Review in cancer
Aldo–Keto Reductase AKR1C1–AKR1C4: Functions, Regulation, and Intervention for Anti-cancer Therapy	Review in cancer
The ketogenic diet in infants – Advantages of early use	Review in Epi
How does the ketogenic diet induce anti-seizure effects?	Review in Epi
The ketogenic diet: metabolic influences on brain excitability and epilepsy	Review in Epi
Dietary treatment of epilepsy: rebirth of an ancient treatment	Review in Epi
Limited efficacy of the ketogenic diet in the treatment of highly refractory epileptic spasms	Review in Epi
Medium-chain Triglyceride Ketogenic Diet, An Effective Treatment for Drug-resistant Epilepsy and A Comparison with Other Ketogenic Diets	Review in Epi
Long-term monitoring of the ketogenic diet : Do's and Don'ts	Review in Epi
Safety and tolerability of the ketogenic diet used for the treatment of refractory childhood epilepsy: a systematic review of published prospective studies	SR in Epi
Ketogenic diet in endocrine disorders: Current perspectives	Review in Endo
Beyond weight loss: a review of the therapeutic uses of very-low-carbohydrate (ketogenic) diets	Review in Obesity
Effects of Ketogenic Diet for Breast Cancer Treatment. A Protocol for Randomized Controlled Clinical Tria	Protocol in non brain Cancer
Ketogenic Diet in Neuromuscular and Neurodegenerative Diseases	Review in Nuro
Targeting Cancer Metabolism: Dietary and Pharmacologic Interventions	Review in cancer
The mechanisms mediating the antiepileptic effects of the ketogenic diet, and potential opportunities for improvement with metabolism-altering drugs	Review in Epi
Advantages and Disadvantages of the Ketogenic Diet: A Review Article	Reivew
Use of the Ketogenic Diet in Adults	Reivew in adults
Ketogenic dietary therapy for epilepsy and other disorders: current perspectives	Review in Epi
Metabolic management of brain cancer.	Reivew
Critical Dietary Factors in Cancer Chemoprevention	Review in cancer
Effect of Ketogenic Diet on Quality of Life in Adults with Chronic Disease: A Systematic Review of Randomized Controlled Trials	Reivew in adult
Provocative Question: Should Ketogenic Metabolic Therapy Become the Standard of Care for Glioblastoma?	Lab Review in BC
Nutrition in Cancer: Evidence and Equality	Reivew
Ketogenic Diet and Health	Reivew
Therapeutic Roles of Ketogenic Diet	Reivew
Roles of Caloric Restriction, Ketogenic Diet and Intermittent Fasting during Initiation, Progression and Metastasis of Cancer in Animal Models: A Systematic Review and Meta-Analysis	Lab Reivew in cancer
Emerging Role of the Ketogenic Dietary Therapies beyond Epilepsy in Child Neurology	Review in Epi
Worldwide Dietary Therapies for Adults With Epilepsy and Other Disorders	Review in Epi in adult
Ketogenic Diets and Cancer: Emerging Evidence	Reivew
Ketogenic Diet	Reivew
A Clinical Perspective of Low Carbohydrate Ketogenic Diets: A Narrative Review	Reivew
Ketogenic diet for primary brain and spinal cord tumours (Protocol)	Review in BC (Protocol)
Metabolic effects of dietary approaches: ketone bodies & ketogenic diet	Reivew
Is the restricted ketogenic diet a viable alternative to the standard of care for managing malignant brain cancer?	lab Review in BC
Personalized Nutrition in Disrupting Cancer — Proceedings From the 2017 American College of Nutrition Annual Meeting	Reivew
Investigations of a Low Carbohy estigations of as a Possible Treatment for Malignant Brain Tumors	Review in BC

The ketogenic diet for the treatment of glioma: Insights from genetic profiling	Review in BC
Current and future strategies for the treatment of malignant brain tumors	Review in BC
Is the ketogenic diet effective in specific epilepsy syndromes?	Reivew
The management of very low-calorie ketogenic diet in obesity outpatient clinic: a practical guide	Reivew
Efficacy and safety of very low calorie ketogenic diet (VLCKD) in patients with overweight and obesity: A systematic review and meta-analysis	Reivew
The Ketogenic Diet for Obesity and Diabetes—Enthusiasm Outpaces Evidence	Reivew
Very-low-carbohydrate ketogenic diet v. low-fat diet for long-term weight loss: a meta-analysis of randomised controlled trials	Reivew
The Ketogenic Diet: Evidence for Optimism but High-Quality Research Needed	Reivew
Beneficial effects of the ketogenic diet on nonalcoholic fatty liver disease: A comprehensive review of the literature	Reivew
Ketogenic Diet: an Endocrinologist Perspective	Reivew
Scientific evidence underlying contraindications to the ketogenic diet: An update	Reivew
The Effect of Ketogenic-Diet on Health	Reivew
Obesity and tumor growth inflammation, immunity, and the role of a ketogenic diet	Reivew
Short-Term Physiological Effects of a Very Low-Calorie Ketogenic Diet: Effects on Adiponectin Levels and Inflammatory States	Reivew
A high-fat, ketogenic diet causes hepatic insulin resistance in mice, despite increasing energy expenditure and preventing weight gain	Reivew
Effect of the ketogenic diet on glycemic control, insulin resistance, and lipid metabolism in patients with T2DM: a systematic review and meta-analysis	Reivew
Interest in the Ketogenic Diet Grows for Weight Loss and Type 2 Diabetes	Reivew
Very low calorie ketogenic diets in overweight and obesity treatment: Effects on anthropometric parameters, body composition, satiety, lipid profile and microbiota	Reivew
A ketogenic diet for reducing obesity and maintaining capacity for physical activity hype or hope?	Reivew
Very-low-calorie ketogenic diet (VLCKD) in the management of metabolic diseases: systematic review and consensus statement from the Italian Society of Endocrinology (SIE)	Reivew
Ketogenic Diet in Neuromuscular and Neurodegenerative Diseases	Reivew
Ketogenic diets for weight loss: A review of their principles, safety and efficacy	Reivew
Treatment of Diabetes and Diabetic Complications With a Ketogenic Diet	Reivew
THE METABOLISM OF TUMORS IN THE BODY. BY OTTO WARBURG, FRANZ WIND, AND ERWI'N NEGELEIN.	lab Reivew
The ketogenic diet for the treatment of glioma: Insights from genetic profiling	lab Reivew
The Ketogenic Diet and Hyperbaric OxygenTherapy Prolong Survivalin Micewith Systemic Metastatic Cancer	lab Reivew
The Ketogenic Diet in the Treatment of Malignant Glioma: Mechanistic Effects on Hypoxia and Angiogenesis	lab Reivew
Reduced Pain and Inflammation in Juvenile and Adult Rats Fed a Ketogenic Die	lab Reivew
A Ketogenic Diet Extends Longevity and Healthspan in Adult Mice	not cancer lab
Mechanisms by Which Low Glucose Enhances the Cytotoxicity of Metformin to Cancer Cells Both In Vitro and In Vivo	lab Reivew
Non-Toxic Metabolic Management of Metastatic Cancer in VM Mice: Novel Combination of Ketogenic Diet, Ketone Supplementation, and Hyperbaric Oxygen Therapy	lab Reivew

Glioma Modified Atkins-based Diet in Patients With Glioblastoma (GLAD)

Strict Classic Ketogenic Diet as a Therapy for Recurrent or Progressive and Refractory Brain Tumors in Children

Strict Classic Ketogenic Diet as a Therapy for Recurrent or Progressive and Refractory Brain Tumors in Children

Therapeutic Targeting of Sex Differences in Pediatric Brain Tumor Glycolysis

Ketogenic Diet in Combination With Standard-of-care Radiation and Temozolomide for Patients With Glioblastoma

Ketogenic Diet in Children With Malignant or Recurrent/Refractory Brain Tumor

Feasibility Study of Modified Atkins Ketogenic Diet in the Treatment of Newly Diagnosed Malignant Glioma

Restricted Calorie Ketogenic Diet as a Treatment in Malignant Tumors

Ketogenic Diet Adjunctive to Salvage Chemotherapy for Recurrent Glioblastoma:a Pilot Study (KGDinrGBM)

Ketogenic Diet Treatment Adjunctive to Radiation and Chemotherapy in Glioblastoma Multiforme: a Pilot Study

Ketogenic Diet With Radiation and Chemotherapy for Newly Diagnosed Glioblastoma

Ketogenic Diet as Adjunctive Treatment in Refractory/End-stage Glioblastoma Multiforme: a Pilot Study (KGDinGBM)

Pilot Study of a Metabolic Nutritional Therapy for the Management of Primary Brain Tumors (Ketones)

Calorie-restricted, Ketogenic Diet and Transient Fasting During Reirradiation for Patients With Recurrent Glioblastoma (ERGO2)

Ketogenic Diets as an Adjuvant Therapy in Glioblastoma

Ketogenic Diet in Advanced Cancer

Ketogenic Diet for Recurrent Glioblastoma

(2020). "Locoregionally Delivered CAR T Cells Are Effective in CNS Cancer Models." Cancer Discov 10(7): OF12.
In pediatric central nervous system cancer models, locoregional CAR T-cell delivery was effective.

Abd Al Gawad, R. Y. and R. M. H. Hanafy (2021). "Success rate of three capping materials used in pulpotomy of primary molars: A randomized clinical trial." Saudi Dent J 33(7): 560-567.
Background: Carious primary molars, symptomless, or with reversible pulpitis are most frequently treated with pulpotomy to maintain arch integrity, otherwise they would be extracted. The present study was conducted to assess clinically and radiographically the success r

Abdullah, I. (2020). "Commentary: Altruism reveals a surreptitious life-threatening myxoma." JTCVS Tech 3: 238-239.

Adachi, I. and J. Heinle (2020). "Commentary: Evolution of pediatric living-donor lung transplant: Adaptation to unique environment." JTCVS Tech 3: 317-318.

Adachi, I., et al. (2020). "Atriopulmonary connection for mechanically assisted Fontan completion: Classic technique for modern strategy." JTCVS Tech 3: 307-309.

Adam, D. L., et al. (2021). "A 10-year-old girl with acute shortness of breath." Paediatr Child Health 26(5): 264-265.

Adekanmi, A. J., et al. (2020). "A 5-Year Review of Ultrasonographic Evaluation of Ocular Diseases at the University College Hospital Ibadan, South-West, Nigeria." Ann Ib Postgrad Med 18(2): 152-159.
Background: Orbito-ocular diseases are a major public health issue, often causing visual impairment with serious socioeconomic implications on individual lives. Ocular ultrasonography is an invaluable diagnostic tool when clinical examination of the ocular fundus is difficul

Adewole, S., et al. (2021). "Deep Learning Methods for Anatomical Landmark Detection in Video Capsule Endoscopy Images." Proc Future Technol Conf (2020) 1288: 426-434.
Video capsule endoscope (VCE) is an emerging technology that allows examination of the entire gastrointestinal (GI) tract with minimal invasion. While traditional endoscopy with biopsy procedures are the gold standard for diagnosis of most GI diseases, they are limited by

Agegnehu, A., et al. (2020). "Pediatric Febrile Urinary Tract Infection Caused by ESBL Producing Enterobacteriaceae Species." Biomed Res Int 2020: 6679029.
Background: Over the past decade, drug resistance pattern has worsened for many of the uropathogens due to overuse of antibiotics for empiric treatment. The burden of extended spectrum beta-lactamase (ESBL) producing Enterobacteriaceae associated urinary tract inf

Aguirre-Martinez, I. L. and A. Torrelo (2021). "Lipoatrophic panniculitis of children." Clin Dermatol 39(2): 220-228.
Idiopathic lipoatrophic panniculitis of children is a rare disease of childhood, characterized by repeated attacks of tender subcutaneous nodules followed by the development of permanent lipoatrophy, often seen on the arms and legs, in association with fever, malaise, and

Ahluwalia, N., et al. (2020). "Newborn With Severely Depressed Left Ventricular Function: Acute Myocardial Infarction in a Newborn." JACC Case Rep 2(12): 1837-1840.
We describe a rare case of spontaneous coronary artery thrombosis in a newborn leading to rapid severe ventricular dysfunction. Early diagnosis is critical and management strategies are varied including hemodynamic support with extracorporeal membrane oxygenation,

Ahmadi, A. R., et al. (2020). "Early Results of the Persian Registry of Cardiovascular Disease/Congenital Heart Disease (PROVE/CHD) in Isfahan." J Tehran Heart Cent 15(4): 158-164.
Background: In 2016, a prospective registry for pediatric patients with congenital heart disease (CHD) was established in Isfahan, Iran. Data on pediatric CHD in Iran are scant; accordingly, we aimed to report the early results of the Persian Registry Of cardioVascular diseasE

Akard, T. F., et al. (2021). "Improved Parent-Child Communication following a RCT Evaluating a Legacy Intervention for Children with Advanced Cancer." Prog Palliat Care 29(3): 130-139.
Although legacy-building is a priority for quality palliative care, research has rarely examined effects of legacy interventions in children, particularly their impact on parent-child communication.We examined the impact of a web-based legacy intervention on parent-child co

Akard, T. F. and M. J. Gilmer (2020). "Research Cooperative Groups in Pediatric Palliative Care Research." Palliat Med Rep 1(1): 321-325.
Research cooperative groups aim to facilitate collaborative and rigorous palliative care research. The purpose of this article is to (1) demonstrate how cooperative groups are taking formal and sustainable steps with commitment to pediatric palliative care research program

Akkawi El Edelbi, R., et al. (2021). "Estimation of body surface area in neonates, infants, and children using body weight alone." Int J Pediatr Adolesc Med 8(4): 221-228.
Background: The aim of this study was to use Body Surface Area (BSA) data calculated with the Mosteller equation to test potential new equations that estimate BSA using Body Weight (BW) alone in children aged 0-18 years.Mosteller's equation, the golden standard at ou

Akobeng, A. K., et al. (2021). "Gastrointestinal manifestations of COVID-19 in children: a systematic review and meta-analysis." Frontline Gastroenterol 12(4): 332-337.
Objectives: To summarise the published evidence on the gastrointestinal manifestations of COVID-19 in children and to determine the prevalence of gastrointestinal symptoms. Methods: In this systematic review and meta-analysis, we searched PubMed, Embase, CINAHL a

Al Haq, A. M., et al. (2021). "Gastrointestinal Basidiobolomycosis in pediatric patients: A diagnostic dilemma and management challenge." Int J Pediatr Adolesc Med 8(4): 212-220.
Introduction: Basidiobolomycosis is a rare fungal disease, lately appearing in the gastrointestinal system of pediatric patients. Their clinical presentation resembles that of lymphoma or granulomatous inflammations. This non-specific presentation makes Gastrointestinal Ba

Al Qadrah, B. H., et al. (2021). "Saudi pediatric residents' confidence in handling ethical situations and factors influencing it." Int J Pediatr Adolesc Med 8(3): 160-164.
Background: During their residency program, pediatric residents frequently face ethical challenges. The aim of the study is to evaluate the pediatric residents' knowledge and confidence to handle common ethical dilemmas during their training. Methods: This is a survey-ba

Al Shamrani, A., et al. (2021). "When is asthma not guilty?" Int J Pediatr Adolesc Med 8(4): 203-211.
Asthma is a common childhood condition. Its prevalence in Saudi Arabia is high, increasing, and could exceed 20% at the current trajectory. Asthma is a syndrome with different clinical presentations and phenotypes. Many conditions are often misdiagnosed as asthma beca

Alavi, A., et al. (2021). "Contact dermatitis: An important consideration in leg ulcers." Int J Womens Dermatol 7(3): 298-303.
The prevalence of chronic wounds is increasing with the aging population, with 1% to 2% of the worldwide population experiencing leg ulcers and positive patch tests reported in up to 75% of this population. With the introduction of modern dressings and compression the

AlAzmi, A., et al. (2021). "Off-Label Drug Use in Pediatric Out-Patient Care: A Multi-Center Observational Study." Hosp Pharm 56(6): 690-696.

Objective: Prescribing a drug for a child is not an easy task and requires using the best available evidence as a guide, especially when a drug is used off-label. The practice of prescribing a drug for off-label use is fairly widespread worldwide. The FDA does not regulate prescr

Aliu, R., et al. (2020). "Juvenile dermatomyositis in a 14-year old Nigerian girl." Arch Clin Cases 7(1): 5-9.

Juvenile Dermatomyositis is a rare idiopathic autoimmune and inflammatory myopathy and vasculopathy whose hallmarks are symmetrical proximal muscle weaknesses and a characteristic rash. Only few cases have been reported in West Africa subregion. We present a 14

Alkharashi, M. (2020). "Anomalous separation of the medial rectus muscle, abnormal separation into superior and inferior compartment, and surgical management." Saudi J Ophthalmol 34(4): 297-299.

We describe a case of an anomalous separation of the medial rectus muscle in an 18-month-old female undergoing strabismus eye muscle surgery for partially accommodative esotropia. During surgery and after hooking the medial rectus muscle, it was noted that the widt

Allemang, B. A., et al. (2020). "Transitions to Postsecondary Education in Young Adults with Hemoglobinopathies: Perceptions of Patients and Staff." Pediatr Qual Saf 5(5): e349.

The transition from high school to postsecondary education can be challenging for adolescents and young adults (AYAs) with chronic health conditions. AYAs with hemoglobinopathies, including sickle cell disease, are a particularly vulnerable group whose academic perforn

Almadani, H., et al. (2020). "Congenital Factor VII Deficiency in Association With Bicuspid Aortic Valve and Multicystic Dysplastic Kidney Disease in a Child." J Med Cases 11(9): 271-274.

Congenital factor VII deficiency is a rare bleeding disorder, with an incidence of 1:500,000. This case report describes an exceptionally unusual combination of congenital factor VII deficiency, multicystic dysplastic kidney disease and bicuspid aortic valve disease, in the same

Almadhi, N. A., et al. (2021). "Knowledge and perception of parents regarding halitosis in their children in Saudi Arabia." Saudi Dent J 33(7): 574-580.

Objective: To evaluate parents' knowledge about halitosis, and to compare parents' perception of halitosis in their children to the presence of halitosis clinically. Methods: Children between 3 and 8 years attended to Dental University Hospital, King Saud University, were sc

Almagor, T., et al. (2021). "High Prevalence of Hearing Impairment in Primary Congenital Hypothyroidism." Eur Thyroid J 10(3): 215-221.

Background: An association between hearing impairment (HI) and congenital hypothyroidism (CH) has been reported previously. However, in general, studies were retrospective and had small sample sizes, and the results were variable and inconclusive. The aim of our stuc

Almahdy, A., et al. (2021). "Using bonding agent prior to pits and fissure sealant application enhances the microtensile bond strength and the interface morphology." Saudi Dent J 33(7): 487-494.

Background: A pits and fissures sealant is an effective method for preventing dental caries. Using a bonding agent before applying the sealant may increase its retention. This study aimed to compare the microtensile strength (microTBS) of a fissure sealant with and withou

Al-Mahroos, F. T., et al. (2021). "Child maltreatment prevention readiness in Bahrain." Int J Pediatr Adolesc Med 8(3): 149-153.

Background and Objectives: Child maltreatment (CM) is a major public health problem worldwide. Despite the well-documented cases of all forms of CM and the improvement of the national response to CM in Bahrain, efforts on the prevention of CM are still limited. The c

Almajed, F., et al. (2020). "Congenital bilateral upper lid eversion." Saudi J Ophthalmol 34(4): 324-327.

We report two healthy Saudi newborns with congenital bilateral upper lid eversion evolving with good outcome using conservative treatment. The current literature including epidemiology, clinical characteristics, possible etiologic factors, and treatment was reviewed.

Almeida, J. F. F. and F. C. C. Campos (2021). "A two-step planning method to increase accessibility to medium complexity procedures for public secondary healthcare." Cien Saude Colet 26(9): 4287-4298.

The specialized care level of the public Brazilian national health system is critical and chronically underfunded. Few studies have evaluated public secondary care planning on a strategic level, so there are open issues yet to examine. This study aims at locating medical cente

Almuneef, M., et al. (2021). "Child mortality in Saudi Arabia: Time for action at all levels." Int J Pediatr Adolesc Med 8(3): 165-171.

Background and Objectives: Although child mortality is declining in Saudi Arabia, new trends and causes are emerging. The objective of the study is to determine the causes of child death in a tertiary care hospital in Saudi Arabia and to identify its preventable causes and as

Alomair, R., et al. (2020). "The prevalence and predictors of refractive error among school children in Riyadh, Saudi Arabia." Saudi J Ophthalmol 34(4): 273-277.

PURPOSE: Refractive error (RE) is one of the most common ocular disorders among children worldwide. This study aimed to investigate the prevalence of RE and possible risk factors among school children in Riyadh. METHODS: This is a cross-sectional study using data colle

Al-Radi, O. O. (2020). "Commentary: If it looks like a duct and it cracks like a duct, it most likely is a duct." JTCVS Tech 4: 247.

Alsabti, N. and N. Talic (2021). "Comparison of static friction and surface topography of low friction and conventional TMA orthodontic arch wires: An in-vitro study." Saudi Dent J 33(5): 268-275.

Background: Arch wire surface characteristics, especially surface roughness and topography, influence the coefficient of friction during sliding. The clinician should be familiar with the properties of orthodontic appliances and materials that could result in high friction to ma

Alsohime, F., et al. (2021). "Barriers to the delivery of enteral nutrition in pediatric intensive care units: A national survey." Int J Pediatr Adolesc Med 8(3): 186-190.

Background and Aim: According to previously reported studies in the literature, a significant number of patients do not receive enteral nutrition in pediatric intensive care unit (PICU) because of avoidable barriers. Optimal nutrition is a fundamental goal in PICU. This study

Alushani, D., et al. (2020). "Thoracoscopic resection of esophageal duplication causing tracheobronchomalacia in a 5-year-old patient." JTCVS Tech 4: 382-385.

AlZabli, S. M., et al. (2021). "Peritonitis in children on peritoneal dialysis: 12 years of tertiary center experience." Int J Pediatr Adolesc Med 8(4): 229-235.

Background and Objective: Peritoneal dialysis (PD) associated peritonitis is the most common cause of morbidity, mortality, and treatment failure in patients undergoing PD. We aimed to identify the incidence, pathogens, antibiotic susceptibility, and the outcome of perito

An, L., et al. (2021). "Bone morphogenetic protein 4 (BMP4) promotes hepatic glycogen accumulation and reduces glucose level in hepatocytes through mTORC2 signaling pathway." Genes Dis 8(4): 531-544.

Liver is an important organ for regulating glucose and lipid metabolism. Recent studies have shown that bone morphogenetic proteins (BMPs) may play important roles in regulating glucose and lipid metabolism. In our previous studies, we demonstrated that BMP4 signific

Anand, E., et al. (2021). "Effect of Neutralization of Gastric Aspirate on culture yield of mycobacterium tuberculosis in children with pulmonary tuberculosis." Indian J Tuberc 68(4): 431-436.

INTRODUCTION: Conventionally gastric aspirates are neutralized with sodium bicarbonate to improve the culture yield of MTB. However, only limited data is there to support this practice. The aim of this study was to compare the contamination rate, culture yield and time

Andersen, N. D., et al. (2021). "Commentary: Mechanical circulatory support for the failing Glenn circulation: Keep all options on the table and don't give up." JTCVS Tech 6: 149-150.

Andresen, J. H. and O. D. Saugstad (2020). "50 Years Ago in TheJournalofPediatrics: Wolf-Hirschorn Versus Cri-du-Chat Syndrome." J Pediatr 226: 95.

Andrews, E., et al. (2020). "Legacy Building in Pediatric End-of-Life Care through Innovative Use of a Digital Stethoscope." Palliat Med Rep 1(1): 149-155.

Background: Legacy making has been the focus of recent literature; however, few studies examine how legacy making affects bereaved parents. Objective: To better understand legacy making's effect on bereaved parents, this study examined (1) the presentation of legacy

Antabak, A., et al. (2020). "Treatment of Pediatric Femoral Fractures in the City of Zagreb." Acta Clin Croat 59(4): 686-695.

Femur fractures in children can be treated with a number of operative and conservative methods. Numerous factors determine which method is optimal for a specific fracture. The aim of this research was to analyze distribution of femur fractures in children living in the ur

Arai, Y., et al. (2020). "Successful recovery from severe hypertension in a patient with Leigh syndrome." Mol Genet Metab Rep 25: 100684.

Hypertension is a rare complication of Leigh Syndrome (LS), but prognosis of patients with hypertension is poor and its presence is indicative of the terminal stage of the disease. Herein, we report a four-year-old girl case diagnosed with LS at 15 months of age who subsequ

Asefa, Y., et al. (2020). "Sharps injuries prevention among healthcare providers in surgical, medical and pediatric wards of a public hospital in Jimma Zone: a best practice implementation project." JBI Evid Implement 19(3): 327-334.

BACKGROUND: Sharps injuries are a serious public health problem that healthcare providers face and constitute a major risk for the transmission of blood-borne infections. Prevention of sharps injuries in hospitals requires that healthcare providers comply with universal p

Aslankurt, M. and L. Aslan (2020). "Horner's syndrome secondary to heart surgery in a pediatric patient." Saudi J Ophthalmol 34(4): 303-305.

Horner's Syndrome (HS) is a disease characterized by miosis, ptosis, and ipsilateral lack of sweating. It can occur with any injury at the level of the ocular sympathetic system neurons from the hypothalamus to the cervical postganglionic fibers. We present here a case of HS

Assari, S. and T. J. Curry (2021). "Parental Education Ain't Enough: A Study of Race (Racism), Parental Education, and Children's Thalamus Volume." J Educ Cult Stud 5(1): 1-21.

Introduction: The thalamus is the hub of the brain and has a significant role in various brain activities. Purpose: This study explored racial differences in the association between parental education and thalamus volume among American children. Methods: Using data from

Asztalos, I. B., et al. (2021). "Development of a narrow-band imaging classification to reduce the need for routine biopsies of gastric polyps." Gastroenterol Rep (Oxf) 9(3): 219-225.

Background: Most incidental gastric polyps identified during upper endoscopy are considered low-risk. However, current guidelines recommend sampling all gastric polyps for histopathologic analysis. We aimed to devise a simple narrow-band imaging (NBI) classification to

Atashzadeh-Shoorideh, F., et al. (2021). "The obstacles to nurses being present with patients." Nurs Open 8(3): 1115-1124.

AIM: The aim of the present research was to investigate the obstacles, which prevent nurses being present with patients. BACKGROUND: It is vital for nurses to be able to spend time with patients for an accurate assessment of patients' needs to take place and to allow pati

Augustin, K., et al. (2018). "Mechanisms of action for the medium-chain triglyceride ketogenic diet in neurological and metabolic disorders." Lancet Neurol 17(1): 84-93.

High-fat, low-carbohydrate diets, known as ketogenic diets, have been used as a non-pharmacological treatment for refractory epilepsy. A key mechanism of this treatment is thought to be the generation of ketones, which provide brain cells (neurons and astrocytes) with

Aydin, M. D., et al. (2021). "New description of vagal nerve commanted intrapancreatic taste buds and blood glucose level: An experimental analysis." Bioimpacts 11(3): 181-185.

Introduction: There have been thousands of neurochemical mechanism about blood glucose level regulation, but intrapancreatic taste buds and their roles in blood glucose level has not been described. We aimed to investigate if there are taste buds cored neural networks

Babu, T., et al. (2021). "A Rare Case of Dapsone Hypersensitivity Syndrome and Leukemoid Reaction With Coexisting Hepatitis E in a Pediatric Patient." Hosp Pharm 56(4): 347-349.

Dapsone is extensively used for a variety of infectious, immunological, and hypersensitivity disorders. Dapsone can cause several adverse effects, the most serious being dapsone hypersensitivity syndrome (DHS), which is potentially fatal. DHS is characterized by triad of eru

Badke, C. M., et al. (2020). "Impact of an untrained CPR Coach in simulated pediatric cardiopulmonary arrest: A pilot study." Resusc Plus 4: 100035.

Aim: To determine if an untrained cardiopulmonary resuscitation (CPR) Coach, with no access to real-time CPR feedback technology, improves CPR quality. Methods: This was a prospective randomized pilot study at a tertiary care children's hospital that aimed to integrate

Bagdure, D. N., et al. (2021). "The Impact of Dedicated Cardiac Intensive Care Units on Outcomes in Pediatric Cardiac Surgery: A Virtual Pediatric Systems Database Analysis." J Pediatr Intensive Care 10(3): 174-179.

Care of children undergoing cardiac surgery occurs in dedicated cardiac intensive care units (CICU) or mixed intensive care units. In this article, we analyzed data from Virtual Pediatric Systems (VPS, LLC) database (2009-2014) for children < 18 years of age undergoing cardia

Baidwan, B. K., et al. (2020). "Severe Hypertriglyceridemia With New-Onset Type 1 Diabetes in Diabetic Ketoacidosis." J Med Cases 11(12): 426-428.

Hypertriglyceridemia is a complication in the presentation of diabetic ketoacidosis (DKA) but has been reported in the pediatric population infrequently. We report a 13-year-old female with new onset type 1 diabetes in DKA, who developed extreme hypertriglyceridemia.

Baker, A. M., et al. (2021). "Sexual Health Information Sources, Needs, and Preferences of Young Adult Sexual Minority Cisgender Women and Non-Binary Individuals Assigned Female at Birth." Sex Res Social Policy 18(3): 775-787.

Background: Young adult sexual minority women (SMW) have unique sexual health needs and higher rates of some poor sexual health outcomes compared to their heterosexual peers. Unequal access to relevant sexual health information may contribute to sexual orientat

Bamira, D. G., et al. (2020). "Unusual Cause of Severe Tricuspid Regurgitation: Tricuspid Leaflet Annular Tear Following Remote Motor Vehicle Accident." JACC Case Rep 2(14): 2156-2161.

Tricuspid regurgitation (TR) is an uncommon and underdiagnosed complication of blunt chest trauma. Typical mechanisms include torn chordae, papillary muscle rupture, and radial leaflet tear. We describe an unusual case of traumatic TR due to circumferential avulsion o

Banerjee, P., et al. (2021). "Every one-minute delay in EMS on-scene resuscitation after out-of-hospital pediatric cardiac arrest lowers ROSC by 5." Resusc Plus 5: 100062.

Objective: To determine which aspects of prehospital care impact outcomes after pediatric cardiac arrest. Methods: In this study, the authors examine 5 years of consecutive data from their county emergency medical system (EMS), to identify predictors of good outcome a

Barnett, M. L., et al. (2020). "Promoting Equity and Resilience: Wellness Navigators' Role in Addressing Adverse Childhood Experiences." Clin Pract Pediatr Psychol 8(2): 176-188.
Objective: Adverse childhood experiences (ACEs) have demonstrable negative effects on long-term physical and mental health. Racial and ethnic minority children disproportionally experience ACEs due to the impacts of structural inequality and discrimination, which could

Batra, A., et al. (2021). "Haploidentical Hematopoietic Stem Cell Transplantation in Leukemia's: Experience from a Cancer Center in India." Indian J Hematol Blood Transfus 37(3): 463-471.
There has been a surge in haploidentical hematopoietic stem cell transplantation (HSCT) in India recently. However, there is a paucity of data on haploidentical HSCT from India. The report is an analysis of data of haploidentical HSCT performed at our center. Analysis of pa

Bauer, D. F., et al. (2020). "Congress of Neurological Surgeons Systematic Review and Evidence-Based Guidelines on the Treatment of Pediatric Hydrocephalus: Update of the 2014 Guidelines." Neurosurgery 87(6): 1071-1075.
BACKGROUND: The Congress of Neurological Surgeons reviews its guidelines according to the Institute of Medicine's recommended best practice of reviewing guidelines every 5 yrs. The authors performed a planned 5-yr review of the medical literature used to develop the

Bawazir, O. A. and A. Bawazir (2021). "Ultrasound guidance for Port-A-Cath insertion in children; a comparative study." Int J Pediatr Adolesc Med 8(3): 181-185.
Background: Gaining vascular access in children is challenging. Ultrasound-guided central line insertion in adults became the standard of care; however, its role in children is not clear. Our objective was to evaluate the ultrasound-guided Port-A-Cath or totally implanted lon

Behan, M., et al. (2020). "A crossed-disciplinary evaluation of parental perceptions surrounding pediatric non-invasive brain stimulation research." Int J Pharm Healthc Mark 14(4): 623-640.
Purpose -: Recruitment for pediatric non-invasive brain stimulation (NIBS) studies is often challenged by low enrollment. Understanding parental perceptions regarding NIBS is crucial to develop new communication strategies to increase enrollment. Design/methodology/a

Bektas, I., et al. (2020). "Developing and Evaluating the Psychometric Properties of the Pediatric Nursing Competency Scale for Nursing Students." Florence Nightingale J Nurs 28(2): 133-142.
Aim: This study aims to develop and evaluate the psychometric properties of the Pediatric Nursing Competency Scale for nursing students. Methods: This study was conducted with 318 nursing students, including third-year students enrolled in a pediatric nursing course ar

Belczak, S. Q., et al. (2020). "Endovascular treatment of a teenager with nutcracker syndrome: a case report." J Vasc Bras 19: e20180126.
The nutcracker syndrome is caused by compression of the left renal vein by the superior mesenteric artery and aorta and is associated with characteristic symptoms, such as lower abdominal pain, varicocele, and hematuria. Diagnosis is often difficult and, therefore, is ofte

Belikina, D. V., et al. (2021). "COVID-19 in Patients with Diabetes: Clinical Course, Metabolic Status, Inflammation, and Coagulation Disorder." Sovrem Tekhnologii Med 12(5): 6-16.
The aim of the investigation was to study the clinical course of COVID-19 in the presence of diabetes mellitus (DM) and elucidate possible mechanisms of their mutual aggravation. Materials and Methods: The study included 64 patients with COVID-19; of them, 32 were wit

Belo, S., et al. (2021). "Stability of Pentobarbital Hydrogel for Rectal Administration in Pediatric Procedural Sedation." Hosp Pharm 56(4): 332-337.
Purpose: Pentobarbital is a sedative agent to limit children motion during computed tomography or magnetic resonance imaging (MRI) and ensures the successful completion of the imaging procedure. However, data on rectal drug formulation and its stability in practice ar

Berni, F., et al. (2021). "Generation of glucosylated sn-1-glycerolphosphate teichoic acids: glycerol stereochemistry affects synthesis and antibody interaction." RSC Chem Biol 2(1): 187-191.
Lipoteichoic acids (LTAs) have been addressed as possible antigen candidates for vaccine development against several opportunistic Gram-positive pathogens. The study of structure-immunogenicity relationship represents a challenge due to the heterogenicity of LTA extra

Bertoni, C. B., et al. (2020). "A Quality Improvement Approach to Reduce Unplanned Extubation in the NICU While Avoiding Sedation and Restraints." Pediatr Qual Saf 5(5): e346.
The unplanned extubation (UE), a common adverse event in the neonatal intensive care unit (NICU), may result in airway trauma, cardiopulmonary resuscitation, and, in extreme cases, death. As part of the Nationwide Children's Hospital NICU's effort to optimize NICU gra

Bhaskar, V. and P. Gupta (2020). "50 Years Ago in TheJournalofPediatrics: Best Practices to Control Fever in Children." J Pediatr 226: 35.

Bicalho, C. G., et al. (2021). "Hearing Loss in Mucopolysaccharidosis." Int Arch Otorhinolaryngol 25(3): e386-e391.
Introduction Mucopolysaccharidosis (MPS) is a set of rare diseases caused by deficiency of lysosomal enzymes that lead to the accumulation of glycosaminoglycans (GAG) in tissues and organs, which, in turn, is responsible for the multisystemic clinical, chronic, and progres

Bin-Shuwaish, M. S., et al. (2021). "An in vitro evaluation of microleakage of resin based composites bonded to chlorhexidine-pretreated dentin by different protocols of a universal adhesive system." Saudi Dent J 33(7): 503-510.
Purpose: This study compared microleakage of different resin based composite (RBC) materials bonded to dentin, after chlorhexidine (CHX) application, by different adhesion protocols of a universal adhesive system. Methods: Class V cavities were prepared on the buccal a

Blatt, N. B., et al. (2020). "Myeloperoxidase immunohistochemical staining can identify glomerular endothelial cell injury in dense deposit disease." Pediatr Nephrol 36(12): 4003-4007.
BACKGROUND: Previous studies have demonstrated residual complement-mediated deposits in repeat kidney biopsies of C3 glomerulopathies (C3G) (dense deposit disease (DDD) and C3 glomerulonephritis) following eculizumab treatment, despite some clinical improveme

Bover-Bauza, C., et al. (2021). "Long-term outcomes of necrotizing pneumonia." An Pediatr (Engl Ed) 95(5): 298-306.
INTRODUCTION: Necrotizing pneumonia (NP) is a serious complication of community-acquired pneumonia characterised by the destruction of normal lung parenchyma. No study has evaluated the repercussions of the lung damage in the years following the episode. The ai

Brault, J., et al. (2021). "Klinefelter's Syndrome with Maternal Uniparental Disomy X, Interstitial Xp22.31 Deletion, X-linked Ichthyosis, and Severe Central Nervous System Regression." J Pediatr Genet 10(3): 222-229.
We presented in this article a patient with Klinefelter syndrome (KS) (47,XXY) who had maternal nondisjunction and uniparental disomy of the X chromosome with regions of heterodisomy and isodisomy, an interstitial Xp22.31 deletion of both X chromosomes, and other p

Bruggeman, B., et al. (2021). "Barriers to retinopathy screening in youth and young adults with type 1 diabetes." Pediatr Diabetes 22(3): 469-473.
Early detection of diabetic retinopathy (DR) is imperative; however, adherence to screening guidelines is poor. We hypothesized that youth and young adults with type 1 diabetes (T1D) who met American Diabetes Association criteria for recommended DR screening at the

Brumfiel, C. M., et al. (2021). "Dermatologic manifestations of COVID-19-associated multisystem inflammatory syndrome in children." Clin Dermatol 39(2): 329-333.
Multisystem inflammatory syndrome in children (MIS-C) affects a small percentage of pediatric patients infected with COVID-19 and is characterized by fever, laboratory evidence of inflammation, multisystem involvement, and severe illness necessitating hospitalization. Sk

Burgoyne, A. M., et al. (2020). "A triple action CDK4/6-PI3K-BET inhibitor with augmented cancer cell cytotoxicity." Cell Discov 6(1): 49.

Buyck, M., et al. (2021). "CPR coaching during cardiac arrest improves adherence to PALS guidelines: a prospective, simulation-based trial." Resusc Plus 5: 100058.
Aim: Recent studies have shown that the integration of a trained cardiopulmonary resuscitation (CPR) Coach during resuscitation enhances the quality of CPR during simulated paediatric cardiac arrest. The objective of our study was to evaluate the effect of a CPR Coach or

Candan, T., et al. (2020). "Comparison of bilateral cerebral and somatic tissue oxygenation with near-infrared spectroscopy in cyanotic and acyanotic pediatric patients receiving cardiac surgery." Arch Med Sci Atheroscler Dis 5: e320-e331.
Introduction: Compromise of tissue oxygenation during surgery is associated with increased mortality and morbidity in the postoperative period in patients with congenital cardiac disorders. It may be monitored with near-infrared spectroscopy (NIRS). We aimed to evaluat

Carson, L. and J. E. Price, 2nd (2021). "Temporary Central Vision Blindness After Oseltamivir Administration in a 15-Year-Old Pediatric Male Positive for Influenza A." Hosp Pharm 56(6): 678-680.
Objective: A 15-year-old pediatric male patient was influenza A positive and started on oseltamivir at an outpatient clinic. Method: The next morning the patient presented to the emergency department (ED) with a chief complaint of visual disturbances including decreased

Cartledge, P. T., et al. (2020). "Antibiotic prescribing practices in three neonatology units in Kigali, Rwanda. - an observational study." Afr Health Sci 20(4): 1646-1654.
Introduction: There is limited published data on antibiotic use in neonatal units in resource-poor settings. Objectives: This study sought to describe antibiotic prescribing practices in three neonatology units in Kigali, Rwanda. Methods: A multi-center, cross-sectional study c

Casella, S. W., et al. (2020). "Rare neurological complication in an adolescent with kidney failure secondary to systemic lupus erythematosus: Answers." Pediatr Nephrol 36(12): 4093-4095.

Casella, S. W., et al. (2020). "Rare neurological complication in an adolescent with kidney failure secondary to systemic lupus erythematosus: Questions." Pediatr Nephrol 36(12): 4089-4091.

Castellanos, D. A., et al. (2020). "Left ventricular pseudoaneurysm after replacement of a Melody valve in the left atrioventricular valve position." JTCVS Tech 4: 248-250.

Cesar, R. G. and A. T. Rotta (2021). "Response from the Authors." J Pediatr Intensive Care 10(3): 240-242.

Chaix, M. A., et al. (2020). "Machine Learning Identifies Clinical and Genetic Factors Associated With Anthracycline Cardiotoxicity in Pediatric Cancer Survivors." JACC CardioOncol 2(5): 690-706.
Background: Despite known clinical risk factors, predicting anthracycline cardiotoxicity remains challenging. Objectives: This study sought to develop a clinical and genetic risk prediction model for anthracycline cardiotoxicity in childhood cancer survivors. Methods: We per

Chandnani, H. K., et al. (2021). "Endotracheal Tube Placement Confirmation with Bedside Ultrasonography in the Pediatric Intensive Care Unit: A Validation Study." J Pediatr Intensive Care 10(3): 180-187.
Critically ill patients who are intubated undergo multiple chest X-rays (CXRs) to determine endotracheal tube position; however, other modalities can save time, medical expenses, and radiation exposure. In this article, we evaluated the validity and interrater reliability of u

Chandran, R., et al. (2020). "Outcomes of Adult Ewing Sarcoma Treated with Multimodality Therapy: A Single-Institute Experience." South Asian J Cancer 9(4): 191-194.
Introduction Ewing sarcoma (ES) is more common in children and relatively rare in adults. Adult ES has poor prognosis than children. Treatment approaches for adults have been extrapolated from pediatric experience. Data on adult ES are very few because of its rarity in a

Chapman, K. E., et al. (2011). "Ketogenic diet in the treatment of seizures associated with hypothalamic hamartomas." Epilepsy Res 94(3): 218-221.
Seizures associated with hypothalamic hamartoma (HH) are notoriously intractable to medical therapy, and while surgical resection affords most affected patients with complete or near seizure-freedom, there remains a need to identify alternative treatments. In this retro:

Chaudhary, B. R., et al. (2020). "Study of Antibiotic Susceptibility among Bacterial Isolates in Neonatal Intensive Care Unit of a Tertiary Care Hospital: A Descriptive Cross-sectional Study." JNMA J Nepal Med Assoc 58(231): 893-899.
INTRODUCTION: Neonatal sepsis is a major cause of neonatal morbidity and mortality worldwide, especially in developing countries like Nepal. Antibiotic resistance among microorganisms poses new challenges in the treatment of neonatal sepsis. The present study is conc

Cheema, H., et al. (2020). "Genomic testing in 1019 individuals from 349 Pakistani families results in high diagnostic yield and clinical utility." NPJ Genom Med 5(1): 44.
We implemented a collaborative diagnostic program in Lahore (Pakistan) aiming to establish the genetic diagnosis, and to asses diagnostic yield and clinical impact in patients with suspected genetic diseases. Local physicians ascertained pediatric patients who had no previ

Chen, Y. C., et al. (1991). "[Lennox-Gastaut syndrome with band form heterotopia: a case report]." Zhonghua Yi Xue Za Zhi (Taipei) 48(3): 242-246.
A 10-year-old girl with developmental delay, mental retardation and intractable seizures is reported. She manifested clinically as Lennox-Gastaut syndrome with unknown etiology before admission. Brain MRI revealed a band-like lesion over the bilateral subcortical region

Cherian, T., et al. (2021). "Complete Peripheral Blast Clearance is Superior to the Conventional Cut-Off of 1000/microL in Predicting Relapse in Pediatric Pre-B Acute Lymphoblastic Leukemia." Indian J Hematol Blood Transfus 37(3): 366-371.
Risk-stratification has contributed to a dramatic improvement in survival in pediatric acute lymphoblastic leukemia (ALL). This study evaluated the utility of prephase response and day 15 bone marrow when a minimal residual disease (MRD) assessment was available. A file

Chernov, A. N., et al. (2020). "Related expression of TRKA and P75 receptors and the changing copy number of MYC-oncogenes determine the sensitivity of brain tumor cells to the treatment of the nerve growth factor in combination with cisplatin and temozolomide." Drug Metab Pe
OBJECTIVES: Oncological diseases are an urgent medical and social problem. The chemotherapy induces not only the death of the tumor cells but also contributes to the development of their multidrug resistance and death of the healthy cells and tissues. In this regard, the

Chiu, P., et al. (2020). "Neonatal repair of unilateral pulmonary vein atresia with associated coarctation of the aorta." JTCVS Tech 4: 277-279.

Chugani, C., et al. (2020). "Managing Student Suicidality on Campus: Perspectives from Diverse Student Affairs Staff." Qual Rep 25(9): 3224-3239.
Suicidal behavior is a substantial public health issue faced by college campuses. College counseling professionals often interact with a variety of other student affairs professionals who may be involved in the management of suicidality on campus. However, research on the

Chu-Shore, C. J. and E. A. Thiele (2010). "Tumor growth in patients with tuberous sclerosis complex on the ketogenic diet." Brain Dev 32(4): 318-322.

PURPOSE: New evidence is emerging that the availability of nutrients plays a key role in regulating the mammalian target of rapamycin complex-1 (mTORC1) signaling pathway in human cancers. Tuberous sclerosis complex (TSC) is a genetic disorder which results in the gro

Colon-Cortes, Y., et al. (2020). "Intra-tracheal delivery of AAV6 vectors results in sustained transduction in murine lungs without genomic integration." Gene 763S: 100037.
Despite the progress made in AAV-based gene therapy targeting different organ systems, lung-targeted gene therapy using AAV vectors has not been effective, mostly due to the poor transduction and un-sustained gene expression in airway epithelium. Furthermore, conc

Coman, D., et al. (1993). Fumarate Hydratase Deficiency. GeneReviews((R)). M. P. Adam, H. H. Ardinger, R. A. Pagon et al. Seattle (WA).
CLINICAL CHARACTERISTICS: Fumarate hydratase (FH) deficiency results in severe neonatal and early infantile encephalopathy that is characterized by poor feeding, failure to thrive, hypotonia, lethargy, and seizures. Dysmorphic facial features include frontal bossing, depre

Corbett, K. L., et al. (2021). "Does Non-Neurologic Multiorgan Dysfunction After Out-of-Hospital Cardiac Arrest among Children Admitted in Coma Predict Outcome 1 Year Later?" J Pediatr Intensive Care 10(3): 188-196.
In this article, we investigated whether non-neurologic multiorgan dysfunction syndrome (MODS) following out-of-hospital cardiac arrest (OHCA) predicts poor 12-month survival. We conducted a secondary data analysis of therapeutic hypothermia after pediatric cardiac a

Cordo, V., et al. (2021). "T-cell Acute Lymphoblastic Leukemia: A Roadmap to Targeted Therapies." Blood Cancer Discov 2(1): 19-31.
T-cell acute lymphoblastic leukemia (T-ALL) is an aggressive hematologic malignancy characterized by aberrant proliferation of immature thymocytes. Despite an overall survival of 80% in the pediatric setting, 20% of patients with T-ALL ultimately die from relapsed or refra

Correa, C. C., et al. (2021). "Quality of the Italian Websites for Parental Guidance on the Indications for Tonsillectomy in Children." Int Arch Otorhinolaryngol 25(3): e446-e452.
Introduction The quality of information on websites about tonsillectomy regarding the knowledge level may be low. Tonsillectomy is a surgical procedure to hypertrophy of the palatine and pharyngeal tonsils. So, it is an invasive procedure with possible complications, whic

Cruz, A. I., Jr., et al. (2020). "Failure Rates of Autograft and Allograft ACL Reconstruction in Patients 19 Years of Age and Younger: A Systematic Review and Meta-Analysis." JB JS Open Access 5(4).
Background: Graft choice for pediatric anterior cruciate ligament reconstruction (ACLR) is determined by several factors. There is limited information on the use and outcomes of allograft ACLR in pediatric patients. The purpose of this systematic review and meta-analysis w

Custer, J. W., et al. (2021). "Bedside Rounds in Intensive Care Units during the COVID-19 Pandemic and Beyond." J Pediatr Intensive Care 10(3): 210-215.
A survey-based pilot study was performed to examine the feasibility of videoconferencing to facilitate multidisciplinary rounds following the initiation of strict isolation and social distancing policies in a pediatric intensive care unit (PICU). The use of a mobile workstation wa

D'Alessandro, M., et al. (2021). "Factors associated with treatment failure of high-flow nasal cannula among children with bronchiolitis: a single-centre retrospective study." Paediatr Child Health 26(5): e229-e235.
Objectives: Bronchiolitis is the most common viral lower respiratory tract infection in children under age 2 for which high-flow nasal cannula (HFNC) is increasingly used. Understanding factors associated with HFNC failure is important to identify patients at risk for respirato

Dang, M. T., et al. (2015). "The Ketogenic Diet Does Not Affect Growth of Hedgehog Pathway Medulloblastoma in Mice." PLoS One 10(7): e0133633.
The altered metabolism of cancer cells has long been viewed as a potential target for therapeutic intervention. In particular, brain tumors often display heightened glycolysis, even in the presence of oxygen. A subset of medulloblastoma, the most prevalent malignant brain

Daubman, B. R., et al. (2020). "Best Practices for Teaching Clinicians to Use a Serious Illness Conversation Guide." Palliat Med Rep 1(1): 135-142.
With the palliative care workforce shortage and changes in advance care planning reimbursement, many institutions are requesting that palliative care specialists provide serious illness communication training across their institution's workforce. Based on our experience tr

Dave, D., et al. (2021). "Intergenerational Effects of Welfare Reform: Adolescent Delinquent and Risky Behaviors." Econ Inq 59: 199-216.
This study investigates effects of welfare reform in the United States on the next generation. Most previous studies of effects of welfare reform on adolescents focused on high-school dropout of girls or fertility; little is known about how welfare reform has affected other te

Davies, R. R., et al. (2021). "Using virtual reality simulated implantation for fit-testing pediatric patients for adult ventricular assist devices." JTCVS Tech 6: 134-137.

de la Llana, R. A., et al. (2021). "Merging Two Hospitals: The Effects on Pediatric Extracorporeal Cardiopulmonary Resuscitation Outcomes." J Pediatr Intensive Care 10(3): 202-209.
In this article, a retrospective study was performed to describe the impact of merging two pediatric intensive care units on the overall and neurocognitive outcomes of children who required extracorporeal cardiopulmonary resuscitation (ECPR). Results from three cohorts w

de Oliveira, A. B., et al. (2021). "Photobiomodulation in the treatment of xerostomia associated with hyposalivation in a pediatric patient with systemic scleroderma." Autops Case Rep 11: e2020220.
Scleroderma is a rare autoimmune disease characterized by excessive collagen production. The oral manifestations of the patient with scleroderma can include microstomia, xerostomia, and changes in the resorption teeth. We report the case of a 7-year-old female patient

Disotuar, M. M., et al. (2021). "Facile synthesis of insulin fusion derivatives through sortase A ligation." Acta Pharm Sin B 11(9): 2719-2725.
Insulin derivatives such as insulin detemir and insulin degludec are U.S. Food and Drug Administration (FDA)-approved long-acting insulin currently used by millions of people with diabetes. These derivatives are modified in C-terminal B29 lysine to retain insulin bioactivity.

Divekar, A. A. and V. A. Sebastian (2020). "Neonatal repair of persistent fifth aortic arch and aortic coarctation." JTCVS Tech 4: 245-246.

Dong, J., et al. (2020). "Coupling Nanostructured Microchips with Covalent Chemistry Enables Purification of Sarcoma-Derived Extracellular Vesicles for Downstream Functional Studies." Adv Funct Mater 30(49).
Tumor-derived extracellular vesicles (EVs) play essential roles in intercellular communication during tumor growth and metastatic evolution. Currently, little is known about the possible roles of tumor-derived EVs in sarcoma because the lack of specific surface markers mak

Donuru, A., et al. (2021). "Gunshot-Related Pediatric Left Ventricular Apical Aneurysm." JACC Case Rep 3(1): 26-30.
Penetrating injuries of the thorax and abdomen, such as gunshot and stabbing, are rare in children. We present the case of a pediatric patient with a history of remote gunshot injury presenting with a late aneurysm in the left ventricle. (Level of Difficulty: Intermediate.).

Dotis, J., et al. (2020). "Congenital ulcerated subcutaneous fat necrosis in a newborn - an unusual entity." Hippokratia 24(3): 144.

Duarte, V. E., et al. (2020). "Transcatheter Pulmonary Valve Performance During Pregnancy and the Postpartum Period." JACC Case Rep 2(6): 847-851.
Increasing numbers of women with congenital heart disease are undergoing pregnancy after transcatheter pulmonary valve replacement (TPVR). We present the course of 9 pregnancies in 7 women with TPVR, noting pre-pregnancy, antepartum, and postpartum gradients,

Duff, J., et al. (2021). "Engaging parents of hospitalized neonates during a pandemic." J Neonatal Nurs 27(3): 185-187.
Background: Engaging families through patient- and family-centered care (PFCC), the NICU nurse upholds the core concepts providing holistic care. The novel coronavirus (COVID-19) pandemic altered the daily routine of visiting parents to hospitals around the nation, parti

Duffy, E. Y., et al. (2021). "Opportunities to improve cardiovascular health in the new American workplace." Am J Prev Cardiol 5: 100136.
Adult working-class Americans spend on average 50% of their workday awake time at their jobs. The vast majority of these jobs involve mostly physically inactive tasks and frequent exposure to unhealthy food options. Traditionally, the workplace has been a challenging en

Duineveld, C., et al. (2021). "Outcome of atypical haemolytic uraemic syndrome relapse after eculizumab withdrawal." Clin Kidney J 14(8): 1939-1945.
Background: The introduction of eculizumab has significantly improved the outcome of patients with atypical haemolytic uraemic syndrome (aHUS). Because of the risk of relapse after discontinuation, eculizumab was proposed as life-long therapy. However, data on the ou

Eckert, M. A., et al. (2020). "The Topology of Pediatric Structural Asymmetries in Language-Related Cortex." Symmetry (Basel) 12(11).
Structural asymmetries in language-related brain regions have long been hypothesized to underlie hemispheric language laterality and variability in language functions. These structural asymmetries have been examined using voxel-level, gross volumetric, and surface area

Ediger, K., et al. (2021). "The curious case of the bleeding twins: Neonatal bleeding secondary to acetylsalicylic acid prescribed for preeclampsia prevention." Paediatr Child Health 26(5): 274-275.

Eghtesady, P. (2020). "Commentary: Living related lung transplantation in children." JTCVS Tech 3: 315-316.

El-Khuffash, A., et al. (2020). "Early targeted patent ductus arteriosus treatment in premature neonates using a risk based severity score: study protocol for a randomised controlled trial (PDA RCT)." HRB Open Res 3: 87.
A patent ductus arteriosus (PDA) in preterm infants is associated with increased ventilator dependence and chronic lung disease, necrotizing enterocolitis, intraventricular haemorrhage, and poor neurodevelopmental outcome. Randomised controlled trials of early PDA tre

Ellsworth, L., et al. (2020). "Lactational exposure to polychlorinated biphenyls is higher in overweight /obese women and associated with altered infant growth trajectory: A pilot study." Curr Res Toxicol 1: 133-140.
Background: Infant exposure to environmental chemicals, such as polychlorinated biphenyls (PCBs), may contribute to developmental programming of long-term metabolic disease risk. PCBs persist given their lipophilicity and long half-lives, allowing them to bio-accumulat

El-Saiedi, S. A., et al. (2021). "Novel biomarkers for subtle myocardial involvement in type I diabetes mellitus." Cardiovasc Endocrinol Metab 10(3): 175-181.
Background: Evaluation of certain biomarkers could be used to predict left ventricular (LV) and right ventricular (RV) function impairment in children with type 1 diabetes mellitus. The aim of this study was to determine the best cardiac biomarker for prediction of diabetic

Emani, S. M. (2021). "Commentary: In pursuit of a pediatric heart valve that can grow with the child." JTCVS Tech 5: 87-88.

Emil, G. E., et al. (2020). "Effects of body weight and posture on pulmonary functions in asthmatic children." Afr Health Sci 20(4): 1777-1784.
Background: Asthma is one of the most common chronic illnesses in the world. Pulmonary function tests are important tools in monitoring of asthmatic patients. There is need for investigating if spirometric indices were affected by body weight or posture or not. Objective

Eor, J. Y., et al. (2021). "Gut microbiota modulation by both Lactobacillus fermentum MSK 408 and ketogenic diet in a murine model of pentylenetetrazole-induced acute seizure." Epilepsy Res 169: 106506.
PURPOSE: Seizures are a threat to the host brain and body and can even cause death in epileptic children. Ketogenic diet (KD) is suggested for children suffering from epileptic seizures and has been investigated for its anti-seizure effect. However, the relationships betweer

Fallah, A., et al. (2016). "Cost-utility analysis of competing treatment strategies for drug-resistant epilepsy in children with Tuberous Sclerosis Complex." Epilepsy Behav 63: 79-88.
BACKGROUND: The management of drug-resistant epilepsy in children with Tuberous Sclerosis Complex (TSC) is challenging because of the multitude of treatment options, wide range of associated costs, and uncertainty of seizure outcomes. The most cost-effective approa

Farooqi, K. M. and D. Kalfa (2021). "Commentary: Virtual reality in presurgical planning: The future is already here." JTCVS Tech 6: 138-139.

Farra, C., et al. (2021). "17p13.3 Microduplication Syndrome: Further Delineating the Clinical Spectrum." J Pediatr Genet 10(3): 239-244.
17p13.3 microduplication syndrome has been associated with a clinical spectrum of phenotypes, and depending on the genes involved in the microduplication, it is categorized into two classes (Class I and Class II). We herein, describe two patients diagnosed with Class I 17

Fattahi, A., et al. (2020). "Aspergillus flavus endocarditis and meningitis in a child with marfan syndrome." Curr Med Mycol 6(4): 70-74.
Background and Purpose: Aspergillus species are implicated as the etiology of approximately 26% of endocarditis cases. Central nervous system aspergillosis is a life-threatening condition that has a mortality rate of 80%. Case report: Herein, we report a four- year- old fema

Feldkotter, M., et al. (2020). "Correction to: Endurance-oriented training program with children and adolescents on maintenance hemodialysis to enhance dialysis efficacy-DiaSport." Pediatr Nephrol 36(12): 4013-4014.

Ferretti, E., et al. (2021). "Extremely low gestational age infants: Developing a multidisciplinary care bundle." Paediatr Child Health 26(6): e240-e245.
Background: Clinical experience in managing extremely low gestational age infants, particularly those born <24 weeks' gestation, is limited in Canada. Our goal was to develop a bedside care bundle for infants born <26 weeks' gestation, with special considerations for infan

First, L. R. and A. R. Kemper (2020). "Seeing the Wellness in Our Patients and Ourselves Through the 2020 Lens of Pediatrics." Pediatrics 145(1).

Fister, N., et al. (2021). "Intraoperative Cardiac Arrest: Immediate Treatment and Diagnostic Evaluation." J Med Cases 12(1): 18-22.
Although perioperative cardiac arrest during anesthetic care in infants and children is a rare event, its consequences can be devastating. Risk factors associated with perioperative cardiac arrest include cardiac surgery, younger age, presence of comorbid conditions and em

Flanders, T. M., et al. (2020). "In Reply: Detailed Analysis of Hydrocephalus and Hindbrain Herniation After Prenatal and Postnatal Myelomeningocele Closure: Report From a Single Institution." Neurosurgery 87(6): E728.

Forsch, N., et al. (2021). "Computational analysis of cardiac structure and function in congenital heart disease: Translating discoveries to clinical strategies." J Comput Sci 52.

Increased availability and access to medical image data has enabled more quantitative approaches to clinical diagnosis, prognosis, and treatment planning for congenital heart disease. Here we present an overview of long-term clinical management of tetralogy of Fallot (TC

Frange, P., et al. (2020). "Preventing respiratory syncytial virus infections in hospitalized children and adults: should we do better?" Infect Prev Pract 2(2): 100041.

Objective: To compare the burden of nosocomial and community-acquired respiratory syncytial virus (RSV)-associated acute lower respiratory tract infections (ALRIs) in adult and pediatric patients concomitantly admitted to a French tertiary hospital, and to evaluate the ef

Freeman, J. M. and E. H. Kossoff (2010). "Ketosis and the ketogenic diet, 2010: advances in treating epilepsy and other disorders." Adv Pediatr 57(1): 315-329.

Garai, J., et al. (2020). Biomarkers of Gastric Premalignant Lesions. Advancing the Science of Cancer in Latinos. A. G. Ramirez and E. J. Trapido. Cham (CH): 81-88.

Gastric cancer is one of the most common and deadliest cancers worldwide. There is a significant diversity in risk and aggressiveness associated with the disease in terms of factors such as ethnicity, age, and diet. However, one thing that is common is the fact that infection

Garcia-Almaraz, R., et al. (2021). "Prevalence and risk factors associated with allergic rhinitis in Mexican school children: Global Asthma Network Phase I." World Allergy Organ J 14(1): 100492.

Background: The International Study of Asthma and Allergies in Childhood (ISAAC) showed a wide variability in prevalence and severity of allergic rhinitis (AR) and rhinoconjunctivitis (ARC), in addition to other atopic diseases (Asher et al, 2006).(1) The Global Asthma Netwo

Garcia-Dominguez, M., et al. (2020). "Acute Lymphoblastic Leukemia Following Incomplete Kawasaki Disease." J Med Cases 11(11): 352-354.

Kawasaki disease (KD) is a multisystemic vasculitis of unknown etiology, typically affecting children younger than 5 years of age. A direct relationship between KD and the development of malignant tumors has not been demonstrated, however, the immunological alteratio

Garg, U., et al. (2020). "Solving an unusual case of acute kidney injury: Answers." Pediatr Nephrol 36(12): 4137-4140.

Garg, U., et al. (2020). "Solving an unusual case of acute kidney injury: Questions." Pediatr Nephrol 36(12): 4135-4136.

Gates, A., et al. (2021). "Effectiveness and safety of interventions to manage childhood overweight and obesity: An Overview of Cochrane systematic reviews." Paediatr Child Health 26(5): 310-316.

Background: Childhood overweight and obesity are associated with adverse physical, social, and psychological outcomes. Objectives: We conducted an overview of Cochrane systematic reviews on the effectiveness and risks of interventions to treat overweight and obesity

Gebre, M., et al. (2021). "Variable Diagnostic Performance of Stool Xpert in Pediatric Tuberculosis: A Systematic Review and Meta-analysis." Open Forum Infect Dis 8(8): ofaa627.

Background: Difficult specimen collection and low bacillary load make microbiological confirmation of tuberculosis (TB) in children challenging. In this study, we conducted a systematic review and meta-analysis to assess the diagnostic accuracy of Xpert on stool for pediatr

Geggel, R. L., et al. (2020). "Scimitar syndrome: A new multipatch technique and incidence of postoperative pulmonary vein obstruction." JTCVS Tech 4: 208-216.

Objective: A review of our center's experience before March 2011 showed that one half of 36 patients who had a baffling or reimplantation procedure to repair scimitar syndrome developed pulmonary vein obstruction. We analyzed the results of a new operation that enla

Gimpel, C., et al. (2020). "Systematic review on outcomes used in clinical research on autosomal recessive polycystic kidney disease-are patient-centered outcomes our blind spot?" Pediatr Nephrol 36(12): 3841-3851.

BACKGROUND: Autosomal recessive polycystic kidney disease (ARPKD) is a rare severe hepatorenal disease. Survivors of pulmonary hypoplasia and patients with milder presentations often achieve long-term survival but frequently require kidney and/or liver transplantatio

Gipson, T. T., et al. (2014). "Early neurodevelopmental screening in tuberous sclerosis complex: a potential window of opportunity." Pediatr Neurol 51(3): 398-402.

BACKGROUND: Infants born with tuberous sclerosis complex, a genetic condition resulting from a mutation in TSC1 or TSC2, are at increased risk for intellectual disability and/or autism. Features of epilepsy, neuropathology, genetics, as well as timing and type of mechanis

Goenaga-Vazquez, Y., et al. (2021). "Therapeutic challenges in managing pediatric psoriasis." Int J Womens Dermatol 7(3): 314-318.

Background: Each year, 20,000 patients aged <10years are diagnosed with psoriasis. Pediatric-onset psoriasis has many similarities to adult-onset disease, and previous studies suggest that the incidence might be increasing in both populations. Objective: The challenges tha

Gorito, V., et al. (2021). "Coarctation of Aorta in Turner Mosaicism." Int J Pediatr Adolesc Med 8(4): 268-270.

The prevalence of hypertension in the pediatric age range is estimated at 1-5% worldwide, with higher rates in adolescence. Although primary hypertension is more common, due to the increasing prevalence of obesity and metabolic syndrome among adolescents, seconda

Gowda, V. K., et al. (2021). "Canavan Disease: Clinical and Laboratory Profile from Southern Part of India." Ann Indian Acad Neurol 24(3): 347-350.

Background: Canavan disease (CD) is an autosomal recessively inherited leukodystrophy. It affects one in 6,400 to 13,500 people in the Jewish population. However, prevalence and presentation of the disease in India is largely unknown; hence, we are reporting this series.

Gowda, V. K., et al. (2021). "Profile of Indian Children with Childhood Ataxia and Central Nervous System Hypomyelination/Vanishing White Matter Disease: A Single Center Experience from Southern India." J Pediatr Genet 10(3): 205-212.

Background Childhood ataxia with central nervous system hypomyelination (CACH) is a recently described childhood inherited white matter disease, caused by mutations in any of the five genes encoding eukaryotic translation initiation factor (eIF2B). Methods Retrospect

Greenberg, B., et al. (2021). "Utilization and Treatment Patterns of Disease-Modifying Therapy in Pediatric Patients with Multiple Sclerosis in the United States." Int J MS Care 23(3): 101-105.

Background: The current landscape and treatment patterns of disease-modifying therapy (DMT) use in pediatric patients with multiple sclerosis (MS) are not yet well understood. This study examined DMT utilization and treatment patterns in pediatric patients newly diagn

Greenblatt, R., et al. (2021). "Quick and Clean: LCME Scientific Method Training Without a Teaching Laboratory." Med Sci Educ 31(1): 7-9.

This exercise satisfies the Liaison Committee on Medical Education Standard 7.3 for medical student training in the scientific method. The students are challenged, individually and in small groups, to state and test hypotheses based on real patient data concerning risk facto

Griffiths, E. R., et al. (2021). "Limited durability of expandable pericardial tissue valves in the mitral position in children." JTCVS Tech 5: 84-86.

Gunay, N., et al. (2020). "A rare cause of membranoproliferative patterns of injury in siblings with steroid-resistant nephrotic syndrome: Answers." Pediatr Nephrol 36(12): 4029-4032.

Gunay, N., et al. (2020). "A rare cause of membranoproliferative patterns of injury in siblings with steroid-resistant nephrotic syndrome: Questions." Pediatr Nephrol 36(12): 4027-4028.

Gunjan, et al. (2020). "Is Ultrasonography a Better Method of Endotracheal Tube Size Estimation in Pediatric Age Group than the Conventional Physical Indices-Based Formulae?" Anesth Essays Res 14(4): 561-565.

Background: Providing safe anaesthesia to paediatric patients is a challenging task. This requires a thorough knowledge of the soft and pliable paediatric airway. Owing to the vulnerability of the anatomical structures involved, choosing an appropiate sized endotracheal tu[illegible]

Gupta, A. C., et al. (2020). "Development of an age-scalable 3D computational phantom in DICOM standard for late effects studies of childhood cancer survivors." Biomed Phys Eng Express 6(6).

Purpose: We previously developed an age-scalable 3D computational phantom that has been widely used for retrospective whole-body dose reconstructions of conventional two-dimensional historic radiation therapy (RT) treatments in late effects studies of childhood can[illegible]

Guruchandrasekar, S. H., et al. (2020). "Endovascular Repair of Thoracic Aortic Pseudoaneurysms in Children." JACC Case Rep 2(12): 1895-1898.

Pediatric aortic pseudoaneurysms are rare and can result in life-threatening sequelae. We describe 2 cases of exclusion of descending thoracic aortic pseudoaneurysm by different approaches, chosen based on the anatomy and cause of the lesions. (Level of Difficulty: Begi[illegible]

Gurugubelli, K. R. and B. V. Bhat (2021). "Coronavirus Disease 2019 Infection among Children: Pathogenesis, Treatment, and Outcome." J Pediatr Intensive Care 10(3): 167-173.

Coronavirus disease 2019 (COVID-19) is a contagious disease that may lead to respiratory distress syndrome and even death. Neonates and children are most vulnerable population to COVID-19 infection; however, the infection is usually milder and has a better prognosis i[illegible]

Haakman, O., et al. (2021). "In vitro fertilization cycles stimulated with follitropin delta result in similar embryo development and quality when compared with cycles stimulated with follitropin alfa or follitropin beta." F S Rep 2(1): 30-35.

Objective: To study the impact of follitropin delta for ovarian stimulation on embryo development and quality compared with that of follitropin alfa or beta in in vitro fertilization/intracytoplasmic sperm injection (IVF/ICSI) cycles. Design: Retrospective cohort study. Setting

Halsey, J., et al. (2021). "Periorbital and Globe Injuries in Pediatric Orbital Fractures: A Retrospective Review of 116 Patients at a Level 1 Trauma Center." Craniomaxillofac Trauma Reconstr 14(3): 183-188.

Study Design: Retrospective chart review of pediatric and globe injuries associated with orbital fractures. Objective: Our study seeks to examine these injuries and their association with orbital fractures at our trauma center to gain a better understanding of how to approa[illegible]

Han, B., et al. (2020). "Use of Chimeric Antigen Receptor Modified T Cells With Extensive Leukemic Myocardial Involvement." JACC CardioOncol 2(4): 666-670.

Harper, B. D., et al. (2020). "How to ACTFAST But Think Deliberately: An Intervention to Teach Critical Thinking in the Pediatric Clerkship." J Pediatr 226: 5-8 e2.

Hawken, S., et al. (2020). "External validation of machine learning models including newborn metabolomic markers for postnatal gestational age estimation in East and South-East Asian infants." Gates Open Res 4: 164.

Background: Postnatal gestational age (GA) algorithms derived from newborn metabolic profiles have emerged as a novel method of acquiring population-level preterm birth estimates in low resource settings. To date, model development and validation have been carried

Heflin, B. H., et al. (2020). "Impact of Parenting Intervention on Observed Aggressive Behaviors in At-Risk Infants." J Child Fam Stud 29(8): 2234-2245.

Aggressive behaviors in early childhood persist through childhood and adolescence and result in negative outcomes. However, studies assessing aggressive behaviors in early childhood have focused primarily on parent report. Additionally, the effects of parenting intervent[illegible]

Heidenreich, L. S., et al. (2020). "Correction to: An infant with hyperechoic cystic kidneys and congenital diaphragmatic hernia: Answers." Pediatr Nephrol 36(12): 4147.

Heidenreich, L. S., et al. (2020). "An infant with hyperechoic cystic kidneys and congenital diaphragmatic hernia: Answers." Pediatr Nephrol 36(12): 4085-4087.

Heidenreich, L. S., et al. (2020). "An infant with hyperechoic cystic kidneys and congenital diaphragmatic hernia: Questions." Pediatr Nephrol 36(12): 4083-4084.

Heise, J. W., et al. (2021). "Laparoscopic Appendectomy: A Safe and Definitive Solution for Suspected Appendicitis." Visc Med 37(3): 180-188.

Introduction: Since conservative antibiotic treatment in uncomplicated appendicitis might not solve the clinical problem definitively, it has to compete with the results of today's laparoscopic appendectomy. Methods: In a county hospital, accommodating also a pediatric d[illegible]

Henderson, A. M., et al. (2021). "Greater Arterial Stiffness in Children with or without Second-generation Antipsychotic Treatment for Mental Health Disorders: Rigidite Arterielle Plus Importante Chez Les Enfants Avec ou Sans Traitement Par Antipsychotiques de la Deuxieme Genera[illegible]

OBJECTIVE: Second-generation antipsychotics (SGAs) are used for a variety of mental disorders and are associated with cardiometabolic side effects in children. The objective of this study was to assess the cardiovascular health of children with mental disorders that are SG[illegible]

Hesaraki, M., et al. (2021). "Knowledge, attitude, practice and clinical recommendations of health care workers towards COVID-19: a systematic review." Rev Environ Health 36(3): 345-357.

OBJECTIVES: This study aimed to evaluate the knowledge, attitude, practice, and clinical recommendations of health care workers (HCWs) towards COVID-19. METHODS: In this systematic review study, international databases (Web of Science, PubMed, and Scopus) were s[illegible]

Hobbs, R. and M. S. Si (2021). "Commentary: On aortic uncrossing: New variation on an old controversy." JTCVS Tech 5: 97-98.

Hongu, H., et al. (2020). "Subaortic aneurysm after arterial switch operation for transposition type double outlet right ventricle." JTCVS Tech 3: 290-293.

Hosic, S., et al. (2021). "Rapid Prototyping of Multilayer Microphysiological Systems." ACS Biomater Sci Eng 7(7): 2949-2963.

Microfluidic organs-on-chips aim to realize more biorelevant in vitro experiments compared to traditional two-dimensional (2D) static cell culture. Often such devices are fabricated via poly(dimethylsiloxane) (PDMS) soft lithography, which offers benefits (e.g., high feature

Hsu, K. L., et al. (2021). "3D cone-beam C.T. imaging used to determine the effect of disinfection protocols on the dimensional stability of full arch impressions." Saudi Dent J 33(7): 453-461.
Aim: This study aimed to investigate the dimensional stability of irreversible hydrocolloid and polyvinylsiloxane (P.V.S.) impressions after exposure to four commercial disinfectants using cone-beam computed tomography (CBCT). Materials and Methods: Two different impr

Huber, J. N., et al. (2020). "Learner Preference of Schedule Type Improves Engagement of Pediatric Residents: Results of a Mixed-Methods Analysis." Med Sci Educ 30(4): 1551-1559.
Objective: Determine whether a call or shift schedule is better for acquiring optimal knowledge and professionalism, while limiting fatigue for pediatric residents during the pediatric intensive care unit (PICU) rotation in a small residency program. Methods: This was a prosp

Hudzik, S. A., et al. (2020). "Sulfamethoxazole-Trimethoprim and Hyperkalemia in an Infant." J Med Cases 11(9): 283-285.
Hyperkalemia is a potentially life-threatening electrolyte abnormality in both children and adults. In the setting of elevated serum potassium concentrations, cardiac conduction disturbances and cardiac arrest may occur. In the pediatric intensive care unit (PICU) setting, th

Hwang, M., et al. (2020). "Brain Contrast-Enhanced Ultrasound Evaluation of a Pediatric Swine Model." Ultrasound Q.
Brain injury remains a leading cause of morbidity and mortality in children. We evaluated the feasibility of using a pediatric swine model to develop contrast-enhanced ultrasound (CEUS)-based measures of brain perfusion for clinical application in various types of brain inju

Illario, M., et al. (2020). "Go for it! Exercising makes you happy and strong." Transl Med UniSa 23: 92-105.
Despite it is generally recognized the beneficial role of physical activity, large portion of the population is physically inactive. Very alarmingly, the well-known gender gap in physical activity is constantly increasing. Several barriers obstacle women to perform physical activit

Imai, K., et al. (2014). "[Dietary therapy of epilepsy]." Nihon Rinsho 72(5): 875-880.
Reappraisal of ketogenic diets (KD) were delayed in Japan compared to USA and Korea. The reasons are unknown, but possible explanations are (1) Japanese food culture prefers rice and less fat and (2) ACTH therapy is preferred for West syndrome in Japan. Since Japanese

Inal, S., et al. (2020). "Meanings of the Feelings and Thoughts on Children's Hematology and Oncology Service Nurses' Experience: A Qualitative Study." Florence Nightingale J Nurs 28(3): 258-267.
AIM: This study aimed to analyze the feelings and thoughts related to working in the pediatric hematology-oncology unit of the nurses who have worked in this unit in the past, related and the meanings they attribute to it. METHOD: In this qualitative study, 15 nurses betw

Ivanov, S., et al. (2020). "Transformation of Chronic Myeloid Leukemia to Acute Biphenotypic Leukemia." J Med Cases 11(8): 239-242.
Chronic myeloid leukemia (CML) is a myeloproliferative disorder with clonal proliferation of all myeloid cell lines. The disease typically manifests in three phases: chronic course followed by an accelerated phase and finally a terminal blast crisis. A blast crisis is defined as th

Iwamura, A. P. D., et al. (2021). "Immunity and inflammatory biomarkers in COVID-19: A systematic review." Rev Med Virol 31(4): e2199.
Coronavirus disease 2019 (COVID-19) is a clinical syndrome caused by the severe acute respiratory syndrome coronavirus 2 (SARS-CoV-2) virus. Patients can be asymptomatic or present respiratory and gastrointestinal symptoms, and even multiple-organ failure which can l

Jha, P., et al. (2020). "Isolated Chylopericardium in an Infant With Hypoplastic Left Heart Syndrome." JACC Case Rep 2(13): 2115-2119.
Isolated chylopericardium is an exceedingly rare complication in any age group. Review of the limited published case series shows the most common cause of isolated chylopericardium to be cardiac surgery. We present a case of isolated chylopericardium after a bidirectio

Jin, X., et al. (2021). "The comparison of fluticasone propionate/formoterol with fluticasone propionate/salmeterol for paediatric asthma: a meta-analysis of randomized controlled trials." Postepy Dermatol Alergol 38(3): 377-383.
Introduction: The comparison of fluticasone propionate/formoterol (FP/FORM) with fluticasone propionate/salmeterol (FP/SAL) for paediatric asthma remains controversial. Aim: We conduct a systematic review and meta-analysis to explore the efficacy and safety of FP/FO

Jomaa, D., et al. (2021). "An 11-month old with failure to thrive." Paediatr Child Health 26(5): 261-263.

Ju, M., et al. (2021). "Gender Representation in Medical Emergency Training Videos. Perpetuating Bias." ATS Sch 2(2): 168-171.

Kabiri, N., et al. (2020). "Promoting parental education of infants hospitalized in a children's hospital in Tabriz, Iran: a best practice implementation project." JBI Evid Implement 19(3): 288-295.
INTRODUCTION: Parents of infants admitted to hospital have high information needs. A good educational program will improve the outcomes and communication needs of these parents. Results from some studies have shown that patient education in Iran is inappropriate

Kalangos, A., et al. (2021). "Pediatric tricuspid valve replacement with a transcatheter aortic valve (SAPIEN 3)." JTCVS Tech 6: 121-124.

Kalfa, D. (2020). "Commentary: Turkish blacksmiths were expert craftsmen at manufacturing scimitars; congenital cardiac surgeons need to be expert craftsmen at repairing them." JTCVS Tech 4: 219-220.

Kantor, P. F., et al. (2021). "Hypertrophic Cardiomyopathy in Adolescence: Application of Guidelines." JACC Case Rep 3(1): 10-15.
We present the course and management of an adolescent male with hypertrophic cardiomyopathy. The importance of family history, early screening, accurate evaluation of hypertrophy, and risk stratification for eligibility for a defibrillator in hypertrophic cardiomyopathy

Karabeg, E., et al. (2021). "Influence of tension of the nuchal cord to the developmental output in a one-year-old child." Int J Pediatr Adolesc Med 8(3): 177-180.
Objective: Influence of nuchal umbilical cord tension in a newborn on the developmental outcome at the end of the first year. Methods: and subjects of research: The research is prospective, conducted in northern Bosnia and Herzegovina (Una Sana Canton), for a period o

Karaci, M. and A. Yasar (2020). "Urinary Stone Related Urinary Tract Infection Caused by Raoultella Ornithinolytica in a Child: A Case Report." Acta Clin Croat 59(4): 751-753.
Raoultella ornithinolytica is one of the three species of Raoultella. The present case report describes a two-year-old child who had complaints of discomfort and fever, and Raoultella ornithinolytica in his urinary culture. Grade-2 hydronephrosis and a 7.5-mm urinary stone

Karamichalis, J. M. and D. N. Campbell (2021). "Commentary: Virtual fit reality and tailored approach in ventricular assist device implantation." JTCVS Tech 6: 140-141.

Karba, B. E., et al. (2021). "The Clinical Dilemma of Autism Spectrum Disorder Diagnosis in a Child with 9p Deletion." J Pediatr Genet 10(3): 250-252.

We reported on a 3-year-old girl child patient with the presence of trigonocephaly, broad nasal bridge, flattened occiput, and midface hypoplasia. Formal assessment of her development profile demonstrated expressive and receptive language delays, fine and gross motor

Karunakar, P., et al. (2020). "A child with tetany, convulsions, and nephrocalcinosis: Answers." Pediatr Nephrol 36(12): 4119-4122.

Karunakar, P., et al. (2020). "A child with tetany, convulsions, and nephrocalcinosis: Questions." Pediatr Nephrol 36(12): 4115-4117.

Kataru, C. R., et al. (2021). "Study of Prothrombotic Gene Variations Associated with the Risk of Development of Thrombosis in Patients with Down Syndrome." Indian J Hematol Blood Transfus 37(3): 507-508.

Katt, T. E., et al. (2020). "Williams Syndrome and Neonatal Cardiac Surgery for Congenital Single Ventricle." JACC Case Rep 2(11): 1716-1719.

Williams syndrome (WS) is an arteriopathic derangement associated with supravalvular aortic stenosis and branch pulmonary stenosis. We describe double-outlet right ventricle with mitral atresia and aortic arch hypoplasia in an infant with WS. This case demonstrates the

Kavarana, M. N. (2020). "Commentary: What switch, to switch or not to switch: That is the question!" JTCVS Tech 4: 227-228.

Kavarana, M. N. (2020). "Commentary: When you hear hoofbeats think horses-but don't forget the zebras!" JTCVS Tech 3: 299.

Kaya, A. and A. Isler-Dalgic (2020). "Evaluating the Job Satisfaction of Nurses Working in Pediatric Clinics in Terms of Eleven Dimensions and the Influencing Factors." Florence Nightingale J Nurs 28(3): 312-320.

AIM: This study aimed to evaluate the job satisfaction of nurses working in pediatric clinics in terms of 11 dimensions and the influencing factors. METHOD: The sample of this descriptive study was composed of 92 nurses who were responsible for patient care in two public

Kenney, D. and E. Wirrell (2014). "Patient considerations in the management of focal seizures in children and adolescents." Adolesc Health Med Ther 5: 49-65.

Focal epilepsy accounts for approximately one-half to two-thirds of new-onset epilepsy in children. Etiologies are diverse, and range from benign epilepsy syndromes with normal neuroimaging and almost certain remission to focal malformations of cortical development or

Khabbush, A., et al. (2017). "Neuronal decanoic acid oxidation is markedly lower than that of octanoic acid: A mechanistic insight into the medium-chain triglyceride ketogenic diet." Epilepsia 58(8): 1423-1429.

OBJECTIVE: The medium-chain triglyceride (MCT) ketogenic diet contains both octanoic (C8) and decanoic (C10) acids. The diet is an effective treatment for pharmacoresistant epilepsy. Although the exact mechanism for its efficacy is not known, it is emerging that C10, but

Khalil, A. F., et al. (2021). "Does faecal calprotectin differentiate between inflammatory bowel disease colitis and non-inflammatory bowel disease colitides?" Prz Gastroenterol 16(3): 219-223.

Introduction: Chronic colitis is a major problem worldwide with high morbidity. Causes of chronic colitis are heterogeneous. A cut-off level of faecal calprotectin to predict inflammatory bowel disease (IBD) as a cause of chronic colitis is lacking. Aim: To study the level of fae

Khasawneh, W., et al. (2021). "Umbilical catheter rupture: A serious complication in neonatal intensive care units." Int J Pediatr Adolesc Med 8(3): 146-148.

Umbilical catheterization is commonly used as a route to provide medications and fluids to the neonates as well as for blood sampling and continuous monitoring. Although the rupture of umbilical catheters is considered as a rare, preventable complication, it has been rep

Khramova, R. N., et al. (2021). "Relationship of Nutritional Status and Spirometric Parameters in Children with Bronchial Asthma." Sovrem Tekhnologii Med 12(3): 12-23.

The potential mechanisms of bronchial asthma (BA) negative modification under the influence of obesity are currently being actively studied. However, at present, the effect of nutritional status on bronchial obstruction in children with BA cannot be considered established

Kim, H. S., et al. (2020). "Acute bilateral vision loss in a toddler with stage 5 chronic kidney disease: Answers." Pediatr Nephrol 36(12): 4125-4127.

Kim, H. S., et al. (2020). "Acute bilateral vision loss in a toddler with stage 5 chronic kidney disease: Questions." Pediatr Nephrol 36(12): 4123-4124.

Kirschen, M. P., et al. (2020). "The association between early impairment in cerebral autoregulation and outcome in a pediatric swine model of cardiac arrest." Resusc Plus 4: 100051.

Aims: Evaluate cerebral autoregulation (CAR) by intracranial pressure reactivity index (PRx) and cerebral blood flow reactivity index (CBFx) during the first four hours following return of spontaneous circulation (ROSC) in a porcine model of pediatric cardiac arrest. Determin

Klambt, V., et al. (2020). "Correction to: Different approaches to long-term treatment of aHUS due to MCP mutations: a multicenter analysis." Pediatr Nephrol 36(12): 4015.

Klaver-Flores, S., et al. (2020). "Genomic Engineering in Human Hematopoietic Stem Cells: Hype or Hope?" Front Genome Ed 2: 615619.

Many gene editing techniques are developed and tested, yet, most of these are optimized for transformed cell lines, which differ from their primary cell counterparts in terms of transfectability, cell death propensity, differentiation capability, and chromatin accessibility to

Korneva, K. G., et al. (2021). "Diagnostic Capabilities of Islet Autoantibodies in Children with New-Onset Type 1 Diabetes Mellitus and Healthy Siblings." Sovrem Tekhnologii Med 12(6): 29-34.

The aim of the study is to determine the diagnostic utility of several islet autoantibodies and their combinations in order to identify individuals susceptible to type 1 diabetes mellitus (T1DM) among healthy siblings in the pediatric population within the scope of the develop

Kossoff, E. H. and H. S. Wang (2013). "Dietary therapies for epilepsy." Biomed J 36(1): 2-8.

Since their introduction in 1921, high-fat, low-carbohydrate "ketogenic" diets have been used worldwide for refractory childhood epilepsy. Approximately half of the children have at least half their seizures reduced, including 15% who are seizure free. The mechanisms of a

Kotcherlakota, S., et al. (2020). "Augmented Reality Technology as a Teaching Strategy for Learning Pediatric Asthma Management: Mixed Methods Study." JMIR Nurs 3(1): e23963.

BACKGROUND: Asthma is a major chronic disease affecting 8.6% of children in the United States. OBJECTIVE: The purpose of this research was to assess the use of clinical simulation scenarios using augmented reality technology to evaluate learning outcomes for nurse prac

Kousta, E., et al. (2020). "Paraganglioma syndrome type 4 presenting as hypertensive encephalopathy in an 8-year-old boy." Hippokratia 24(3): 143.

Krasilnikova, S. V., et al. (2021). "Periostin as a Biomarker of Allergic Inflammation in Atopic Bronchial Asthma and Allergic Rhinitis (a Pilot Study)." Sovrem Tekhnologii Med 12(5): 37-45.
The involvement of periostin in Th2-dependent allergic inflammation has been documented. However, the significance of periostin as a biomarker of local allergic inflammation in the nasal mucosa (NM) of patients with atopic bronchial asthma (BA) and allergic rhinitis (AR)

Kumar, A., et al. (2021). "Initial experience with use of infrared assistance for intravenous injection of radiopharmaceuticals." World J Nucl Med 20(2): 172-175.
Radiopharmaceutical injection is challenging as it poses radiation exposure to staff as well as patient. Infrared light-assisted devices have been available since many years and have garnered mixed reviews in the pediatric age group. However, there are no data on outcome

Kumar, T. K. S., et al. (2020). "Modified Warden operation using aortic homograft." JTCVS Tech 4: 271-273.

Kumari, R. K., et al. (2020). "Sleep quality assessment among college students using Pittsburgh Sleep Quality Index in a municipal corporation area of Uttarakhand, India." Ceylon Med J 65(4): 86-94.
Introduction: Poor sleep quality have been reported among adults in most countries and are increasingly been reported in their predecessors, the college students. The present study aimed to assess sleep patterns and determine its association with various correlates amor

Lara-Mendoza, L. (2020). "50 Years Ago in TheJournalofPediatrics: The Newborn Skin." J Pediatr 226: 265.

Lavery, M. J., et al. (2021). "Cutaneous manifestations of COVID-19 in children (and adults): A virus that does not discriminate." Clin Dermatol 39(2): 323-328.
Coronavirus disease 2019 (COVID-19) is caused by the severe acute respiratory syndrome coronavirus 2 (SARS-CoV-2), a beta coronavirus with a characteristic S-glycoprotein spike on the cell surface. Initial reports did not include cutaneous manifestations as a feature of CC

Leao, H. M. and A. P. Caldeira (2021). "Accessibility and care pathways for children admitted to hospital for ambulatory care sensitive conditions." Cien Saude Colet 26(8): 3301-3310.
The aim of this study was to analyze health service accessibility and the care pathways of children admitted to hospital for ambulatory care sensitive conditions (ACSCs). A cross-sectional study was conducted of a random sample of children hospitalized over a period of one

Lee, M. E., et al. (2020). "A rare pediatric case of left ventricular outflow tract infective endocarditis in hypertrophic cardiomyopathy." JTCVS Tech 4: 281-282.

Lee, S. Y., et al. (2020). "Perioperative Temperature Management in Children: What Matters?" Pediatr Qual Saf 5(5): e350.
Inadvertent perioperative hypothermia is common and associated with increased risk of perioperative complications. Adult data drives most guidelines for pediatric perioperative temperature management and does not consistently demonstrate effectiveness in children. T

Leerink, J. M., et al. (2020). "Cardiac Disease in Childhood Cancer Survivors: Risk Prediction, Prevention, and Surveillance: JACC CardioOncology State-of-the-Art Review." JACC CardioOncol 2(3): 363-378.
Cardiac diseases in the growing population of childhood cancer survivors are of major concern. Cardiotoxicity as a consequence of anthracyclines and chest radiotherapy continues to be relevant in the modern treatment era. Mitoxantrone has emerged as an important tre

Lenhard, F., et al. (2020). "Long-term outcomes of therapist-guided Internet-delivered cognitive behavior therapy for pediatric obsessive-compulsive disorder." NPJ Digit Med 3(1): 124.
Cognitive behavior therapy (CBT) is the recommended first-line intervention for children and adolescents with obsessive-compulsive disorder (OCD), but is not broadly accessible. Internet-delivered CBT (ICBT) with minimal therapist support is efficacious and cost-effective,

Leung, K. C. P. and T. C. S. Ko (2021). "Childhood cone-rod dystrophy with macular cyst formation in ABCA4 mutation identified by serial spectral-domain optical coherence tomography." Taiwan J Ophthalmol 11(3): 312-316.
Cone-rod dystrophy (CORD) is a type of progressive hereditary retinal dystrophies that causes cone predominant photoreceptor degeneration characterized by wide genotypic and phenotypic heterogeneity. Macular cyst (MC) occurs very infrequently in the pediatric age gr

Leventoglu, E., et al. (2020). "Pseudo-Bartter syndrome and staghorn calculi in an infant with chronic diarrhea: Answers." Pediatr Nephrol 36(12): 4099-4101.

Leventoglu, E., et al. (2020). "Pseudo-Bartter syndrome and staghorn calculi in an infant with chronic diarrhea: Questions." Pediatr Nephrol 36(12): 4097-4098.

Leventoglu, E., et al. (2020). "Severe kidney dysfunction in a child who presented with constipation: Answers." Pediatr Nephrol 36(12): 4079-4082.

Leventoglu, E., et al. (2020). "Severe kidney dysfunction in a child who presented with constipation: Questions." Pediatr Nephrol 36(12): 4077-4078.

Leventoglu, E., et al. (2020). "A rare cause and a rare complication of hypertension in an adolescent: Answers." Pediatr Nephrol 36(12): 4105-4108.

Leventoglu, E., et al. (2020). "A rare cause and a rare complication of hypertension in an adolescent: Questions." Pediatr Nephrol 36(12): 4103-4104.

Leventoglu, E., et al. (2020). "Macroscopic hematuria in a child with portal hypertension: Answers." Pediatr Nephrol 36(12): 4037-4040.

Leventoglu, E., et al. (2020). "Macroscopic hematuria in a child with portal hypertension: Questions." Pediatr Nephrol 36(12): 4033-4035.

Levy, K., et al. (2020). "The Photographs of Meaning Program for Pediatric Palliative Caregivers and Its Impact on Meaning, Well-Being, and Perceived Social Support." Palliat Med Rep 1(1): 84-91.
Background: Caring for a child or adolescent with palliative care needs can significantly influence the physical, mental, financial, and social well-being of caregivers. Due to this multifaceted impact, there is a demand for evidence-based support that meets the distinct needs

Li, X. and H. Wang (2020). "A 'normotensive' adult with dysplastic double aortic arch." Eur Heart J.

Li, X., et al. (2020). "Mitochondrial dysfunction in fibrotic diseases." Cell Death Discov 6(1): 80.

Although fibrosis is a common pathological feature of most end-stage organ diseases, its pathogenesis remains unclear. There is growing evidence that mitochondrial dysfunction contributes to the development and progression of fibrosis. The heart, liver, kidney and lung a

Ligon, R. A., et al. (2020). "Rerouting of Cerebral Circulation: Extensive Transcatheter Aortic Arch Revision." JACC Case Rep 2(6): 855-859.
We describe an adolescent with long-standing atresia of the head/neck arteries and severe aortic coarctation. Because of progressive symptoms, a series of interventions was undertaken to provide direct aorta-to-carotid artery flow and coarctation treatment. This case hig

Lindefeldt, M., et al. (2019). "The ketogenic diet influences taxonomic and functional composition of the gut microbiota in children with severe epilepsy." NPJ Biofilms Microbiomes 5(1): 5.
The gut microbiota has been linked to various neurological disorders via the gut-brain axis. Diet influences the composition of the gut microbiota. The ketogenic diet (KD) is a high-fat, adequate-protein, low-carbohydrate diet established for treatment of therapy-resistant e

Lirette, M. P., et al. (2021). "A 4-week-old infant with wheezing and abnormal movements." Paediatr Child Health 26(6): 327-329.

Liu, C., et al. (2020). "Severe maternal morbidity among migrants with insecure residency status in Sweden 2000-2014: a population-based cohort study." J Migr Health 1-2: 100006.
Background: Migrants with insecure residency status (i.e., undocumented migrants and asylum-seekers, who are denied or waiting for authorized residency) often experience social and psychosocial adversities and limited access to health care. Nonetheless, they have not l

Lopez-Alvarez, J. M., et al. (2021). "Evaluation of Training in Pediatric Ultrasound-guided Vascular Cannulation Using a Model." J Med Ultrasound 29(3): 171-175.
Background: The study objective was to evaluate a training program and a training model for pediatric ultrasound-guided vascular cannulation (USGVC) by inexperienced operators. Methods: An observational descriptive study was conducted at the pediatric intensive care

Lovin, B. D., et al. (2021). "Outcomes in Pediatric Transsphenoidal Pituitary Surgery Stratified by the Use of Image Guidance: An Analysis of the Kids' Inpatient Database from 1997 to 2016." J Neurol Surg B Skull Base 82(Suppl 3): e45-e50.
Background To evaluate the utilization of image guidance technology for pediatric transsphenoidal pituitary resection (TSPR) and analyze the complication rates, length of stay (LOS), and total cost for such surgeries as function of time and utilization of image guidance. Met

Lyashenko, C., et al. (2020). "Adjunctive dental therapies in caries-active children: Shifting the cariogenic salivary microbiome from dysbiosis towards non-cariogenic health." Hum Microb J 18.
Background: The oral microbiome is a complex assembly of microbial species, whose constituents can tilt the balance towards progression of oral disease or sustained health. Recently we identified sex-specific differences in the salivary microbiome contained within caries-

Lyons, V. H., et al. (2021). "Firearms and protective orders in intimate partner homicides." J Fam Violence 36: 587-596.
Purpose: To determine differences among intimate partner homicides (IPH) by whether or not a firearm was used in and whether a protective order (PO) was filed prior to IPH. Method: We identified all incidents of IPH recorded in the National Violent Death Reporting Syst

Ma, R., et al. (2021). "Global and Simultaneous Hypothesis Testing for High-Dimensional Logistic Regression Models." J Am Stat Assoc 116(534): 984-998.
High-dimensional logistic regression is widely used in analyzing data with binary outcomes. In this paper, global testing and large-scale multiple testing for the regression coefficients are considered in both single- and two-regression settings. A test statistic for testing the gl

Madden, C., et al. (2020). "Team Science Process Builds Research Coordinators' Voices in a National Pediatric Clinical Trials Network." SOCRA Source 2020(105): 68-73.
Geographically-dispersed teams have become the norm in clinical research collaborations. The Institutional Development Awards (IDeA) Program, first authorized by Congress in 1993 and managed by the National Institute of General Medical Sciences, has been developed

Maeda, K. and K. R. Ryan (2020). "Commentary: A new option for patch material on coronary artery ostium plasty." JTCVS Tech 4: 235-236.

Magar, S. T. and B. R. Gyawali (2020). "Otorhinolaryngological and Ophthalmological Manifestations of COVID-19 in the Pediatric Population." JNMA J Nepal Med Assoc 58(232): 1093-1096.
Severe Acute Respiratory Syndrome Coronavirus 2 pandemic has affected several countries throughout the world. Being very contagious, it can affect any individual. So far, the prevalence of Severe Acute Respiratory Syndrome Coronavirus 2 in children amongst the total in

Maharjan, A., et al. (2020). "Congenital Tracheoesophageal Fistula in Very Low Birth Weight Preterm Neonate with an Oligohydramnios as a Rare Presentation: A Case Report." JNMA J Nepal Med Assoc 58(232): 1075-1079.
Tracheoesophageal fistula is a challenging anomaly with a rare prevalence with symptoms mainly respiratory, sometimes digestive. We present a rare case of oesophageal atresia with distal Tracheoesophageal fistula in a female child whose mother presented with severe o

Mahmoud, A., et al. (2020). "Prevalence of Helicobacter pylori infection among children with primary nephrotic syndrome: a cross-sectional study." Afr Health Sci 20(4): 1624-1631.
Background: Limited data are available about the prevalence of helicobacter pylori (H.pylori) infection among primary NS children. Objectives: To assess the frequency and risk factors of H.pylori infection among children with primary NS. Methods: A cross-sectional study w

Malaika, R., et al. (2020). "Pediatric spectacle prescription: Understanding practice patterns among ophthalmologists and optometrists in Saudi Arabia." Saudi J Ophthalmol 34(4): 278-283.
PURPOSE: This study aims to understand the approach to prescribing spectacles for children and the interpretation of refractive errors among ophthalmologists/optometrists in Saudi Arabia. METHODS: A cross-sectional survey was conducted between September and Nove

Mandal, D., et al. (2020). "Posterior Reversible Encephalopathy Syndrome in a Patient with Post Streptococcal Glomerulonephritis: A Case Report." JNMA J Nepal Med Assoc 58(232): 1072-1074.
Posterior reversible encephalopathy syndrome is a clinical-radiological syndrome neurological disorder with varied symptoms which include headache, visual field defects, seizures, altered consciousness. It is a rare complication of post-streptococcal glomerulonephritis and

Manji, I., et al. (2021). "Correction of neonatal auricular deformities with DuoDERM: A simple technique." Paediatr Child Health 26(5): 270-273.
Ear moulding in neonates has been shown to successfully correct congenital auricular anomalies. There are several available moulding techniques. However, commercially available moulding devices (e.g., EarWell and Ear Buddy) can be costly, and their alternatives have lin

Mansfield, S. A., et al. (2020). "Validating an opioid prescribing algorithm in post-operative pediatric surgical oncology patients." J Pediatr Surg.
PURPOSE: We developed an algorithm to decrease opioid prescriptions for pediatric oncology patients at discharge following surgery, based on a retrospective analysis to decrease variability and over-prescribing. The aim of this study was to prospectively test the algorithm

Maroon, J., et al. (2013). "Restricted calorie ketogenic diet for the treatment of glioblastoma multiforme." J Child Neurol 28(8): 1002-1008.
Glioblastoma multiforme is the most common malignant primary brain tumor in adults and generally considered to be universally fatal. Glioblastoma multiforme accounts for 12% to 15% of all intracranial neoplasms and affects 2 to 3 adults per every 100,000 in the United

Marrache, M., et al. (2020). "Scoliosis Research Society Annual Meeting 2019 Abstracts." J Bone Joint Surg Am 102(16): e96.
The Scoliosis Research Society (SRS) aims to foster optimal care of pediatric and adult patients with spinal deformity of all etiologies. Founded 53 years ago, the SRS has maintained a strong commitment to research and education. At the 2019 SRS Annual Meeting in Montr

Martin-McGill, K. J., et al. (2018). "The role of ketogenic diets in the therapeutic management of adult and paediatric gliomas: a systematic review." CNS Oncol 7(2): CNS17.
AIM: We performed a systematic review of the evidence for effectiveness and acceptability of different ketogenic diets (KDs) in the therapeutic management of gliomas. METHODS: The search strategy included searches of seven electronic databases. Data extraction and qu

Massey, R. J., et al. (2020). "Left Ventricular Systolic Function in Long-Term Survivors of Allogeneic Hematopoietic Stem Cell Transplantation." JACC CardioOncol 2(3): 460-471.
Background: Allogeneic hematopoietic stem cell transplantation (allo-HSCT), a potentially curative therapy for malignant and nonmalignant diseases, is being increasingly used in younger patients. Although allo-HSCT survivors have an established increased risk of cardiovas

Mathew, J. L. (2020). "Innovations to automate manual ventilation during Covid-19 pandemic and beyond." Natl Med J India 33(6): 366-371.
Manual ventilation by compressing self-inflating bags is a life-saving option for respiratory support in many resource-limited settings. Previous efforts to automate manual ventilation using mechatronic systems were unsuccessful. The Covid-19 pandemic stimulated re-expl

Mautone, J. A., et al. (2020). "Exploring Predictors of Treatment Engagement in Urban Integrated Primary Care." Clin Pract Pediatr Psychol 8(3): 228-240.
Objective: Integrated primary care (IPC) is intended to address the gap in access to behavioral health care. This may be particularly true in urban settings; however, there is a paucity of research on treatment engagement in urban IPC. This study explored factors associated

Maya, M., et al. (2021). "Growing Spectrum of Episodic Apnea with Hypotonia in a Young Infant." Ann Indian Acad Neurol 24(3): 458-459.

McDonald, C. R., et al. (2020). "Pregnant Women in Low- and Middle-Income Countries Require a Special Focus During the COVID-19 Pandemic." Front Glob Womens Health 1: 564560.

McElfish, P. A., et al. (2020). "Leveraging community engagement capacity to address COVID-19 disparities among Pacific Islander and Latinx Communities in Arkansas." J Clin Transl Sci 5(1): e81.
Northwest Arkansas, particularly Benton and Washington counties, is one of the highest COVID-19 hot spots in the United States (US), with more than half of all reported cases in this area identifying as Latinx or Pacific Islander, even though these communities account for l

McNeely, M. M., et al. (2021). "Two-Stage Dual-Nerve Facial Reanimation: Outcomes and Complications in a Series of Pediatric Patients." Plast Surg (Oakv) 29(4): 280-286.
Background: In facial reanimation via microneurovascular muscle transfer, dual-nerve reinnervation of the muscle capitalizes on the synergistic effects of spontaneous motion from cross facial nerve grafting (CFNG) and increased excursion from masseteric nerve transfer. T

Mehta, A., et al. (2021). "Improv: Transforming Physicians and Medicine." Med Sci Educ 31(1): 263-266.

Meis, C. M., et al. (2021). "Self-Assembled, Dilution-Responsive Hydrogels for Enhanced Thermal Stability of Insulin Biopharmaceuticals." ACS Biomater Sci Eng 7(9): 4221-4229.
Biotherapeutics currently dominate the landscape of new drugs because of their exceptional potency and selectivity. Yet, the intricate molecular structures that give rise to these beneficial qualities also render them unstable in formulation. Hydrogels have shown potential

Menon, J., et al. (2021). "Late-Onset Peripheral T-Cell Lymphoma Not Otherwise Specified in a Liver Transplant Recipient: A Rare Subtype of Posttransplant Lymphoproliferative Disorder." J Clin Exp Hepatol 11(4): 511-514.
Introduction: Posttransplant lymphoproliferative disorder (PTLD) is a rare complication seen in the period after liver transplant. The commonest subtype is B-cell PTLD which is usually associated with Epstein-Barr virus (EBV) infection. T-cell PTLD is rare and the association v

Merdler-Rabinowicz, R., et al. (2020). "Kidney and urinary tract findings among patients with Kabuki (make-up) syndrome." Pediatr Nephrol 36(12): 4009-4012.
BACKGROUND: Kabuki syndrome (KS) is a genetic disorder caused mainly by de novo pathogenic variants in KMT2D or KDM6A, characterized by recognizable facial features, intellectual disability, and multi-systemic involvement, including short stature, microcephaly, hearii

Mishra, A., et al. (2020). "Transposition of great arteries with total anomalous pulmonary venous connection: A modified Senning procedure for late presentation." JTCVS Tech 4: 223-226.

Mizrahi, M., et al. (2020). "Unusual presentation of an obstructing cardiac myxoma." JTCVS Tech 3: 234-235.

Morgun, A. V., et al. (2021). "Molecular Mechanisms of Proteins - Targets for SARS-CoV-2 (Review)." Sovrem Tekhnologii Med 12(6): 98-108.
The rapidly accumulating information about the new coronavirus infection and the ambiguous results obtained by various authors necessitate further research aiming at prevention and treatment of this disease. At the moment, there is convincing evidence that the pathog

Mousa, A., et al. (2021). "Atypical Teratoid Rhabdoid Tumors (ATRT): King Faisal Specialist Hospital and Research Centre experience." Int J Pediatr Adolesc Med 8(3): 154-159.
Background: Atypical teratoid rhabdoid tumor is an uncommon aggressive central nervous system tumor. All retrospective series have shown a short mean overall survival rate. Considering the rarity of the disease, few prospective clinical trials addressed treatment recomr

Muniraman, H., et al. (2020). "Parental perceptions of the impact of neonatal unit visitation policies during COVID-19 pandemic." BMJ Paediatr Open 4(1): e000899.
Objectives: To ascertain parental perceptions of the impact of restricted visiting policies to neonatal intensive care units during the current COVID-19 pandemic. Design: Cross-sectional survey of parents impacted by visitation policies. Setting: Six tertiary level neonatal unit

Murphy, T. W., et al. (2020). "Cardiac arrest: An interdisciplinary scoping review of the literature from 2019." Resusc Plus 4: 100037.
Objectives: The Interdisciplinary Cardiac Arrest Research Review (ICARE) group was formed in 2018 to conduct a systematic annual search of peer-reviewed literature relevant to cardiac arrest. Now in its second year, the goals of the review are to illustrate best practices in

Naka, F., et al. (2021). "A dermatologic perspective on multisystem inflammatory syndrome in children." Clin Dermatol 39(2): 337-343.
As of May 2020, an emerging immune-mediated syndrome primarily affecting children has been detected primarily in Europe and the United States. The incidence of this syndrome appears to mirror the initial infectious assault with a delay of several weeks. This syndrome

Nakarmi, K. K. and B. D. Pathak (2020). "Prevalence of Acute Pediatric Burns in a Tertiary Care Hospital." JNMA J Nepal Med Assoc 58(231): 862-865.
INTRODUCTION: Burn injury is an important cause of mortality and morbidity in children worldwide. Mortality is higher in developing countries than in developed ones. Most of them occur in predictable domestic settings and can be prevented. The objective of this study w

Namuli, J. D., et al. (2020). "Complementary Alternative Medicine (CAM) Use and Associated Factors among HIV Infected Children and Adolescents Seeking Mental Health Services in Uganda." EC Psychol Psychiatr 9(10): 1-8.
Background: Given the limited integration of mental health services into pediatric HIV care in sub-Saharan Africa, there is limited information on the nature of mental health service use sought by caregivers of children and adolescents living with HIV/AIDS. Methods: We an

Namuli, J. D., et al. (2020). "Knowledge Gaps about Autism Spectrum Disorders and its Clinical Management among Child and Adolescent Health Care Workers in Uganda: A Cross-Sectional Study." EC Psychol Psychiatr 9(9): 112-121.
Background: There is limited literature on the knowledge about Autism Spectrum Disorder (ASD) among child and adolescent health professionals from resource limited settings. Methods: 40 child and adolescent health professionals were purposively sampled from the tw

Nateghian, A. R., et al. (2021). "A decade of trends in the distribution and antimicrobial susceptibility of prevalent uropathogens among pediatric patients from Tehran, Iran during 2005-2016." Asian J Urol 8(3): 253-259.
Objectives: To determine changes in the distribution of uropathogens and their antimicrobial resistance in pediatric patients in a children's hospital from 2005 to 2016. Methods: A cross-sectional analysis of uropathogens and their antimicrobial resistance within inpatient

Navanandan, N., et al. (2020). "Seventy-two-hour Return Initiative: Improving Emergency Department Discharge to Decrease Returns." Pediatr Qual Saf 5(5): e342.
Unscheduled return visits within 72 hours of discharge account for 4% of pediatric emergency department (ED) visits each year and are a quality indicator of ED care. This project aimed to reduce the unexpected 72-hour return visit rate for a network of ED and urgent care

Nellan, A. and T. J. Fry (2020). "Optimizing CARs for ocular delivery." Nat Cancer 1(10): 939-940.
Effective methods for treating retinoblastoma while preserving vision are an unmet clinical need. Subretinal delivery of a hydrogel containing T cells that secrete the cytokine IL-15 and express a chimeric antigen receptor directed at the ganglioside protein GD2 completely

Nellis, J. R., et al. (2021). "Commentary: Minimally invasive pulmonary valve replacement and the adoption curve for novel techniques." JTCVS Tech 6: 130-131.

Neuhauser, C. A., et al. (2021). "Successful Management of an Infant with Atypical Presentation of Alveolar Capillary Dysplasia with Misalignment of the Pulmonary Veins." J Pediatr Intensive Care 10(3): 228-231.
A newborn infant patient presented with persistent pulmonary hypertension. For right ventricular decompression, the ductus arteriosus was kept open by prostaglandin E 1 infusion and was stented at the age of 4 weeks during heart catheterization. The child was weaned

Niedziela, M., et al. (2021). "Intrathyroidal Thymus (Incidentaloma) Mimicking Thyroid Neoplasia in DICER1 Syndrome." Eur Thyroid J 10(3): 257-261.
Introduction: With the use of ultrasonography for the evaluation of thyroid and nonthyroid neck diseases, the incidental discovery of previously unsuspected thyroid nodules/nonpalpable lesions has increased. Intrathyroidal thymus arises due to aberrant thymic migration

Nunes, G. A., et al. (2021). "Osteonecrosis of the Intermediate Cuneiform: A Case Report." Rev Bras Ortop (Sao Paulo) 56(3): 394-398.
Osteonecrosis is a disease that rarely affects the bones of the foot. When present, it is more common in the talus and in the navicular. Cases of osteonecrosis of the intermediate cuneiform are extremely rare, and after a thorough bibliographic review, we found only five re

Okamura, M., et al. (2021). "Incidence of Unintentional Intraoperative Hypothermia in Pediatric Scoliosis Surgery and Associated Preoperative Risk Factors." Spine Surg Relat Res 5(3): 154-159.
Introduction: Intraoperative hypothermia is associated with perioperative complications such as blood loss and wound infection. Thus, perioperative heat retention methods to prevent perioperative hypothermia such as providing a warmed blanket and active patients' wa

Okland, T. S., et al. (2020). "Prospective Evaluation of the Safety and Efficacy of THRIVE for Children Undergoing Airway Evaluation." Pediatr Qual Saf 5(5): e348.
Transnasal Humidified Rapid-Insufflation Ventilatory Exchange (THRIVE) is a humidified high-flow nasal cannula capable of extending apneic time. Although THRIVE is assumed to stent upper airway soft tissues, this has not been objectively evaluated. Also, there are no pric

Oleck, N. C., et al. (2021). "Pediatric Replantation after Traumatic Amputation at the Distal Forearm: Rehabilitation Protocol and Outcomes." J Hand Microsurg 13(3): 169-172.
Major limb replantation is a formidable task, especially in the pediatric setting. While meticulous microsurgical technique is required in the operating room, the authors aim to highlight the importance of postoperative rehabilitation therapy for optimal function. We highlig

Omrani, A., et al. (2021). "Evaluation of myocardial perfusion and function in patients with asymptomatic beta-thalassemia major using myocardial gated single-photon-emission computed tomography." World J Nucl Med 20(2): 145-149.
This study was conducted to evaluate the cardiac perfusion and function of patients with beta-thalassemia major (TM) using(99m)Tc-MIBI cardiac gated single-photon-emission computed tomography (SPECT) and to compare the obtained indices with echocardiographic an

Oriby, M. E. and A. Elrashidy (2021). "Comparative Effects of Total Intravenous Anesthesia with Propofol and Remifentanil Versus Inhalational Sevoflurane with Dexmedetomidine on Emergence Delirium in Children Undergoing Strabismus Surgery." Anesth Pain Med 11(1): e109048.
Background: Emergence delirium (ED) is common after strabismus surgery due to postoperative visual disturbance, vomiting, and pain. Total intravenous anesthesia (TIVA) has many advantages like smooth emergence from anesthesia, decreased incidence of postoperative

Osorio, M. F. and R. G. Vaca (2021). "Coronavirus Disease 2019 in Children: Is It Really Mild?" Infect Dis Clin Pract (Baltim Md) 29(2): e78-e81.
Coronavirus disease 2019 pandemic has spread rapidly to the world. The disease can vary from mild cases to severe respiratory distress; this may increase rapidly and overwhelm the pediatric intensive care units. Lately, there have been various reports about a de novo mu

Overbey, D. M. and A. J. Lodge (2020). "Commentary: Getting in the groove with salvage extracorporeal membrane oxygenation and left ventricular venting." JTCVS Tech 3: 218-219.

Ozdemir, R., et al. (2016). "The Impact of 3:1 Ketogenic Diet on Cardiac Repolarization Changes in Children with Refractory Seizures: A Prospective Follow-Up Study." Neuropediatrics 47(3): 157-161.
Background The association between ketogenic diet (KD) and prolonged QT interval, life-threatening ventricular arrhythmias, and sudden death is controversial. Aim We aimed to prospectively evaluate the effect of KD on electrocardiography (ECG) measures in children wi

Pagano, E., et al. (2020). "Alterations in Metabolites Associated with Hypoxemia in Neonates and Infants with Congenital Heart Disease." Congenit Heart Dis 15(4): 251-265.
Objectives: (1) To measure the global shift in the metabolome in hypoxemic versus non-hypoxemic infants with congenital heart disease; (2) To identify metabolites and metabolic pathways that are altered in hypoxemia. Study Design: Analysis of serum samples obtained p

Panda, P. K., et al. (2021). "Leigh Syndrome and SURF1 Gene Presenting with Febrile Seizure." Ann Indian Acad Neurol 24(2): 251-252.

Papes, D. (2021). "A Review of Arterial Grafts Used for Microvascular Arterial Reconstruction." J Hand Microsurg 13(3): 181-184.

Arterial grafts are sometimes used in microvascular reconstruction and their clinical benefit over standard venous grafts is unknown. To determine arterial graft utilization in clinical microvascular arterial reconstruction, a review of the literature was done. PubMed search r

Pasca, L., et al. (2016). "The changing face of dietary therapy for epilepsy." Eur J Pediatr 175(10): 1267-1276.

UNLABELLED: Ketogenic diet is an established and effective non-pharmacologic treatment for drug-resistant epilepsy. Ketogenic diet represents the treatment of choice for GLUT-1 deficiency syndrome and pyruvate dehydrogenase complex deficiency. Infantile spasms, Dra

Patel, B. K., et al. (2020). "Cowpea Mosaic Virus (CPMV)-Based Cancer Testis Antigen NY-ESO-1 Vaccine Elicits an Antigen-Specific Cytotoxic T Cell Response." ACS Appl Bio Mater 3(7): 4179-4187.

Cancer vaccines are promising adjuvant immunotherapies that can stimulate the immune system to recognize tumor-associated antigens and eliminate the residual or recurring disease. The aberrant and restricted expression of highly immunogenic cancer testis antigen NY

Patel, M., et al. (2021). "Trajectories of oral glucose tolerance testing in cystic fibrosis." Pediatr Pulmonol 56(5): 901-909.

INTRODUCTION: Annual oral glucose tolerance testing (OGTT) is the recommended screening modality for cystic fibrosis-related diabetes (CFRD) in patients with cystic fibrosis (CF). This study aimed to determine if there were patterns of progression of worsening glucose ho

Patneaude, A. and J. Kett (2020). "Cultural Responsiveness and Palliative Care during the COVID-19 Pandemic." Palliat Med Rep 1(1): 171-173.

During the COVID-19 pandemic, much has changed in the delivery of palliative care (PC). However, cultural responsiveness remains critical to our mission. It is essential to our aims of identifying individual goals of care and providing relief of suffering for our patients. Cultur

Paul, P., et al. (2020). "Quantifying long-term health and economic outcomes for survivors of group B Streptococcus invasive disease in infancy: protocol of a multi-country study in Argentina, India, Kenya, Mozambique and South Africa." Gates Open Res 4: 138.

Sepsis and meningitis due to invasive group B Streptococcus (iGBS) disease during early infancy is a leading cause of child mortality. Recent systematic estimates of the worldwide burden of GBS suggested that there are 319,000 cases of infant iGBS disease each year, and a

Penslar, J., et al. (2021). "Nonauscultatory clinical criteria are sensitive for cardiac pathology in low-risk paediatric heart murmurs." Paediatr Child Health 26(5): 294-298.

Background: Healthy children with likely innocent heart murmurs are frequently referred to cardiologists for reassurance. Existing guidelines that advise against these referrals are not consistently followed partly because they involve subjective auscultatory judgements wit

Perez, A., et al. (2019). "Comment on: Ketogenic diet treatment in recurrent diffuse intrinsic pontine glioma in children: A safety and feasibility study." Pediatr Blood Cancer 66(7): e27664.

Perez, A., et al. (2021). "Ketogenic diet treatment in diffuse intrinsic pontine glioma in children: Retrospective analysis of feasibility, safety, and survival data." Cancer Rep (Hoboken) 4(5): e1383.

BACKGROUND: Diffuse intrinsic pontine glioma (DIPG) is one of the most devastating diseases among children with cancer, thus novel strategies are urgently needed. AIMS: We retrospectively evaluated DIPG patients exposed to the carbohydrate restricted ketogenic diet (

Perez-Torres-Lobato, M. R., et al. (2021). "Paediatric Horner syndrome. A case series of 14 patients in a tertiary hospital." Arch Soc Esp Oftalmol (Engl Ed) 96(7): 356-365.

BACKGROUND: Horner syndrome (HS) is characterised by the triad of upper eyelid ptosis, miosis, and facial anhidrosis. Due to its wide variety of causes, it can occur at any age, and is uncommon in paediatrics. The aetiology and diagnostic approach of paediatric HS (PHS) is

Peters, D. R., et al. (2021). "Orbitozygomatic Craniotomy via an Eyebrow Incision: Management of the Opened Frontal Sinus." J Neurol Surg B Skull Base 82(Suppl 3): e190-e195.

Background The eyebrow orbitozygomatic craniotomy is a minimally invasive approach that can access a wide variety of lesions. Unintentional breach of the frontal sinus frequently occurs and has been cited as a reason to avoid this approach. Lack of access to a large peric

Petit, C. J. and R. A. Ligon (2021). "Direct Percutaneous Transthoracic Cardiac Access for Recanalization of Longstanding Branch Pulmonary Artery Atresia." JACC Case Rep 3(2): 180-186.

We describe the use of direct percutaneous cardiac access to recanalize an atretic right pulmonary artery in an adolescent with complex congenital heart disease and right heart failure. This case highlights the problems associated with loss of central venous access and pote

Pfluger, M. J., et al. (2021). "Intrahepatic De Novo Tumors in Liver Recipients are Highly Associated With Recurrent Viral Hepatitis." J Clin Exp Hepatol 11(4): 435-442.

Background/Aims: Long-term survival of liver transplant recipients is endangered by tumorigenesis at different sites. Little is known about primary de novo tumors developing in the graft. Methods: We analyzed the follow-up data of 2731 liver recipients that were transplan

Piccolo, G., et al. (2021). "Complex Neurological Phenotype Associated with a De Novo DHDDS Mutation in a Boy with Intellectual Disability, Refractory Epilepsy, and Movement Disorder." J Pediatr Genet 10(3): 236-238.

Mutations in the DHDDS gene (MIM: 617836), encoding a subunit of dehydrodolichyl diphosphate synthase complex, have been recently implicated in very rare neurodevelopmental diseases. In total, five individuals carrying two de novo mutations in DHDDS have been rep

Pojskic, M., et al. (2020). "Microsurgical Management of Low-Grade Spinal Cord Astrocytoma in Adults: A Personal Case Series Report and Brief Literature Review." Acta Clin Croat 59(3): 505-512.

Astrocytoma is the second most common intramedullary tumor of predominantly low-grade malignancy in adult patients. Adult astrocytomas have better-quality prognosis compared with astrocytomas in children. Although a standardized surgical management protocol fo

Pontell, M., et al. (2021). "Interfacility Transfers for Isolated Craniomaxillofacial Trauma: Perspectives of the Facial Trauma Surgeon." Craniomaxillofac Trauma Reconstr 14(3): 201-208.

Study Design: Secondary overtriage is a burden to the medical system. Unnecessary transfers overload trauma centers, occupy emergency transfer resources, and delay definitive patient care. Craniomaxillofacial (CMF) trauma, especially in isolation, is a frequent culprit. Ob

Pooboni, S. K. (2021). "Neonatal extra corporeal membrane oxygenation." Indian J Thorac Cardiovasc Surg 37(4): 411-420.

Extracorporeal life support (ECLS) has been proven to be very useful in the neonatal period. For reversible respiratory and cardiac disorders, when maximal conventional measures have failed to provide life support, extracorporeal membrane oxygenation (ECMO) becomes

Porper, K., et al. (2021). "Dietary-Induced Ketogenesis: Adults Are Not Children." Nutrients 13(9).

There is increasing interest in the use of a ketogenic diet for various adult disorders; however, the ability of adults to generate ketones is unknown. Our goal was to challenge the hypothesis that there would be no difference between adults and children regarding their abil

Potekhina, Y. P., et al. (2021). "Mechanosensitivity of Cells and Its Role in the Regulation of Physiological Functions and the Implementation of Physiotherapeutic Effects (Review)." Sovrem Tekhnologii Med 12(4): 77-89.

Regulatory signals in the body are not limited to chemical and electrical ones. There is another type of important signals for cells: those are mechanical signals (coming from the environment or arising from within the body), which have been less known in the literature. Th

Prestowitz, S. (2020). "Public Health Implications for the Future: : Unifying a Fragmented System." Dela J Public Health 6(3): 84-85.

Price, J., et al. (2020). "Toolkit for Emotional Coping for Healthcare Staff (TECHS):: Helping Healthcare Workers Cope with the Demands of COVID-19." Dela J Public Health 6(3): 10-13.

In response to the COVID-19 pandemic, healthcare workers (HCWs) are experiencing elevated levels of emotional distress, including traumatic stress, which may continue for months and years to come. To support HCWs, the Center for Pediatric Traumatic Stress created th

Qiu, B., et al. (2020). "Bioprinting Neural Systems to Model Central Nervous System Diseases." Adv Funct Mater 30(44): 1910250.

To date, pharmaceutical progresses in central nervous system (CNS) diseases are clearly hampered by the lack of suitable disease models. Indeed, animal models do not faithfully represent human neurodegenerative processes and human in vitro 2D cell culture systems car

Radhakrishnan, R. C., et al. (2021). "Lentiform Fork Sign in a Child on Hemodialysis." Indian J Nephrol 31(3): 329-330.

Rahimzadeh, G., et al. (2021). "Evaluation of in-situ gel-forming eye drop containing bacteriophage against Pseudomonas aeruginosa keratoconjunctivitis in vivo." Bioimpacts 11(4): 281-287.

Introduction: Eradication of Pseudomonas aeruginosa has become increasingly difficult due to its remarkable capacity to resist antibiotics. Bacteriophages have been suggested as an alternative treatment for bacterial infections. Methods: In-situ gel-forming eye drop conta

Rajab, T. K. and M. N. Kavarana (2021). "Commentary: One size might not fit all: Planning ventricular assist device implantation in young children." JTCVS Tech 6: 142-143.

Rajab, T. K. and M. N. Kavarana (2021). "Commentary: Please mind the gap between the superior vena cava and the right atrial appendage." JTCVS Tech 5: 99-100.

Ramos-Lopes, J., et al. (2021). "A De Novo BSCL2 Gene S90L Mutation in a Progressive Tetraparesis with Urinary Dysfunction and Corpus Callosum Involvement." J Pediatr Genet 10(3): 253-258.

A Silver syndrome is a rare autosomal dominant spastic paraparesis in which spasticity of the lower limbs is accompanied by amyotrophy of the small hand muscles. The causative gene is the Berardinelli-Seip congenital lipodystrophy 2 (BSCL2) , which is related to a spectru

Ravanshad, Y., et al. (2021). "Analgesia for Pediatric Arteriovenous Fistula Cannulation in Hemodialytic Patients: A Comparison of Lidocaine Gel, Lidocaine Spray, and Needle Plate." Indian J Nephrol 31(4): 349-352.

Background: Children undergoing hemodialysis (HD) via arteriovenous fistula (AVF) experience approximately 300 painful punctures per year which may lead to non-compliance with HD. This study was conducted to show the effect of local anesthetics on pain perception ir

Ray, A. and E. Wyllie (2005). "Treatment options and paradigms in childhood temporal lobe epilepsy." Expert Rev Neurother 5(6): 785-801.

Temporal lobe epilepsy in adults is a relatively homogenous syndrome with hippocampal sclerosis being its most common pathologic substrate. In the pediatric age group, low-grade neoplasms and cortical dysplasia are much more common than hippocampal sclerosis. Pec

Razavian, N., et al. (2020). "A validated, real-time prediction model for favorable outcomes in hospitalized COVID-19 patients." NPJ Digit Med 3(1): 130.

The COVID-19 pandemic has challenged front-line clinical decision-making, leading to numerous published prognostic tools. However, few models have been prospectively validated and none report implementation in practice. Here, we use 3345 retrospective and 474 pros

Richards, K. A., et al. (2020). "Recombinant HA-based vaccine outperforms split and subunit vaccines in elicitation of influenza-specific CD4 T cells and CD4 T cell-dependent antibody responses in humans." NPJ Vaccines 5(1): 77.

Although traditional egg-based inactivated influenza vaccines can protect against infection, there have been significant efforts to develop improved formats to overcome disadvantages of this platform. Here, we have assessed human CD4 T cell responses to a traditional eg

Rios-Barnes, M., et al. (2021). "The Spectrum of COVID-19 Disease in Adolescents." Arch Bronconeumol 57: 84-85.

Rodd, C., et al. (2021). "World Health Organization growth standards: How do Canadian children measure up?" Paediatr Child Health 26(5): e208-e214.

Background: World Health Organization (WHO) growth standards for children aged 0 to 5 years describe growth under optimal conditions and were adopted for use in Canada in 2012. We are seeking to validate these charts in a well-characterized, longitudinal cohort of he

Roeper, R., et al. (2021). "A preterm infant with hematuria." Paediatr Child Health 26(7): 388-389.

Rogers, S., et al. (2021). "Clinical utility of correction factors for febrile young infants with traumatic lumbar punctures." Paediatr Child Health 26(6): e258-e264.

Objectives: Correction factors have been proposed for traumatic lumbar punctures (LPs) in febrile young infants. However, no studies have assessed their diagnostic utility. We sought to determine the proportion of traumatic LPs safely reclassified as low risk for bacterial m

Roksmall o, C. a. C. E. A., et al. (2021). "Technologies for Prediction of Preeclampsia." Sovrem Tekhnologii Med 12(5): 78-84.

The aim of the study was to develop technologies for predicting the development of preeclampsia (PE) based on biomedical and molecular-genetic predictors and the calculation of individual risks for this pregnancy complication. Materials and Methods: The study involved

Ross, M. K., et al. (2020). "Accuracy of Asthma Computable Phenotypes to Identify Pediatric Asthma at an Academic Institution." Methods Inf Med 59(6): 219-226.

OBJECTIVES: Asthma is a heterogenous condition with significant diagnostic complexity, including variations in symptoms and temporal criteria. The disease can be difficult for clinicians to diagnose accurately. Properly identifying asthma patients from the electronic health

Rothman, J. M., et al. (2020). "Exclusion of an Azygos Vein Varix With a Covered Stent." JACC Case Rep 2(12): 1911-1916.

An azygos vein varix was incidentally discovered in a 26-year-old man. Owing to the potential risk of pulmonary emboli, we implanted a covered stent in the superior vena cava, effectively excluding the varix. Eighth months later, the varix was thrombosed and involuted. (L

Rowold, D. J., et al. (2020). "The Y-chromosome of the Soliga, an ancient forest-dwelling tribe of South India." Gene 763S: 100026.

A previous autosomal STR study provided evidence of a connection between the ancient Soliga tribe at the southern tip of the Indian subcontinent and Australian aboriginal populations, possibly reflecting an eastbound coastal migration circa (15 Kya). The Soliga are consic

Runkel, B. G., et al. (2020). "Brain Abscess and the Nonfenestrated Fontan Circulation." JACC Case Rep 2(7): 1029-1032.

Brain abscess is a rare but life-threatening condition. Intracardiac or extracardiac right-to-left shunting in patients with unrepaired cyanotic congenital heart disease, pulmonary arteriovenous malformations, or venovenous collaterals allows microbes unfiltered access to th

S, L. K., et al. (2020). "Comparative study of prophylaxis with high and low doses of voriconazole in children with malignancy." Curr Med Mycol 6(4): 27-34.
Background and Purpose: Children with acute myeloid leukemia and relapses of leukemia are at high risk of developing fungal infections and need antifungal prophylaxis. This study aimed to compare the efficacy and toxicity of two different dosage regimens of voriconazol

Saad, K., et al. (2021). "Prevalence and associated risk factors of recurrent otitis media with effusion in children in Upper Egypt." World J Otorhinolaryngol Head Neck Surg 7(4): 280-284.
Objective: We conducted this study to determine the associations of possible risk factors and prevalence of recurrent otitis media with effusion (OME) in a cohort of children in Upper Egypt. Methods: This was a cross-sectional study undertaken in two tertiary referral cent

Saeed, A., et al. (2020). "Clinical presentation of paediatric patients with COVID-19 admitted to a single paediatric intensive care unit (PICU) in Iran." BMJ Paediatr Open 4(1): e000715.
Objectives: To describe the clinical characteristics of paediatric patients admitted to a single paediatric intensive care unit (PICU) in Iran with COVID-19. Methods: A cross-sectional study of paediatric patients who were admitted to a COVID-19-dedicated PICU from 16 Marc

Sagar, P. and K. Sivakumar (2021). "A covered stent used in aortic coarctation migrates proximally during deployment causing transverse arch obstruction: Transcatheter repositioning after one month." J Cardiol Cases 24(1): 23-26.
Stent angioplasty with covered stents to avoid risk of aortic injury is the preferred mode of management of coarctation of aorta in adults and older children. Severe isthmic coarctation in an adult male with uncontrolled systemic hypertension was managed at another insti

Said, S. M. (2020). "Commentary: Aortic homograft for tension-free caval translocation in adults: The resurrection of the Ehrenhaft operation." JTCVS Tech 4: 275-276.

Said, S. M. (2020). "Commentary: Cor triatriatum dexter: A tale of 2 horns." JTCVS Tech 4: 259-260.

Said, S. M. (2021). "Minimally invasive pulmonary valve replacement via left anterior minithoracotomy." JTCVS Tech 6: 127-129.

Said, S. M., et al. (2020). "Aortic uncrossing procedure: When the right becomes left." JTCVS Tech 4: 239-242.

Sainz, T., et al. (2021). "Lung Ultrasound for Evaluation of COVID-19 in Children." Arch Bronconeumol 57: 94-96.

Salama, K., et al. (2020). "The relationships between pancreatic T2* values and pancreatic iron loading with cardiac dysfunctions, hepatic and cardiac iron siderosis among Egyptian children and young adults with beta-thalassaemia major and sickle cell disease: a cross-sectional study
Background: Cardiac, hepatic and pancreatic T2* measured by magnetic resonance imaging (MRI) has been proven to be an accurate and non-invasive method for measuring iron overload in iron overload conditions. There is accumulating evidence that pancreatic iron can

Sanchez, R. E., et al. (2021). "Case Report: Acute Abdominal Pain as Presentation of Pneumonia and Acute Pancreatitis in a Pediatric Patient With COVID-19." JPGN Rep 2(1): e011.
Abdominal pain, nausea, and vomiting are known gastrointestinal symptoms of symptomatic SARS-CoV-2 infection (COVID-19 disease) in pediatric patients.(1) There is little literature regarding pancreatitis in COVID-19. We describe a 16-year-old male diagnosed with acute

Sanchez-Pinto, L. N., et al. (2020). "Sepsis hysteria? Not for children." Lancet 396(10259): 1332-1333.

Sathyan, A., et al. (2021). "Antiepileptic Drugs-Induced Enuresis in Children: An Overview." J Pharm Technol 37(2): 114-119.
Objective: To evaluate enuresis-induced antiepileptic drugs in children. Data Sources: A PubMed search (1917 to July 2020) was performed using the following keywords and associated medical subject headings: antiepileptic drugs, enuresis, pediatric population, drug-induc

Sato, T., et al. (2020). "First clinical application of radiofrequency identification (RFID) marking system-Precise localization of a small lung nodule." JTCVS Tech 4: 301-304.
Objectives: Precise small lung nodule resection is challenging in minimally invasive thoracoscopic surgery. Various methods that help surgeons to locate the target nodule have been devised; however, the ideal way that satisfies the demand has not yet been realized. We ha

Schiwe, D., et al. (2021). "Diagnostic performance of the physical activity-related question of the GINA questionnaire to detect exercise-induced bronchoconstriction in asthma." An Pediatr (Engl Ed) 95(1): 40-47.
INTRODUCTION: The aim of the study was to evaluate the diagnostic performance of the item concerning physical activity of the Global Initiative for Asthma (GINA) asthma control questionnaire for detection of exercise-induced bronchoconstriction (EIB) in children and ad

Schuelke, T. and J. Rubenstein (2020). "Dignity Therapy in Pediatrics: A Case Series." Palliat Med Rep 1(1): 156-160.
Objective: To report our first case series of Dignity Therapy modified for a pediatric palliative care population. Background: Dignity Therapy has been utilized successfully with terminally ill adult patients to help restore a sense of dignity and personhood as well as cope with

Schuler, B. R., et al. (2020). "Poverty and Food Insecurity Predict Mealtime Structure: Mediating Pathways of Parent Disciplinary Practices and Depressive Symptoms." J Child Fam Stud 29(11): 3169-3183.
Objective: Structured, well-organized mealtime routines can provide many physical and mental health benefits for children. Poverty and food insecurity (FI) are socioeconomic risk factors for less effective mealtime routines. However, the specific mechanisms by which thes

Sedler, J., et al. (2020). "Quality Improvement Analyses Revealed a Hidden Shift Following a Retrospective Study on Breastfeeding Rates." Pediatr Qual Saf 5(5): e347.
Factors affecting exclusive breastfeeding rates are complex. Evaluations for early-onset sepsis can negatively impact breastfeeding success. We sought to determine whether implementing an algorithm utilizing the sepsis risk score (SRS) in chorioamnionitis-exposed newbo

Segar, J. and J. G. Jetton (2020). "Diuretic use, acute kidney injury, and premature infants: the call for evidence-based guidelines." Pediatr Nephrol 36(12): 3807-3811.

Serrano-Gomez, S. J., et al. (2020). Molecular Profiles of Breast Cancer in Hispanic/Latina. Advancing the Science of Cancer in Latinos. A. G. Ramirez and E. J. Trapido. Cham (CH): 103-109.
Breast cancer (BC) is the most common malignancy in women worldwide. Different risk factors are associated with the disease which is classified into several intrinsic subtypes according to expression of hormone receptors (estrogen and progesterone) and human epiderm

Seyfried, B. T., et al. (2009). "Targeting energy metabolism in brain cancer through calorie restriction and the ketogenic diet." J Cancer Res Ther 5 Suppl 1: S7-15.

Malignant brain tumors are a significant health problem in children and adults and are largely unmanageable. As a metabolic disorder involving the dysregulation of glycolysis and respiration (the Warburg effect), malignant brain cancer can be managed through changes in

Seyfried, T. N., et al. (2011). "Metabolic management of brain cancer." Biochim Biophys Acta 1807(6): 577-594.
Malignant brain tumors are a significant health problem in children and adults. Conventional therapeutic approaches have been largely unsuccessful in providing long-term management. As primarily a metabolic disease, malignant brain cancer can be managed through cha

Seyfried, T. N. and P. Mukherjee (2005). "Targeting energy metabolism in brain cancer: review and hypothesis." Nutr Metab (Lond) 2: 30.
Malignant brain tumors are a significant health problem in children and adults and are often unmanageable. As a metabolic disorder involving the dysregulation of glycolysis and respiration, malignant brain cancer is potentially manageable through changes in metabolic er

Shahein, A. R., et al. (2021). "Phlegmonous Ileocolitis as a Presentation of Post SARS-CoV-2 (COVID-19) Multisystem Inflammatory Syndrome in Children." JPGN Rep 2(1): e040.

Shakeri, F., et al. (2021). "Correlation between Ethical Intelligence, Quality of Work Life and Caring Behaviour of Paediatric Nurses." Nurs Open 8(3): 1168-1174.
AIMS: The aim of this study is to determine correlation between paediatric nurses' "ethical intelligence" with "quality of work life" and "caring behaviour." DESIGN: Descriptive correlational study. METHODS: Data were collected with EIQ, QWL and CBI. Two hundred and on

Sharawat, I. K., et al. (2021). "Distinctive Imaging in a Toddler with Joubert's Syndrome." Ann Indian Acad Neurol 24(2): 253-254.

Sharma, R., et al. (2021). "Next-Generation Sequencing Based Approach to Identify Underlying Genetic Defects of Glanzmann Thrombasthenia." Indian J Hematol Blood Transfus 37(3): 414-421.
Glanzmann thrombasthenia (GT) is an autosomal recessive platelet function disorder characterized by mucocutaneous bleeding as the most common clinical phenotype. Patients with GT have normal platelet counts, platelet morphology but reduced platelet aggregation in

Shehata, M., et al. (2020). "A Deep Learning-Based Cad System for Renal Allograft Assessment: Diffusion, Bold, and Clinical Biomarkers." Proc Int Conf Image Proc 2020: 355-359.
Recently, studies for non-invasive renal transplant evaluation have been explored to control allograft rejection. In this paper, a computer-aided diagnostic system has been developed to accommodate with an early-stage renal transplant status assessment, called RT-CAD. C

Shen, Z., et al. (2020). "Huoxin pill attenuates myocardial infarction-induced apoptosis and fibrosis via suppression of p53 and TGF-beta1/Smad2/3 pathways." Biomed Pharmacother 130: 110618.
Huoxin Pill (HXP), a Traditional Chinese Medicine, is used widely to treat patients with coronary heart disease and angina pectoris in China. However, the underlying protective mechanism of HXP on cardiac apoptosis and fibrosis has never been evaluated. Therefore, the ai

Sherlock, M. E., et al. (2021). "Clinical and Laboratory Characteristics Are Associated With Biologic Therapy Use in Pediatric Inflammatory Bowel Disease: A Retrospective Cohort Study." J Can Assoc Gastroenterol 4(5): e92-e100.
Background: Biologic agents are a highly useful class of medications for treating inflammatory bowel disease (IBD). Limited evidence exists to guide initiation of biologic therapy, especially in pediatric patients. It is unclear if disease severity is connected to biologic response

Shrestha, S. and S. K. Adhikari (2020). "Colonic Duplication Cyst in an Adult Woman: A Case Report." JNMA J Nepal Med Assoc 58(231): 948-950.
Intestinal duplications are rare congenital anomaly found in pediatric age group. Although, ileum is the most common site, there are cases of colonic duplications even in the adult. We report a case of 43 years woman presented with chronic constipation, intermittent colic

Shrestha, S., et al. (2020). "Knowledge, Attitude and Practices among Mothers of Children 6 to 24 months of Age Regarding Complementary Feeding." JNMA J Nepal Med Assoc 58(230): 758-763.
INTRODUCTION: Complementary foods fill the gap between the total nutritional needs of the child and the amounts provided by breast milk. Inappropriate feeding practices are a major cause of the onset of malnutrition in young children. The objective of this study was to

Siddicky, S. F., et al. (2021). "Exploring infant hip position and muscle activity in common baby gear and orthopedic devices." J Orthop Res 39(5): 941-949.
Infant positioning in daily life may affect hip development. While neonatal animal studies indicate detrimental relationships between inactive lower extremities and hip development and dysplasia, no research has explored infant hip biomechanics experimentally. This stud

Silahli, M., et al. (2020). "Prophylactic intravenous paracetamol use in extremely premature infants for patent ductus arteriosus." J Basic Clin Physiol Pharmacol 32(5): 943-950.
OBJECTIVES: Patency of ductus arteriosus (PDA) is a very common problem among extremely low birth weight infants (ELBW). Hemodynamic instability caused by PDA is associated with important morbidities. This study aims to evaluate the effects of prophylactic intraveno

Singer, L. T., et al. (2020). "Fifty Years of Research on Prenatal Substances: Lessons Learned for the Opioid Epidemic." Advers Resil Sci 1(4): 223-234.
Current efforts to design research on developmental effects of prenatal opioid exposure can benefit from knowledge gained from 50 years of studies of fetal alcohol and prenatal drug exposures such as cocaine. Scientific advances in neurobiology, developmental psychopa

Singh, A., et al. (2020). "Osteopetrosis and renal tubular acidosis: Answers." Pediatr Nephrol 36(12): 4055-4059.

Singh, A., et al. (2020). "Osteopetrosis and renal tubular acidosis: Questions." Pediatr Nephrol 36(12): 4051-4053.

Slovis, J. C., et al. (2020). "The physiologic response to rescue therapy with vasopressin versus epinephrine during experimental pediatric cardiac arrest." Resusc Plus 4: 100050.
Aim: Compare vasopressin to a second dose of epinephrine as rescue therapy after ineffective initial doses of epinephrine in diverse models of pediatric in-hospital cardiac arrest. Methods: 67 one- to three-month old female swine (10-30 kg) in six experimental cohorts fror

Smith, A. C., et al. (2020). "Queensland Telepaediatric Service: A Review of the First 15 Years of Service." Front Digit Health 2: 587452.
In November 2000, the Queensland Telepaediatric Service (QTS) was established in Brisbane, Australia, to support the delivery of telehealth services to patients and clinicians in regional and remote locations. The QTS was built on a centralized coordination model, where te

Somasundaram, P. S., et al. (2020). "Analysis of common genetic mutations in a cohort of children with salt wasting form of Congenital Adrenal Hyperplasia." Ceylon Med J 65(4): 95-104.
Introduction: Steroid hydroxylase deficiency due to CYP21A2 gene mutation is the most common cause of Congenital Adrenal Hyperplasia (CAH). Mutation spectrum in Sri Lankan CAH patients has not been investigated adequately. Objectives: This study attempted to study

Sood, V. and M. S. Si (2020). "Commentary: Novel repair technique for scimitar syndrome." JTCVS Tech 4: 217-218.

Sood, V. and M. S. Si (2020). "Commentary: Staged cone repair for Ebstein anomaly." JTCVS Tech 3: 288-289.

Sriharan, A., et al. (2020). "Occupational Stress, Burnout, and Depression in Women in Healthcare During COVID-19 Pandemic: Rapid Scoping Review." Front Glob Womens Health 1: 596690.
Objectives: The overall objectives of this rapid scoping review are to (a) identify the common triggers of stress, burnout, and depression faced by women in health care during the COVID-19 pandemic, and (b) explore individual-, organizational-, and systems-level interventi

Stafford, P., et al. (2010). "The ketogenic diet reverses gene expression patterns and reduces reactive oxygen species levels when used as an adjuvant therapy for glioma." Nutr Metab (Lond) 7: 74.
BACKGROUND: Malignant brain tumors affect people of all ages and are the second leading cause of cancer deaths in children. While current treatments are effective and improve survival, there remains a substantial need for more efficacious therapeutic modalities. The k

Stankovic, N., et al. (2020). "Socioeconomic status and in-hospital cardiac arrest: A systematic review." Resusc Plus 3: 100016.
Aim: To perform a review of the literature on the association between socioeconomic status and risk of and outcomes after in-hospital cardiac arrest. Data sources: PubMed and Embase were searched on January 24, 2020 for studies evaluating the association between soc

Stellato, P., et al. (2020). "Successful management plan of COVID-19 in a pediatric hemato-oncology department: a single-centre experience." BMJ Paediatr Open 4(1): e000818.
COVID-19 pandemic raised concern about management of patients with paediatric cancer. We present the operating system that the Hemato-Oncology Department of the Santobono-Pausilipon Hospital applied. We divided our department in three zones: surveillance and

Stone, J. K., et al. (2021). "Prevalence of Gastroduodenal Polyps in Children With Familial Adenomatous Polyposis." J Can Assoc Gastroenterol 4(5): e101-e109.
Objective: To assess the prevalence of upper gastrointestinal adenomatous polyps in a cohort of pediatric familial adenomatous polyposis (FAP) patients to determine if early screening is warranted. Study Design: All 11 pediatric FAP patients diagnosed in Manitoba betwee

Stultz, J. S., et al. (2021). "Analysis of Community-Acquired Urinary Tract Infection Treatment in Pediatric Patients Requiring Hospitalization: Opportunity for Use of Narrower Spectrum Antibiotics." J Pharm Technol 37(2): 79-88.
Background: The most narrow-spectrum antibiotic possible should be used for empiric and definitive treatment of pediatric urinary tract infections (UTIs). Objectives: The objectives of this study were to determine an appropriate narrow-spectrum antibiotic for empiric UTI

Sudhanthar, S., et al. (2020). "Simulation 2.0: Integrating Basic Scientists and Clinicians in a Simulation Environment." Med Sci Educ 30(4): 1367-1372.
An increasing number of medical schools are implementing curricular changes that better integrate clinical and basic sciences throughout all four years of medical school. One of the most frequently cited reasons is to improve medical student clinical reasoning skills while s

Sun, D., et al. (2021). "Multi-Ancestry Genome-wide Association Study Accounting for Gene-Psychosocial Factor Interactions Identifies Novel Loci for Blood Pressure Traits." HGG Adv 2(1).
Psychological and social factors are known to influence blood pressure (BP) and risk of hypertension and associated cardiovascular diseases. To identify novel BP loci, we carried out genome-wide association meta-analyses of systolic, diastolic, pulse, and mean arterial BP ta

Tabaac, A. R., et al. (2021). "Sexual and reproductive health information: Disparities across sexual orientation groups in two cohorts of U.S. women." Sex Res Social Policy 18(3): 612-620.
Introduction: Limited research exists about how receiving/seeking sexual and reproductive health (SRH) information differs by sexual orientation. Our goal was to identify how sources and topics of SRH information differed by sexual orientation during adolescence in a sam

Taber, P., et al. (2020). "Physicians' strategies for using family history data: having the data is not the same as using the data." JAMIA Open 3(3): 378-385.
Objective: To identify needs in a clinical decision support tool development by exploring how primary care providers currently collect and use family health history (FHH). Design: Survey questionnaires and semi-structured interviews were administered to a mix of primary a

Takenouchi, T., et al. (2020). "Clinical Utility of SARS-CoV-2 Whole Genome Sequencing in Deciphering Source of Infection." J Hosp Infect.
COVID-19 caused by SARS-CoV-2 is a worldwide problem. From the standpoint of hospital infection control, determining the source of infection is critical. We conducted the present study to evaluate the efficacy of using whole genome sequencing to determine the source

Tamhankar, P. M., et al. (2021). "Clinical Characteristics, Molecular Profile, and Outcomes in Indian Patients with Glutaric Aciduria Type 1." J Pediatr Genet 10(3): 213-221.
Glutaric acidemia type 1 (GA-1, OMIM 231670) is an autosomal recessive inborn error of metabolism caused by the deficiency of glutaryl-coenzyme A (CoA) dehydrogenase with most children presenting in infancy with encephalopathy, dystonia, and macrocephaly. In this a

Tastemel Ozturk, T., et al. (2020). "Acute kidney injury in a patient with COVID-19: Answers." Pediatr Nephrol 36(12): 4111-4113.

Tastemel Ozturk, T., et al. (2020). "Acute kidney injury in a patient with COVID-19: Questions." Pediatr Nephrol 36(12): 4109-4110.

Tatsumi, K., et al. (2020). "New device for sperm preparation involving migration-gravity sedimentation without centrifugation compared with density-gradient centrifugation for normozoospermic intrauterine insemination." F S Rep 1(2): 106-112.
Objective: To investigate the efficacy of a new device for sperm preparation involving migration-gravity sedimentation without centrifugation (MIGLIS), compared with density-gradient centrifugation (DGC) for normozoospermic intrauterine insemination (IUI). Design: Retr

Teitelman, A. M., et al. (2020). "Vaccipack, A Mobile App to Promote Human Papillomavirus Vaccine Uptake Among Adolescents Aged 11 to 14 Years: Development and Usability Study." JMIR Nurs 3(1): e19503.
Background: More than 90% of human papillomavirus (HPV)-related cancers could be prevented by widespread uptake of the HPV vaccine, yet vaccine use in the United States falls short of public health goals. Objective: The purpose of this study was to describe the develo

Thiele, E. A. (2013). "Implications of dietary therapy into the 21st century: conclusion to special issue." J Child Neurol 28(8): 1054-1055.
The concept of dietary therapy for epilepsy has played an important role in the approach to the treatment of seizures for centuries--particularly with the development and utilization of the classic ketogenic diet over the past 90 years. Recently, there has been developing ir

Tisdale, C. E., et al. (2020). "The Impact of Meeting Patients with Neurological Disorders on Medical Student Empathy." Med Sci Educ 30(4): 1561-1568.
Purpose: Empathy tends to decline during medical education, typically beginning in the third year of medical school and often continuing throughout residency and the physician's medical career. The purpose of this study was to determine if first year medical student emp

Tom, K., et al. (2021). "Mast Cell Activation in the Systemic Sclerosis Esophagus." J Scleroderma Relat Disord 6(1): 77-86.

Introduction: Previously, we discovered similar esophageal gene expression patterns in patients with systemic sclerosis (SSc) and eosinophilic esophagitis (EoE) where eosinophil/mast cell-targeted therapies are beneficial. Because SSc and EoE patients experience similar es

Tor-Diez, C., et al. (2020). "Unsupervised MRI Homogenization: Application to Pediatric Anterior Visual Pathway Segmentation." Mach Learn Med Imaging 12436: 180-188.
Deep learning strategies have become ubiquitous optimization tools for medical image analysis. With the appropriate amount of data, these approaches outperform classic methodologies in a variety of image processing tasks. However, rare diseases and pediatric imaging

Torun, E. G., et al. (2021). "A Case of Pulmonary Artery Sling Anomaly with Tracheal Stenosis and Management of Difficult Airway." J Pediatr Intensive Care 10(3): 235-239.
Pulmonary artery sling is a rare congenital vascular abnormality, where the left pulmonary artery originates from the right pulmonary artery, passes between trachea, and esophagus and reaching the left hilum. Cough, wheezing, and difficulty in feeding are three major syr

Trezzi, M. (2021). "Commentary: When things just mix like you hoped." JTCVS Tech 6: 126.

Turek, J. W. and N. Ad (2020). "Commentary: A synergy of two is better than one: Modern-day heart team approach to robotic congenital heart surgery." JTCVS Tech 4: 267-268.

Turkdogan, M. and A. Akcan (2020). "Prospective Monitoring of Breastfeeding Behaviors in Primiparous Mothers with Risky and Non-risky Age Groups." Florence Nightingale J Nurs 28(3): 276-286.
AIM: This study aimed at monitoring breastfeeding behaviors in primiparous mothers with risky and non-risky age groups prospectively. METHOD: This is a kind of comparative and prospective study that was carried out in 306 mothers who were registered at seven family

Ubeda Tikkanen, A., et al. (2021). "Acquired Brain Injury in the Pediatric Intensive Care Unit: Special Considerations for Delirium Protocols." J Pediatr Intensive Care 10(4): 243-247.
The goal of this article was to highlight the overlapping nature of symptoms of delirium and acquired brain injury (ABI) in children and similarities and differences in treatment, with a focus on literature supporting an adverse effect of antipsychotic medications on recovery

Uchakin, P. N., et al. (2020). "Azithromycin Reduces Markers of Vascular Damage in Pediatric Patients With Sickle Cell Disease." J Hematol 10(4): 178-186.
Background: Immunomodulatory effects of macrolides in chronic inflammation are well known. In this study, we tested our hypothesis that azithromycin (AZT) can decrease inflammation in pediatric patients with sickle cell disease (SCD). Methods: The use of AZT as an anti

Udayabhanu, H. N., et al. (2021). "Relevance of Modified Bondy Mastoidectomy in Pediatric Cholesteatoma." Indian J Otolaryngol Head Neck Surg 73(4): 403-407.
Modified Bondy mastoidectomy is a type of canal wall down mastoidectomy well described in literature for adult patients. We present our experience with the use of modified Bondy mastoidectomy in pediatric population. Using retrospective chart review, pediatric patien

Ukwishaka, J., et al. (2020). "Pediatric self-medication use in Rwanda - a cross sectional study." Afr Health Sci 20(4): 2032-2043.
Background: Self-medication, a worldwide practice, has both benefits and risks. Many countries have regulated non-prescription medications available for use in self-medication. However, in countries such as Rwanda, where prescriptions are not required to purchase med

Vaidyanathan, S., et al. (2021). "Screen Time Exposure in Preschool Children with ADHD: A Cross-Sectional Exploratory Study from South India." Indian J Psychol Med 43(2): 125-129.
Background: Concern is mounting regarding screen exposure among young children and its association with mental health. Children with attention deficit hyperactivity disorder (ADHD) may be more vulnerable to its effects such as increased externalizing behaviors and pro

Vallance, H., et al. (2021). "Diagnostic yield from routine metabolic screening tests in evaluation of global developmental delay and intellectual disability." Paediatr Child Health 26(6): 344-348.
Global developmental delay and intellectual disability (GDD/ID) affect 3% of the paediatric population. Although inborn errors of metabolism (IEM) are not a common cause of GDD/ID, early therapeutic intervention can improve neurodevelopmental manifestations. In 201

Vallejo, F. A., et al. (2020). "The contribution of ketone bodies to glycolytic inhibition for the treatment of adult and pediatric glioblastoma." J Neurooncol 147(2): 317-326.
PURPOSE: Glioblastoma (GBM) remains one of the most lethal primary brain tumors in children and adults. Targeting tumor metabolism has emerged as a promising-targeted therapeutic strategy for GBM and characteristically resistant GBM stem-like cells (GSCs). METHOD

van Buuren, A., et al. (2021). "A 12-year-old boy with fever and weight loss." Paediatr Child Health 26(6): 323-326.

van der Feltz-Cornelis, C. M., et al. (2020). "IL-6 and hsCRP in Somatic Symptom Disorders and related disorders." Brain Behav Immun Health 9: 100176.
Interleukin 6 (IL-6) and high-sensitivity C-reactive protein (hsCRP) are biomarkers of systemic low-grade inflammation (SLI) in depression and anxiety. The question if SLI in those conditions is related to comorbid chronic medical conditions has not been resolved. DSM-5 Son

van der Louw, E., et al. (2019). "Ketogenic diet treatment in recurrent diffuse intrinsic pontine glioma in children: A safety and feasibility study." Pediatr Blood Cancer 66(3): e27561.
BACKGROUND: The mean overall survival rate of children with diffuse intrinsic pontine glioma (DIPG) is 9-11 months, with current standard treatment with fractionated radiotherapy and adjuvant chemotherapy. So far, novel therapeutic strategies have not yet resulted in s

Vasconez, W. A., et al. (2021). "Severe Diabetic Ketoacidosis in a Child with Type-1 Diabetes, Asthma, and COVID-19." J Pediatr Intensive Care 10(3): 232-234.
Little is known about the association between novel coronavirus disease 2019 (COVID-19) and type-1 diabetes in children. A 16-year-old female patient with history of type-1 diabetes was admitted for life threatening diabetic ketoacidosis (DKA). She recovered from the DK

Verrotti, A., et al. (2017). "Ketogenic diet and childhood neurological disorders other than epilepsy: an overview." Expert Rev Neurother 17(5): 461-473.
INTRODUCTION: In the last years, ketogenic diet (KD) has been experimentally utilized in various childhood neurologic disorders such as mitochondriopathies, alternating hemiplegia of childhood (AHC), brain tumors, migraine, and autism spectrum disorder (ASD). The aim

Vijayakumar, V., et al. (2021). "Double Axillary Artery and an Owl Face during an Ultrasound-guided Axillary Brachial Plexus Block." J Med Ultrasound 29(2): 132-133.

Wade, S. L., et al. (2020). "Telepsychotherapy With Children and Families: Lessons Gleaned From Two Decades of Translational Research." J Psychother Integr 30(2): 332-347.
The novel coronavirus, COVID-19, has led to sweeping changes in psychological practice and the concomitant rapid uptake of telepsychotherapy. Although telepsychotherapy is new to many clinical psychologists, there is considerable research on telepsychotherapy treatm

Wang, G., et al. (2020). "Association of ERCC gene polymorphism with osteosarcoma risk." Afr Health Sci 20(4): 1840-1848.

Background: The relationship between ERCC gene polymorphism and osteosarcoma risk / overall survival of osteosarcoma is still conflicting, and this meta-analysis was performed to assess these associations. Material and methods: The association studies were identified f

Wang, Z., et al. (2020). "Dual ARID1A/ARID1B loss leads to rapid carcinogenesis and disruptive redistribution of BAF complexes." Nat Cancer 1(9): 909-922.
SWI/SNF chromatin remodelers play critical roles in development and cancer. The causal links between SWI/SNF complex disassembly and carcinogenesis are obscured by redundancy between paralogous components. Canonical cBAF-specific paralogs ARID1A and ARID1B a

Ward, A., et al. (2020). "Machine learning and atherosclerotic cardiovascular disease risk prediction in a multi-ethnic population." NPJ Digit Med 3(1): 125.
The pooled cohort equations (PCE) predict atherosclerotic cardiovascular disease (ASCVD) risk in patients with characteristics within prespecified ranges and has uncertain performance among Asians or Hispanics. It is unknown if machine learning (ML) models can improve

Ward, S. L., et al. (2021). "Successful Rapid Desensitization to Micafungin in a Pediatric Patient." Pediatr Allergy Immunol Pulmonol 34(3): 106-108.
Introduction: Echinocandin antifungal medications including micafungin are being used more commonly in the treatment of invasive fungal infections in both pediatric and adult patients. Micafungin is also a first-line therapeutic option for candidemia and antifungal proph

Wawszczak, M., et al. (2021). "Hypersensitivity pneumonitis in children." Ann Agric Environ Med 28(2): 214-219.
INTRODUCTION: Hypersensitivity pneumonitis (HP) is one of the most common forms of interstitial lung disease in children. Due to its common association with occupational environment, it used to be considered an exclusively adult disease; however, hypersensitivity pneu

Weedin, E. A., et al. (2020). "Elevated activity levels of activating autoantibodies to the GnRH receptor in patients with polycystic ovary syndrome." F S Rep 1(3): 299-304.
Objectives: 1) To confirm the correlation of GnRH receptor (GnRHR) activating autoantibody (AAb) activity with polycystic ovary syndrome (PCOS) diagnosis in large well defined cohorts; and 2) to evaluate suppression of AAb activity with GnRH antagonist medication in tra

Wilke, A. C., et al. (2020). "SHMT2 inhibition disrupts the TCF3 transcriptional survival program in Burkitt lymphoma." Blood.
Burkitt lymphoma (BL) is an aggressive lymphoma type that is currently treated by intensive chemoimmunotherapy. Despite the favorable clinical outcome of the majority of BL patients, chemotherapy-related toxicity and disease relapse remain as major clinical challenges,

Wilson, J. A., et al. (2020). "Emicizumab Associated Rhabdomyolysis in Hemophilia A." Clin Hematol Int 2(4): 165-167.
Emicizumab is increasingly the front-line treatment for patients with Hemophilia A with or without inhibitors. Rhabdomyolysis is a syndrome of muscle necrosis and release of intracellular muscle constituents into the circulation. Creatine kinase (CK) levels are typically mar

Withrow, D., et al. (2021). "Sleep and Circadian Disruption and the Gut Microbiome-Possible Links to Dysregulated Metabolism." Curr Opin Endocr Metab Res 17: 26-37.
Insufficient sleep and circadian misalignment are associated with adverse metabolic health outcomes. Alterations in gut microbial diversity occur with insufficient sleep and circadian misalignment, which can lead to modifications in microbial structure and function. Change

Woods, R. K. (2020). "Commentary: Pumping up the definition of Fontan candidacy and an old-school trick." JTCVS Tech 3: 310.

Woods, R. K. and M. H. Salinger (2020). "Commentary: Rusty pipes, clever plumbing, and a quiet aorta." JTCVS Tech 3: 263-264.

Worley, G. and B. G. Rocque (2020). "Letter: Detailed Analysis of Hydrocephalus and Hindbrain Herniation After Prenatal and Postnatal Myelomeningocele Closure: Report From a Single Institution." Neurosurgery 87(6): E727.

Yazici, M. U., et al. (2021). "Detrimental Results of COVID-19 Fear to Child Health." J Pediatr Intensive Care 10(3): 197-201.
The outbreak of coronavirus disease 2019 (COVID-19) and its consequences have led to fear and anxiety among individuals worldwide. The risk of coronavirus transmission frightens people more than any other health problem they face. Parents have concerns about being

Yesilbas, O., et al. (2021). "Acute Myocarditis and Eculizumab Caused Severe Cholestasis in a 17-Month-Old Child Who Has Hemolytic Uremic Syndrome Associated with Shiga Toxin-Producing Escherichia coli." J Pediatr Intensive Care 10(3): 216-220.
Cardiovascular involvement is uncommon in pediatric patients with hemolytic uremic syndrome associated with Shiga toxin-producing Escherichia coli (STEC-HUS). In this case report we presented a case of 17-month-old toddler who had a sporadic type of STEC-HUS compl

Yi, H., et al. (2020). "The competence of village clinicians in the diagnosis and management of childhood epilepsy in Southwestern China and its determinants: A cross-sectional study." Lancet Reg Health West Pac 3: 100031.
Background: Due to lack of neurologists in low- and middle-income countries, communities of patients living with epilepsy are calling for task-shifting of diagnosis and management from physicians to paramedical providers in the primary health care systems to narrow the

Yildirim, A., et al. (2020). "The Effect of Education Given to Children with Functional Constipation and Fecal Incontinence and Their Mothers on Anxiety and Constipation Management." Florence Nightingale J Nurs 28(3): 321-332.
AIM: This study aimed to determine the effect of multidimensional training program on children with constipation and fecal incontinence and their mothers on anxiety and their constipation management. METHOD: The study had an experimental design with a pretest-pos

Yildirim, D. G., et al. (2021). "Milk of calcium: A rare manifestation of juvenile dermatomyositis." Arch Rheumatol 36(2): 302-304.

Yoshino, A., et al. (2020). "A case of hypoparathyroidism, sensorineural deafness, and renal dysplasia syndrome with kidney failure and recurrent pancreatitis: Answers." Pediatr Nephrol 36(12): 4071-4075.

Yoshino, A., et al. (2020). "A case of hypoparathyroidism, sensorineural deafness, and renal dysplasia syndrome with kidney failure and recurrent pancreatitis: Questions." Pediatr Nephrol 36(12): 4067-4069.

Yost, C. E., et al. (2020). "A Long-Term Survivor With Alveolar Capillary Dysplasia." JACC Case Rep 2(10): 1492-1495.
A patient with alveolar capillary dysplasia has survived more than 56 months with medical therapy. Intrauterine exposure to metformin potentially modified the severity of disease. In combination with other agents, endothelin receptor antagonists and amlodipine have be

Zar, H. J., et al. (2020). "Diagnosis and management of community-acquired pneumonia in children: South African Thoracic Society guidelines." Afr J Thorac Crit Care Med 26(3).
Background: Pneumonia remains a major cause of morbidity and mortality amongst South African children. More comprehensive immunisation regimens, strengthening of HIV programmes, improvement in socioeconomic conditions and new preventive strategies have im

Zhang, X., et al. (2020). "Magnetic Resonance Imaging-Guided Focused Ultrasound-Based Delivery of Radiolabeled Copper Nanoclusters to Diffuse Intrinsic Pontine Glioma." ACS Appl Nano Mater 3(11): 11129-11134.

Diffuse intrinsic pontine glioma (DIPG) is an invasive pediatric brainstem malignancy exclusively in children without effective treatment due to the often-intact blood-brain tumor barrier (BBTB), an impediment to the delivery of therapeutics. Herein, we used focused ultraso

Zhao, C., et al. (2020). "Photothermal Intracellular Delivery Using Gold Nanodisk Arrays." ACS Mater Lett 2(11): 1475-1483.

Local heating using pulsed laser-induced photothermal effects on plasmonic nanostructured substrates can be used for intracellular delivery applications. However, the fabrication of plasmonic nanostructured interfaces is hampered by complex nanomanufacturing scheme

Zhong, W., et al. (2021). "Neuromuscular Blocking Agents and Reversal Agents Among Hospitalized Children: A Cerner Database Study." Hosp Pharm 56(5): 424-429.

Background: Sugammadex (Bridion) was approved by the US Food and Drug Administration (FDA) in December 2015 for the reversal of neuromuscular block (NMB) induced by rocuronium and vecuronium bromide in adults undergoing surgery and approved for use in both

Zhou, Q., et al. (2021). "PGC-1alpha promotes mitochondrial respiration and biogenesis during the differentiation of hiPSCs into cardiomyocytes." Genes Dis 8(6): 891-906.

Although it is widely accepted that human induced pluripotent stem cell-derived cardiomyocytes (hiPSC-CMs) are readily available, robustly reproducible, and physiologically appropriate human cells for clinical applications and research in the cardiovascular field, hiPSC-CM

Zhou, W., et al. (2007). "The calorically restricted ketogenic diet, an effective alternative therapy for malignant brain cancer." Nutr Metab (Lond) 4: 5.

BACKGROUND: Malignant brain cancer persists as a major disease of morbidity and mortality in adults and is the second leading cause of cancer death in children. Many current therapies for malignant brain tumors fail to provide long-term management because they ineff

Zittersteijn, H. A., et al. (2020). "A Small Key for a Heavy Door: Genetic Therapies for the Treatment of Hemoglobinopathies." Front Genome Ed 2: 617780.

Throughout the past decades, the search for a treatment for severe hemoglobinopathies has gained increased interest within the scientific community. The discovery that -globin expression from intact HBG alleles complements defective HBB alleles underlying beta-thalass

Feasibility of a modified Atkins diet in glioma patients during radiation and its effect on radiation sensitization
Ketogenic diets as an adjuvant therapy in glioblastoma (the KEATING trial): study protocol for a randomised pilot study
ERGO: A pilot study of ketogenic diet in recurrent glioblastoma
The Modified Ketogenic Diet in Adults with Glioblastoma: An Evaluation of Feasibility and Deliverability within the National Health Service
Targeting metabolism with a ketogenic diet during the treatment of glioblastoma multiforme
Metabolic management of glioblastoma multiforme using standard therapy together with a restricted ketogenic diet: Case Report
Isocaloric Ketogenic Diet in Adults with High-Grade Gliomas: A Prospective Metabolic Study
Efficacy of a ketogenic diet with concomitant intranasal perillyl alcohol as a novel strategy for the therapy of recurrent glioblastoma
ERGO2: A Prospective, Randomized Trial of Calorie-Restricted Ketogenic Diet and Fasting in Addition to Reirradiation for Malignant Glioma
Ketogenic diets as an adjuvant therapy for glioblastoma (KEATING): a randomized, mixed methods, feasibility study
A ketogenic diet exerts beneficial effects on body composition of cancer patients during radiotherapy: An interim analysis of the KETOCOMP study
Impact of a ketogenic diet intervention during radiotherapy on body composition: I. Initial clinical experience with six prospectively studied patients
Efficacy of Metabolically Supported Chemotherapy Combined with Ketogenic Diet, Hyperthermia, and Hyperbaric Oxygen Therapy for Stage IV Triple-Negative Breast Cancer
Management of Glioblastoma Multiforme in a Patient Treated With Ketogenic Metabolic Therapy and Modified Standard of Care: A 24-Month Follow-Up
Favorable Effects of a Ketogenic Diet on Physical Function, Perceived Energy, and Food Cravings in Women with Ovarian or Endometrial Cancer: A Randomized, Controlled Trial
Impact of modified short-term fasting and its combination with a fasting supportive diet during chemotherapy on the incidence and severity of chemotherapy-induced toxicities in cancer patients - a controlled cross-over pilot study
Modified Atkins diet in advanced malignancies - final results of a safety and feasibility trial within the Veterans Affairs Pittsburgh Healthcare System
A Phase I clinical trial of dose-escalated metabolic therapy combined with concomitant radiation therapy in high-grade glioma
Ketogenic Metabolic Therapy, Without Chemo or Radiation, for the Long-Term Management of IDH1-Mutant Glioblastoma: An 80-Month Follow-Up Case Report
Application of Bayesian evidence synthesis to modelling the effect of ketogenic therapy on survival of high grade glioma patients
Exploring the Feasibility and Effects of a Ketogenic Diet in Patients With CNS Malignancies: A Retrospective Case Series
4-year long progression-free and symptom-free survival of a patient with recurrent glioblastoma multiforme: A case report of the Paleolithic Ketogenic Diet (PKD) used as a stand-alone treatment after failed standard oncotherapy
Treatment of glioma patients with ketogenic diets: report of two cases treated with an IRB-approved energy-restricted ketogenic diet protocol and review of the literature
Short-term fasting in glioma patients: analysis of diet diaries and metabolic parameters of the ERGO2 trial
Cancer as a metabolic disease: on the origin, management, and prevention of cancer
Changes in cerebral metabolism during ketogenic diet in patients with primary brain tumors: 1H-MRS study
Glycemic modulation in neuro-oncology: experience and future directions using a modified Atkins diet for high-grade brain tumors
Does Metabolic management of gliomas using restricted Ketogenic diet combined with hyperbaric oxygen therapy (HBOT) improve clinical outcome and reduce epileptic risk? (Poster)
Ketogenic Diet With Radiation and Chemotherapy for Newly Diagnosed Glioblastoma
Feasibility and Biological Activity of a Ketogenic/Intermittent-Fasting Diet in Patients With Glioma
Ketogenic Diet as Adjunctive Treatment in Refractory/End-stage Glioblastoma Multiforme: a Pilot Study
Patient with recurrent glioblastoma responding favorably to ketogenic diet combined with intranasal delivery of perillyl alcohol: a case report and literature review
Tumor Metabolism, the Ketogenic Diet and β-Hydroxybutyrate: Novel Approaches to Adjuvant Brain Tumor Therapy
Fasting, Fats, and Physics: Combining Ketogenic and Radiation Therapy against Cancer
Investigating the ketogenic diet as treatment for primary Aggressive Brain cancer: challenges and Lessons Learned
Ketogenic Diet for Malignant Gliomas: a Review
Glycemic modulation in neuro-oncology: experience and future directions using a modified Atkins diet for high-grade brain tumors
Neutropenia in Children Treated With Ketogenic Diet Therapy

Impact of a ketogenic diet intervention during radiotherapy on body composition: I. Initial clinical experience with six prospectively studied patients
Effects of a ketogenic diet on the quality of life in 16 patients with advanced cancer: Apilot trial
Targeting insulin inhibition as a metabolic therapy in advanced cancer: A pilot safety and feasibility dietary trial in 10 patients
The development of tumours under a ketogenic diet in association with the novel tumour marker TKTL1: A case series in general practice
A Nutritional Perspective of Ketogenic Diet in Cancer: A Narrative Review
Impact of a ketogenic diet intervention during radiotherapy on body composition: I. Initial clinical experience with six prospectively studied patients
A Ketogenic Diet Reduces Central Obesity and Serum Insulin in Women with Ovarian or Endometrial Cancer
Consuming a Ketogenic Diet while Receiving Radiation and Chemotherapy for Locally Advanced Lung Cancer and Pancreatic Cancer: The University of Iowa Experience of Two Phase 1 Clinical Trials
Ketogenic Diets Enhance Oxidative Stress and Radio-Chemo-Therapy Responses in Lung Cancer Xenografts
Growth of human gastric cancer cells in nude mice is delayed by a ketogenic diet supplemented with omega-3 fatty acids and medium-chain triglycerides
A Ketogenic Formula Prevents Tumor Progression and Cancer Cachexia by Attenuating Systemic Inflammation in Colon 26 Tumor-Bearing Mice
Metabolic reprogramming induced by ketone bodies diminishes pancreatic cancer cachexia
Optimal Control Analysis of a Mathematical Model for Breast Cancer
Carbohydrate restriction, prostate cancer growth, and the insulin-like growth factor axis
The Effects of Varying Dietary Carbohydrate and Fat Content on Survival in a Murine LNCaP Prostate Cancer Xenograft Model
Beta-hydroxybutyrate (3-OHB) can influence the energetic phenotype of breast cancer cells, but does not impact their proliferation and the response to chemotherapy or radiation
Aldo–keto reductase family 1 B10 gene silencing results in growth inhibition of colorectal cancer cells: Implication for cancer intervention
Ketogenic *HMGCS2* Is a c-Myc Target Gene Expressed in Differentiated Cells of Human Colonic Epithelium and Down-Regulated in Colon Cancer
Mathematical modeling for optimal control of breast cancer
A Ketogenic Diet Is Acceptable in Women with Ovarian and Endometrial Cancer and Has No Adverse Effects on Blood Lipids: A Randomized, Controlled Trial
The Potential Use of a Ketogenic Diet in Pancreatobiliary Cancer Patients After Pancreatectomy
Effects of Ketogenic metabolic therapy on patients with breast cancer: A randomized controlled clinical trial
A ketogenic diet exerts beneficial effects on body composition of cancer patients during radiotherapy: An interim analysis of the KETOCOMP study
The Impact of a Ketogenic Dietary Intervention on the Quality of Life ofStage II and III Cancer Patients: A Randomized Controlled Trial inthe Caribbean
A ketogenic diet consumed during radiotherapy improves several aspects of quality of life and metabolic health in women with breast cancer
Impact of a ketogenic diet intervention during radiotherapy on body composition: III-final results of the KETOCOMP study for breast cancer patients
Low Carb and Ketogenic Diets Increase Quality of Life, Physical Performance, Body Composition, and Metabolic Health of Women with Breast Cancer
Ketogenic diets consumed during radio-chemotherapy have beneficial effects on quality of life and metabolic health in patients with rectal cancer
Cancer cachexia: influence of systemic ketosis on substrate levels and nitrogen metabolism
Total nutritional manipulation in humans: report of acancer patient
Effects of Pre-surgical Vitamin D Supplementation and Ketogenic Diet in a Patient with Recurrent Breast Cancer
Effect of energy substrate manipulation on tumour cell proliferation in parenterally fed cancer patients
Glucose-based total parenteral nutrition does not stimulate glucose uptake by humans tumours
Decline of Lactate in Tumor Tissue After Ketogenic Diet: In Vivo Microdialysis Study in Patients with Head and Neck Cancer
Reduction of weight loss and tumour size in a cachexia model by a high fat diet
Halted Progression of Soft Palate Cancer in a Patient Treated with the Paleolithic Ketogenic Diet Alone: A 20-months Follow-up
Clinical observation of a novel, complementary, immunotherapeutic approach based on ketogenic diet, chondroitin sulfate, vitamin D 3, oleic acid and a fermented milk and colostrum product
Halted progression of soft palate cancer in a patient treated with the paleolithic ketogenic diet alone
Efficacy of metabolically supported chemotherapy combined with ketogenic diet, hyperthermia, and hyperbaric oxygen therapy for stage IV triple-negative breast cancer
Complete cessation of recurrent cervical intraepithelial neoplasia (CIN) by the paleolithic ketogenic diet: a case report
Effects of a high-fat diet on body composition in cancer patients receiving chemotherapy: a randomized controlled study.
Metabolic Treatment of Cancer: Intermediate Results of a Prospective Case Series
The effects of short-term fasting on tolerance to (neo) adjuvant chemotherapy in HER2-negative breast cancer patients: a randomized pilot study
Differential effects of patient-related factors on the outcome of radiation therapy for rectal cancer
Favorable Effects of a Ketogenic Diet on Physical Function, Perceived Energy, and Food Cravings in Women with Ovarian or Endometrial Cancer: A Randomized, Controlled Trial
Feasibility study of metabolically supported chemotherapy with weekly carboplatin/paclitaxel combined with ketogenic diet, hyperthermia and hyperbaric oxygen therapy in metastatic non-small cell lung cancer
Ketogenic diets: from cancer to mitochondrial diseasesand beyond
Beneficial effects of ketogenic diets for cancer patients: a realist review with focus on evidence and confirmation
When less may be more: calorie restriction and response to cancer therapy
The infuence of diet on anti-cancer immune responsiveness
Selectively starving cancer cells through dietary manipulation: methods and clinical implications
The Pros and Cons of Low Carbohydrate and Ketogenic Diets in the Prevention and Treatment of Cancer
Perspective: Do Fasting, Caloric Restriction, and Diets Increase Sensitivity to Radiotherapy? ALiterature Review
The use of ketogenic diets in cancer patients: a systematic review
Restricted Calorie Ketogenic Diet for the Treatment of Glioblastoma Multiforme
SIGNIFICANCE OF LOW-CARBOHYDRATE DIETS AND FASTING IN PATIENTS WITH CANCER

Ketogenic Diet in Advanced Cancer: A Pilot Feasibility and Safety Trial in the Veterans Affairs Cancer Patient Population
Knowledge Translation of Low Carbohydrate Diet Intervention in Cancer Survivorship: From Basic Science to Clinical Practice and Policy Making
Ketogenic Diet in Cancer Prevention and Therapy: Molecular Targets and Therapeutic Opportunities
Wilhelm Brünings' forgotten contribution to the metabolic treatment of cancer utilizing hypoglycemia and a very low carbohydrate (ketogenic) diet
Complete Cessation of Recurrent Cervical Intraepithelial Neoplasia (CIN) by the Paleolithic Ketogenic Diet: A Case Report
Ketogenic Diet in Refractory Childhood Epilepsy: Starting With a Liquid Formulation in an Outpatient Setting
Safety and Eectiveness of the Prolonged Treatment of Children with a Ketogenic Diet
Incidence of potential adverse events during hospital-based ketogenic diet initiation among children with drug-resistant epilepsy
The efficacy comparison of classic ketogenic diet and modified Atkins diet in children with refractory epilepsy: a clinical trial
Cognitive benefits of the ketogenic diet in patients with epilepsy: A systematic overview
Use of ketogenic diet therapy in infants with epilepsy: A systematic review and meta-analysis
Ketogenic diet effects on 52 children with pharmacoresistant epileptic encephalopathy: A clinical prospective study
A Prospective Study on Changes in Nutritional Status and Growth Following Two Years of Ketogenic Diet (KD) Therapy in Children with Refractory Epilepsy
Impact of the Ketogenic Diet on Linear Growth in Children: A Single-Center Retrospective Analysis of 34 Cases
Efficacy and Safety of a Ketogenic Diet in Children and Adolescents with Refractory Epilepsy—A Review
Ketogenic diet treatment of children in the intensive care unit: Safety, tolerability, and effectiveness
Short-term and long-term efficacy of classical ketogenic diet and modified Atkins diet in children and adolescents with epilepsy: A systematic review and meta-analysis
The ketogenic diet influences taxonomic and functional composition of the gut microbiota in children with severe epilepsy
A Systematic Review of the Quality of Life for Families Supporting a Child Consuming the Ketogenic Diet for Seizure Reduction
Efficacy of KetogenicDiet,ModifiedAtkinsDiet,andLowGlycemicIndex TherapyDietAmongChildrenWithDrug-ResistantEpilepsy ARandomizedClinical Trial
The Effect of Ketogenic Diet Treatment in Drug-resistant Epilepsies of Childhood
Ketogenic diet and other dietary treatments for epilepsy (reviw)
Cognitive and behavioral impact of the ketogenic diet in children and adolescents with refractory epilepsy: A randomized controlled trial
Can an early 24-hour EEG predict the response to the ketogenic diet? A prospective study in 34 children and adults with refractory epilepsy treated with the ketogenic diet
Valproate effect on ketosis in children under ketogenic diet
Growth of children on the ketogenic diet
The ketogenic diet for the treatment of childhood epilepsy: a randomised controlled trial
Ketogenic Diet Therapy in Infants: Efficacy and Tolerability
Ten-year single-center experience of the ketogenic diet: factors influencing efficacy, tolerability, and compliance
Use of the modified Atkins diet for treatment of refractory childhood epilepsy: a randomized controlled trial
Seizure control and biochemical profile on the ketogenic diet in young children with refractory epilepsy--Indian experience
Efficacy of 4:1 (classic) versus 2.5:1 ketogenic ratio diets in refractory epilepsy in young children: a randomized open labeled study
A randomized trial of classical and medium-chain triglyceride ketogenic diets in the treatment of childhood epilepsy
A randomized controlled trial of the ketogenic diet in refractory childhood epilepsy
The MCT-ketogenic diet as a treatment option in refractory childhood epilepsy: A prospective study with 2-year follow-up
Efficacy of the classic ketogenic and the modified Atkins diets in refractory childhood epilepsy
Ketogenic diet efficacy in the treatment of intractable epileptic spasms
Comparison of short- versus long-term ketogenic diet for intractable infantile spasms
Effectiveness of the ketogenic diet used to treat resistant childhood epilepsy in Scandinavia
Modified Atkins diet vs classic ketogenic formula in intractable epilepsy
The effect of the classical and medium chain triglyceride ketogenic diet on vitamin and mineral levels
Long-term follow-up of the ketogenic diet for refractory epilepsy: multicenter Argentinean experience in 216 pediatric patients
Cerebral Ketones Detected by 3T MR Spectroscopy in Patients with High-Grade Glioma on an Atkins-Based Diet
The Ketogenic Diet Is an Effective Adjuvant to Radiation Therapy for theTreatment of Malignant Glioma
Enhanced immunity in a mouse model of malignant glioma is mediated by a therapeutic ketogenic diet
The ketogenic diet reverses gene expression patterns and reduces reactive oxygen species levels when used as an adjuvant therapy for glioma
Differential utilization of ketone bodies by neurons and glioma cell lines: a rationale for ketogenic diet as experimental glioma therapy
The Ketogenic Diet Alters the Hypoxic Response and Affects Expression of Proteins Associated with Angiogenesis, Invasive Potential and Vascular Permeability in a Mouse Glioma Model
A Supplemented High-Fat Low-Carbohydrate Diet for the Treatment of Glioblastoma
Ketolytic and glycolytic enzymatic expression profiles in malignant gliomas: implication for ketogenic diet therapy
The Ketogenic Diet as an Adjuvant Therapy for Brain Tumors and Other Cancers
Drug/diet synergy for managing malignant astrocytoma in mice: 2-deoxy-D-glucose and the restricted ketogenic diet
The Ketogenic Diet Does Not Affect Growth of Hedgehog Pathway Medulloblastoma in Mice
The contribution of ketone bodies to glycolytic inhibition for the treatment of adult and pediatric glioblastoma
A very low carbohydrate ketogenic diet improves glucose tolerance in ob/ob mice independently of weight loss
Effectiveness of a Very Low Calorie Ketogenic Diet on Testicular Function in Overweight/Obese Men

Effects of Very Low Calorie Ketogenic Diet on the Orexinergic System, Visceral Adipose Tissue, and ROS Production
MRI estimated changes in visceral adipose tissue and liver fat fraction in patients with obesity during a very low-calorie-ketogenic diet compared to a standard low-calorie diet
Energy expenditure and body composition changes after an isocaloric ketogenic diet in overweight and obese men
Very Low-Carbohydrate Ketogenic Diet Before Bariatric Surgery: Prospective Evaluation of a Sequential Diet
Comparing the Efficacy of Ketogenic Diet with Low-Fat Diet for Weight Loss in Obesity Patients: Evidenc-Based Case Report
Effects of a high-protein ketogenic diet on hunger, appetite, and weight loss in obese men feeding ad libitum1,
The effects of a low-carbohydrate, ketogenic diet on the polycystic ovary syndrome: A pilot study
Acid–base safety during the course of a very low-calorie-ketogenic diet
Efficacy of a 2-Month Very Low-Calorie Ketogenic Diet (VLCKD) Compared to a Standard Low-Calorie Diet in Reducing Visceral and Liver Fat Accumulation in Patients With Obesity
Effect of A Very Low-Calorie Ketogenic Diet on Food and Alcohol Cravings, Physical and Sexual Activity, Sleep Disturbances, and Quality of Life in Obese Patients
Weight loss, improved physical performance, cognitive function, eating behavior, and metabolic profile in a 12-week ketogenic diet in obese adults
Ketogenic Diet-Induced Weight Loss is Associated with an Increase in Vitamin D Levels in Obese Adults

Fine, E. J., et al. (2012). "Targeting insulin inhibition as a metabolic therapy in advanced cancer: a pilot safety and feasibility dietary trial in 10 patients." Nutrition 28(10): 1028-1035.
Foppiani, A., et al. (2021). "Isocaloric ketogenic diet in adults with high-grade gliomas: a prospective metabolic study." Nutrition and Cancer 73(6): 1004-1014.
Gano, L. B., et al. (2014). "Ketogenic diets, mitochondria, and neurological diseases." Journal of lipid research 55(11): 2211-2228.
Gupta, L., et al. (2017). "Ketogenic diet in endocrine disorders: Current perspectives." Journal of postgraduate medicine 63(4): 242.
Hader, W. J., et al. (2013). "Complications of epilepsy surgery—a systematic review of focal surgical resections and invasive EEG monitoring." Epilepsia 54(5): 840-847.
Hallböök, T., et al. (2015). "Effectiveness of the ketogenic diet used to treat resistant childhood epilepsy in Scandinavia." European Journal of Paediatric Neurology 19(1): 29-36.
Han, L., et al. (2014). "Perspective research of the influence of caloric restriction combined with psychotherapy and chemotherapy associated by hybaroxia on the prognosis of patients suffered by glioblastoma multiforme." Zhonghua yi xue za zhi 94(27): 2129-2131.
İyikesici, M. S., et al. (2017). "Efficacy of metabolically supported chemotherapy combined with ketogenic diet, hyperthermia, and hyperbaric oxygen therapy for stage IV triple-negative breast cancer." Cureus 9(7).
Jansen, N. and H. Walach (2016). "The development of tumours under a ketogenic diet in association with the novel tumour marker TKTL1: a case series in general practice." Oncology letters 11(1): 584-592.
Jóźwiak, S., et al. (2011). "Dietary treatment of epilepsy: rebirth of an ancient treatment." Neurologia i Neurochirurgia Polska 45(4): 370-378.
Kang, H. C., et al. (2011). "Comparison of short-versus long-term ketogenic diet for intractable infantile spasms." Epilepsia 52(4): 781-787.
Kargiotis, O., et al. (2010). "Effects of irradiation on tumor cell survival, invasion and angiogenesis." Journal of neuro-oncology 100(3): 323-338.
Kayyali, H. R., et al. (2014). "Ketogenic diet efficacy in the treatment of intractable epileptic spasms." Pediatric neurology 50(3): 224-227.
Kim, J. A., et al. (2016). "Efficacy of the classic ketogenic and the modified Atkins diets in refractory childhood epilepsy." Epilepsia 57(1): 51-58.
Klement, R. J. and R. A. Sweeney (2016). "Impact of a ketogenic diet intervention during radiotherapy on body composition: I. Initial clinical experience with six prospectively studied patients." BMC research notes 9(1): 1-13.
Klement, R. J., et al. (2020). "A ketogenic diet exerts beneficial effects on body composition of cancer patients during radiotherapy: an interim analysis of the KETOCOMP study." Journal of traditional and complementary medicine 10(3): 180-187.
Kossoff, E. H., et al. (2018). "Optimal clinical management of children receiving dietary therapies for epilepsy: Updated recommendations of the International Ketogenic Diet Study Group." Epilepsia open 3(2): 175-192.
Lai, J. S., et al. (2005). "Anorexia/cachexia-related quality of life for children with cancer: Testing the psychometric properties of the pediatric functional assessment of anorexia and cachexia therapy (peds-FAACT)." Cancer 104(7): 1531-1539.
Lambrechts, D. A., et al. (2015). "The MCT-ketogenic diet as a treatment option in refractory childhood epilepsy: A prospective study with 2-year follow-up." Epilepsy & Behavior 51: 261-266.
Lambrechts, D., et al. (2017). "A randomized controlled trial of the ketogenic diet in refractory childhood epilepsy." Acta Neurologica Scandinavica 135(2): 231-239.
Leung, A., et al. (2011). "The EORTC QLQ-BN20 for assessment of quality of life in patients receiving treatment or prophylaxis for brain metastases: a literature review." Expert Review of Pharmacoeconomics & Outcomes Research 11(6): 693-700.
Liu, Y.-M. and H.-S. Wang (2013). "Medium-chain triglyceride ketogenic diet, an effective treatment for drug-resistant epilepsy and a comparison with other ketogenic diets." Biomed j 36(1): 9-15.
Liu, Y.-M. C., et al. (2003). "A prospective study: growth and nutritional status of children treated with the ketogenic diet." Journal of the American Dietetic Association 103(6): 707-712.
Lockwood, G., et al. (2018). "TBIO-09. IN VITRO EVALUATION OF THE POTENTIAL OF GLUCOSE RESTRICTION AS AN ADJUVANT THERAPY FOR PAEDIATRIC BRAIN TUMOURS." Neuro-oncology 20(Suppl 2): i182.
Martin, K., et al. (2016). "Ketogenic diet and other dietary treatments for epilepsy." Cochrane Database of Systematic Reviews(2).
Martin-McGill, K. J., et al. (2018). "The modified ketogenic diet in adults with glioblastoma: an evaluation of feasibility and deliverability within the national health service." Nutrition and cancer 70(4): 643-649.
Martin-McGill, K. J., et al. (2020). "Ketogenic diets as an adjuvant therapy for glioblastoma (KEATING): a randomized, mixed methods, feasibility study." Journal of neuro-oncology 147(1): 213-227.
McGirt, M. J., et al. (2008). "Persistent outpatient hyperglycemia is independently associated with decreased survival after primary resection of malignant brain astrocytomas." Neurosurgery 63(2): 286-291.
Milder, J. and M. Patel (2012). "Modulation of oxidative stress and mitochondrial function by the ketogenic diet." Epilepsy research 100(3): 295-303.
Moher, D., et al. (2009). "Preferred reporting items for systematic reviews and meta-analyses: the PRISMA statement." PLoS Med 6(7): e1000097.
Neal, E. G., et al. (2008). "Growth of children on classical and medium-chain triglyceride ketogenic diets." Pediatrics 122(2): e334-e340.
Neal, E. G., et al. (2008). "The ketogenic diet for the treatment of childhood epilepsy: a randomised controlled trial." The Lancet Neurology 7(6): 500-506.
Neal, E. G., et al. (2009). "A randomized trial of classical and medium-chain triglyceride ketogenic diets in the treatment of childhood epilepsy." Epilepsia 50(5): 1109-1117.
Nebeling, L. C., et al. (1995). "Effects of a ketogenic diet on tumor metabolism and nutritional status in pediatric oncology patients: two case reports." Journal of the American College of Nutrition 14(2): 202-208.
Oliveira, C. L., et al. (2018). "A nutritional perspective of ketogenic diet in cancer: a narrative review." Journal of the Academy of Nutrition and Dietetics 118(4): 668-688.
Panhans, C. M., et al. (2020). "Exploring the feasibility and effects of a ketogenic diet in patients with CNS malignancies: a retrospective case series." Frontiers in neuroscience: 390.
Paoli, A., et al. (2013). "Beyond weight loss: a review of the therapeutic uses of very-low-carbohydrate (ketogenic) diets." European journal of clinical nutrition 67(8): 789-796.
Poff, A. M., et al. (2015). "Non-toxic metabolic management of metastatic cancer in VM mice: novel combination of ketogenic diet, ketone supplementation, and hyperbaric oxygen therapy." PloS one 10(6): e0127407.
Poff, A., et al. (2014). "Ketone supplementation decreases tumor cell viability and prolongs survival of mice with metastatic cancer." International journal of cancer 135(7): 1711-1720.
Raju, K. V., et al. (2011). "Efficacy of 4: 1 (classic) versus 2.5: 1 ketogenic ratio diets in refractory epilepsy in young children: a randomized open labeled study." Epilepsy research 96(1-2): 96-100.
Rieger, J., et al. (2014). "ERGO: A pilot study of ketogenic diet in recurrent glioblastoma Erratum in/ijo/45/6/2605." International journal of oncology 44(6): 1843-1852.
Ruskin, D. N. and S. A. Masino (2012). "The nervous system and metabolic dysregulation: emerging evidence converges on ketogenic diet therapy." Frontiers in neuroscience 6: 33.
Salek, T., et al. (2017). "Topiramate induced metabolic acidosis and kidney stones–a case study." Biochemia Medica 27(2): 404-410.

Santos, J. G., et al. (2018). "Efficacy of a ketogenic diet with concomitant intranasal perillyl alcohol as a novel strategy for the therapy of recurrent glioblastoma." Oncology Letters 15(1): 1263-1270.

Schmidt, M., et al. (2011). "Effects of a ketogenic diet on the quality of life in 16 patients with advanced cancer: A pilot trial." Nutrition & metabolism 8(1): 1-13.

Schreck, K. C., et al. (2019). "Effect of ketogenic diets on leukocyte counts in patients with epilepsy." Nutritional neuroscience 22(7): 522-527.

Schwartz, K., et al. (2015). "Treatment of glioma patients with ketogenic diets: report of two cases treated with an IRB-approved energy-restricted ketogenic diet protocol and review of the literature." Cancer & metabolism 3(1): 1-10.

Servaes, P., et al. (2002). "Fatigue in cancer patients during and after treatment: prevalence, correlates and interventions." European journal of cancer 38(1): 27-43.

Seyfried, B. T. N., et al. (2009). "Targeting energy metabolism in brain cancer through calorie restriction and the ketogenic diet." Journal of cancer research and therapeutics 5(9): 7.

Seyfried, T. N. and L. M. Shelton (2010). "Cancer as a metabolic disease." Nutrition & metabolism 7(1): 1-22.

Seyfried, T. N., et al. (2012). "Is the restricted ketogenic diet a viable alternative to the standard of care for managing malignant brain cancer?" Epilepsy research 100(3): 310-326.

Sharma, S., et al. (2009). "Seizure control and biochemical profile on the ketogenic diet in young children with refractory epilepsy—Indian experience." Seizure 18(6): 446-449.

Sharma, S., et al. (2013). "Use of the modified A tkins diet for treatment of refractory childhood epilepsy: A randomized controlled trial." Epilepsia 54(3): 481-486.

Shelton, L. M., et al. (2010). "Calorie restriction as an anti-invasive therapy for malignant brain cancer in the VM mouse." ASN neuro 2(3): AN20100002.

Shen, V., et al. (2016). "HG-32USE OF KETOGENIC DIET AS A COMPLIMENTARY METABOLIC THERAPY DURING CHEMO-RADIATION THERAPY IN A 7 YEAR OLD FEMALE WITH GLIOBLASTOMA." Neuro-Oncology 18(Suppl 3): iii54.

Shinojima, N., et al. (2017). "P18. 11 The effect of ketogenic diet on survival and quality of life in patients with malignant brain tumors in palliative care." Neuro-Oncology 19(Suppl 3): iii123.

Shinojima, N., et al. (2017). "P19. 05 Safety and tumor inhibitory effect of ketogenic diet for pediatric patients with malignant brain tumors." Neuro-Oncology 19(suppl_3): iii126-iii126.

Storer, L. C., et al. (2018). "MBRS-20. KETOLYTIC AND GLYCOLYTIC ENZYMATIC EXPRESSION IN PAEDIATRIC MEDULLOBLASTOMAS: IMPLICATION FOR KETOGENIC DIET THERAPY." Neuro-Oncology 20(Suppl 2): i132.

Suzuki, H., et al. (2013). "Cancer cachexia—pathophysiology and management." Journal of gastroenterology 48(5): 574-594.

Szerlip, N., et al. (2011). "Factors impacting volumetric white matter changes following whole brain radiation therapy." Journal of neuro-oncology 103(1): 111-119.

Tan-Shalaby, J. L., et al. (2016). "Modified Atkins diet in advanced malignancies-final results of a safety and feasibility trial within the Veterans Affairs Pittsburgh Healthcare System." Nutrition & metabolism 13(1): 1-12.

van der Louw, E. J., et al. (2019). "Ketogenic diet treatment in recurrent diffuse intrinsic pontine glioma in children: A safety and feasibility study." Pediatric Blood & Cancer 66(3): e27561.

van der Louw, E., et al. (2016). "Ketogenic diet guidelines for infants with refractory epilepsy." European Journal of Paediatric Neurology 20(6): 798-809.

Vander Heiden, M. G., et al. (2009). "Understanding the Warburg effect: the metabolic requirements of cell proliferation." science 324(5930): 1029-1033.

Vining, E. P., et al. (2002). "Growth of children on the ketogenic diet." Developmental medicine and child neurology 44(12): 796-802.

Voss, M., et al. (2020). "ERGO2: a prospective, randomized trial of calorie-restricted ketogenic diet and fasting in addition to reirradiation for malignant glioma." International Journal of Radiation Oncology* Biology* Physics 108(4): 987-995.

Wang, X. S., et al. (2014). "Prevalence and characteristics of moderate to severe fatigue: a multicenter study in cancer patients and survivors." Cancer 120(3): 425-432.

Warburg, O., et al. (1927). "The metabolism of tumors in the body." The Journal of general physiology 8(6): 519.

Weber, D. D., et al. (2020). "Ketogenic diet in the treatment of cancer–where do we stand?" Molecular metabolism 33: 102-121.

Wells, G. A., et al. (2000). The Newcastle-Ottawa Scale (NOS) for assessing the quality of nonrandomised studies in meta-analyses, Oxford.

Wibisono, C., et al. (2015). "Ten-year single-center experience of the ketogenic diet: factors influencing efficacy, tolerability, and compliance." The Journal of pediatrics 166(4): 1030-1036. e1031.

Winter, S. F., et al. (2017). "Role of ketogenic metabolic therapy in malignant glioma: a systematic review." Critical reviews in oncology/hematology 112: 41-58.

Wirrell, E., et al. (2018). "Ketogenic diet therapy in infants: efficacy and tolerability." Pediatric Neurology 82: 13-18.

Woodhouse, C., et al. (2019). "Feasibility of a modified Atkins diet in glioma patients during radiation and its effect on radiation sensitization." Current Oncology 26(4): 433-438.

Woolf, E. C. and A. C. Scheck (2015). "The ketogenic diet for the treatment of malignant glioma." Journal of lipid research 56(1): 5-10.

Woolf, E. C., et al. (2016). "Tumor metabolism, the ketogenic diet and β-hydroxybutyrate: novel approaches to adjuvant brain tumor therapy." Frontiers in molecular neuroscience 9: 122.

Zuccoli, G., et al. (2010). "Metabolic management of glioblastoma multiforme using standard therapy together with a restricted ketogenic diet: Case Report." Nutrition & metabolism 7(1): 1-7.

rate of three capping materials: Nanohydroxyapatite (NHA), Mineral Trioxide Aggregate (MTA), and Formocresol (FC) in pulpotomy of primary molars. Methods: A clinical trial was carried out on healthy, four to eight years old children, with 72- second primary molars indicated for pulpot

t. Objectives: To describe the indications, sonographic findings, and contribution of orbito-ocular ultrasonography to the management of orbito-ocular diseases in the University College Hospital, Ibadan. Materials and Methods: A retrospective review of B-mode ocular ultrasound finding

y how far the scope can be advanced in the tract and are also invasive. VCE allows gastroenterologists to investigate GI tract abnormalities in detail with visualization of all parts of the GI tract. It captures continuous real time images as it is propelled in the GI tract by gut motility. Even th

ections (UTI) has become increasingly more common, limiting treatment options among children presenting with febrile UTI. We investigated the burden and correlates of ESBL producing Enterobacteriaceae associated UTI among children and antibacterial resistance pattern. Methods: [illegible]

d other less common clinical manifestations such as abdominal pain or arthralgia. The pathogenesis is unknown, and autoimmune origins, chromosomal alterations, and other causes have been proposed. The nosology of this condition is confusing in the literature, and the precise diagno

systemic/local thrombolytic therapy with tissue plasminogen activator, or surgical thrombectomy. (Level of Difficulty: Advanced.).

[illegible] (PROVE/CHD) Registry in Isfahan. Methods: All patients with CHD and associated defects diagnosed by pediatric cardiologists were assessed via echocardiography for inclusion in the present study between late 2016 and August 2019. The participants' sociodemographic characteristics,

mmunication. We hypothesized that compared to usual care, legacy-making would improve quality of parent-child communication.Between 2015 and 2018, Facebook advertisements were used to recruit families of children (ages 7-17) with relapsed/refractory cancer. Parent-child dyad

ns and (2) provide an example of how one cooperative group is implementing these innovative efforts to partner with programs to integrate pediatrics on an expanding scale. Details are described for how pediatric studies can benefit from cooperative group infrastructure and expertise.

r hospital, was used to calculate the BSA in infants and children aged 0-18 years using BW and height data from 27,440 hospital visits by 20,635 patients over one year. Methods: The best fit of three nonlinear regression equations (third-order polynomial, Meeh-type, and modified Boyd

nd the WHO's database of publications on novel coronavirus. We included English language studies that had described original demographic and clinical characteristics of children diagnosed with COVID-19 and reported on the presence or absence of gastrointestinal symptoms. Meta-ar

asidiobolomycosis (GIB) a diagnostic challenge. Methods: We are reporting the largest series of pediatric GIB, from Saudi Arabia. 12 patients were diagnosed between January 2012 and December 2019, between the ages of 16 months and 8 years. Results: The most common symptoms w

ased cross-sectional study on all pediatric residents in the largest pediatric training center in Saudi Arabia. The survey had six sections: a) Demographics and self-assessment of religiosity, b) Sources of ethics education, c) Degree of confidence in dealing with ethical challenges in clinical p

ause they share the same symptoms, particularly coughing and shortness of breath; physical findings, such as wheezing; radiological findings, such as hyperinflation on chest X-ray; or even responses to asthma therapies, as in some patients with bronchiolitis. When treating the younger

erapies, clinicians should be cognizant of the potential risk of contact dermatitis in patients with leg ulcers. Contact dermatitis (both allergic and irritant) to wound products may present as maceration, pain, and overall impaired wound healing. Herein, we review the literature on contact

ribing patterns or practices of individual practitioners and, therefore, allows off-label use. The main objective of this study is to evaluate off-label prescribing among the pediatric population in the Kingdom of Saudi Arabia (KSA). Method: This is a retrospective, simple random selection of

4-year old Nigerian girl with clinical and histopathologic features of definitive juvenile dermatomyositis based on EULAR/ACR classification criteria but probable Juvenile dermatomyositis according to Bohan and Peter criteria. The patient had normal aspartate and alanine aminotransfera

:h of the muscle tendon was shorter than usual and that the muscle insertion was displaced inferiorly, this prompt further exploration. It was found that the medial rectus muscle had an anomaly were it was seperated into two (superior and inferior) compartments which were 5 mm apa

nance is impacted by unpredictable disease symptoms. AYA with hemoglobinopathies may require academic accommodations to promote postsecondary success; however, accessing appropriate supports can be complicated. Methods: Given these complexities, a multidisciplinary team

e patient.

:reened. Children satisfying inclusion criteria and their parents agreed to participate were included. Questionnaires regarding parents' knowledge and perception about halitosis in their children were filled by attending parents. Then, the assessment of halitosis in the participating childre

dy was to assess the prevalence of HI among patients with CH and to examine factors potentially predictive of HI including severity of CH, etiology of CH, and timing of treatment initiation. Methods: Audiometry was undertaken prospectively in 66 patients aged 3-21 years diagnosed with

t a bonding agent and to characterize the enamel-sealant interface using confocal laser scanning microscopy (CLSM). The null hypothesis was that the use of a bonding agent before fissure sealant application would not change the microtensile strength or the enamel-sealant interface. M

objective of this study was to assess the readiness to implement a national evidence-based CM prevention (CMP) program in Bahrain. Methods: The cross-sectional study was conducted with 45 key informants who had influence and decision-making power over CMP. Results: The overal

ers and sizing equipment based on a two-step optimization process to meet the population's needs. The models consider physicians' propensity for working on a metropolis and the patients' choice on moving the least from their municipalities, therefore, conflicting decisions. The models

ssociated risk factors. Methods: A modified UNICEF Multiple Indicator Cluster Survey (MICS) was used to analyze all deaths among children under the age of 18 which occurred at the King Abdullah Specialized Children's Hospital (KASCH) between 2010 and 2016. Results: After reviewing a

cted at various schools. To achieve the aim of the study, we selected a random sample of 850 school children aged 6-15 years. The examination was based on the RESC protocol and included full visual assessment. Furthermore, a questionnaire was sent to the parents of the participants

aximize the efficiency of treatment. This study aimed to compare the static friction of orthodontic arch wire materials, including a newly introduced low-friction TMA, conventional TMA, and stainless steel arch wires, using an Instron universal testing machine and to evaluate their surfac

aims to identify the barriers of enteral nutrition in PICU. Setting and Design: A cross-sectional study of the results of a 25-item questionnaire-based survey distributed during the Annual International Critical Care Conference by the Saudi Critical Care Society. Methods and material: A 7-p

oneal dialysis (PD)-associated peritonitis in children. Methods: Data from medical records of children who underwent PD between 2007 and 2018 in King Fahad Medical City were retrospectively collected. All children aged <14 years undergoing chronic PD were included. The demographi

cantly inhibits hepatic steatosis and lowers serum triglycerides, playing a protective role against the progression of non-alcoholic fatty liver disease (NAFLD). However, the direct impact of BMP4 on hepatic glucose metabolism is poorly understood. Here, we investigated the regulatory ro

to detection of Mycobacterium tuberculosis (MTB) in neutralized and non-neutralized gastric aspirate samples and report the drug resistance. MATERIALS AND METHODS: A total of 336 neutralized and non-neutralized gastric aspirate samples were simultaneously cultured by both LJ cu

making to parents, (2) parent satisfaction, and (3) parent utilization of the project. Design: Eko CORE (Eko Devices, Inc., Berkeley, CA), a digital stethoscope that generates a phonocardiogram, a graphical representation of S1 and S2 heart sounds, was used to record children's heartbeats

ban communities of Zagreb and Zagreb County by localization, type and frequency of treatment methods used according to age and fracture mechanism. The research included 103 children aged up to 18 years, treated for femur fractures at the Zagreb University Hospital Centre and Za

uently developed severe hypertension and respiratory failure. Physical examination and laboratory findings did not indicate a secondary cause of hypertension. Her respiratory failure was treated with non-invasive ventilation and hypertension controlled with enalapril, furosemide and s

recautions. OBJECTIVES: The aim of this study was to assess and improve the compliance of healthcare providers with protocols based on best available evidence for prevention of sharps injuries in medical, surgical and pediatric wards of the hospital. METHODS: A three-phase clinical au

that developed after heart surgery in a 9-year-old boy. Ventricular septal defect, aortic and mitral valves repair, and pacemaker implant procedures were noted in his medical records. Preganglionic HS was diagnosed with bilateral unresponsiveness to a 0.1% adrenaline and positive resu

the Adolescent Brain Cognitive Development (ABCD), we analyzed the structural Magnetic Resonance Imaging (sMRI) data of 11141 9-10 years old children. The main outcome was the left thalamus volume. The independent variable was parental education. Age, sex, ethnicity, family ma

reduce the need for routine biopsies of low-risk gastric polyps. Methods: Pairs of NBI and white-light images were collected from 73 gastric polyps for which concurrent histopathologic diagnosis was available. A diagnostic accuracy cohort study was performed. Two blinded endoscopist

ients to express their concerns. The factors, which prevent nurses spending time with patients, are still unclear. METHOD: Data were collected using semi-structured interviews with thirty-five participants, including the nurses and physicians from educational hospitals of Tehran. The ana

an energy source that is more efficient than glucose, resulting in beneficial downstream metabolic changes, such as increasing adenosine levels, which might have effects on seizure control. However, some studies have challenged the central role of ketones because medium-chain fatty

in the pancreas, and there is any relationship between blood glucose levels. Methods: This examination was done on 32 chosen rats with their glucose levels. Animals are divided into owned blood glucose levels. If mean glucose levels were equal to 105 +/- 10 mg/dL accepted as euglyc

uptions, fever, and organ involvement (including liver, kidney, hematological system, etc.). DHS can develop several weeks to as late as 6 months after treatment initiation with dapsone. Here, we report a case of DHS and leukemoid reaction with coexisting hepatitis E in a 10-year-old girl

an untrained CPR Coach into resuscitation teams during simulated pediatric cardiac arrest. Simulation events were randomized to two arms: control (no CPR Coach) or intervention (CPR Coach). Simulations were run by pediatric intensive care unit (PICU) providers and video recorded. S

ac surgery, classified according to Society of Thoracic Surgery-European Association of Cardiothoracic Surgery (STS-EACTS) risk category. We had 25,052 (52%) patients in 53 mixed units (mortality rate, 2.99%), and 22,762 (48%) patients in 19 dedicated CICUs (mortality rate, 2.62%). Ther

Our patient's case is unique as her triglyceride levels were markedly higher than those in other reports and required a longer duration of time to resolve. We review the literature on the concomitant presentation of DKA and hypertriglyceridemia, as well as its pathophysiology and treatr

ion disparities in sexual health among women, but research on sexual health communication among SMW is sparse. Methods: In-depth interviews conducted in 2016-2017 investigated sexual health communication in a sample of 29 racially/ethnically diverse cisgender women and non-

f the anterior tricuspid leaflet from the tricuspid annulus and the crucial role of multimodality imaging in its diagnosis and treatment. (Level of Difficulty: Intermediate.).

after pediatric cardiac arrest, including return of spontaneous circulation (ROSC), survival to hospital admission (HA) and survival to hospital discharge (HD). Three logistic regression models were performed using JMP 14.1 Pro for Windows, each with the following nine predictors: age, se

l drive health disparities. Pediatric settings offer an opportune context to address ACEs and improve health equity, and to link families to the necessary resources to promote resilience. Wellness navigators (WNs), who can reflect patients' cultural, linguistic, and other shared characterist

tients with acute leukemia or chronic myeloid leukemia who underwent haploidentical HSCT during 2014-2019 was performed. The graft versus host disease (GVHD) prophylaxis was post-transplant Cyclophosphamide with Mycophenolate-mofetil and Cyclosporine. All patients were trar

: "Pediatric hydrocephalus: systematic literature review and evidence-based guidelines" and determined the need for an update to the original guideline based on new available evidence. OBJECTIVE: To perform an update to include the current medical literature for the "Pediatric hydro

ig-term venous access device insertion in pediatric patients compared to the traditional approach. Methods: This single-institution retrospective cohort study included 169 children who had port-A-catheters between May 2016 and Oct 2019. The patients were divided into two groups; gı

pproach -: Integrating a crossed-disciplinary approach, the authors conducted a survey at the 2018 Minnesota State Fair querying the perception of risk and preferences of current and future parents associated with pediatric NIBS research. The survey consisted of 28 closed-text questior

id fourth-year students completing a pediatric nursing internship. Factor analysis, Cronbach's alpha, item-total score analysis, and known-groups comparison were used to assess the research data. In total, 16 items were eliminated from the scale on the basis of experts' recommendatior

n delayed. Invasive treatment is controversial, particularly in pediatric patients. However, it is indicated in cases of gross hematuria associated with anemia, renal function impairment, severe pelvic pain, or ineffective conservative treatment. We report the case of a 12-year-old boy pres

:h DM (main group) and 32 were DM-free (control group). The groups were formed according to the "case-control" principle. During hospitalization, the dynamics of clinical, glycemic, and coagulation parameters, markers of systemic inflammation, as well as kidney and liver functions we

re not available. The aim of this study was to formulate and evaluate the stability of a ready-to-use rectal pentobarbital gel. Methods: The formulation consisted of a hydrated gel containing 25 mg/mL of pentobarbital sodium, packaged in 10-mL amber glass bottles and stored at either 2

cted from native sources. LTAs are built up from glycerol phosphate (GroP) repeating units and they can be substituted at the C-2-OH with carbohydrate appendages or d-alanine residues. The substitution pattern, but also the absolute chirality of the GroP residues can impact the intera

duates' neurodevelopmental outcomes, skin-to-skin care of intubated infants is encouraged, while sedation and restraints to prevent UE are strongly discouraged. This project aimed to decrease the UE rate from 1.85 to 1.5 per 100 endotracheal tube (ETT) days. Methods: The project oc

sive symptoms. Objective To describe the profile of the otorhinolaryngological clinical examination and audiology tests of patients with MPS disease. Methods The present study is a case series. The evaluation was performed, initially, in 24 patients with MPS types I, II, IIIA, IV and VI. Res

ınd lingual surfaces of 40 premolar teeth. The "etch-and-rinse" technique of a universal bond adhesive system (Single Bond Universal Adhesive) was used on buccal preparations, while the "self-etch" protocol was used on the lingual surfaces. Two RBCs, one bulk fill (Filtek Bulk Fill [FBF]) a

ent. With residual complement deposition, it is difficult to determine whether there is a reduced complement-mediated endothelial cell injury. We validated that myeloperoxidase (MPO) immunohistochemical staining identified glomerular endothelial cell injury in crescentic glomerulon

im of this study was to assess the long-term impact on lung function and respiratory symptoms in children hospitalised due to NP. METHODS: We analysed outcomes in children given a diagnosis of NP between January 2003 and April 2016. We selected patients aged more than 4 years c

ıroblems. His mother also possesses the same Xp22.31 deletion. The patient presented with status epilepticus and stroke, followed by severe brain atrophy and developmental regression. His unusual clinical and cytogenetic findings apparently have not been reported with either KS or X

time of the study (10 years old or greater with diabetes duration of 5 years or more) would report multiple barriers to screening and that targeted barriers and subpopulations could be identified to improve access to care. 271 youth aged 10 to 26 years with T1D of at least 5 years durati

kin findings are often present in these patients, and when initially compared with Kawasaki disease, they likely represent distinct phenomena and overall remain poorly characterized. In this retrospective review of 34 case reports and series, we identified cutaneous manifestations docur

ı adherence to Paediatric Advanced Life Support (PALS) guidelines during simulated paediatric cardiac arrest. Methods: This was a secondary analysis of data collected from a multicentre randomized controlled trial assessing the quality of CPR in teams with and without a CPR Coach. For

:e the tissue oxygenation and factors which may affect it by bilateral cerebral and somatic NIRS levels during cardiopulmonary bypass and to compare the NIRS values of cyanotic and acyanotic patient groups. Material and methods: Two groups of patients with cyanotic and acyanotic co

d central vision. Prior to presenting to the ED the patient was evaluated by his optometrist and his eye exam tested 20/400 bilaterally. His previous year's eye exam was normal, 20/25 bilaterally. Results: In the ED, the patient had an MRI which showed a normal appearing optic nerve, ch

:onducted in two tertiary and one urban district hospital in Kigali, Rwanda. Participants were neonates admitted in neonatology who received a course of antibiotics during their admission. Data collected included risk factors for neonatal sepsis, clinical signs, symptoms, investigations for

formed exome sequencing in 289 childhood cancer survivors at least 3 years from anthracycline exposure. In a nested case-control design, 183 case patients with reduced left ventricular ejection fraction despite low-dose doxorubicin (</=250 mg/m(2)), and 106 control patients with pre:

ltrasound to confirm endotracheal tube (ETT) position in patients. A prospective study was performed on intubated patients with cuffed ETTs. The accuracy of ultrasound to confirm correct ETT placement in 92 patients was 97.8%. Sensitivity, positive predictive value, and agreement of !

dults. The present study was done to analyze the clinical profile and outcome of adult ES. Aims The aim was to study the clinical and pathological treatment and outcomes in adult ES. Subjects and Methods Between 2010 and 2017, a total of 73 ES patients with age more than 18 years w

spective study, we identified six patients from a large cohort of 220 patients with HH who were treated with the ketogenic diet (KD). Four patients had a 50-90% reduction in multiple seizure types (including gelastic, partial-onset and atonic seizures), and two individuals failed to respond

lucted with the objectives of determining clinico-bacteriological profile and antibiotic susceptibility among isolated bacteria in a neonatal intensive care unit. METHODS: A descriptive cross-sectional study was conducted from January 1, 2017, to December 31, 2019, in the neonatal inten

ous access to genetic testing. More than 1586 genetic tests were performed in 1019 individuals (349 index cases, 670 relatives). Most frequently performed tests were exome/genome sequencing (ES/GS, 284/78 index cases) and specific gene panels (55 index cases). In 61.3% of the patie

being later recognized as band-form heterotopia. This type of heterotopia may be overlooked easily due to its symmetrical distribution and mild degree of cortical convolutional anomaly. As it is often associated with intractable seizures and psychomotor retardation, the prognosis is poo

: review of children aged </= 15 years diagnosed with precursor-B ALL from 2014 to 2019 was performed. The protocol used for risk stratification and treatment was based on a UKALL-2003 backbone. All patients received one week of prephase therapy comprised of intravenous dexame

ers Ther 35(4).
: search for the new pharmacological substances with anticancer activity against drug-resistant tumors is of utmost importance. In the present study we primarily investigated the correlation between the expression of TrkA and p75 receptors with the nerve growth factor (NGF) and cispl

ir experiences and perspectives on this topic is scarce. In this study, we build on literature related to management of suicidality on campus, which is predominantly focused on campus counseling professionals. Fifteen semi-structured qualitative interviews were conducted with student a

wth of hamartomatous lesions in multiple organs due to insufficient suppression of the mTORC1 pathway. A minority of patients with TSC who develop epilepsy which is intractable to standard anticonvulsant medical and/or surgical treatments are treated with the ketogenic diet. To pro

erns over possible harmful insertional mutagenesis seen in other cell types, particularly hepatocytes, raised a question about AAV safety. In this study, we evaluate the long-term persistence of this vector in mouse lungs and any possible harmful integration of these vectors into the host

essed nasal bridge, and widely spaced eyes. Many affected individuals are microcephalic. A spectrum of brain abnormalities are seen on magnetic resonance imaging, including cerebral atrophy, enlarged ventricles and generous extra-axial cerebral spinal fluid (CSF) spaces, delayed myeli

rrest out-of-hospital randomized trial involving children who remained unconscious and intubated after OHCA (n = 237). Associations between MODS and 12-month outcomes were assessed using multivariable logistic regression. Non-neurologic MODS was present in 95% of patients ar

ctory disease. Therefore, there is an urgent need for novel therapies. Molecular genetic analyses and sequencing studies have led to the identification of recurrent T-ALL genetic drivers. This review summarizes the main genetic drivers and targetable lesions of T-ALL and gives a compreh

:h creates insecurity in parents. Significantly, Internet searches have been increased to address possible health concerns, questioning the quality of websites about tonsillectomy. Objective To evaluate the readability, reliability, and comprehensiveness of the Italian websites dedicated to

vas to quantify reported failure rates of allograft versus autograft ACLR in patients </=19 years of age with >/=2 years of follow-up. We hypothesized that there would be higher rates of failure for allograft compared with autograft ACLR in this population. Methods: PubMed/MEDLINE an

as implemented as the central hub for rounding at the bedside by the attending physicians, while other members of the multidisciplinary and multispecialty team joined rounds from other locations with maintaining appropriate social distance. Fifty-eight staff members who participated

ory deterioration. The objective of this study was to evaluate patient characteristics associated with HFNC failure in bronchiolitis. Methods: A retrospective review of patients aged 0 to 24 months, with bronchiolitis who received HFNC within a single tertiary paediatric intensive care unit,

ı tumor in children, arises as a consequence of activating mutations in the Hedgehog (HH) pathway, which has been shown to promote aerobic glycolysis. Therefore, we hypothesized that a low carbohydrate, high fat ketogenic diet would suppress tumor growth in a genetically engineer

raining clinicians to use the Partners Serious Illness Conversation Guide, a structured guide to teach basic palliative care communication skills, we propose a set of best practices to help others teach use of a communication guide at their institution, including fostering a safe learning envi

eenage behaviors or boys. We use a difference-in-difference-in-differences framework to identify gender-specific effects of welfare reform on skipping school, fighting, damaging property, stealing, hurting others, smoking, alcohol, marijuana, and other illicit drugs. Welfare reform led to

were compared: 2008 to 2014: premerge, 2014 to 2017: initial time period postmerge, and 2018 to 2019: established merge. Survival to hospital discharge (and with good neurological outcome) was of 68% (61%), 46% (36%), and 79% (71%), respectively, for the three time periods. Merg

t diagnosed with systemic scleroderma where photobiomodulation therapy was used to treat xerostomia associated with hyposalivation. She attended a pediatric clinic and presented with dry and rigid facial skin, trismus, xerostomia, malocclusion, and difficulty swallowing. Stimulated s

New and efficient methods for facile synthesis of insulin derivatives may lead to new discovery of therapeutic insulin. Herein, we report a new method using sortase A (SrtA)-mediated ligation for the synthesis of insulin derivatives with high efficiency and functional group tolerance in th

kes it technically challenging to purify sarcoma-derived EVs. In this study, a specific purification system is developed for Ewing sarcoma (ES)-derived EVs by coupling covalent chemistry-mediated EV capture/ release within a nanostructure-embedded microchip. The purification platform-

, as well as maternal cardiac, obstetric, and neonatal outcomes. (Level of Difficulty: Intermediate.).

cularly for pediatric and neonatal populations. Methods: This paper describes innovative strategies implemented in a large Level IV NICU to promote the core concepts of PFCC that ensured parent-infant bonding while limiting exposure to a pandemic infection, such as COVID-19. Discus

ıvironment for cardiovascular prevention, where cardiovascular guidelines have had limited implementation. Despite the impact that unhealthy lifestyles at the workplace may have on the cardiovascular health of U.S. workers, there is currently no policy in place aimed at improving this

ıtcome of relapse are limited. In the Netherlands, patients with aHUS are treated with a restrictive eculizumab regime and are included in a national observational study (CUREiHUS, Dutch Trial Register NTR5988/NL5833). Methods: For this interim safety analysis, we evaluated the outco

measures of gray matter and white matter. Here we used deformation-based and persistent homology approaches to characterize the three-dimensional topology of brain structure asymmetries within language-related areas that were defined in functional neuroimaging experiments. F

eatment have not established a drop in the aforementioned morbidities. Those trials did not physiologically categorise PDA severity. Incorporating the specific physiological features of a haemodynamic significant PDA may evolve our understanding of this phenomenon, allowing accurate

e in adipose tissue. These compounds can then be excreted into maternal milk resulting in infant exposure. Objective: To determine the level of PCBs in milk from mothers with pre-pregnancy overweight and obese (OW/OB) versus normal weight status (NW) and evaluate the associatio

cardiomyopathy. Methodology: This study was designed as case-control study. A total of 55 children with type 1 diabetes mellitus (group/G1) and 55 healthy controls (G2) were subjected to echocardiography including 3D-Speckle Tracking Echocardiography and tissue Doppler imaging fo

s: The aim of this study was to evaluate the spirometric measurements in standing and sitting positions in a group of Egyptian asthmatic children with different body weights. Methods: Sixty patients were included. They were stable asthmatics and were following up in the allergy clinic.

ı KD and gut microbiota (GM) is not yet been deeply understood. Herein, we investigated the anti-seizure effect by administering KD and a lactic acid bacteria (LAB) in murine model of chemically induced seizures. We hypothesized that a single Lactobacillus fermentum MSK 408 (MSK 40

ach for children whose epilepsy has failed to improve with first-line medical therapy is uncertain. METHODS: A review of MEDLINE from 1990 to 2015 was conducted. A cost-utility analysis, from a third-party payer perspective, was performed for children with drug-resistant epilepsy that

p13.3 microduplication by BACs-on-Beads (BoBs) assay and further confirmed by fluorescence in situ hybridization (FISH). Our patients (Patient 1: 4-year-old male; Patient 2: 2-year-old male) presented with developmental delay, intellectual disability, and dysmorphic facial features. Wh

ale who was admitted to the pediatric infectious ward due to a fever of unknown origin in January 2020. She was a known case of Marfan syndrome with a family history of this syndrome in her mother. The species was identified using (PCR) and the antifungal susceptibility test was perf

ts of <24 weeks, to harmonize and improve quality of care. Methods: We created a multidisciplinary working group with experience in caring for preterm infants, searched the literature from 2000 to 2019 to identify best practices for the care of extremely preterm infants and consulted

ergency surgery. Although medication-related etiologies formerly predominated, the elimination of halothane from anesthetic care has resulted in a shift in etiology to hemodynamic events related to blood loss or hyperkalemia associated with the rapid administration of blood products

)F) and its intersection with novel computational and data science approaches to discovering biomarkers of functional and prognostic importance. Efforts in translational medicine that seek to address the clinical challenges associated with cardiovascular diseases using personalized and

ffectiveness of existing infection control measures. Patients and methods: We prospectively included all adult and pediatric patients admitted to Necker hospital (Paris) between October 2018 and February 2019 with a diagnosis of RSV-associated ALRI. We compared characteristics of AL

with Helicobacter pylori (H. pylori) is often associated with the development of gastric adenocarcinoma. Infection with H. pylori induces a series of events that lead to non-atrophic gastritis (NAG), multifocal atrophic gastritis (MAG), intestinal metaplasia (IM), dysplasia, and cancer. All th

ork (GAN) has continued to study these conditions. Objective: To estimate the prevalence of AR and ARC in children and adolescents in Mexico and to assess their association with different risk factors. Methods: GAN Phase I is a cross-sectional, multicentre survey carried out in 15 centres

ns of KD could be associated with its development. An 11-month-old male was diagnosed with incomplete KD. No coronary abnormalities were detected. He was treated with intravenous immunoglobulin (IVIG) and aspirin. Four weeks later, he developed fever, otitis media, bullous phar

in children and adolescents. Methods: In June 2019, we searched the Cochrane Database of Systematic Reviews for eligible reviews. The primary outcomes were change in adiposity (body mass and body mass index [BMI] z-score) and adverse events. Two reviewers screened studies and

ic tuberculosis. Methods: Our search included studies from 2011 through 2019, and specific search terms were used to retrieve articles from Pubmed, EMBASE, BIOSIS, ClinicalTrials.gov, and Google Scholar. Risk of bias was assessed using the QUADAS 2 tool. The protocol was registered

arges the left atrium and avoids circuitous pathways or tension on the scimitar pulmonary vein. Methods: Between April 2011 and November 2018, 22 patients underwent scimitar vein surgery; 11 had baffling or reimplantation and 11 only had the new operation that included resection

on. OBJECTIVE: To examine the use of clinical, surrogate and patient-centered outcomes in studies on ARPKD with special attention to core outcomes of the Standardized Outcomes in NephroloGy project for children with chronic kidney disease (SONG-Kids). DATA SOURCES AND STUDY E

m-based medications have been proposed as risk factors. Neurodevelopmental outcomes have been reported among these studies; however, few include data about the individuals' early neurodevelopmental profile, a factor that may contribute significantly to these outcomes. Further,

at arise when treating patients with psoriasis, especially those age <12years, are summarized, as well as the limited available treatment options for treating pediatric patients with psoriasis and the evidence supporting each of them. Methods: Recently published guidelines by the Americ

ary hypertension should be always considered and excluded. We present the case of an adolescent with secondary hypertension and a challenging diagnosis associated with coarctation of aorta and Turner Mosaicism.

Methods: This is a retrospective chart review in a tertiary care hospital from January 2015 to March 2020. CD was confirmed by elevated N- acetyl aspartate (NAA) levels in urinary gas chromatography and mass spectrometry (GCMS)/increased NAA peak in magnetic resonance spectrosc

tive review of the charts of children with CACH was performed from January 2014 to March 2020 at tertiary care center from Southern India. Diagnosis was based on magnetic resonance imaging (MRI) criteria or genetic testing. Results Total number of children with CACH enrolled were [illegible]

nosed as having MS. Methods: Pediatric patients (<18 years old) with two MS diagnosis claims from January 1, 2010, to December 31, 2016, were identified from the MarketScan Commercial Database. The index date was defined as the date of first MS diagnosis, and patients were follow

ors for the development of hepatocellular carcinoma.

be (ETT) is important in these cases. A larger sized ETT may lead to trauma and a smaller one would result in leakage and risk of aspiration. Both situations demand an immediate tube change, thereby complicating the condition. The physical indices- based formulae have often failed to j

cer survivors. This phantom is modeled in the FORTRAN programming language and is not readily applicable for dose reconstructions for survivors treated with contemporary RT whose treatment plans were designed using computed tomography images and complex treatment fields. Th

nner.).

n pediatric patients compared with adults. It remains unclear why pediatric population is less symptomatic than adults. Children frequently experience respiratory infections and their immune system is in developing stage. However, large proportion of the asymptomatic pediatric popula

: University-affiliated, hospital-based fertility clinic. Patients: A total of 403 IVF/ICSI cycles were conducted from September 1, 2018 to December 31, 2019. Cycles were grouped on the basis of stimulation with follitropin delta vs. follitropin alfa or beta. Interventions: None. Main Outcom

ch pediatric patients with orbital fractures. Methods: A retrospective review of all facial fractures in pediatric patients at an urban level 1 trauma center was performed for the years 2002 to 2014. Patient demographics were collected, as well as orbital fracture location, mechanism of inj

out in North American settings. Validation outside of these settings is warranted. Methods: This was a retrospective database study using data from newborn screening programs in Canada, the Philippines and China. ELASTICNET machine learning models were developed to estimate GA

tions and associated parenting skills on early observed aggression have not been examined. In the present study, we examined the direct effect of a brief, in-home adaptation of Parent-Child Interaction Therapy, the Infant Behavior Program (IBP), on observed frequency of aggressive beh

epartment, all cases of appendectomy for suspected appendicitis over 15 years were analyzed retrospectively for the following items: beginning of symptoms, time from admission to surgery, surgical technique as "open," "laparoscopic" or "converted," if perforated at operation and hist

tion Pour des Troubles de Sante Mentale." Can J Psychiatry 66(7): 667-676.
A-treated or SGA-naive. METHODS: SGA-treated (n = 47) or SGA-naive (n = 37) children (aged 6 to 18 years) with mental disorders and control children (n = 83, no mental disorder) underwent assessment for cardiac function and morphology by echocardiography, aortic pulse wave veloci

earched for the relevant studies published in English from the inception of databases until July 30, 2020. Hoy et al.'s tool was used to evaluate the quality of studies. All search steps, screening, selection of studies, quality assessment, and data extraction were performed separately by tw

resolution) along with drawbacks (e.g., prototyping time/costs). Here, we report benchtop fabrication of multilayer, PDMS-free, thermoplastic organs-on-chips via laser cut and assembly with double-sided adhesives that overcome some limitations of traditional PDMS lithography. Cut a

ression materials were tested: irreversible hydrocolloid and P.V.S. Four disinfection solutions were applied: BirexSE, Opti-Cide3, COEfect MinuteSpray, and CaviCide Spray. Distilled water was used as a control group. Each solution remained in contact with the impression for 5 min. Additi

pective, randomized, crossover, mixed-methods study in which pediatric residents were randomized to either a call or shift schedule during their PICU rotation. Attentiveness, bedside care, perceived knowledge, and professionalism were assessed by the resident participants, attending p

ne differential diagnosis of hyperkalemia may be extensive including increased potassium intake or administration, increased endogenous production, decreased renal excretion, and intracellular to extracellular shifts related to changes in acid-base status. We present a 4-month-old infar

ury monitoring. Six-week-old, 10-kg swine (N = 10) were anesthetized, and an acoustic window was created in the right frontal cranium to provide visualization of an oblique coronal plane and bilateral thalami. Ultrasound contrast agent was administered via a femoral venous catheter as

y although exercising would be of paramount importance for their health in particular during pregnancy and menopause. In addition to physical health benefits, physical activity may influence well-being and resilience, greatly impacting on quality of life. Here we explore the relationship

e child neurologists were surprised at dramatic effects on glucose transporter 1 deficiency syndrome (Glut-1DS) in 2003, KD have been slowly accepted for treatment of epilepsy in Japan. New generation KD including modified Atkins diet (mAD) are preferred to classical KD. KD can be cau

ween the age of 29 and 53 years, who had previously worked in the pediatric hematology-oncology unit and left the unit, were interviewed in depth. The interviews were held using a semi-structured interview form. Each interview lasted for 35 to 45 minutes. RESULTS: In the study, 5 mai

e presence of > 20% blasts in the peripheral blood or bone marrow. The blasts could be characterized as either myeloid (60-80% of cases) resulting in acute myeloblastic leukemia or lymphoid (20-30% of cases) resulting in acute lymphoblastic leukemia. In rare instances, a blast crisis cou

ead to death. The balance between an effective antiviral response and dysregulated immune response is the key factor determining the severity of COVID-19 progression. A systematic review was performed using the NCBI-PubMed database to find the articles related to COVID-19 immu

nal Glenn procedure in an infant with hypoplastic left heart syndrome. (Level of Difficulty: Intermediate.).

ORM versus FP/SAL for paediatric asthma. Material and methods: We have searched PubMed, Embase, Web of science, EBSCO, and Cochrane library databases through August 2019 for randomized controlled trials (RCTs) assessing the effect of FP/FORM versus FP/SAL for paediatric asth

e and should be improved. OBJECTIVES: The aim of this evidence implementation project was to evaluate the current practice and implement the best practice related to promoting patient education in a children's hospital in Tabriz, Iran. METHODS: A clinical audit was undertaken using t

are emphasized. Learning points are seen in the light of new guidelines. (Level of Difficulty: Intermediate.).

f five years. In the research study, we included newborns with a nuchal cord (tight and loose) and newborns without a nuchal cord (control group). We were tracking and recording the Apgar score and the conventional cardiotocography (CTG) findings in both groups. The development o

were detected in the patient's left ureter. The stone was expelled upon consultation with pediatric urology department. The patient was treated according to culture antibiotic sensitivity report and continued to be followed-up.

delays, and no imaginative or symbolic representative play. Investigation of the etiology of her developmental delays revealed a genetic diagnosis of a 9p24 deletion by chromosomal microarray analysis. The possibility of an additional co-occurring disorder of autism spectrum disorder (

difficulty in managing patients with WS with complex cardiac defects. To our knowledge, this is the first reported single-ventricle physiology in a patient with WS. (Level of Difficulty: Advanced.).

hospitals located in Antalya city. The data were collected using the Personal Information Form and Healthcare Environment Survey. The study was conducted between December 2018 and January 2019. RESULTS: The scale scores of the nurses were found to be moderate (4.20+/-0.86).

hippocampal sclerosis with intractable seizures persisting lifelong. Other important etiologies in children include pre-, peri-, or postnatal brain injury, low-grade neoplasms, vascular lesions, and neuroimmunological disorders. Cognitive, behavioral, and psychiatric comorbidities are com

not C8, interacts with targets that can explain antiseizure effects, for example, peroxisome proliferator-activated receptor-gamma (eliciting mitochondrial biogenesis and increased antioxidant status) and the alpha-amino-3-hydroxy-5-methyl-4-isoxazolepropionic acid receptor. For such

cal calprotectin in different causes of colitis and to measure the cut-off level to differentiate between IBD and non-IBD colitides. Material and methods: This prospective study was conducted from June 2018 to May 2019. The study included all patients aged 2 months up to 18 years who

orted several times in the literature. Healthcare providers need to be cautious with catheter placement, maintenance, and removal to prevent such a complication. Hereby, we review the literature about this complication after presenting two incidents of umbilical venous catheter rupt

l. In this regard, the purpose of this work was to study the relationship of spirometric parameters reflecting bronchial patency with nutritional status in children with asthma. Materials and Methods: The study involved 54 patients with BA at the age of 8 to 17 years, 33 boys and 21 girls

e whether impaired CAR is associated with neurologic outcome. Methods: Four-week-old swine underwent seven minutes of asphyxia followed by ventricular fibrillation induction and hemodynamic-directed CPR. Those achieving ROSC had arterial blood pressure, intracranial pressure (

gene editing tools. Researchers are working to overcome the challenges associated with gene editing of primary cells, namely, at the level of improving the gene editing tool components, e.g., the use of modified single guide RNAs, more efficient delivery of Cas9 and RNA in the ribonucl

pment of a screening program. Materials and Methods: A total of 424 children were evaluated, 260 children with new-onset T1DM and 164 healthy children with brothers and/or sisters with T1DM.Blood tests for a complex of autoantibodies to insulin (IAA), tyrosine phosphatase (IA-2A)

ction of dietary therapies are under active investigation and appear to involve mitochondria. Once perceived as a last resort, modifications to initiation and maintenance, as well as the widespread use of pre-made ketogenic formulas have allowed dietary treatment to be used earlier in

ctitioner students studying pediatric asthma management. METHODS: A mixed-methods pilot study was conducted with 2 cohorts of graduate pediatric nurse practitioner students (N=21), with each cohort participating for 2 semesters. RESULTS: Significant improvements in pediatric ast

ı is yet to be determined. The aim of the study was to determine the presence of periostin and evaluate its role as a non-invasive marker of allergic inflammation in the nasal secretions of children with atopic BA and AR. Materials and Methods: In 43 patients aged 4-17 years with atopic I

of infrared assistance for radiopharmaceutical injection. We compared results of first-attempt intravenous access (in cubital veins) with and without infrared assistance device for injection of radiopharmaceuticals. All adult patients who underwent nuclear scan in the initial weeks of infr

ıgst college students for effective and timely interventions in the habit-forming years of the life. Methodology: This was a cross sectional study done among college students enrolled in different courses, in Rishikesh municipal corporation area, in Dehradun district of Uttarakhand, India.

)VID-19; however, there is a growing repertoire of reports demonstrating an array of dermatologic manifestations on the skin in children and adults. Dermatologic afflictions have been summarized into different categories several times, with the most recent analysis identifying six clinica

e year in a town in the north of Minas Gerais, Brazil. The Primary Care Assessment Tool Child Edition, adapted and validated for use in Brazil (PCAT-CE), was used to assess accessibility and interviews were conducted with the children's carers to determine the pathway taken to hospitaliz

'his study aims to identify risk factors for hypothermia and determine the effectiveness of current interventions in the pediatric population. Methods: We carried out a prospective observational study in children undergoing anesthesia in our tertiary pediatric unit. We included 869 patie

atment-related risk factor and evidence on traditional cardiovascular risk factors in childhood cancer survivors is accumulating. International surveillance guidelines have been developed with the aim to detect and manage cardiac diseases early and prevent symptomatic disease. There i

at least in the short term. Whether the therapeutic gains of ICBT for OCD are sustained in the long run is unknown. In this study, 61 adolescents with OCD who participated in a randomized trial of ICBT were followed-up 3 and 12 months after treatment. The proportion of treatment resp

roup and has rarely been described in CORD. We report a case of young-onset CORD that was affected by an isolated ABCA4 mutation complicated by the development of MC. Through serial spectral-domain ocular coherence tomography MC has been observed to persist for 24 months l

s of this population. Objective: This study aims to evaluate the effect the Photographs of Meaning Program (POM) has on meaning and purpose, well-being, and perceived social support of pediatric palliative caregivers (PPCGs). Design: Over an eight-week period, participants followed a

are highly oxygen-consuming organs that are sensitive to mitochondrial dysfunction. Moreover, the fibrotic process of skin and islet is closely related to mitochondrial dysfunction as well. This review summarized emerging mechanisms related to mitochondrial dysfunction in different fib

ghlights the unusual physiological features associated with atresia of all head and neck arteries. (Level of Difficulty: Advanced.).

epilepsy in children. Its efficacy in reducing seizures has been confirmed, but the mechanisms remain elusive. The diet has also shown positive effects in a wide range of other diseases, including Alzheimer's, depression, autism, cancer, and type 2 diabetes. We collected fecal samples fron

been profiled on the risk of severe maternal morbidity (SMM), a sentinel measure of maternal health and maternity care. Methods: A cohort study on all births recorded in the Swedish Medical Birth Register from 2000-2014 (N = 1,570,472). Lacking a maternal personal identification nur

unit of a level-III hospital. The study protocol comprised the following parts: (1) pretraining test; (2) theory and practice training session consisting of an explanation of basic vascular ultrasound concepts plus performing vascular cannulation in a model; (3) posttraining test; and (4) evalu

thods The Healthcare Cost and Utilization Project Kids' Inpatient Database (KID) was queried for all cases of TSPR between 1997 and 2016. Factors extracted included patient demographics, use of image guidance, LOS, total cost, and complications, including panhypopituitarism, diabetes

active and caries-free children. In this study, we sought to ascertain if adjunctive dental therapies, including povidone iodine and chlorhexidine, were effective in shifting the cariogenic microbiome from dysbiosis to non-cariogenic health. Design: We recruited young children (ages 2-12 y

tem from 2003-2018, based on the relationship between victim and perpetrator. We characterized incidents, perpetrators and victims in IPH cases by whether or not a firearm was used, and whether a PO had been sought or issued prior to the IPH. Results: We identified 8,375 IPH incide

obal null hypothesis is constructed using a generalized low-dimensional projection for bias correction and its asymptotic null distribution is derived. A lower bound for the global testing is established, which shows that the proposed test is asymptotically minimax optimal over some spars

for the purpose of broadening the geographic distribution of National Institutes of Health (NIH) funding for biomedical and behavioral research by enhancing the competitiveness for research funding of institutions located in states in which the aggregate success rate for grant applicatio

nfected population is very low, ranging from 1-5%. Difficulty in diagnosing the disease clinically in the pediatric population owing to their inability to explain their symptoms often renders a possibility of overlooking this disease. Moreover, new modes of presentation are being reported a

oligohydramnios on ultrasonography with intrauterine growth retardation before cesarean section. After the birth of preterm and very low birth weight neonate, we initially diagnosed as respiratory distress syndrome with a club foot. However, we diagnosed oesophageal atresia with dis

vas carried out in Mansoura University Children's Hospital, Egypt during the period from 2017 to 2019 including 100 NS children (NS group) and 100 healthy controls. NS group included 88 steroid sensitive (SSNS) and 12 steroid resistant (SRNS) cases. All patients were assessed for H.pylo

ember 2018 using a database of online registrants for ophthalmologists and optometrists practicing in Saudi Arabia. The survey included 10 multiple-choice questions designed to elicit information about how ophthalmologists and optometrists deal with commonly encountered case scer

d results in life-threatening manifestations if not managed on time. Although reversible by definition, complications like status epilepticus, intracranial hemorrhage, and ischemic infarction may lead to mortality and morbidity. We report a case of a 9-year-old female patient with posteric

nited customizability. We present a technique using cost-effective and customizable materials for moulding common anomalies (Stahl's ear, constricted ear, and prominent ear). DuoDERM Extra-thin, Steri-strips, and 3M Kind Removal Silicone tape are used to splint the ear in a preferred

n. METHODS: Opioid-naive patients undergoing surgery for tumor resection at a single institution were included. A prescribing algorithm was developed based on surgical approach, day of discharge, and inpatient opioid use. Prospectively collected data included outpatient opioid consu

States annually. In children glioblastoma multiforme accounts for only approximately 7% to 9% of central nervous system tumors. The mean survival rate in adults after diagnosis ranges from 12 to 18 months with standard therapy and 3 to 6 months without therapy. The prognosis in cl

eal, Quebec, Canada, >170 papers were presented on an array of spinal deformity topics. This article represents the top abstracts that were presented at the SRS 54th Annual Meeting. Two of the abstracts were nominees for the Hibbs Basic Research Award, and 8 were nominees for the

uality assessment were undertaken independently by two authors. RESULTS: No randomized clinical trials were identified. Six studies (n = 39) met the eligibility criteria for this review - all were case series or reports and therefore at high risk of bias. All studies reported overall or progress

cular disease, there is limited knowledge of the long-term effects on cardiac function in survivors. Objectives: The purpose of this study was to describe left ventricular (LV) systolic function in long-term allo-HSCT survivors treated in childhood, adolescence, or early adulthood. Methods:

oration of automating manual ventilation as an economically viable alternative to address the anticipated shortage of mechanical ventilators. Many devices have been developed and displayed in the lay press and social media platforms. However, most are unsuitable for clinical use for a

with treatment engagement. Method: Data were collected via retrospective chart review for 410 patients of diverse backgrounds who received an IPC referral in an urban primary care site. Patient-related factors included having multiple types of referral concerns, patient primary care s

less than 20% of the overall population. The University of Arkansas for Medical Sciences (UAMS) leveraged their existing relationship with 18 key community partners. Partners collaboratively developed a COVID-19 Response Strategy to ensure coordinated effort for Latinx and Pacific Isla

wo-stage approaches that delay masseteric nerve transfer until the time of the muscle flap increase spontaneity by maximizing muscle reinnervation from the CFNG. While this 2-stage, dual-nerve approach has been described in adults, we present a series of pediatric patients who unde

as stabilizing excipients for biotherapeutic drugs, providing protection against harsh thermal conditions experienced during distribution and storage. In this work, we report the utilization of a cellulose-based supramolecular hydrogel formed from polymer-nanoparticle (PNP) interaction

with EBV is again rarer. Case: Our patient, a 21-year-old young adult, presented to us with generalized lymphadenopathy, 5 years after liver transplantation. The biopsy of the lymph node was suggestive of peripheral T-cell lymphoma not otherwise specified, which was associated with E

ng loss, cardiac defects, and additional congenital anomalies. While congenital anomalies of the kidneys and urinary tract (CAKUT) are known manifestations of this disorder, studies focused solely on kidney involvement are scarce, and its prevalence is most likely underestimated. This st

gen affects not only the respiratory but also the central nervous system (CNS). The aim of the study is to provide an insight into the molecular mechanisms underlying the damage to the CNS caused by the new coronavirus SARS-CoV-2. Results: By analyzing the literature, we provide evid

nendations for such aggressive tumors, and consequently no definitive treatment guidelines have been established. In this study, we are reviewing our experience in treating atypical teratoid rhabdoid tumor patients. Methods: We reviewed the medical charts of 43 patients with atypica

s, four from the UK and two from the USA, participated in the study. Participants: Parents and families of infants hospitalised in the participating centres between 1 May 2020 and 21 August 2020. Methods: Online-based and/or paper-based survey, querying the visitation policies and the

research and help reduce compartmentalization of knowledge by disseminating clinically relevant advances in the field of cardiac arrest across disciplines. Methods: An electronic search of PubMed using keywords related to cardiac arrest was conducted. Title and abstracts retrieved by

has been termed "multisystem inflammatory syndrome in children" (MIS-C) and is observed in association with the coronavirus disease 2019. The phenotypes of presentation include several characteristic features, including prolonged fever, skin eruptions, neck stiffness, and gastrointes

was to find out the prevalence of acute pediatric burns in a hospital setting. METHODS: A descriptive cross-sectional study was conducted by reviewing the secondary data of burn cases admitted during the years 2016 AD to 2018 AD in a tertiary care hospital after taking ethical clearance

alyzed data from a sample of 135 children and adolescents living with HIV/AIDS and attending a pediatric HIV care service whose parents or caregivers had sought for mental health care for their emotional or behavior problems in the past year. We assessed complementary alternative

national referral hospitals in Uganda. Participants completed a standardized questionnaire that collected data on socio-demographic characteristics, the Knowledge about Childhood Autism among Health Workers (KCAHW) and the challenges related to diagnosing and managing ASD.

children was performed over the 11-year period, 2005 to 2016, in Ali Asghar children's hospital. The rate of antibiotic resistance among patients was evaluated according to demographic data including age, sex, urinary tract abnormities and history of antibiotic consumption. Results: In t

s (UC) by improving discharge processes. Methods: A multidisciplinary team conducted a quality improvement initiative in the EDs/UCs of a tertiary children's hospital network. The team developed discharge interventions through successive Plan-Do-Study-Act cycles. They included stand

controls retinoblastoma in immunocompromised mice, with no obvious damage to the surrounding retina.

from mechanical ventilation, since pulmonary functions were adequate. A small atrial septal defect was identified and closed in cardiac catheterization laboratory to decrease preductal hypoxemia. Diagnostic workup led to the diagnosis of alveolar capillary dysplasia with misalignment

during embryogenesis. It is thought to be rare, fulfils the classiccriteria for diagnosis of an incidentaloma and it can be mistaken for a thyroid nodule. Multinodular goiter is the main endocrine manifestation of DICER1 syndrome, a tumor predisposition syndrome. Careful thyroid examin

eports in the literature, all of them in pediatric patients. Below, we present the case of an adult patient with osteonecrosis of the intermediate cuneiform that was resistant to conservative treatment. Thus, we proposed a surgical approach with good results. Level of Evidence V; Therape

rming are important. Although major surgery and pediatric patient age are noted as risk factors, only a few studies focus on hypothermia as an intraoperative complication in pediatric scoliosis surgery. The aim of this study is to investigate the incidence of intraoperative hypothermia in

r studies providing safety and efficacy data for those patients undergoing upper airway evaluation using THRIVE. Methods: This report is a prospective study of the safety and efficacy of THRIVE in pediatric patients younger than 18 years old undergoing drug-induced sleep endoscopy.

ght the case of a 12-year-old boy who suffered complete traumatic amputation through the distal left forearm. The limb was successfully replanted with successful restoration of sensation and function with the aid of intensive postoperative occupational therapy. A multidisciplinary team

d hematological parameters. Patients with TM who were referred for regular blood transfusion and periodic checkup were included in this study. A questionnaire containing demographic and medical data was provided for all patients by an expert pediatrician. All of the patients were o

nausea and vomiting (PONV), and postoperative analgesia. Objectives: Our study aimed to compare the incidence of ED using inhalational sevoflurane with dexmedetomidine (DEX) versus TIVA with remifentanil. Methods: Eighty-four patients aged 3 - 11 years scheduled for strabismus

ltisystem inflammatory syndrome in children or pediatric inflammatory multisystem syndrome temporally associated with severe acute respiratory syndrome coronavirus 2 infection. We classified the disease into 2 spectrums: the acute phase in severely ill patients and the postinflamma

th refractory epilepsy. Method A total of 70 children with drug-resistant epilepsy who received a KD for at least 12 months were included in the study. The standard 12-lead electrocardiography was performed in all patients before the beginning and in the 12th month of KD. Heart rate,

rior to cardiopulmonary bypass from 82 infants </=120 days old with congenital heart disease requiring surgery at Children's Hospital Colorado. Infants were divided into groups based on pre-operative oxygen saturations: non-hypoxemic (>92%), mild hypoxemia (85-92%), and severe h

resulted with 4,352 finds, and after screening for relevance, 11 articles reporting on 55 arterial grafts were analyzed. All reports were retrospective studies, case reports, and case series, with no randomized controlled trials. Two retrospective series reported better patency of arterial ver

avet syndrome and myoclonic-astatic epilepsy are epilepsy syndromes for which ketogenic diet should be considered early in the therapeutic pathway. Recently, clinical indications for ketogenic diet have been increasing, as there is emerging evidence regarding safety and effectiveness. S

Y-ESO-1 in several malignancies, including triple-negative breast cancer, melanoma, myelomas, and ovarian cancer, makes NY-ESO-1 an attractive antigenic target for cancer vaccines. This study describes a NY-ESO-1 vaccine based on a bio-inspired nanomaterial platform technology, spec

omeostasis in pediatric CF patients and to explore any relationship to lung function. METHODS: We conducted a retrospective cohort study of CF patients, ages 10-18 years, without CFRD and with >/=3 OGTT from 2013 to 2016. Latent class mixture models were used to determine uniqu

al responsiveness may also enhance trust and could be a mitigating factor in the staggering health care disparities unmasked during this pandemic. In this study, the authors outline the rationale for renewed focus on this issue, and offer some initial suggestions for how culturally respon

n estimated 147,000 stillbirths and young-infant deaths, with the highest burden occurring in Sub-Saharan Africa. The following priority data gaps were highlighted: (1) long-term outcome data after infant iGBS, including mild disability, to calculate quality-adjusted life years (QALYs) or d

th which many care providers are uncomfortable. Here, we investigate whether clinical criteria with no subjective auscultatory component are sensitive for cardiac pathology. Methods: A retrospective chart review was performed of all new patients seen in our paediatric cardiology clini

KD) with regard of feasibility, safety, and overall survival (OS). METHODS AND RESULTS: Searches of MEDLINE and Embase identified five hits meeting the search criteria (diagnosis of DIPG and exposure to KD). One additional case was identified by contact with experts. Individual patient

s controversial. OBJECTIVE: The purpose of this study is to describe the clinical characteristics of a 14 case series, focusing on the aetiology of HS and the clinical evolution the patients presented. METHODS: A retrospective observational study was conducted on patients under 14 years-o

cranial graft and the inability to completely cranialize the sinus requires alternate techniques of sinus repair. We describe a technique for repairing an opened frontal sinus and retrospectively reviewed complications related to this approach. Methods All patients, who underwent an orbi

ential advantages of a direct cardiac approach to catheterization. (Level of Difficulty: Intermediate.).

nted between 1988 and 2019 at our institution (Charite - Universitatsmedizin Berlin, Department of Surgery). All cases with new intrahepatic tumors during follow-up were identified. Results: A total of nine patients were diagnosed at a median of 16 years (range, 2-24 years) after surger

ported so far, but genotype-phenotype correlations remain elusive. We reported a boy with a de novo mutation in DHDDS (NM_205861.3: c.G632A; p.Arg211Gln) featuring a complex neurological phenotype, including mild intellectual disability, impaired speech, complex hyperkinetic m

r spinal cord glioma is currently unavailable, surgery of low-grade astrocytoma should be aimed at gross total resection to preserve neurological function and to improve the outcome. Herein, we present a personal case series of four consecutive adult spinal cord astrocytoma patients w

bjective: The aim of this study is to assess the perspectives of facial trauma surgeons regarding the interfacility transfer of patients with isolated CMF trauma. Methods: A 31-item survey was developed using Likert-type scale and open-ended response systems. Internal consistency testing

the treatment of choice. The indications, contra-indications for ECMO, optimization of the care prior to embracing ECMO, cannulation techniques, daily management of ECMO from the practical standpoint, weaning and decannulation, complications, and special circumstances in neona

lity to enter ketosis. METHODS: Two populations were studied, both treated with identical very low-carbohydrate high-fat diets: a retrospective series of children with epilepsy or/and metabolic disorders (2009-2016) and a prospective clinical trial of adults with glioblastoma. Dietary inta

e review summarizes new information on the mechanosensitivity of various cells of connective tissue and nervous system. Participation of mechanical stimuli in the regulation of growth, development, differentiation, and functioning of tissues is described. The data focus on bone remod

e Toolkit for Emotional Coping for Healthcare Staff (TECHS), a free, online, evidence-supported program. TECHS offers self-assessment tools for traumatic stress reactions and three coping tools that are rooted in cognitive behavioral and family therapy principles. TECHS, which comes in

nnot recapitulate the in vivo complexity of neural systems. The search for valuable models of neurodegenerative diseases has recently been revived by the addition of 3D culture that allows to re-create the in vivo microenvironment including the interactions among different neural cell t

aining phage against P. aeruginosa keratoconjunctivitis was prepared. The Cystoviridae phage was formulated as in-situ gel-forming formulation which is a solution formulation but turns into gel when it contacts the eye. Therapeutic effectiveness of the in-situ gel forming formulation wa

um of neurological phenotypes. In the current study, we presented a 14-year-old male with a slowly progressive spastic paraparesis with urinary incontinence that later on exhibited atrophy and weakness in the thenar and dorsal interosseous muscles. Magnetic resonance imaging (MRI)

AVF cannulation. Methods: This randomized clinical trial included 20 children under HD via AVF in Sheikh Children's Hospital Hemodialysis Center in February 2014. The first intervention was conducted as the baseline pain assessment (control), then every patient randomly received all

diatric temporal lobe epilepsy has distinct semiologic, electrophysiologic and imaging characteristics as compared with its adult counterpart. The various treatment options for pediatric temporal lobe epilepsy include antiepileptic drugs, resective surgery, vagal nerve stimulation and the

spective hospitalizations to develop and validate a parsimonious model to identify patients with favorable outcomes within 96 h of a prediction, based on real-time lab values, vital signs, and oxygen support variables. In retrospective and prospective validation, the model achieves high a

g-based influenza vaccine with recently available cell-derived vaccines and recombinant baculovirus-derived vaccines. Adults were administered either egg-derived Fluzone((R)), mammalian cell-derived Flucelvax((R)) or recombinant HA (Flublok((R))). CD4 T cell responses to each HA prot

ealthy, Canadian youngsters, assess tracking over time, and evaluate the prognostic implications of early growth. Methods: Data from 2,795 mother-infant dyads from the CHILD birth cohort were classified by feeding modality at 6 months as exclusively breastfed, partially breastfed, or fe

neningitis using recently derived white blood cell (WBC) and protein correction factors. Methods: We retrospectively analyzed traumatic LPs among all febrile infants </=60 days old at two tertiary paediatric hospitals from 2006 through 2018. Traumatic LPs were defined as >/=10,000 RB

457 pregnant women. Of them, 147 women had chronic arterial hypertension (CAH); 109 pregnant women had CAH and secondary preeclampsia (PE); 201 patients had PE. The control group consisted of 105 pregnant women without hypertensive disorders or proteinuria. We performe

record is consequently challenging as current algorithms (computable phenotypes) rely on diagnostic codes (e.g., International Classification of Disease, ICD) in addition to other criteria (e.g., inhaler medications)-but presume an accurate diagnosis. As such, there is no universally accept

evel of Difficulty: Advanced.).

dered to be among India's earliest inhabitants. In this investigation, we focus on the Y chromosomal characteristics shared between the Soliga population and other Indian tribes as well as western Eurasia and Sub-Saharan Africa groups. Some noteworthy findings of this present analysis

ne brain. Brain abscess must be considered when cyanotic patients present with headache. (Level of Difficulty: Beginner.).

e (VRC) during prophylactic administration in children with malignancy and neutropenia. Materials and Methods: This prospective study was conducted at the Belarusian Research Center for Pediatric Oncology, Hematology, and Immunology from May 2017 to December 2019. The prese

ers in Upper Egypt. Associations of possible risk factors with prevalence of recurrent OME were studied. Multi-factor logistic regression analysis was done to recognize the statistically significant risk factors associated with recurrent OME. Results: We collected the data of 2003 pediatric p

h 2020 to 21 April 2020 with COVID-19. Results: Six children had confirmed COVID-19 and four had suspected COVID-19. Six had pre-existing chronic medical conditions. Nine had respiratory failure and needed ventilation. Five children, of whom four had chronic medical conditions, diec

tution using a covered stent. A proximal stent migration during deployment resulted in obstruction of transverse aortic arch and left subclavian artery and was recognized one month after the procedure. This resulted in significant aortic gradients and left arm hypoperfusion. It was mana

/." F1000Res 9: 1108.
predict cardiac iron in young children because the pancreas loads earlier than the heart. The aim of our study was to assess the relationships between pancreatic T2* values and pancreatic iron loading with cardiac dysfunctions and liver and cardiac iron among patients with beta-thalas

pancreatitis in the setting of a SARS-COV-2 infection and associated fluid balance considerations.

ced enuresis, and epilepsy. Study Selection and Data Extraction: The search was conducted to find the role of antiepileptic drugs-induced enuresis in children in studies published in English. Data Synthesis: Enuresis or bedwetting is an underreported adverse drug reaction of antiepileptic

ave developed and applied a novel marking system to localize small lung nodules for the first time in humans. Methods: A radiofrequency identification tag (1.8 mm in diameter and 7 mm in length) that can communicate with a wand-shaped antenna (10 mm in diameter) from the distar

olescents. MATERIAL AND METHODS: We divided participants (aged 6-18 years) with a diagnosis of asthma into two groups according to the GINA severity classification: mild/moderate asthma (MMA) and severe therapy-resistant asthma (STRA). We collected anthropometric, clinical an

n existential distress near the end of life. To our knowledge, there are no published reports of this treatment modality in pediatric patients. Methods: The authors report the experience of a single-center case series of Dignity Therapy in a pediatric palliative care population. The adult Dig

se social factors may negatively impact mealtime structure are not well-understood. We test whether poverty and FI are associated with parenting factors (mental health and parent disciplinary practices), and whether these parenting factors in turn associate with less mealtime structure

rns would increase exclusive breastfeeding rates. We collaborated with healthcare systems experts to analyze and understand our outcomes. Methods: We describe a retrospective cohort study of chorioamnionitis-exposed newborns 35 weeks and older gestation in the Mother-Baby Ur

al growth factor receptor 2 (HER2). The advent of high throughput techniques has allowed the molecular classification of BC and development of diagnostic/prognostic tools to predict disease outcome and progression. However, most molecular classifications and diagnostic/prognostic t

metabolic environment. In contrast to malignant brain tumors that are mostly dependent on glycolysis for energy, normal neurons and glia readily transition to ketone bodies (beta-hydroxybutyrate) for energy in vivo when glucose levels are reduced. The transition from glucose to ketc

anges in metabolic environment. In contrast to normal neurons and glia, which readily transition to ketone bodies (beta-hydroxybutyrate) for energy under reduced glucose, malignant brain tumors are strongly dependent on glycolysis for energy. The transition from glucose to ketone bo

ıvironment. A radically different approach to brain cancer management is proposed that combines metabolic control analysis with the evolutionarily conserved capacity of normal cells to survive extreme shifts in physiological environment. In contrast to malignant brain tumors that are

ıe nurses and 201 caregivers of children hospitalized in a paediatric hospital in Tehran were randomly selected as participants. Data were analysed by SPSS. The data were collected in 2019. RESULTS: Comparison of the subscale "ethical intelligence" with the scale "quality of work life" inc

response to various agonists. Homozygosity or compound heterozygosity for variants in the ITGA2B/ITGB3 genes is the genetic basis for GT. Establishing a molecular diagnosis is definitive and is important for predictive testing. Using multi-gene panels is an accurate, faster, and cost-effe

Our model of this system integrated multiple sources for a more accurate diagnosis: two image-based sources and two clinical-based sources. The image-based sources included apparent diffusion coefficients (ADCs) and the amount of deoxygenated hemoglobin (R2*). More specifically,

m of this study was to investigate the role of HXP in a myocardial infarction (MI) mouse model. The mice were randomly divided into 3 groups and subjected to surgical ligation of the left anterior descending (LAD) coronary artery or sham surgery (n = 6 for each group) and treated with H

. We hypothesized that the clinical, biochemical and radiographic characteristics of pediatric IBD at diagnosis were associated with subsequent initiation of biologic therapy. Methods: We performed a retrospective analysis of the charts of all pediatric patients diagnosed with IBD at our

ky abdomen pain and a cystic lump in left abdomen diagnosed as tubular duplication cyst of descending colon. Colonoscopy is a good investigation tool for diagnosis of colonic duplications; however, it may not be true in all cases. She was managed with left hemicolectomy and excision

assess the knowledge, attitude, and practices of mothers of children between 6 to 24 months of age regarding complementary feeding. METHODS: This Knowledge, Attitude, and Practice Study was conducted among 250 mothers in Kathmandu Medical College and Teaching Hospital fro

y evaluated hip joint position and lower extremity muscle activity of healthy infants in common body positions, baby gear, and orthopedic devices used to treat hip dysplasia (the Pavlik harness and the Rhino cruiserabduction brace). Surface electromyography(EMG) and marker-based m

ous paracetamol on the hemodynamically significant patent ductus arteriosus (hsPDA). METHODS: A total of 75 infants of <28 week-gestational age were enrolled into the study which was retrospective. Prophylactic paracetamol as the experimental group and none-prophylaxis group as

ıthology, infant assessments, genetics, and imaging support the principles of developmental neurotoxicology that guide research in prenatal exposures. Important to research design is accurate assessment of amount, frequency, and timing of exposure which benefits from accurate self-r

n one laboratory received hemodynamic-directed CPR, a resuscitation method where high quality chest compressions are provided and vasopressor administration is titrated to coronary perfusion pressure (CoPP) >/=20 mmHg. Vasopressors are given when CoPP is <20 mmHg, in sequer

elehealth services could be effectively managed by a dedicated telehealth coordinator. In doing so, telehealth referral and consultation processes were efficient and clinicians felt better supported as they adjusted to new processes for engaging with patients. We have conducted a retros

y the spectrum of mutations in CYP21A2 gene in 30 patients with salt wasting form of CAH in Sri Lanka. Methods: Allele specific polymerase chain reaction was carried out using mutation site specific primers for eight mutations (P30L, I2G, 8bp deletion, I172N, E6 cluster, V281L, Q318X ar

ons that can support the well-being of women HCWs during a pandemic. Design: This scoping review is registered on Open Science Framework (OSF) and was guided by the JBI guide to scoping reviews and reported using the Preferred Reporting Items for Systematic reviews and Meta-A

etogenic diet (KD) - a high-fat, low-carbohydrate treatment for medically refractory epilepsy - has been suggested as an alternative strategy to inhibit tumor growth by altering intrinsic metabolism, especially by inducing glycopenia. METHODS: Here, we examined the effects of an experi

cioeconomic status and risk of and/or outcomes after in-hospital cardiac arrest. Two reviewers independently screened the titles/abstracts and selected full texts for relevance. Data were extracted from included studies. Risk of bias was assessed using the Quality In Prognosis Studies (QU

screening; quarantine and COVID free, in order to screen admitted patients and to reduce the risk of cross infection. From 3 April until 29 May 2020 (56 days), 662 patients and caregivers underwent rapid serological tests for a total of 1397 assays. No patient or parent with SARS-CoV2 i

n January 2012 and December 2019 were recruited. Patient records were examined and data on age of diagnosis, gene mutation, age of first screening endoscopy, number of endoscopies, number of gastric and colonic polyps, associated pathology, medications, symptoms and FAP-rela

treatment, factors differentiating empiric first-generation cephalosporin (FGC) versus third-generation cephalosporin (TGC) coverage, and factors associated with unnecessarily broad-spectrum definitive antibiotic treatment. Methods: This was a retrospective chart review of children ad

simultaneously aiming to decrease the attrition of basic science knowledge. Multiple pedagogical strategies have been explored to achieve this goal. We have found that simulation is a viable medium to integrate basic science within standardized patient encounters for early medical stu

aking into account the interaction effects of genetic variants with three psychosocial factors: depressive symptoms, anxiety symptoms, and social support. Analyses were performed using a two-stage design in a sample of up to 128,894 adults from 5 ancestry groups. In the combined me

nple of U.S. women. Methods: A sample of 8,541 U.S. women ages 22-35 years from two cohorts of the Growing Up Today Study completed a 2016 questionnaire measure about receiving/seeking SRH information before age 18 years. Adjusted log-linear models assessed differences in S

and specialty care clinicians within the University of Utah Health system (40 surveys, 12 interviews). Results: Three key themes emerged regarding providers' collection and use of FHH: (1) Strategies for collecting FHH vary by level of effort; (2) Documentation practices extend beyond the

of infection in hospitalized patients who do not have a clear infectious contact history. Recently, we encountered two seemingly separate COVID-19 clusters in a tertiary hospital. Whole viral genome sequencing distinguished the two clusters according to the viral haplotype. However, th

article, we presented the clinical characteristics, molecular profile, and outcomes in 29 unrelated families with affected children (30 cases total). The mean age at onset of illness was 10 months (+/-14.58), whereas the mean age at referral for molecular diagnosis was 29.44 months (+/-28

ospective cohort study. Setting: Not applicable. Patients: A total of 10,318 cases of IUI (3,015 MIGLIS and 7,303 DGC) between October 2013 and September 2019. Interventions: None. Main Outcome Measures: Sperm analysis, subsequent pregnancy outcomes, and complications. Resul

pment, acceptability, and intention to use the mobile app Vaccipack, which was designed to promote uptake and completion of the adolescent HPV vaccine series. Methods: Development of the mobile health (mHealth) content was based on the integrated behavioral model (IBM). The

nterest in the utilization of diet in other medical disorders, including autism spectrum disorders, traumatic brain injury, degenerative neurologic disorders, and cancer. As the utilization of dietary therapy expands, there are several issues that need to be addressed and better characterize

athy is affected by small group interactions with patients with neurological disorders, and to investigate if changes in empathy persisted over time. Materials and Methods: Eighty first year medical students participating in a Neuroscience Module interacted with a variety of neurological

ophageal symptoms, we hypothesized that eosinophil/mast cell-directed therapy may potentially benefit SSc patients. Herein, we determine the association between esophageal mast cell quantities, gene expression and clinical parameters in order to identify SSc patients who may bene

often lack extensive data. Specially, MRI are uncommon because they require sedation in young children. Moreover, the lack of standardization in MRI protocols introduces a strong variability between different datasets. In this paper, we present a general deep learning architecture for

nptoms. Untreated pulmonary sling carries high morbidity and mortality, most of which is due to the airway and other associated anomalies. Herein, we reported a 40-day-old male infant who admitted to the pediatric intensive care unit with progressive respiratory distress and diagnos

health centers. The study sample comprised 68 primiparous mothers who were in the risky age group and 238 primiparous mothers who were not in the risky age group. It was interviewed with the mothers twice when their babies were one and six months old. Personal Information For

from brain injury. An interdisciplinary approach to education regarding overlap between symptoms of delirium and ABI is important for pediatric intensive care settings, particularly at this time when standardized procedures for delirium screening and management are being increasingl

i-inflammatory agent was evaluated in double-blind, placebo-controlled, cross-over study for 8 weeks of treatment with 8 weeks of washout. Blood samples were collected before (PRE) and after (POST) each 8-week treatment period. Repeated measures analysis of variance (ANOVA) wi

its, who underwent modified Bondy procedure for attic cholesteatoma between 1983 and 2015 at our quaternary referral center for otology and lateral skull base surgery, were analyzed after obtaining permission from institutional review board. The demographic data, air-bone gap bef

ications, prescription, non-prescription and traditional medications have been used for self-medication. Objectives: To determine the reported self-medication use in Rwanda and to determine attitudes and reasons associated with parental decisions to self-medicate their children. Meth

oblems with language and cognitive development and biological functions such as sleep. We aimed to assess screen exposure in preschool children with ADHD and to study the correlation of screen time with the severity of ADHD and parental stress levels. Methods: Children of age 2.5-6

2, a first-tier test panel, including specialized metabolic and routine chemistry tests, was piloted to community-based paediatricians in British Columbia with aims to achieve earlier diagnosis of treatable IEM. Objective: The aim of this retrospective review was to evaluate the diagnostic y

OS: Gene expression data was obtained from the online patient-histology database, GlioVis. GSC mitochondria morphology was examined by TEM. Cell viability and effect on GSC self-renewal was determined via MTS assay and neurosphere assay, respectively. Proteins were evaluated by

natic symptom disorders and related disorders (SSRD) are conditions with serious distress related to physical symptoms as main criterion. They can occur in patients with medically unexplained symptoms (MUS) and in patients with known comorbid chronic medical conditions. Often, co

significantly better survival. The main source of energy for glioblastoma cells is glucose. Therefore, metabolic alterations induced by the use of the extremely carbohydrate-restricted ketogenic diet (KD) as adjuvant therapy are subject of interest in cancer research. PROCEDURE: This stud

A after 24 hours of insulin infusion and rehydration. On day 2, she was diagnosed with COVID-19. The DKA relapsed and required restarting insulin. She developed leukopenia, neutropenia, and high ferritin. Upon recovery, she was discharged for self-quarantine. Severity of DKA in childr

of this review is to analyze how KD can target these different medical conditions, highlighting possible mechanisms involved. Areas covered: We have conducted an analysis on literature concerning KD use in mitochondriopathies, AHC, brain tumors, migraine, and ASD. Expert commenta

ents. Nearly 2 decades of clinical research on telepsychotherapy treatments with children with neurological conditions has the potential to inform emerging clinical practice in the age of COVID-19. Toward that end, we synthesized findings from 14 clinical trials of telepsychotherapy prol

rom PubMed, and eligible reports were included and calculated using meta-analysis method. Results: Four studies were included for the association of ERCC gene polymorphism with osteosarcoma risk, and nine studies were recruited into this meta-analysis for the relationship between

are synthetic lethal in some contexts, but simultaneous mutations in both ARID1s are prevalent in cancer. To understand if and how cBAF abrogation causes cancer, we examined the physiologic and biochemical consequences of ARID1A/ARID1B loss. In double knockout liver and skin, ag

ASCVD risk prediction across broader diverse, real-world populations. We developed ML models for ASCVD risk prediction for multi-ethnic patients using an electronic health record (EHR) database from Northern California. Our cohort included patients aged 18 years or older with no pri

ylaxis in a variety of clinical settings. Hypersensitivity reactions have not been well described; however, isolated cases have been reported. No cases of desensitization to echinocandins have been previously described. Case Presentation: In this report, we described a 14-year-old female

umonitis also affects the paediatric population, and is often associated with exposure to antigens in the home environment and with the pastime activities of children. OBJECTIVE: The aim of the study is to present the current state of knowledge on hypersensitivity pneumonitis in childre

nsfected GnRHR cells exposed to serum of PCOS patients. Design: Cross-sectional matched case-control study. Setting: University-based research facility. Patients: Sera from 200 patients with PCOS from the Pregnancy in Polycystic Ovary Syndrome II (PPCOS II) trial and from 200 race, pa

, emphasizing the need for innovative therapies. Using genome-scale CRISPR-Cas9 screens, we identified B-cell receptor (BCR) signaling, specific transcriptional regulators and one-carbon metabolism as vulnerabilities in BL. We focused on serine hydroxymethyltransferase 2 (SHMT2), a k

kedly elevated, and muscle pain and myoglobinuria may be present. The severity of illness ranges from asymptomatic elevations in serum muscle enzymes to life-threatening disease associated with extreme enzyme elevations, electrolyte imbalances, acute kidney injury and disseminate

es in microbially produced and modified metabolites such as short chain fatty acids and secondary bile acids may contribute to chronic inflammation, positive energy balance and endocrine changes, and represent potential mechanisms linking insufficient sleep and circadian misalignmer

infected with COVID-19 and delay accessing hospitals even in an emergency which can be very detrimental to child health. Here, in this article, we would like to present eight patients delayed in admission to the hospital to draw attention to the harmful consequences of COVID-19 fear ir

icated by acute myocarditis. The patient was successfully treated by a single dose of eculizumab after six doses of therapeutic plasma exchange (TPE) were inefficient to prevent the cardiac complication. Hepatotoxicity was observed after a single dose of eculizumab. Hepatic and cholest

huge treatment gap. Evidence to guide this work has been limited. This study assesses the competence of village clinicians (VC)- mostly paramedical providers- in the diagnosis and management of a presumptive case of childhood epilepsy and its determinants. Methods: A cross-sectiona

ttest control group. The intervention group consisted of children diagnosed with functional constipation (n=20) and fecal incontinence (n=21) and their mothers in the Pediatric Surgery Clinic. The control group consisted of healthy (n=20) children who applied to two different Family He

en key medications in lowering pulmonary arterial pressure and managing right heart failure. (Level of Difficulty: Beginner.).

pacted on the epidemiology of pneumonia. Furthermore, sensitive diagnostic tests and better sampling methods in young children improve aetiological diagnosis. Objectives: To produce revised guidelines for pneumonia in South African children under 5 years of age. Methods: The Paed

ound (FUS) to transiently open BBTB and delivered radiolabeled nanoclusters ((64)Cu-CuNCs) to tumors for positron emission tomography (PET) imaging and quantification in a mouse DIPG model. First, we optimized FUS acoustic pressure to open the blood-brain barrier (BBB) for effectiv

es. Here, we demonstrate the fabrication of large-area plasmonic gold (Au) nanodisk arrays that enable photothermal intracellular delivery of biomolecular cargo at high efficiency. The Au nanodisks (350 nm in diameter) were fabricated using chemical lift-off lithography (CLL). Nanoseco

adults and children in the European Union in 2008. Sugammadex use in children has been reported in the United States, but to what extent is not clear. Aims: The aim was to describe the utilization pattern of NMB agents and factors associated with the use of reversal agents (neostigm

Is cultured in vitro retain an immature metabolic phenotype that limits their application, and little is known about the underlying molecular mechanism controlling mitochondrial metabolic maturation during human induced pluripotent stem cells (hiPSCs) differentiation into cardiomyoc

ectively target tumor cells while negatively impacting the health and vitality of normal brain cells. In contrast to brain tumor cells, which lack metabolic flexibility and are largely dependent on glucose for growth and survival, normal brain cells can metabolize both glucose and ketone bo

semia and sickle cell disease, has provided a promising opening for research directed at relieving -globin repression mechanisms and, thereby, improve clinical outcomes for patients. Various gene editing strategies aim to reverse the fetal-to-adult hemoglobin switch to up-regulate -globi

tomy. Molars were divided into 3 equal groups (24 teeth each) designated to NHA (group 1), MTA (group 2), and FC (group 3) as pulp medicaments. Treated teeth were finally restored with stainless steel crowns. Subjects were monitored clinically and radiographically after three, six, anc

gs and hospital data of all patients referred to the Radiology department of the University College Hospital, Ibadan for ocular Ultrasound between January 2014 - December 2018. Results: There were 142 patients, aged 1-85 years, (median age =28 years). 72 (50.7%) patients were under

nough VCE allows for thorough examination, reviewing and analyzing up to eight hours of images (compiled as videos) is tedious and not cost effective. In order to pave way for automation of VCE-based GI disease diagnosis, detecting the location of the capsule would allow for a more fo

284 midstream urine specimens were collected using standard aseptic techniques from 284 children who were diagnosed with suspected UTI. Urine culture and bacteria isolation were performed following standard bacteriological techniques. The Kirby-Bauer disk diffusion technique anc

osis still relies on a combination of clinicopathologic and laboratory assessments. Methotrexate may be recommended to minimize disease progression and morbidity.

maternal history, birth history, medical history, current clinical presentations in the clinic or hospital, paraclinical data, cardiac diagnoses based on the International Classification of Diseases, 10th Revision (ICD-10), disease management plans, and medications were entered into a questi

s were randomly assigned to the intervention or usual care group. The intervention website guided children to create digital storyboards over 2 weeks by directing them to answer legacy questions about themselves and upload photographs, videos, and music. Families received a copy c

In turn, we describe how cooperative groups can benefit from collaborating on pediatric studies through broadening of data dictionaries, data repositories, and reach in palliative care research communities.

self-adjusting-type) to a plot of the calculated Mosteller BSA values versus BW was then investigated. The correlation between the BSA values estimated by these equations and the Mosteller BSA values was established by the Spearman rank correlation test. Bias and precision were eva

nalysis was conducted using the random-effects model. The pooled prevalence of gastrointestinal symptoms was expressed as proportion and 95% CI. Results: The search identified 269 citations. Thirteen studies (nine case series and four case reports) comprising data for 284 patients we

vere fever and abdominal pain. Further examination revealed an abdominal mass. Biopsy of the mass was the mainstay of diagnosis, with histological findings of typical filamentous fungal hyphae and zygospores, surrounded by eosinophils. Conclusion: Role of surgery was limited to esta

ractice, d) Rating of the quality of ethics education during residency, e) Agreement or disagreement regarding ten ethical scenarios, and f) Confidence level in handling 21 different ethical situations.The response to the survey questions was based on a Likert scale; the survey was electro

age group (>5 years old), there should be a high degree of suspicion of alternative causes when evaluating patients presenting with clinical features suggestive of asthma or patients who do not respond well to asthma therapies. This study will highlight common conditions that may mim

dermatitis to wound-care products.

observational study of children (</=15 years) who visited pediatric clinics and had at least 1 drug prescribed over a 12-month period (January to December 2018). Results: A total of 865 drugs (mean 1 and SD 0.24) were prescribed to 326 children. Off-label was identified in 39.4% of the dr

ase levels. Creatine kinase, Lactate dehydrogenase and aldolase which are not available in our center could not be evaluated. There was remarkable clinical improvement 3 weeks after the onset of systemic corticosteroid therapy. Our case highlights that relying on these normal enzyme

art. This finding may further suggest the compartmentalization theory of the horizontal rectus muscles where the muscle is separated into two nonoverlapping superior and inferior zones, each working independently of the other. In this report, we present the case and suggest surgical t

in a pediatric outpatient clinic designed and implemented a standardized intervention to support AYA with hemoglobinopathies in navigating the transition to postsecondary education. A quality improvement (QI) project was initiated to support the referral of all eligible patients with he

en carried out by portable gas chromatograph device (OralChroma(TM)). Results: A total of 67 children (44 girls and 23 boys) were included in this study with a mean age of 5.3 years (SD = 1.7). The majority of the questionnaires (n = 44, 65.7%) were filled by accompanying fathers. One ir

n primary CH and 49 healthy matched controls. All patients with HI underwent examination by an otolaryngologist, and in patients with sensorineural loss, brainstem evoked response audiometry was performed. A next-generation sequencing (NGS) panel for genes involved in deafness v

Materials and methods: Twenty caries-free premolars were used. Each tooth was divided into four parts. The first two parts were assigned to the bonded group, where a bonding system was used before sealant application. The remaining two parts were treated only with a fissure sealan

l score indicated low to moderate readiness. The key informants scored the highest on legislation, mandates, and policies (7.9), which was followed by the knowledge of CM prevention (7.2), institutional resources and links (5.2), and informal social resources (noninstitutional) (5.2). How

s provide the location of medical centers, the assignment of equipment to such locations, and the additional hours of specialists required to meet official standards of demand. Available equipment with idle capacity should partly satisfy the requirement for exams within the current infra

all the death charts of 1138 children, the team determined that 15% (172) of all deaths could have been prevented and the preventability increased with age. Only 2% of the neonates died of preventable causes, while 53% of the children of 6 years of age or older died of preventable cau

to ascertain information about lifestyle factors. We constructed a logistic regression model to evaluate the predictors of RE. RESULTS: Close to a third of the children had a RE. Of those identified as having the condition, 60% did not wear glasses (newly diagnosed). Nearly all the children

e topographical features using a noncontact optical profilometer. Methods: A total of 30 arch wire specimens were used, including 10 low-friction TMA (TMA-Low), 10 conventional TMA (TMA-C), and 10 stainless steel (SS), (Ormco, Orange, CA, USA) measuring 0.016 x 0.022 in. The static

oint Likert-type scale was used to rank the participants' responses, and the relative importance index (RII) approach was used to analyze the relative contribution of each indicator to its main theme.The factor and parallel analysis methods were used to assess the factorial and unidimens

c characteristics of patients, peritonitis rates, and clinical outcomes were collected. Results: In total, 131 children [boys, 68 (51.9%)] underwent automated PD for 305 years. The most common age group was 6-12 years (61 patients, 46.6%). A total of 74.0% of patients were new to dialys

les of BMP4 in hepatic glucose metabolism. Through a comprehensive analysis of the 14 types of BMPs, we found that BMP4 was one of the most potent BMPs in promoting hepatic glycogen accumulation, reducing the level of glucose in hepatocytes and effecting the expression of gene

ulture and MGIT 960 to compare the difference in isolation rate, time to detection and contamination rate. First line drug susceptibility testing was performed using MGIT 960 SIRE kit. RESULTS: MTB was isolated from 8.6% (29/336) of GA samples by one or more of the culture methods.

s as they approached end of life. The heartbeat was then overlaid to a song or voice recording or kept as a stand-alone file. An artistic embellishment of the phonocardiogram was also created. Parents were surveyed about their experience with the Music Therapy Heart Sounds (MTHS) p

greb Children's Hospital. Data were collected from these institutions and a retrospective study covered the 2010-2015 period. The cause of fracture and diagnosis were coded with the help of the International Statistical Classification of Diseases and Related Health Problems. Operative tr

pironolactone. To our knowledge, this is the first case of a patient with LS recovering from severe hypertension.

dit was carried out using the online JBI Practical Application of Clinical Evidence System and Getting Research into Practice Program. Six audit criteria based on available evidence were used. Phase 1 of the project was the baseline audit, phase 2 the implementation of the best practice, a

ult in the right eye to 0.5% apraclonidine tests. HS is often related to injuries of the brain stem, upper spinal cord, lung apex tumors and lesions, aortic coarctation, cervical lesions, and carotid lesions have been reported. However, it is rare secondary to heart surgery among the pediatric

arital status, and intracranial volume were the covariates. Race was the moderator. To analyze the data, we used mixed-effects regression models. Results: In race-stratified models, high parental education was associated with smaller thalamus volume in White but not Black children. In

ts independently analysed NBI features of each polyp for color, vessel pattern, surface pattern, and any combinations thereof to develop a classification scheme to differentiate low-risk polyps (fundic-gland or hyperplastic) from high-risk polyps (adenomatous or adenocarcinoma) and fu

lysis was performed through the conventional content analysis. To achieve accuracy and trustworthiness of the data, the Lincoln and Guba criteria were used. RESULT: The results of the study can be summarized as: "conflict between human considerations and bureaucratic structure," "

acids, which are part of a commonly used variation of the diet (the medium-chain triglyceride ketogenic diet), have been shown to directly inhibit AMPA receptors (glutamate receptors), and to change cell energetics through mitochondrial biogenesis. Through these mechanisms, mediu

emic (G-I; n = 14), 142 +/- 18 mg/dL values accepted as hyperglycemic (G-II; n = 9) and 89 +/- 9 mg/dL accepted as hypoglycemic (G-III; n = 9). After the experiment, animals were sacrificed under general anesthesia. Their pancreatic tissues were examined histological methods and numbe

l. Three weeks prior to the current admission, she was treated with dapsone (1 mg/kg/day in two divided doses) for 8 days by a local doctor for lichen nitidus. She was managed successfully for DHS with intravenous (IV) steroids followed by the oral steroid. This case is being reported to

cenarios focused on full cardiopulmonary arrest; neither team had access to real-time CPR feedback technology. The primary outcome was CPR quality. Secondary outcomes included workload assessments of the team leader and CPR Coach using the NASA Task Load Index and perceptio

e was a direct relationship between STS-EACTS risk category and death rate in both units. By multivariable logistic and linear regression, there was no difference in mortality between mixed unit and CICU death rates within STS-EACTS risk categories. We found no difference in outcomes

nent.

binary individuals assigned female at birth who were between 19 and 36 years of age and identified as a sexual minority. Data were analyzed using a thematic analysis approach that involved inductive and deductive coding to identify themes. Results: Three broad themes were identified

x, ventilation method (endotracheal intubation vs. supraglottic airway), initial rhythm (pulseless electrical activity vs. asystole), epinephrine administration, bystander treatment prior to EMS arrival, time from collapse to EMS arrival, automatic external defibrillator (AED) placement, and

ics, have the potential to improve patient care and integrated behavioral health services to mitigate the public health impact of ACEs. In the current study, bilingual and bicultural WNs helped to deliver an ACEs screening and response to predominately Latinx patients in a pediatric servi

nsfused peripheral blood stem cells from donors. Overall survival (OS) was calculated using the Kaplan-Meier method. Twenty-one patients underwent haploidentical HSCT. Fourteen-patients were males. The median age of patients was 15 years. Fludarabine with total body irradiation w

cephalus: systematic literature review and evidence-based guidelines", originally published in 2014. METHODS: The Guidelines Task Force used the search terms and strategies consistent with the original guidelines to search PubMed and Cochrane Central for relevant literature publishe

roup A included patients who had Port-A-Cath insertion using the landmark method (n = 117), and Group B included patients who had ultrasound-guided Port-A-Cath insertion (n = 52). Preoperative, operative, and postoperative data were collected and compared between the two grou

ns including demographics, photographs portraying NIBS, terminologies and factors related to NIBS studies. Findings -: Complete surveys were analyzed from 622 parent participants. A significant number of participants (42.8%) perceived the photographs of NIBS as "risky." Additionally,

ns. Results: The scale consisted of 39 items and 8 sub-scales. The 8 sub-scales exhibited 66.4% of the total variance. Both exploratory factor analysis and confirmatory factor analysis (CFA) revealed that all factor loads were greater than 0.40. The CFA also revealed that all of the fit indices

enting with severe hematuria for 12 hours, with no abnormal findings at a first evaluation, who progressed with severe anemia and urinary retention. Further investigation provided images suggestive of nutcracker syndrome, and endovascular stenting (smart control stent) followed by l

ere monitored and compared. Results: Among patients with DM, the course of viral pneumonia was more severe, as evidenced by a 2.2-fold higher number of people with extensive (>50%) lung damage (p=0.05), an increased risk of death according to the CURB-65 algorithm (1.3-fold, p=

22 degrees C to 25 degrees C or 2 degrees C to 8 degrees C. At each predetermined time point, samples were taken for visual inspection, pH measurement, and analysis by a validated stability-indicating high-performance liquid chromatography (HPLC) method. The viscosity parameters c

ction with chiral biomolecules including antibodies and biosynthesis enzymes. We have generated a set of diastereomeric GroP hexamers bearing a glucosyl modification at one of the residues. The chirality of the glycerol building block had an important impact on the stereoselectivity o

curred in a 114-bed, level-IV NICU with approximately 850 admissions per year and 100% outborn infants. A multidisciplinary team began biweekly meetings to review all UE events, later separating these into preventable and nonpreventable. Important ongoing tests of change included

ults The most common hearing complaint was hearing loss, which was confirmed by audiology tests in almost 100% of the patients, most of whom presented conductive hearing loss. Conclusions It is important to evaluate the complaints, physical examination, and audiology tests in pat

and one conventional (Filtek Z350 XT [Z350XT]), were used. Teeth were divided into two groups of 20 teeth each, 10 per each RBC (n = 10): (1) control; and (2) pretreatment with 2% CHX. For FBF groups, teeth were restored with a single increment; however, for Z350XT, a layering techni

ephritis and C3G. CASE (DIAGNOSIS/TREATMENT): We report that MPO staining in the glomerular endothelium of the post-treatment kidney biopsy was significantly reduced after 3 years of eculizumab treatment and clinical improvement in a 5-year-old boy with initial DDD and second

apable of undergoing a lung function test, that had been followed up for at least 2 years. The patients completed a respiratory questionnaire and underwent a lung function test. RESULTS: We included a total of 24 patients (12 male). The median age at the time of diagnosis was 28 mon

p22.31 deletions. Based on the patient's available genetic and biochemical information, we cannot satisfactorily explain his seizures, strokes, or catastrophic brain regression.

ion were recruited from clinic, diabetes camp, and a diabetes conference and completed a patient-reported questionnaire. 113 (41.7%) reported at least one barrier to DR screening, with missed school and work being the most common (20.7%). Older participants (P = 0.007) and those v

nented in 417 of 736 patients (57%) with MIS-C associated with COVID-19. "Rash" was the sole descriptor of skin findings in nearly half of patients. Case reports and smaller case series provided more detail, outlining a broad range of lesion morphologies (polymorphic, maculopapular, m

ty paediatric resuscitation teams were equally randomized into 2 groups (with or without a CPR Coach). The primary outcome was adherence to PALS guidelines during a simulated paediatric cardiac arrest case as measured by the Clinical Performance Tool (CPT). Video recordings were

ngenital heart diseases were included in the study. Each group consisted of 15 patients between 0 and 5 years of age. All data were collected following anesthesia induction (T1), the 10th (T2) and 30(th) min (T3) of cardiopulmonary bypass (CPB), every 30 min during CPB (T4, T5, T6) and

iasm, and optic tracts. The oseltamivir therapy was discontinued, and the patient followed up with an ophthalmologist outpatient. Conclusion: At a 10-week follow-up visit the patient had 90% recovery of his vision.

neonatal sepsis, antibiotics prescribed, and the number of deaths in the included cohort. Results: 126 neonates were enrolled with 42 from each site. Prematurity (38%) followed by membrane rupture more than 18 hours (25%) were the main risk factors for neonatal sepsis. Ampicillin a

served left ventricular ejection fraction despite doxorubicin >250 mg/m(2) were selected as extreme phenotypes. Rare/low-frequency variants were collapsed to identify genes differentially enriched for variants between case patients and control patients. The expression levels of 5 top-r

97.7, 93.3, and 91.3% were found on comparing ultrasound to CXR findings. Ultrasound is feasible, reliable, and has good interrater reliability in assessing correct ETT position in children.

were retrospectively analyzed. Survival analysis was done by plotting Kaplan-Meier curves. Results A total of 73 patients were diagnosed with ES during 2010 to 2017. Among them, 43 (58.9%) had localized disease with a median age of 24.5 years. Males were 44 (60.3%) and females were

d. In order to study possible mechanisms, we then performed microelectrode recordings of small neurons in surgically resected HH tissue slices. Exposure to ketone bodies decreased spontaneous firing in 5 of 7 small HH neurons. These preliminary results suggest that seizures associated

sive care unit of a tertiary care hospital after obtaining ethical clearance from Institutional Review Committee (Reference Number: 2020-064). The sample size was calculated and 77 neonates with culture-proven sepsis were included in the study. The antibiotic susceptibility tests of the

ents (n = 214) a genetic diagnosis was established based on pathogenic and likely pathogenic variants. Diagnostic yield was higher in consanguineous families (60.1 vs. 39.5%). In 27 patients, genetic diagnosis relied on additional biochemical testing, allowing rapid assessment of the funct

or. This patient responded poor to ketogenic diet and anticonvulsants. We present this case and review the related articles.

thasone in the first 48 h followed by oral prednisolone. The median age of the 255 patients in the study was 5 years. Following the prephase, the peripheral blood absolute blast count was 0 and >/= 1000/microL blasts in 141 (56%) and 29 (11%), respectively. Ten of 199 (5%) patients wit

atin or temozolomide sensitivity of anaplastic astrocytoma (AA), glioblastoma (GB) and medulloblastoma (MB) cell cultures. We then evaluated the changing of copy numbers of MYCC and MYCN and its correlation with cytotoxicity index (CI) in MB cells under NGF exposition. METHODS:

affairs professionals to explore how professionals on campuses might better work together to prevent crises and support students at elevated risk for suicide. Recurrent and emerging themes included barriers impeding their ability to best serve suicidal students, their perceptions on wh

ovide insight into the effects of nutrient manipulation on tumor growth in this condition, we describe our experience in a unique group of patients with known tuberous sclerosis complex who are on the ketogenic diet for seizure control. METHODS: A retrospective chart review was perf

genome. AAV6 vectors expressing reporter gene (firefly luciferase) were delivered to the lungs of C57BL/6 mice through intra-tracheal intubation. Despite the large variation among individual animals, most animals had high and sustained luciferase activity with a peak from 2 to 3 weeks

nation for age, thinning of the corpus callosum, and an abnormally small brain stem. Brain malformations including bilateral polymicrogyria and absence of the corpus callosum can also be observed. Development is severely affected: most affected individuals are nonverbal and nonamb

nd sensitive (97%; 95% confidence interval [CI]: 93-99%) for 12-month survival but had poor specificity (10%; 95% CI: 4-21%). Development of non-neurologic MODS is not helpful to predict long-term neurologic outcome or survival after OHCA.

ensive overview of the novel treatments for patients with T-ALL that are currently under clinical investigation or that are emerging from preclinical research. Significance: T-ALL is driven by oncogenic transcription factors that act along with secondary acquired mutations. These lesions, t

o parental guidance regarding the indications for tonsillectomy in children. Methods The search engine google.it was used to search the websites. The Gulpease index, which is a widely used readability formula ranging from 0 (difficult) to 100 (easy readability), was employed to evaluate

d Embase databases were systematically searched for literature regarding allograft and autograft ACLR in pediatric/adolescent patients. Articles were included if they described a cohort of patients with average age of </=19 years, had a minimum of 2 years of follow-up, described graft

in videoconferencing rounds completed the postimplementation survey. Eighty-eight per cent of staff agreed that the use of videoconferencing to facilitate rounds was an effective strategy to maintain social distancing between team members during the pandemic. Sixty-four percent o

, between January 2014 and December 2018 was conducted. HFNC treatment failure was defined as escalation to non-invasive positive pressure or invasive mechanical ventilation. Multivariable regression analysis was used to identify demographic, clinical, and biochemical parameters a

ed mouse model of medulloblastoma. However, we found that the ketogenic diet did not slow the growth of spontaneous tumors or allograft flank tumors, and it did not exhibit synergy with a small molecule inhibitor of Smoothened. Serum insulin was significantly reduced in mice fed t

ronment, explicit teaching of structured communication, and preparing cofacilitators to adapt to differing skill levels of learners.

increases in delinquent behaviors of boys as well as increases in substance use of boys and girls, with substantially larger effects for boys.

ging two hospitals resulted in a nonsignificant trend toward temporary worse outcomes in pediatric patients requiring ECPR.

salivary flow was assessed before, during, and after treatment. Photobiomodulation therapy was conducted at four points at the sublingual glands with 660 nm, 100 mW, and 0.8 J/cm(2) to each point; eight points at the parotid glands; and six points at the submandibular glands with 80

e C-terminal B chain. This new insulin molecule (Ins-SA) with an SrtA-recognizing motif can be conjugated to diverse groups with N-terminal oligoglycines to generate new insulin derivatives. We further demonstrated that a new insulin derivative synthesized by this SrtA-mediated ligatio

ES-EV Click Chip-takes advantage of specific anti-LINGO-1 recognition and sensitive click chemistry-mediated EV capture, followed by disulfide cleavage-driven EV release. Since the device is capable of specific and efficient purification of intact ES EVs with high purity, ES-EV Click Chip is id

sion: Strategies discussed include virtual visits between parents and infants to promote bonding; virtual parent support groups to encourage information sharing; remote music therapy options which included take-home music kits; diaries, albums, and celebration boards to support part

. In this review, we discuss recent evidence on the prevalence of physical inactivity among Americans, with a special focus on the time spent at the workplace; and the invaluable opportunity that workplace-based lifestyle interventions may represent for improving the prevention of carc

ome of all adult patients with a suspected relapse, defined as the need to intensify eculizumab after tapering or withdrawal of therapy. Results: We describe 11 patients who received renewed eculizumab therapy because of suspected relapse. In three patients with aHUS in native kidney

Persistence diagrams representing the range of values for each spatially unique structural asymmetry were collected within language-related regions of interest across 212 children (mean age (years) = 10.56, range 6.39-16.92; 39% female). These topological data exhibited both leftward

triaging using echocardiography and targeted treatment. Our group has recently demonstrated that a PDA severity score (PDAsc) derived at 36-48 hours of age can accurately predict the later occurrence of chronic lung disease or death (CLD/Death). Using echocardiography, we assess

n of milk PCB levels with infant growth over the first 6 months of life. Methods: A pilot study of a subset of milk samples from mothers with NW (pre-pregnancy body mass index (BMI) < 25 kg/m(2), n = 11) and OW/OB (pre-pregnancy BMI >/= 25 kg/m(2), n = 8) were examined approxim

or assessment of RV and LV systolic and diastolic functions. As well as HbA1c, troponin I, brain natriuretic peptide (BNP), plasma cardiotrophin (CT-1), activin-A, transforming growth factor-beta, and human insulin-like growth factor binding protein-7 (IGFBP-7) measurements. Results: Dia

Spirometry was conducted at pulmonary functions laboratory of Pediatric Allergy and Chest Unit of New Children's University Hospital, Cairo. The one-way analysis of variance was used to test the differences between groups. The Duncan multiple comparison test was used to test the sig

08) strain with or without KD may exert a neuroprotection by modulating host gut microbiota. METHOD: We performed animal study using pentylenetetrazole (PTZ) to induce seizure. Thirty 3-week-old male Institute of Cancer research (ICR) mice were divided in six groups, Normal diet (N

had failed to improve with 2 antiseizure drugs (ASDs) and that was amenable to resective epilepsy surgery, across a time-horizon of 5years. Four strategies were included: (1) resective epilepsy surgery, (2) vagus nerve stimulator (VNS) implantation, (3) ketogenic diet, and (4) addition of

en compared with the literature, our patients manifested distinctive features (Patient 1: primary hypothyroidism; Patient 2: bilateral cryptorchidism) that were not previously described in the duplication 17p13.3 spectrum.

ormed using four antifungal agents based on the Clinical and Laboratory Standards Institute M38 3rd edition. Fluconazole-resistant Aspergillus flavus was identified to be responsible for endocarditis and meningitis as well as fever of unknown origin. Conclusion: The clinicians should be a

colleagues across Canada and internationally. Iterative improvements were made following the Plan-Do-Study-Act methodology. Results: A care bundle, created in October 2015, was divided into three time periods: initial resuscitation/stabilization, the first 72 hours and days 4 to 7, wit

s. Rarely, cardiac arrest can be sudden and unexpected without an obvious pre-existing etiology in an otherwise apparently healthy patient. We present a 16-month-old child who experienced a sudden cardiac arrest following anesthetic induction for a routine urologic procedure. The po

precision-based approaches are then discussed. The considerations and challenges of translational cardiovascular medicine are reviewed, and examples of digital platforms with collaborative, cloud-based, and scalable design are provided.

.RIs between patients with community-acquired versus nosocomial infections and, in each group, between children and adults. Results: Community-acquired and nosocomial RSV-associated ALRIs were diagnosed in 229 and 11 inpatients, respectively. The burden of community-acquired

ne premalignant, proinflammatory stages are treatable, but it has been shown that the rate of progression to more advanced lesions is higher than the rate of regression. Most of the research efforts have focused on understanding the cancer stage, but there is still a large gap in the kno

s corresponding to 14 Mexican cities throughout 2016-2019 using the validated Spanish language version of the GAN Phase I questionnaires. The questionnaires were completed by 35 780 parents of 6-7 year old primary school pupils (children) and by 41 399 adolescents, 13-14 years old

yngitis, irritability, anemia and hyperleukocytosis, and neutropenia. Blasts forms were observed in peripheral blood. Bone marrow smear demonstrated acute lymphoblastic leukemia (ALL). KD has diverse clinical presentations, atypical manifestations, and several complications such as r

one reviewer extracted and another verified data. Two reviewers assessed methodological quality and reached consensus. Data were synthesized narratively. Results: We included seven Cochrane reviews published between 2011 and 2017 containing evidence from 167 randomized cor

in PROSPERO (CRD42018083637). Summary estimates of sensitivity and specificity were conducted using meta-disc Software assuming a random-effects model. Results: We identified 12 eligible studies, which included data from 2177 children, of whom 295 (13.6%) had bacteriologically

of the atrial septum with removal of the muscular limbus. The left atrium was pulled down toward the scimitar vein and a V-shaped incision made at the scimitar vein atrial junction with the space filled with a pulmonary homograft. If the scimitar vein coursed adjacent to the atrium, a V

LIGIBILITY CRITERIA: A systematic MEDLINE literature search identified 367 ARPKD studies published since 1990; however, of these 134 were excluded because they did not report any clinical outcomes (e.g. only histopathological, genetic, protein structure or radiological markers), 19 stu

there is no clinical standard for the neurodevelopmental assessment of these infants. The paucity of data regarding the natural history of neurodevelopment in infants with tuberous sclerosis complex and the lack of a gold standard for neurodevelopmental evaluation present a significa

an Academy of Dermatology and the National Psoriasis Foundations, as well as guidelines published by the German Society of Dermatology, provide considerable insight in managing patients who have this condition. The latest studies on pediatric psoriasis treatment were reviewed, incl

copy (MRS) and/or detection of mutations. The data was extracted in a predesigned proforma and analyzed. Results: We had 12 children with mean age at presentation being 6.8 months (range 3 months to 10 months.). Males were more commonly affected (83.3%, n = 10). Ten children

18. Male/female ratio was 10:8. Mean age of presentation was 37.11 months (range = 6-144 months). Affected siblings were seen in five (28%) cases. All children had spasticity, ataxia, and diffuse white matter changes with similar signal as cerebrospinal fluid on all pulse sequences on M

ed up for 1 year post-index date. Outcomes evaluated included percentage of patients who initiated treatment after MS diagnosis, different DMTs initiated, treatment discontinuation, and switching treatment during follow-up. Results: Of 182,057 patients newly diagnosed as having MS

ustify the purpose leading to repeated laryngoscopy and tube change during intubation. The increase in availability of the modern ultrasound devices have shown promise in these cases. Aims and Objectives: In this study we examine the accuracy of ultrasonography (USG)to assess the a

ne goal of this work was to adapt the current FORTRAN model of our age-scalable computational phantom into Digital Imaging and Communications in Medicine (DICOM) standard so that it can be used with any treatment planning system (TPS) to reconstruct contemporary RT. Addition

ation may contribute to transmission. This review explored several aspects of COVID-19 infection such as its epidemiology, its molecular pathogenesis with respect to angiotensin-converting enzyme 2 receptor and inflammatory mediators, intrauterine vertical transmission, imaging findi

e Measures: Embryo parameters and clinical pregnancy and implantation rates. Results: Ovarian stimulation using follitropin delta resulted in no statistically significant difference in day 3 embryo quality between the control group and follitropin delta group (median 0.50 vs. 0.54 for goc

ury, concomitant injuries, ophthalmologic documentation, imaging, and perioperative records. Results: One hundred sixteen pediatric patients over a 12-year period sustained an orbital fracture. The orbital floor was the most commonly fractured orbital bone in our series (60%). Thirty-

ı in a cohort of infants from Canada using sex, birth weight and metabolomic markers from newborn heel prick blood samples. Final models were internally validated in an independent sample of Canadian infants, and externally validated in infant cohorts from the Philippines and China.

naviors and global ratings of aggression in infants ages 12 to 15 months. Additionally, we examined behaviorally-based parenting skills as a mechanism by which the IBP impacted observed infant aggressive behaviors. Sixty infants with elevated levels of behavior problems were randomiz

tological confirmation of acute inflammation. Surgical morbidity was detected in distinct categories. To evaluate changes over time, 3 time periods of 5 years each were defined. Results: Resulting in a total of 1,956 cases there were 731 in group I, 633 in group II and 592 in group III withi

ty (PWV), and carotid intima-media thickness (cIMT). Body mass index (BMI) z-scores, waist circumference z-scores, systolic and diastolic blood pressure (BP) percentiles for height and sex, and fasting plasma glucose, insulin, triglycerides, and cholesterol were also assessed. Differences l

vo researchers. RESULTS: Out of 3460 articles searched, 28 articles conducted on 16,427 HCWs were included in the study. Most of the HCWs had good knowledge (72.2%), a positive attitude (70.9%), and good practice (78.8%) towards COVID-19. The most important clinical recommend

nd assembled chips are economical to prototype ($2 per chip), can be fabricated in parallel within hours, and are Luer compatible. Biocompatibility was demonstrated with epithelial line Caco-2 cells and primary human small intestinal organoids. Comparable to control static Transwell c

onal contact time of 5 min compromises time for scanning. The materials were evaluated for dimensional stability after the impression of a maxillary complete edentulous template via CBCT before and after being in contact with the disinfectant agents. Measurements were assessed on

ohysicians, and nursing staff. Epworth Sleepiness Scale determined the level of resident fatigue. Statistical analysis utilized a t test of unequal variances. Two focus groups were conducted of resident non-participants and participants. Graduated resident participants and non-participants

nt who developed hyperkalemia during the recovery phase of her PICU course for respiratory failure. A thorough investigation demonstrated that the hyperkalemia was most likely the result of the commonly used antibiotic, trimethoprim-sulfamethoxazole (Bactrim((R))). Potential etiolo

s a weight-based (0.03 mL/kg) bolus. After localization of the imaging plane, CEUS cine clips were acquired for 90 seconds. Bolus injection of contrast agent provided global visualization of cerebral perfusion and highlighted microvasculature in the brain. Preliminary evaluation of bolus ki

between physical activity resilience and well-being in a group of 1107 female residents in the Metropolitan area of Naples.

usal therapy in Glut-1DS and some of mitochondrial disorders, though anti-epileptic drugs are symptomatic therapy. KD can alleviate intractable seizures in epilepsies with brain malformation in addition to West syndrome and Dravet syndrome, etc. KD may work for brain tumor, cancer,

in themes and sub-themes related to these were identified from the perspective of the nurses with working experience in the pediatric hematology-oncology unit in the past with regard to their feelings and thoughts about this experience. These included 1) feelings: (a) fear and anxiety,

ld present with biphenotypic expression for both myeloid and lymphoid blasts. In such cases, about 6-10% of the time, the course of the disease is more aggressive and renders a poorer prognosis especially if there is evidence of extramedullary involvement. We present a case of a 41-ye

unity and inflammatory response published from 1 December 2019 to 15 April 2020. Haematological, immunological and biochemical parameters were extracted and correlated with disease severity, age and presence of comorbidities. Twelve articles were analysed comprising a total of

ma. This meta-analysis is performed using the random-effects model. Results: Three RCTs are included in the meta-analysis. Overall for paediatric asthma, FP/FORM and FP/SAL demonstrate a comparable influence on FEVj (Std. MD = -0.01; 95% CI: -0.04 to 0.03; p = 0.62), FVC (Std. MD =

the Joanna Briggs Institute Practical Application of Clinical Evidence System tool. Eight audit criteria that represent the best practice recommendations for patient education were used. A baseline audit was conducted followed by implementing multiple strategies, and the project was fin

of newborns was monitored by the Munich Functional Scale and the development was evaluated at the end of the first year of life. Results: Statistically, there was a significant presence (P < .001) of Apgar score lower than 7 in newborns with nuchal cord concerning the control group. The

ASD) was also raised by a referring clinician. This case report highlighted the clinical dilemma of diagnosing ASD in those with existing genetic syndromes.

Although the nurses obtained the highest mean score for the patient care subscale (5.09+/-1.04), they obtained the lowest mean score for the distributive justice subscale (2.53+/-1.42). Job satisfaction was higher in nurses who were aged 31-40 years and were continuously working dur

ımonly seen and must be addressed in addition to seizure control. Given the diverse nature of focal epilepsies in children and adolescents, investigations and treatments must be individualized. First-line therapy consists of prophylactic antiepileptic drugs; however, prognosis is poor afte

ı effects to occur, significant concentrations of C10 are likely to be required in the brain. METHODS: To investigate how this might occur, we measured the beta-oxidation rate of (13) C-labeled C8 and C10 in neuronal SH-SY5Y cells using isotope-ratio mass spectrometry. The effects of car

› were confirmed to have chronic colitis endoscopically and histopathologically attending the Gastroenterology Clinic at Alexandria University Children's Hospital. Faecal calprotectin level was measured. Results: We included 110 patients. Allergic colitis was the commonest cause followe

ure in two separate patients in our neonatal ICU. One was removed easily through the umbilical stump, whereas the other required surgical exploration.

with different nutritional status. Assessment of nutritional status was carried out with the calculation of body mass index (BMI), relative body mass index (RBMI), and determination of body fat (% BF). Spirogram parameters were evaluated, including forced vital capacity (FVC), forced exp

ICP), and microvascular cerebral blood flow (CBF) monitored for 4 h. Animals were assigned an 8 -h post-ROSC swine cerebral performance category score (1 = normal; 2-4=abnormal neurologic function). In this secondary analytic study, we calculated PRx and CBFx using a continuous, m

eoprotein of these cells. Despite these efforts, the low efficiency of proper gene editing in true primary cells is an obstacle that needs to be overcome in order to generate sufficiently high numbers of corrected cells for therapeutic use. In addition, many of the therapeutic candidate gen

, zinc transporter 8 (ZnT8A), pancreatic beta-cells (ICA), and glutamate decarboxylase (GADA) were conducted in all the subjects with the enzyme immunoassay method. Results: It was found that the diagnostic utility of individual autoantibodies is not equal and varies with age. The opti

the course of epilepsy. For infantile spasms (West syndrome) specifically, the ketogenic diet is successful about 50% of the time as a first-line treatment. New "alternative" diets such as the modified Atkins diet were created in 2003 and can be started more easily and are less restrictive.

hma test scores (P<.001) of student learning were found in both cohorts at posttest in both semesters. Student satisfaction with the augmented reality technology was found to be high. The focus group discussions revealed that the simulation was realistic and helpful for a flipped classr

BA and AR, the NM was examined using nasal video-endoscopy and (if indicated) computed tomography; the amount of periostin in the nasal secretion was determined by the enzyme immunoassay. Results: Exacerbation of AR was accompanied by a statistically significant increase in the

ared device installation were injected utilizing infrared device assistance. These were compared with those who underwent injection without infrared assistance. Three hundred consecutive patients were studied for success of intravenous injection with and without infrared assistance. C

Sleep Quality was assessed using a validated Pittsburgh Sleep Quality Index (PSQI), which has seven components while Perceived Stress Scale-10 assessed Stress. Results: Poor sleep quality was reported among 66% of respondents. Mean sleep latency among respondents was 27.2 minu

al patterns: urticaria, maculopapular-morbilliform eruption, papulovesicular exanthem, chilblain-like acral pattern, livedo reticularis-livedo racemosa pattern, and purpuric vasculitic pattern. In children, the dermatologic features appear to occur before or concomitantly with other COVID

ation. Of the 376 pediatric hospitalizations assessed, 109 (28.9%) were classified as ACSC admissions. Health service accessibility and utilization scores were low for both the ACSC and non-ACSC groups. No statistically significant differences (p<0.05) were found between the two groups.

nts (<16 y) undergoing emergency/elective surgeries over 2 months. Our team recorded the incidence of hypothermia (tympanic core temperature < 36 degrees C) within 15 minutes of arrival to the postanesthetic care unit. We collected data such as patient demographic, surgical, anes

s growing interest in risk prediction models to individualize prevention and surveillance. This State-of-the-Art Review summarizes literature from a systematic PubMed search focused on cardiac diseases after treatment for childhood cancer. Here, we discuss the prevalence, risk factors,

ponders and remitters remained stable from post-treatment to 3-month follow-up and increased significantly from 3-month to 12-month follow-up. This study suggests that the gains of ICBT for youth with OCD are not only maintained long-term, but that further improvements continue

before its resolution, followed by retinal thinning and macular atrophy with corresponding visual acuity decline. The formation of MC and visual acuity appeared to be directly correlated in ABCA4-related CORD and its manifestation is invaluable in predicting eventual visual loss. We furt

meaning-making curriculum via social media. Following the social media portion of the intervention, a reflection and closure event and a community photograph exhibit were held. Participants completed pre- and post-test measures. Setting/Subjects: Eighteen PPCGs participated in POM

rotic organs and tissues above. First, it highlighted the important elucidation of mitochondria morphological changes, mitochondrial membrane potential and structural damage, mitochondrial DNA (mtDNA) damage and reactive oxidative species (ROS) production, etc. Second, it introdu

n 12 children with therapy-resistant epilepsy before starting KD and after 3 months on the diet. Parents did not start KD and served as diet controls. Applying shotgun metagenomic DNA sequencing, both taxonomic and functional profiles were established. Here we report that alpha dive

mber was used as an indicator for insecure residency status (1.3% of all births). We used Poisson regression models to estimate risk ratios of SMM in migrant women with insecure residency status compared to the Swedish-born or migrant women with long-term residency, adjusting for

uation of the training model. Results: A total of 25 health-care professionals participated in the study. All of them possessed the skills to locate vessels and ultrasound planes, and they performed USGVC using the training model. On a 1-5 scale, the model was rated to have 87.6% fidelity

insipidus (DI), cerebrospinal fluid (CSF) rhinorrhea, and cranial nerve (CN) III, IV, and VI palsies. Multivariate logistic regression was utilized to determine the odds ratio of developing panhypopituitarism, DI, CSF rhinorrhea, and CN palsies for image-guided versus nonimage-guided cases.

years) to enter five enrollment groups, with each group (N = 9-30 participants/group) receiving caries restorative and/or adjunctive therapies, either singularly or in combination (OHSU IRB #6535). Saliva specimens were collected pre- and post-treatment (4-8 weeks) of caries preventive

ents with a total of 9,130 victims. Overall 306 (3.3%) victims were killed in a firearm IPH with PO, 4,519 (53.9%) in a firearm IPH without PO, 176 (2.1%) in a non-firearm IPH with PO and 3,416 (40.7%) in a non-firearm IPH without PO. Based on review of incident narratives, 5.4% (n=451) c

sity range. For testing the individual coefficients simultaneously, multiple testing procedures are proposed and shown to control the false discovery rate (FDR) and falsely discovered variables (FDV) asymptotically. Simulation studies are carried out to examine the numerical performance

ons to the NIH has historically been low. The IDeA States are composed of the Commonwealth of Puerto Rico and the following 23 states: Alaska, Arkansas, Delaware, Hawaii, Idaho, Kansas, Kentucky, Louisiana, Maine, Mississippi, Montana, Nebraska, Nevada, New Hampshire, New Mexi

part from the classical tell-tale signs. In this scenario, medical professionals dealing with the children should be well aware of different modes of presentation of this disease in the pediatric population. This study thus aims to review otorhinolaryngological and ophthalmological manifest

stal Tracheoesophageal fistula on the 2nd day as nasogastric tube insertion was failed beyond 10cm and confirmed by X-ray with a rubber catheter. Right thoracotomy with ligation of the fistula with end to end anastomosis was performed successfully without complications. Breastfeedi

ri infection using H.pylori stool antigen (HpSA) test. Statistical analysis was done using chi-square, fisher exact and Mann-Whitney tests. Results: With regard to HpSA test results, no significant differences were detected between control and NS groups (p = 0.193) and between SSNS and

narios covering different practical aspects of pediatric refraction. The responses were compared using the proportion of pediatric clientele received in the respondents' practices (Group A: <25%, Group B: 25%) and drawing upon concepts from the published literature. RESULTS: One hun

or reversible encephalopathy syndrome who presented with multiple episodes of seizures and bilateral painless loss of vision for 1 day. Due to her severity, a computed tomography scan was sent which revealed a hypodense lesion in the brain. She was admitted to the pediatric intensiv

l position. The DuoDERM is rolled into a putty, placed in the ear, and secured with tapes. This treatment is initiated in the clinic, with weekly splint changes carried out at home by caregivers, and intermittent follow-up appointments. DuoDERM moulding is a safe, inexpensive, highly cust

mption and patient/family satisfaction. Total home dose prescribed was equal to that used in the 8 or 24h, depending on length of stay and operative approach, prior to discharge, divided into 0.15mg/kg doses. RESULTS: The algorithm was used in 121 patients and correctly predicted ou

hildren is better compared to adult tumor onset with a mean survival of approximately 4 years following gross total surgical resection and chemotherapy. There have been few advances in the treatment of glioblastoma multiforme in the past 40 years beyond surgery, radiotherapy, chen

e Hibbs Clinical Research Award.

sion-free survival; however the effectiveness of KD interventions could not be established. Dietary acceptability was not reported. CONCLUSION: The effectiveness and acceptability of KDs in the management of gliomas is unknown and high quality randomized controlled trials are neede

Our cross-sectional cohort study included 104 patients (56% women), age 18 +/- 10 years at time allo-HSCT with 17 +/- 6 years of follow-up. Echocardiography included 2-dimensional (2D) and 3-dimensional (3D) analyses and speckle tracking imaging. In total, 55 healthy control subjects

a variety of reasons. These include failure to understand the clinical needs, complex ventilatory requirements in Covid-19 patients, lack of technical specifications to guide innovators, technical challenges in delivering ventilation parameters in a physiological manner, absence of guideline

show rate, and number of visits with referring clinician. Service-related factors included referral type (warm handoff/ electronic), number of days between referral and intake, and average number of days between IPC treatment sessions. Engagement outcomes included attendance at IP

ander communities with four interrelated strategies: health education, testing, contact tracing, and supported quarantine/case management.

erwent this reconstructive technique. Methods: We retrospectively reviewed all pediatric patients who underwent 2-stage, dual-nerve reconstruction with CFNG and ipsilateral masseteric nerve transfers. Procedures were performed between 2004 and 2016 by 2 surgeons at a single cent

ns to encapsulate and stabilize insulin, an important biotherapeutic used widely to treat diabetes. Encapsulation of insulin in these hydrogels prevents insulin aggregation and maintains insulin bioactivity through stressed aging conditions of elevated temperature and continuous agitatio

BV infection. The Positron emission tomography and computerised tomography (PET-CT) scan showed stage 3 disease. He was treated with standard cyclophosphamide, doxorubicin, etoposide, vincristine, and prednisolone chemotherapy and is currently in remission. Conclusion: Periph

tudy aimed to describe the prevalence and nature of CAKUT and other renal manifestations, in a cohort of KS patients followed at a single tertiary center. METHODS: All patients who were evaluated at the Sheba Medical Center and received a clinical and/or molecular diagnosis of KS, ov

ence that the brain is targeted by this virus. SARS-CoV-2 enters the body with the help of the target proteins: angiotensin-converting enzyme 2 (ACE2) and associated serine protease TMPRSS2 of the nasal epithelium. Brain damage develops before the onset of pulmonary symptoms. The

l teratoid rhabdoid tumor who were treated in King Faisal Specialist Hospital and Research Centre, Riyadh, Saudi Arabia, between 1996 and 2013. We evaluated the overall survival rate and the influence of different clinical features and treatment protocols on survival. Results: The medi

eir impact on parents' ability to visit, care for and bond with their infants. Results: A total of 231 responses were received. Visitation limited to a single visitor with no restrictions on duration was the most frequently reported policy; 140/217 (63%). Visitation policies were perceived as be

these searches were screened for relevance, classified by article type (original research or review), and sorted into 7 categories. Screened manuscripts underwent standardized scoring of overall methodological quality and impact on the categorized fields of study by reviewer teams leac

stinal manifestations with pronounced abdominal pain. Shock and organ dysfunction on presentation are frequent but inconsistent, whereas respiratory distress is typically, and notably, absent. We have reviewed the recent published data aiming to better understand MIS-C, with a focu

e from the Institutional Review Committee (IRC No. 016-2019). The sample size was calculated and systematic random sampling was done. Data analysis was done using Statistical Package for the Social Sciences, version 23. Point estimate at 95% Confidence Interval was calculated along

medicine use, socio-demographic characteristics and types of behavioral problems using a SOCIO-DEMOGRAPHIC questionnaire and the child behavioral checklist (6 - 18) respectively. Logistic regression models were used to explore factors independently associated with specific patterns

General linear model with the Poisson regression was used to evaluate bivariate and multivariate factors associated with limited knowledge about ASD. Results: The study participation rate was 90% with the majority being females (N = 21, 58%). Also, most (N = 20, 55.6%) were mental h

otal, 958 female and 349 male positive cultures were analyzed. Escherichia coli (E. coli) (77.6%) was the most common causative agent of urinary tract infection (UTI) in children and Klebsiella pneumoniae (10.4%), Pseudomonas aeruginosa (2.4%), and Enterococcus spp (2.4%) were less

dardization of the electronic health record discharge workflow and implementation of "mini-after care instructions" and teach-back education. The team used a statistical process control chart to follow the 72-hour return rate, and a chi-square test to compare the pre- and post-interven

of the pulmonary veins. Suprasystemic pulmonary arterial hypertension with persisting nitric oxide dependency remained the leading symptoms. The child underwent bilateral lung transplantation at the age of 28 months. He is well at the age of 44 months.

ation including ultrasound is thus necessary for DICER1 syndrome patients. Case Report: A 4-year-old prepubertal boy was referred to the pediatric endocrinology department following an earlier diagnosis of pleuropulmonary blastoma (PPB) type III, which is a hallmark component of DI

utic Studies; Expert Opinion .

pediatric scoliosis surgery and the associated preoperative risk factors. Methods: We retrospectively reviewed the records of pediatric patients who underwent posterior spinal fusion at a single institution between 2015 and 2019. We recorded the background data, perioperative data,

Ve positioned a flexible laryngoscope to view the larynx, and photographs were taken with no THRIVE flow (control) and with THRIVE flow at 10 and 20 liters per minute (LPM). Upper airway patency was measured using epiglottis to posterior pharynx distance, laryngeal inlet area, and m

n is of paramount importance to maximize function of a replanted upper extremity.

n Desferal chelation therapy and none of them had clinical signs of heart failure. Myocardial gated perfusion SPECT, echocardiography, and complete blood tests were performed for each patient. In total, 24 patients including 14 men (58.3%) and 10 women (41.7%) aged 15-36 years wit

surgery under general anesthesia were randomly allocated into two groups. Patients in group I received sevoflurane and DEX (group I, n = 42), while group II patients received TIVA with propofol and remifentanil infusion (group II, n = 42). Mean arterial pressure (MAP), heart rate (HR), a

atory phase. Neither of them could be classified as mild because there is enough evidence that supports a wide range of complications. The goals of this brief review were to summarize available literature and to give some awareness about the current status of the coronavirus disease 2(

P-wave duration and dispersion, corrected QT interval and QT dispersion, and Tp-e interval were measured. Results All ECG-derived parameters, but P-wave dispersion increased after 12 months of KD compared with the baseline values. However, these changes were not statistically sigr

ypoxemia (<85%). Tandem mass spectrometry was used to analyze 165 targeted metabolites. Partial least squares discriminant analysis and t-tests were used to determine differences among metabolic profiles and individual metabolites respectively. Results: The broad metabolic fingerp

sus venous grafts in upper-limb revascularization for chronic occlusion, but the findings were highly biased. Better patency of arterial grafts did not lead to higher rate of clinical improvement. Antiplatelet and lipid-lowering agents seem to be underused in venous graft recipients and use

Specifically, ketogenic diet response has been investigated in refractory status epilepticus and encephalopathy with status epilepticus during sleep. New targets in neuropharmacology, such as mitochondrial permeability transition, are being studied and might lead to using it effectively i

cifically a plant virus nanoparticle. The 30 nm icosahedral plant virus cowpea mosaic virus (CPMV) displaying multiple copies of human HLA-A2 restricted peptide antigen NY-ESO-1157-165 exhibited enhanced uptake and activation of antigen-presenting cells and stimulated a potent CD8

e trajectories of 2-h OGTT glucose values (2hrGlu) over time. Multivariable linear models were used to adjust for clinical covariates. RESULTS: For 63 subjects, three unique 2hrGlu trajectories were identified: high (impaired glucose tolerance) to higher (n = 8), low (normal glucose toleran

sive PC can be provided even in this extraordinary time.

isability-adjusted life years (DALYs) and (2) economic burden for iGBS survivors and their families. Geographic data gaps were also noted with few studies from low- and middle- income countries (LMIC), where the GBS burden is estimated to be the highest. In this paper we present the p

c for assessment of a murmur from January 1, 2016 through June 30, 2018. Patients were characterized as "low-risk" if they met all of the following criteria: asymptomatic; normal physical examination other than the murmur; no risk factors for congenital heart disease; and age over 12 r

t data were extracted from publications or obtained from investigators. The inclusion criteria for analysis of the data were defined as DIPG patients who were exposed to the KD for >/=3 months. Feasibility, as described in the literature, was the number of patients able to follow the KD f

ld (enrolled between 1st January 2009 and 30th April 2020). Depending on the age at diagnosis (before or after the first 5 months of life), the study cases were divided into two groups: congenital or acquired. RESULTS: Fourteen patients, with a mean age of 8.5 months, were enrolled. Th

itozygomatic craniotomy via an eyebrow incision by a single surgeon from August 1, 2012 to August 31, 2018, were included in this retrospective analysis. Data were collected on patient demographics, pathology treated, operative details, and perioperative morbidity. Follow-up ranged f

y. Eight patients presented with hepatocellular carcinoma (HCC), and one patient presented with epithelioid hemangioendothelioma (EHE). All eight HCC patients had a recurrence of the initial disease that had caused liver failure before transplantation. This was associated with viral reir

ovements, and refractory epilepsy. We defined the electroclinical and movement disorder phenotype associated with the monoallelic form of the DHDDS -related neurodevelopmental disease and possible underlying dominant-negative mechanisms.

vho were operated on during the last few years. Tumor resection was performed in all patients utilizing microsurgical technique and intraoperative neurophysiologic monitoring. We also provide a literature review of the treatment of intramedullary astrocytoma in adults and discuss con

g among facial trauma surgeons yielded a Cronbach's alpha calculation of .75. The survey was distributed anonymously to the American Society of Maxillofacial Surgeons, the North American Division of AO Craniomaxillofacial, and the American Academy of Facial Plastic and Reconstructi

tal period have been described. The follow-up of neonatal ECMO and various system manifestations necessitating careful review will be highlighted.

ake was assessed based upon written food diaries and 24-h dietary recall. Ketogenic ratio was calculated according to [grams of fat consumed]/[grams of carbohydrate and protein consumed]. Ketone levels (beta-hydroxybutyrate) were measured in blood and/or urine. RESULTS: A total c

eling, wound healing, neurite growth, and the formation of neural networks. Mechanotransduction, cellular organelles, and mechanosensitive molecules involved in these processes are discussed as well as the role of the extracellular matrix. The importance of mechanical characteristics

the form of a slide set and a pre-recorded webinar, can be implemented flexibly (e.g., small or large groups, individually, one one-hour administration or multiple shorter sections of time). Ideally, small groups of HCWs engage in TECHS together to help support team resilience. In implen

ypes and the surrounding extracellular matrix (ECM) components. In this review, the new challenges in the field of CNS diseases in vitro 3D modeling are discussed, focusing on the implementation of bioprinting approaches enabling positional control on the generation of the 3D microe

s evaluated by histological examination on day 12 post-infection. Results: The viscosity of selected formulation increased when it was instilled into the eye. The histological results showed edema, abscesses, and destruction of the stromal structure of cornea in groups where no in-situ ge

revealed discrete atrophy of the corpus callosum isthmus and an extended next-generation sequencing panel identified a de novo heterozygous mutation in BSCL2 gene, c.269C > T p.(S90L). Various clinical expression and incomplete penetrance of BSCL2 gene mutations complicate the

three other interventions: Lidocaine gel, lidocaine spray, and needle plate, before venipuncture. Pain perception was expressed and recorded by patients using the visual analogue scale (VAS). VAS scores were compared, and a P value of <0.05 was considered significant. Results: The VA

ketogenic diet. In spite of the multiple antiepileptic drugs currently available, 5-10% of all newly diagnosed cases will remain intractable to medical therapy and should be referred for presurgical evaluation. Resective surgery offers the best chance of seizure freedom in carefully selected

iverage precision (88.6% 95% CI: [88.4-88.7] and 90.8% [90.8-90.8]) and discrimination (95.1% [95.1-95.2] and 86.8% [86.8-86.9]) respectively. We implemented and integrated the model into the EHR, achieving a positive predictive value of 93.3% with 41% sensitivity. Preliminary results

ein were assessed by cytokine EliSpot and intracellular staining assays. The specificity and magnitude of antibody responses were quantified by ELISA and HAI assays. By all criteria, Flublok vaccine exhibited superior performance in eliciting both CD4 T cell responses and HA-specific antib

ormula-fed. WHO z-scores (z) were calculated at birth, 3 months, 1 year, and 3 years. Receiver operator characteristics (ROC) assessed the predictive performance of early weight (WT), weight-for-length (WfL), or body mass index (BMI) z-scores for overweight/obesity at 3 years. Results:

Cs/mm(3). Abnormal cerebrospinal fluid (CSF) WBCs and protein were adjusted downward using a newly derived correction factor (877 red blood cells [RBCs]: 1 WBC), three commonly used correction factors (500 WBCs: 1 RBC; 1,000 WBCs: 1 WBC; peripheral RBCs: WBCs), and a newly

ed a retrospective analysis of gestation course and labor outcomes, calculated risk factors using the Open Epi system and logistic regression method. Polymorphisms of genes controlling the vascular tone were identified in venous blood. Results: There were identified risk factors for deve

ed or rigorously tested computable phenotype for asthma. METHODS: We compared two established asthma computable phenotypes: the Chicago Area Patient-Outcomes Research Network (CAPriCORN) and Phenotype KnowledgeBase (PheKB). We established a large-scale, consensus

include the following: The three most frequent haplogroups detected in the Soliga population are F*, H1 and J2. F*, the oldest (43 to 63 Kya), has a significant frequency bias in favor of Indian tribes versus castes. This observation coupled with the fact that Y-STR haplotypes shared with

ent study included 21 Caucasian patients with malignant hematological diseases (20 patients with acute myeloid leukemia and relapses of leukemia and 1 patient with Non-Hodgkin's lymphoma) aged 2-18 years. All patients were randomly divided into two groups that received different

patients, of which 1016 were males (50.7%). A total number of 310 children have OME, including 159 males (51.3%). The prevalence rate of OME in our cohort was 15.5%. Multi-factor logistic regression analysis of the risk factors related to recurrent OME showed it was strongly associate

l. Four had cardiac arrhythmias. Clinical presentation included fever and cough. Conclusion: COVID-19 can be fatal in paediatric patients, especially in those with a chronic medical condition.

aged successfully by use of snares and additional overlapping stents, the first of this type in literature. Awareness of factors leading to stent migration and techniques involved in repositioning these stents may help cardiologists who deal with these interventions. <Learning objective: Dis

saemia major (betaTM) and sickle cell disease (SCD). Methods: 40 betaTM and 20 transfusion-dependant SCD patients were included along with 60 healthy age and sex-matched controls. Echocardiography and Tissue Doppler Imaging were performed for all subjects as well as the contro

drugs. Owing to that fact, it is a condition that is outgrown with age and also could cause embarrassment. As antiepileptic drugs sometimes need to be taken for a long duration until epilepsy relapses, the occurrence of enuresis in this situation can be troublesome for both the child and

nce of 3 cm was prepared. The tag was delivered adjacent to a 7-mm subsolid nodule in the right lower lobe of a patient under cone beam computed tomography guidance and video-assisted thoracoscopic surgery wedge resection was subsequently performed. Results: The delivery of th

d functional data (spirometry) and performed an EIB test. We used item 4 of the GINA questionnaire regarding exercise-induced symptoms to assess the diagnostic power of this instrument. RESULTS: We included 40 patients (17 with MMA and 23 with STRA) with a mean age of 11.3 yea

nity Therapy process was adapted to fit the pediatric population and their families. Modifications are explained in some detail, and specific cases are shared to illustrate the process. The goal of this case series is to report on the application of Dignity Therapy to the pediatric population.

e. Methods: Low-income families (N = 270), recruited when children were approximately 6-years-old (wave 1), were followed for 2 years (wave 2). Socioeconomic and parenting factors were assessed at W1 via parent-report. Associations of poverty and FI with two measures of mealtime

nit at our institution following a quality improvement project that implemented an SRS algorithm. We compared exclusive breastfeeding rates over 2 time periods, 33 months before and 15 months after SRS algorithm implementation. We completed bivariate comparisons using chi-squa

tools were developed using samples from European/Caucasian women. Hispanic/Latino describes an admixed group of individuals with different fractions of European, African, and Indigenous American ancestries. We determined the frequency of the different intrinsic subtypes of BC in

›ne bodies as a major energy source is an evolutionary conserved adaptation to food deprivation that permits the survival of normal cells during extreme shifts in nutritional environment. Only those cells with a flexible genome, honed through millions of years of environmental forcing a

odies as a major energy source is an evolutionary conserved adaptation to food deprivation that permits the survival of normal cells during extreme shifts in nutritional environment. Only those cells with a flexible genome and normal mitochondria can effectively transition from one ene

largely dependent on glycolysis for energy, normal neurons and glia readily transition to ketone bodies (beta-hydroxybutyrate) for energy in vivo when glucose levels are reduced. The bioenergetic transition from glucose to ketone bodies metabolically targets brain tumors through integ

dicated a significant positive correlation between "honesty" with "job and carrier satisfaction" and "forgiveness" with "job and carrier satisfaction". In addition, findings showed a significant positive correlation between "honesty" and "control at work" and between "accountability" with

ective mode as compared to Sanger sequencing in large genes. We used a targeted resequencing based approach to identify pathogenic variants in eight cases in seven families. These variants were validated using Sanger sequencing in patients as well as family members and were predic

these ADCs were extracted from 47 diffusion weighted magnetic resonance imaging (DW-MRI) scans at 11 different b-values (b0, b50, b100, ..., b1000 s/mm(2)), while the R2* values were extracted from 30 blood oxygen level-dependent MRI (BOLD-MRI) scans at 5 different echo times (

HXP (50 mg/kg/day) or saline by gavage for 2 weeks. At 2 weeks post MI, we found that HXP significantly enhanced myocardial function and attenuated the increase of heart weight index (HWI) and pathological changes in MI mice. RNA-sequencing and KEGG pathway analyses identified

centre over 14 years. Kaplan-Meier curves evaluated patient characteristics at diagnosis with time to initiation of biologic therapy. A Cox proportional hazards model was used for multivariate characteristic analysis. Results: A total of 198 patients were included, 57.6% had Crohn's diseas

of cyst with uneventful post-operative days. This case has been reported as it is a rare condition.

om June 2019 to November 2019 after obtaining ethical approval from the institutional review committee (Ref no. 150320199). Convenient sampling method was applied. The mothers of children between 6 to 24 months were interviewed using a structured questionnaire to ascertain th

notion capture recorded lower extremity muscle activity and kinematics of 22 healthy full-term infants (4.2+/-1.6 months, 13M/9F) during five conditions: Pavlik harness, Rhino brace, inward-facing soft-structured baby carrier, held in arms facing inwards, and a standard car seat. Mean f

the control group were compared in the study. RESULTS: There were 35 subjects in the prophylactic paracetamol group (PPG), and 40 in the control group. Primary outcome measures were ductal closure, ductal diameter, grade 3-4 IVH, and repeated ibuprofen need for the treatment c

report and biomarkers of exposure. Identifying and control of pre- and postnatal factors that impact development are difficult and dependent on appropriate research design and selection of comparison groups and measurement of confounding, mediating, and moderating variables. Pc

nces of two doses of 0.02 mg/kg epinephrine separated by minimum one-minute, then a rescue dose of 0.4 U/kg vasopressin followed by minimum two-minutes. Invasive measurements were used to evaluate and compare the hemodynamic and neurologic effects of each vasopressor dc

spective review of activity associated with the QTS and summarized key activities which have arisen from this extensive program of work. Telehealth service records and associated publications were used to describe the evolution of the QTS over a 15-year period. From November 2000 t

nd R356W) reported as frequently occurring in other populations. Results: Fourteen patients had homozygous mutations; six patients were compound heterozygotes as determined by investigating parents of the patients, one patient had a large gene deletion which was previously repo

nalysis (PRISMA) extension to scoping reviews. A systematic search of literature databases (Medline, EMBASE, CINAHL, PsycInfo and ERIC) was conducted from inception until June 12, 2020. Two reviewers independently assessed full-text articles according to predefined criteria. Interven

mental KD on a mouse model of glioma, and compared patterns of gene expression in tumors vs. normal brain from animals fed either a KD or a standard diet. RESULTS: Animals received intracranial injections of bioluminescent GL261-luc cells and tumor growth was followed in vivo. KD

JIPS) tool. Results: The literature search yielded 4960 unique records. We included nine studies evaluating the association between socioeconomic status and risk of and/or outcomes after in-hospital cardiac arrest. All studies were observational cohort studies, of which seven were from

nfection was found, demonstrating the effectiveness of COVID-19 screening process.

ted surgeries were extracted and descriptive statistics reported. Results: A total of 11 children were diagnosed with FAP over the study period with a mean age at diagnosis of 6.3 +/- 3.2 years with 72.3% males and median follow-up of 4.8 years. The mean age at first gastroscopy was 10

lmitted from 2013 to 2015 who were diagnosed with a UTI and received treatment. Multivariable logistic regression assessed independent factors associated with our outcomes. Results: Of 568 diagnosed UTIs, 88.6% received empiric TGC treatment. Empiric coverage among cultured or

dents.

ta-analyses of Stages 1 and 2, we identified 59 loci (p value <5e-8), including nine novel BP loci. The novel associations were observed mostly with pulse pressure, with fewer observed with mean arterial pressure. Five novel loci were identified in African ancestry, and all but one showed

RH information topics and sources by reported sexual orientation (completely heterosexual with no same-sex partners [reference]; completely heterosexual with same-sex partners; mostly heterosexual; bisexual; lesbian). Results: Compared to the referent, most sexual minority subgrou

electronic health record's dedicated FHH module; and (3) Providers desire feedback from genetic services consultation and are uncertain how to refer patients to genetic services. Conclusion: Study findings highlight the varying degrees of engagement that providers have with collecting

ne source of infection was unclear in 14 patients with COVID-19 who were clinically unlinked to clusters #1 or #2. These patients, who had no clear history of infectious contact within the hospital ("undetermined source of infection"), had haplotypes similar to those in cluster #2 but did r

3.11). Patients were residents of nine different states of India. Clinical presentation varied from acute encephalitis followed by neuroregression and chronic/insidious developmental delay. Neurological sequelae varied from asymptomatic (no sequelae, 2 patients) to moderate (5 patients

ts: MIGLIS was associated with a lower sperm recovery rate and fewer injected sperm compared with DGC. However, the overall pregnancy rates following MIGLIS and DGC were similar (MIGLIS 8.8%, DGC 9.3%). In a subanalysis according to age, the pregnancy rate was higher for MIGLIS

technology acceptance model (TAM) was used to guide the app usability evaluation. App design utilized an iterative process involving providers and potential users who were parents and adolescents. App features include a vaccine-tracking function, a discussion forum, and stories with

ed to better understand the role of diet in the treatment of epilepsy and other disease.

patients in a small group informational session. Prior to the experience, participants completed the Jefferson Scale of Physician Empathy-Student (JSPE-S) version. After the experience, students completed a post-test JSPE-S questionnaire, and a final post-post-test JSPE-S questionnaire

efit from eosinophil/mast cell-directed therapy. Methods: Esophageal biopsies from SSc patients and healthy participants were stained for tryptase, a mast cell marker, and associations with relevant clinical parameters including 24h esophageal pH testing were assessed. Intra-epithelial r

MRI homogenization that also provides the segmentation map of an anatomical region of interest. Homogenization is achieved using an unsupervised architecture based on variational autoencoder with cycle generative adversarial networks, which learns a common space (i.e. a represe

ed with left pulmonary sling with tracheal stenosis. We discussed the diagnosis and management of pulmonary artery sling and present the successful use of laryngeal mask in difficult airway management.

m, Breastfeeding Self-Efficacy Scale-Short Form (BSES-SF), and LATCH Breastfeeding Assessment Tool were used for data collection. RESULTS: The mothers in the risky age group had a lower breastfeeding rate when their babies were one month old and a higher formula feeding rate whe

ly employed. Development of treatment protocols specific to children with ABI that combine both nonpharmacologic and pharmacologic strategies will reduce the risk of reliance on treatment strategies that are less preferred and optimize care for this population.

th post hoc multiple comparison procedures and Chi-square test were used for statistical analysis of the data. Complete blood count, distribution of the lymphocyte subsets, and plasma levels of markers of vascular damage were analyzed. Results: A significant decrease in the number of

fore and after surgery (at a follow up of 1 month, 6 months, 2 year and 5 years), intraoperative findings and postoperative outcomes were recorded. A total of 36 (5.8%) pediatric cholesteatoma patients underwent Modified Bondy procedure. Out of these, 5-year follow up was available

hods: A cross-sectional multi-center questionnaire based quantitative study of 154 parents/caregivers of children under ten years undertaken in private and public health facilities. Results: The use of self-medication was reported to be 77.9%. Among these parents/caregivers, 50.8% usec

years, diagnosed with ADHD (n = 56) were included, and details of the total duration of screen exposure, maximum continuous screen exposure time, and types of screen-based devices used, reasons for screen exposure were collected from primary caregivers. ADHD symptom severity v

yield from these first-tier tests in the 7 years before (2006 to 2012) and after (2013 to 2019) implementation at the community paediatrician level. Results: Prior and postimplementation diagnostic yield of an IEM from first-tier metabolic testing was 9 out of 986 (0.91%) and 11 out of 4,3

Western Blot. RESULTS: Enzymes necessary for ketone catabolism (BDH1, OXCT1 and ACAT1) are significantly downregulated in adult and pediatric GBM. GSC mitochondrial ultrastructure suggested defects in oxidative phosphorylation. Treatment of both GBM and GSC cell lines resulte

morbid depression and anxiety are present. SSRDs offer the opportunity to explore the role of SLI in relation to mental distress, including trauma, MUS, chronic medical conditions and comorbid mental disorder. AIM: We hypothesized that increased IL-6 and hsCRP may be directly linkec

y explores the safety and feasibility of the KD in children with recurrent DIPG and no remaining treatment options. Safety was defined as the number of adverse effects. Feasibility was defined as the number of patients who were able to use the KD for three months. Coping of patients a

en with COVID-19 is multifactorial. Clinical suspicion of COVID should be heightened in patients who present with unexplainedly severe DKA.

ry: The role of KD in reducing seizure activity in some mitochondriopathies and its efficacy in pyruvate dehydrogenase deficiency is known. Recently, few cases suggest the potentiality of KD in decreasing paroxysmal activity in children affected by AHC. A few data support its potential us

blem-solving and parent-training interventions involving more than 800 children and families with diverse diagnoses, including traumatic brain injury, epilepsy, brain tumors, congenital heart disease, and perinatal stroke. We summarize efficacy across studies and clinical populations anc

ERCC gene polymorphism and overall survival of osteosarcoma. The meta-analysis indicated that ERCC1 rs3212986 (8092 C>A) gene polymorphism, ERCC1 rs11615 (19007 T>C) gene polymorphism, ERCC2 rs1799793 (A>G) gene polymorphism, ERCC2 rs13181 (Lys751Gln) gene polymorp

ggressive carcinogenesis followed de-differentiation and hyperproliferation. In double mutant endometrial cancer, add-back of either induced senescence. Biochemically, residual cBAF subcomplexes resulting from loss of ARID1 scaffolding were unexpectedly found to disrupt polybromo

lor CVD and not on statins at baseline (n = 262,923), stratified by PCE-eligible (n = 131,721) or PCE-ineligible patients based on missing or out-of-range variables. We trained ML models [logistic regression with L2 penalty and L1 lasso penalty, random forest, gradient boosting machine (GB

with high-risk pre-B cell acute lymphoblastic leukemia diagnosed with pulmonary aspergillosis. She developed a hypersensitivity reaction to micafungin, which was deemed first-line therapy for the infection. A rapid intravenous desensitization protocol was successfully completed withou

en with a focus on the peculiarities of diagnostic investigation and management of the disease in this age group. The study includes a case report of the disease in a child. STATE OF KNOWLEDGE: In children, the most common factors causing HP are avian and fungal antigens present in th

rity-, age-, and body mass index (BMI)-matched ovulatory unexplained infertile control patients from the Assessment of Multiple Intrauterine Gestations from Ovarian Stimulation (AMIGOS) trial were obtained and used for this study. Interventions: GnRHR AAb activity was determined w

ey enzyme in one-carbon metabolism. Inhibition of SHMT2 by either knockdown or pharmacological compounds induced anti-BL effects in vitro and in vivo. Mechanistically, SHMT2 inhibition led to a significant reduction of intracellular glycine and formate levels, which inhibited the mT

ed intravascular coagulation. We present a case of an African American male with severe hemophilia A and history of factor VIII inhibitor, maintained on emicizumab prophylaxis, who developed rhabdomyolysis with a symptomatic hyperCKemia. To date, there is no known link between r

nt with metabolic dysregulation. Literature primarily from the last two years is reviewed here, examining the impact of sleep and circadian rhythms and their disruption on the gut microbiome in human and non-human models, with an emphasis on the hypothesis that the altered gut mi

n the community. Although anxiety and fear are encouraging to take necessary precautions, exaggeration of these emotions may cause greater health problems.

tatic enzyme levels slowly returned to normal within 6 months. To the best of our knowledge, this is the first case of myocarditis/cardiomyopathy treated with eculizumab in STEC-HUS. This case illustrates the need for vigilance regarding myocardial involvement and eculizumab-induced

al study was conducted in rural areas of a province in Southwestern China from July 2017 to January 2018. We randomly selected 370 VCs who practiced Western medicine and assumed the main responsibility of providing medical services in his/her clinic. A standardized clinical vignette

alth Centers for any reason. Data were collected between February 2016 and June 2017 using a descriptive questionnaire, a nutrient consumption frequency form, the Rome III diagnostic criteria form, and the Trait Anxiety Scale for Children. RESULTS: After the training program, the mea

iatric Assembly of the South African Thoracic Society and the National Institute for Communicable Diseases established seven expert subgroups to revise existing South African guidelines focusing on: (i) epidemiology; (ii) aetiology; (iii) diagnosis; (iv) antibiotic management and supportiv

ve delivery of (64)Cu-CuNCs to pons in wildtype mice. Then the optimized FUS pressure was used to deliver radiolabeled agents in DIPG mouse. Magnetic resonance imaging (MRI)-guided FUS-induced BBTB opening was demonstrated using a low molecular weight, short-lived (68)Ga-DO

nd laser pulses were used to excite the plasmonic nanostructures, thereby generating transient pores at the outer membranes of targeted cells that enable the delivery of biomolecules via diffusion. Delivery efficiencies of >98% were achieved using the cell impermeable dye calcein (0.6

ine and sugammadex) in US children. Methods: Cross-sectional study of children with exposure to NMB agents between 2015 and 2017 in the Cerner Health Facts((R)) database, which is an electronic health record (EHR) database across 600 facilities in the United States. Logistic regressi

cytes. In this study, we found that peroxisome proliferator-activated receptor gamma coactivator-1alpha (PGC-1alpha) played an important role in inducing mitochondrial biogenesis and establishing oxidative phosphorylation (OXPHOS) during the cardiac differentiation of hiPSCs. Knock

dies for energy. This study evaluated the efficacy of KetoCal, a new nutritionally balanced high fat/low carbohydrate ketogenic diet for children with epilepsy, on the growth and vascularity of a malignant mouse astrocytoma (CT-2A) and a human malignant glioma (U87-MG). METHODS:

n expression through disabling either HBG repressor genes or repressor binding sites in the HBG promoter regions. In addition to these HBB mutation-independent strategies involving fetal hemoglobin (HbF) synthesis de-repression, the expanding genome editing toolkit is providing incr

d twelve months. Statistical analysis was presented as intended to treat analysis. Categorical data were analyzed using Fisher's exact test. The significance level was set at p </= 0.05.

30 years of age, and 50 (35.2%) were pediatric cases. Male to female ratio was 1.84:1. The commonest presenting complaint was blurred vision in 97 (68.3%) cases, followed by eye trau

cused analysis as well as abnormality detection in each region of the GI tract. In this paper, we compared four deep Convolutional Neural Network models for feature extra

d the double-disc synergy test were used to investigate antibiotic susceptibility and presence of ESBL production. Results: UTI was confirmed using a positive urine culture for a relevant pathogen in 96/2

onnaire by the subjects' parents/legal custodians and physicians and then transferred to the PROVE/CHD Registry. Results: The PROVE/CHD registry encompasses 1252 patients wi

of the child's final digital story. Children and parents completed the Parent-Adolescent Communication Scale pre- (T1) and post-intervention (T2). Linear regressions tested for differences in cha

luated as outlined by Sheiner and Beal. Measured and estimated BSA values were compared using the Eksborg plot. Results: The estimated BSA values from all three equations and the BSA values

ere included. Overall, we rated four studies as having a low risk of bias, eight studies as moderate and one study as high risk of bias. In a meta-analysis of nine studies, comprising 280 patients, th

blishing the diagnosis and dealing with complications. Antifungal medication was the cornerstone of treatment in all our patients. Three of our patients were exceptional with complications such a

nically distributed to all pediatrics residents. Mean knowledge scores and 95% confidence intervals (CI) were calculated for each independent variable to test for asso

ic asthma and, as a result of incorrect treatment, unnecessarily expose patients to steroids and other therapies for extended periods. Furthermore, we seek to alert healthcare providers t

ugs with a frequency of 512 (as 1 drug may belong to more than 1 off-label category). The most common reason for off-label prescribing was related to doses that were

values, especially where muscle biopsy and EMG are not available as is the case in most centers in developing countries, would have resulted in missed diagnosis using Bohan and Peter criteria.

echnique that can be utilized to address this abnormality, along with the postoperative results.

emoglobinopathies to postsecondary accessibility offices. This article will describe the development of the intervention and present key findings from qualitative interviews with patients (ages 18-19

n two (52.2%) parents obtained high knowledge score about halitosis. Parents' knowledge was significantly associated with the mean age of the children (P = 0.02). Thirty-ni

was performed in patients with sensorineural HI to exclude additional genetic etiologies. Results: HI was found in 19 patients (28.7%). Among them, 5 (7.6%) had moderate to severe bilateral sensor

t (i.e., the nonbonded group). In each group, the microTBS was examined after 24 h (n = 20) and after a 3-month aging period (n = 20). Five other caries-free extracted premolars were used

wever, the lowest scores were the human and technical resources (1.2), attitudes toward CM prevention (3.3), will to address the problem, and material resources (3.8), current programs implement

istructure. For the remaining uncovered demand, the second step of the optimization model suggests the acquisition of additional equipment for the elected medical centers to mee

ises. The highest percentage of preventable deaths occurred in children aged 13-18 years (39.3%), followed by the age group of 6-12 years (32.4%) and the age group of 29 days to 5 yea

ı (95.4%) in our sample reported using electronic devices, according to parents. No association was found between using electronic devices and having a RE (P = 0.26). Doing outdoor a

: frictional force of each arch wire material was measured using the universal Instron machine. The surface topography was evaluated using a noncontact profilometer machine. Re

sionality of the enteral feeding barriers scale. Results: A total of 223 PICU healthcare workers from various intensive care settings responded to the survey. The top-th

sis; 25.2% were transferred from hemodialysis. Peritonitis incidence was 0.6 episodes/patient-year. Gram-positive and -negative organisms were identified in 50.1% and 22% episodes, respec

es related to glucose metabolism. Mechanistically, we demonstrated that BMP4 reduced the hepatic glucose levels through the activation of mTORC2 signaling pathway in vitro and in vivo. Collectiv

The isolation rate of MTB from neutralized and non-neutralized GA samples by combined LJ and MGIT 960 culture was 7.1% (24/336) and 6.8% (23/336), respectively. Both of them detected 18 MTB isol

program. Twelve parents completed the survey. Setting/subjects: Tertiary care children's hospital. The subjects were bereaved parents. Measurements: Five-question survey. Institutional Rev

reatment was applied in 55% of cases, which is contrary to previous researches. The highest incidence of femur fractures was recorded in the 0- to 4-year age groups, accounting for 49.1%

and phase 3 the follow-up audit. Data were collected through observation, interview and resource inventory. RESULTS: The postintervention compliance report showed maximum improve

: age group.

the pooled sample, significant interactions were found between race and parental education suggesting that the effect of parental education on left thalamus volume is significantly smaller fo

ndic-gland polyps (FGPs) from non-FGPs. Results: An isolated lacy vessel pattern and a homogenous absence of surface pattern successfully differentiated low-risk from high-risk gastric po

'failure to meet basic needs," "the personal and interpersonal aspects of caring" and "safety in caring context." CONCLUSION: To ensure high-quality care, it is important to understand

m-chain fatty acids rather than ketones are likely to block seizure onset and raise seizure threshold. The mechanisms underlying the ketogenic diet might also have roles in other disorders, su

ers of newly described taste bud networks analyzed by Stereological methods. Results compared with Mann-Whitney U test P < 0.005 considered as significant. Results: The mean normal blood gl

highlight the importance to ensure timely diagnosis of DHS and its appropriate management. Patients started on dapsone for various clinical conditions need to be observed ca

ons of CPR quality. Results: Thirteen simulations were performed; 5 were randomized to include a CPR Coach. There was a significantly shorter duration to backboard placement in the intervention group

for children undergoing cardiac surgery based on the unit type (dedicated CICU or mixed unit).

l: 1) sources of sexual health information; 2) sexual health information needs; and 3) preferences for sexual health information delivery. Participants discussed and critiqued the Internet, other mass media,

l whether the arrest was witnessed. Odds ratio confidence intervals were calculated using the Wald method, and corresponding p-values were obtained with the likelihood ratio chi

ce setting. Methods: Quantitative data on referrals made by WNs and qualitative interviews were analyzed to understand the role of WNs in ACEs screening. Results: Among famili

vas the most common conditioning regimen (n = 15, 71.4%). The median duration for neutrophil and platelet engraftment was 14 days. Cumulative incidence of acute and chronic GVHD was 19%, and 38% res

d between March 2012 and November 2019. The same inclusion/exclusion criteria were also used to screen abstracts and to perform the full-text review. Full text articles were then reviewed and w

ps. The study outcomes were operative time and catheter insertion-related complications. Results: There was no significant difference in age or gender between both groups (P = .33 and .71, res

65.43% perceived the term "Non-invasive brain therapy" as not risky, a word combination not currently being used when recruiting potential participants. Over 90% (561/622) of participants

; were greater than 0.85, and the root mean square error of approximation was less than 0.08. Cronbach's alpha was 0.96 for the entire scale, and greater than 0.80 for all sub-sc

oalloon dilatation was the treatment of choice. Hematuria ceased after the procedure, and the patient is still asymptomatic at 5-year follow-up.

:0.043), and a longer duration of insufficient blood oxygen saturation (p=0.0004). With the combination of COVID-19 and DM, hyperglycemia is persistent, without pronounced variability (MAGE - 1.5+/

of the hydrogel formulation were assessed. Results: The freshly prepared rectal formulations appeared clear, colorless, and particular-free with pH readings of 9.75 to 9.83. Over the 90 days o

f the glycosylation reaction between the glycosyl donor and the glycerol C-2-OH acceptor. The GroP C-2-chirality also played an important role in the interaction with TA recognizing anti

assigning a single process owner for UE reporting, ensuring proper ETT securement, and using 2 clinical staff during patient and/or ETT manipulation. Results: Early in the project, enhanced det

ients with MPS. The otorhinolaryngologistshould be part of the group of professionals that follows these patients to better monitor their hearing and provide early hearing rehabil

ique was used. Teeth were aged by thermo-cycling and prepared for microleakage testing. Dye penetration was evaluated and scored from 0 to 4. Data were analyzed at a significance leve

ary crescent formation. CONCLUSION: We find that immunostaining for MPO is a useful method to compare glomerular endothelial injury in C3G following eculizumab treatment. This finding also supports the notion

ths, the median length of stay was 15 days, and 18 patients required pleural drainage. The mean duration of follow-up after NP was 8.75 years. During the evaluation, none of the patients exhibited asthma

with a longer diabetes duration (P = 0.018) were more likely to report barriers to screening. Recruitment location, sex, race and ethnicity, HbA1c, insulin regimen, and clinic visit frequency

orbilliform, erythrodermic, urticarial, reticular, petechial, purpuric) in variable anatomic distribution. More thorough descriptions of dermatologic manifestations in patients with M

assigned to 2 pairs of expert raters. Raters were trained to independently score performances using the tool. Results: The reliability of the rating was adequate for the Clinical Performanc

l 1 h after (TS). Bilateral and somatic NIRS, blood gases, mean arterial pressure, and temperatures were recorded. Results: Left and right somatic NIRS values in groups at all measurements

and Gentamicin (85%) were the most commonly used first-line antibiotics for suspected neonatal sepsis. Most neonates (87%) did not receive a second-line antibiotic. Cefotaxim

ranked genes were evaluated in human induced pluripotent stem cell-derived cardiomyocytes, and variant enrichment was confirmed in a replication cohort. Using random forest, a risk

e 29 (39.7). Pain (75.3%) was the most common symptom at presentation. Nine patients had incomplete details and were excluded from the analysis. Among 21 (28.8%) patients, the lung (61.9%) wa

d with HH may respond favorably to the KD, and that ketone bodies might directly modulate the intrinsic epileptogenicity of HH tissue.

isolates were done by Kirby-Bauer disc diffusion method. Data entry was done in Statistical Packages for the Social Sciences version 20. RESULTS: Of the 841 specimens (blood, cerebros

tional effect of the variants. Remarkably, the genetic diagnosis had a direct impact on clinical management. Most relevant consequences were therapy related such as initiation of th

th an evaluable day 15 bone marrow had M3 status. At the end of induction, 30 (12%), 127 (50%) and 98 (38%) patients belonged to the standard-risk, intermediate-risk and high-risk (HR) groups,

: The primary cell cultures were obtained from the tumor biopsy samples of the patients with AA (n=5), GB (n=7) or MB (n=25) prior to radiotherapy and chemotherapy. The cytotoxicity effect of N

at factors make students vulnerable to suicide, and suggestions for future research. We conclude with a discussion of options to increase quantity and quality of service provision o

ormed of patients with TSC treated with the ketogenic diet between January 2002 and May 2007 at Massachusetts General Hospital. RESULTS: Five patients with definite TSC underwent serial imagin

; post-transduction before a significant decline between 15 and 19 weeks post-transduction. More importantly, even after its decline, most animals maintained detectable luciferase expres

ulatory, and many die during early childhood. Less severely affected individuals with moderate cognitive impairment and long-term survival have been reported. DIAGNOSIS/TESTING: Isolated

ogether with active signaling pathways, may be targeted by therapeutic agents. Bridging research and clinical practice can accelerate the testing of novel treatments in clin

these websites. The Health on the Net Code of Conduct (HONcode) was used to assess the quality of information, by taking ethical principles into account, with values ranging f

failure as an outcome, and had a Level of Evidence grade of I to III. Qualitative review and quantitative meta-analysis were performed to compare graft failure rates. A random-effect

f staff agreed that the use of videoconferencing improved participation of the PICU team and consultants by increasing access to rounds. Over 50% of staff agreed that the use of vi

ıssociated with HFNC failure. Results: Two hundred eight patients met inclusion criteria, of which 61 (29.33%) failed HFNC. Risk factors for HFNC failure included younger age (

he ketogenic diet, but no alteration in PI3 kinase activity was observed. These findings indicate that while the ketogenic diet may be effective in inhibiting growth of other tumor types, it d

8 nm, 100 mW, and 0.8 J/cm(2) for 8 seconds at each point. After this therapy, an increase in salivary flow, remission of the xerostomia, and an improvement in mastication and swallowing were

n shows strong cellular and in vivo bioactivity. This enzymatic method can therefore be used for future insulin design and development.

leal for conducting downstream functional studies of ES EVs. Absolute quantification of the molecular hallmark of ES (i.e., EWS rearrangements) using reverse transcription D

:icipation; among others. Parent collaboration throughout implementation promoted partnership. Conclusion: Utilizing a variety of unique and innovative approaches to promote PFCC strategies

liovascular disease. We describe the current regulatory context, the key stakeholders involved, and present specific, guideline-inspired initiatives to be considered by both Congress an

/s, estimated glomerular filtration rate (eGFR) returned to baseline value and remained stable without overt proteinuria after follow-up. Six out of eight transplanted patients responded to ec

and rightward asymmetries within the same language-related regions. Permutation testing demonstrated that age and sex effects were most consistent and pronounced in the superior temporal sulcus,

ed PDA characteristics, as well as left ventricular diastolic function and markers of pulmonary overcirculation, and from this formulated a PDAsc. Gestation was also incorporated into the score.

ately 2-weeks postpartum. PCB congeners 138 + 163, 132 + 153, 180, and the sum were measured using gas chromatography/mass spectrometry and adjusted for milk fat content. Infant growth was monitored

abetic patients showed RV and LV systo-diastolic dysfunction compared to controls, the best predictor of LV systolic dysfunction was CT-1 (sensitivity: 69%, while IGFBP-7 was found to

gnificant differences between each pair of groups. Results: The study found that sitting FEV1/FVC is significantly lower in overweight/obese asthmatic children compared to normal weigh

ND), ND + PTZ, ND + PTZ + LAB, KD, KD + PTZ, and KD + PTZ. Based on our previous study, 4:1 KD and selected MSK 408 strain was orally gavaged (4 x 10(9) CFU/mL) with both diets for 4 weeks

a third ASD (specifically, carbamazepine). The incremental cost per quality-adjusted life year (QALY) gained was analyzed. RESULTS: Given a willingness-to-pay (WTP) of $100,000 per QAL

aware and consider fungal endocarditis in blood culture-negative endocarditis even in patients with no significant risk factor when antibiotic therapy fails.

h each period subdivided in 8 to 12 care themes. Revisions and practice changes were implemented to improve skin integrity, admission temperature, timing of initiation of feeds, re

otential etiology of cardiac arrest during anesthesia is reviewed, keys to resuscitation discussed, and an outline for the investigative work-up presented.

infections was higher in children than in adults: 2.1% versus 0.2% of the total number of pediatric and adult inpatients, respectively (p < 0.0001); 4.2% versus 0.2% of the total number of hospit

wledge of biomarkers of the inflammatory stages and those of the progression/regression. Using samples from African American and Caucasian individuals with gastritis,

l. Results: The current and cumulative prevalence of AR was higher in the adolescents (26.2-37.5%, respectively) in comparison to the children (17.9-24.9%, respectively), especially

nacrophage activation syndrome. As our case highlights, lymphoid neoplasms may follow KD.

ntrolled trials with 21,050 participants. Lifestyle and behavioural interventions more effectively reduced weight compared with no intervention, usual care, or another behavioural treatment (three reviews, lo

confirmed TB on respiratory specimens. The pooled sensitivity of Xpert MTB/RIF on stool specimens compared with bacteriologically confirmed tuberculosis with respiratory specimens was 0.50

/-shaped incision was made into the scimitar vein and directly anastomosed to the atrium. A patch of autologous pericardium was used to septate the atrium and an additional patch placed anterio

udies because they only included prenatal patients and 138 because they were case reports with </= 3 patients. STUDY APPRAISAL: Seventy-six eligible studies were examined for study type, size, inte

nt challenge for clinicians and researchers. METHOD: During the first year of life, we tracked the onset of infantile spasms, the type and timing of antiepileptic treatments, and the associa

luding recent and current clinical trials with U.S. Food and Drug Administration approved and nonapproved medications, case reports, case series, and reviews. The authors also reviewed

(83.3%) were born out of consanguineous parentage. All of them had visual impairment and pyramidal signs. Seizures were noted in five (42%) children. Normal head size in three (25%) and microcephaly

RI brain. Of the 18 children, only nine are alive. Duration of illness among deceased children was 9.6667 months (range = 2-16 months). Waxing and waning of symptoms were seen in seven cas

, 288 pediatric patients (mean age, 14 years; 61% female) were identified. Within the first year of diagnosis, 188 patients (65.3%) did not receive any DMT. The most common first-initiated treatme

appropriate ETT size, comparing it with physical indices based formulae suggested ETT size so that repeated attempts on intubation can be minimized. Materials and Methods: The study gr

ally, we report a detailed description of the phantom's age-based scaling functions, information that was not previously published. Method: We developed a Python script that adapts our phantom model from FORTRAN

ngs, and complications like cytokine release syndrome (multisystem inflammatory syndrome in children). We also looked at prognostic factors and treatment modalities like corticosteroids, RN

od quality embryos and median 0.25 vs. 0.20 for intermediate quality embryos). Although on initial analysis there was a lower proportion of good quality blastocysts i

four (30%) of the pediatric patients with orbital fractures had documented periorbital and/or globe injuries at the time of presentation. The most common periorbital injury w

Results: Cohorts included 39,666 infants from Canada, 82,909 from the Philippines and 4,448 from China. For the full model including sex, birth weight and metabolomic markers, GA estimates were withi

zed to receive the IBP or standard pediatric primary care. Infants receiving the IBP demonstrated a significant decrease in the observed frequency of aggressive behaviors during infant-le

n the 3 time periods, respectively. The median age was 17 years. The percentage of perforations was 16.8%. Those patients had - with 47 compared to 27 h - a significantly prolong

between SGA-treated, SGA-naive, and control children were assessed by linear and log-linear regression models. RESULTS: SGA-treated children had greater BMI z-scores and overweight/

ation to improve knowledge, attitude, and practice (KAP) was to provide HCWs with a periodic training program regarding COVID-19. The most important source of information for HCWs on COVID-19 was

ultures, Caco-2 and organoids cultured on chips formed confluent monolayers expressing tight junctions with low permeability. Caco-2 cells-on-chip differentiated approximately 4 tim

the digital models from A-B, B-C, and C-A points. Paired analyses (Wilcoxon Signed Rank test or paired Student's t-test) were used to analyze each measurement before and after the contact

; were surveyed via anonymous e-mail responses. Results: Thirty residents participated in the study and twenty residents were surveyed and participated in a focus group. No maj

gies of hyperkalemia in the PICU patient are discussed and previous reports of hyperkalemia associated with trimethoprim-sulfamethoxazole presented.

netics in piglets showed a central gray nuclei-to-cortex ratio similar to human infants with a steep wash-in that crossed and remained above the 1.0 threshold for most of the

neurodegenerative disorders including Alzheimer's disease. C7-8 triglycerides or fatty acid esters are under development as medicines replacing KD.

(b) desperation, mercy, and guilt, (c) exhaustion, and (d) longing; 2) coping; 3) professional attributions; 4) meanings related to life; and 5) unmet requirements and suggestions. CO

ar-old woman with history of CML who presented with acute biphenotypic blast crisis with extramedullary involvement in the context of aggressive chemotherapy. Literature

1042 hospitalized patients infected with SARS-CoV-2 and 95 different parameters. Total lymphocyte count and levels of CD3+ and CD4+ T cells were decreased in severe and critical cases. Neutrophilia was f

: 0; 95% CI: -0.07 to 0.06; p = 0.87), FEF25 (Std. MD = -1.69; 95% CI: -6.69 to 3.31; p = 0.51), FEF50 (Std. MD = 0.10; 95% CI: -0.12 to 0.33; p = 0.37), FEF75 (Std. MD = 0.01; 95% CI: -0.21 to 0.24; p =

ıalized with a follow-up audit to determine a change in practice. RESULTS: Significant improvements in the follow-up audit in comparison with the baseline audit were as follows: evaluation of pa

ere were significantly lower Apgar score findings in newborns with a tight nuchal cord compared to ones with a loose nuchal cord (P <.001). Pathological cardiotocographic findings were monito

ring day shift than in nurses who were aged 41 years and older and working in the night shift (p<0.05). CONCLUSION: The job satisfaction of nurses working in pediatric clinics was moderate and af

r failure of two to three drugs for lack of efficacy. Refractory cases should be referred for an epilepsy surgery workup. Dietary treatments and neurostimulation may be consider

nitine palmitoyltransferase I (CPT1) inhibition, with the CPT1 inhibitor etomoxir, on C8 and C10 beta-oxidation were also investigated. RESULTS: Both fatty acids were catabolized, a

d by IBD followed by infectious colitis (50.9%, 38.1% and 6.3% respectively). Faecal calprotectin above 744 microg/g could predict IBD as a cause of chronic colitis with 86.8

piratory volume in 1 second (FEV1), FEV1/FVC ratio, maximum expiratory flow at the point of 25% loop flow-volume (MEF 25). Results: Among the children examined, taking into acc

oving correlation coefficient between mean arterial pressure (MAP) and ICP, and between MAP and CBF, respectively. Burden of impaired CAR was the area under the PRx or CBFx curve using a thr

es for gene editing are expressed in more mature blood cell lineages but not in the hematopoietic stem cells (HSCs), where they are tightly packed in heterochromatin, ma

mal age groups for the immunological control of the risks of developing type 1 diabetes in healthy siblings were determined. The highest risks were noted with the combination of GADA

They may have particular value for countries in Asia. Side effects include constipation, dyslipidemia, growth slowing, acidosis, and kidney stones. Additionally, neurologists

oom approach. CONCLUSIONS: The study results suggest augmented reality simulation to be valuable in teaching pediatric asthma management content in graduate nursing education.

e level of periostin in the nasal secretion: up to 0.84 [0.06; 48.79] ng/mg, whereas in remission, that was 0.13 [0.00; 0.36] ng/mg; p=0.04. This value increased progressively as the severi

Of these, 150 were injected with and 150 without infrared assistance. A success rate of 72%/51.3% was noted with and without infrared assistance, respectively, on the first attem

tes (SD 20.75 min), with a median and mode of 20 minutes and 60 minutes respectively.On logistic regression analysis, yoga/ meditation, which have been recognized as effective relaxation

)-19 manifestations. Dermatologists play a key role in diagnosing patients with COVID-19 who may present for the first time unwittingly exhibiting early signs of COVID-19. We h

The care pathways reveal that most families visited hospitals as the first service and that visits to other health services were frequent. Well over half (63.3%) of the familie

thetic (including monitoring and warming measures used), and operating theater (OT) temperature. We performed statistical analysis to identify risk factors associated with hypothermia. Result

prevention, risk prediction, and surveillance of cardiac diseases in survivors of childhood cancer.

to occur during follow-up.

her speculate that dysfunctional outer blood-retinal barrier may play a role in the pathophysiology of MC development in CORD.

M. Settings included participant homes, the medical campus of the palliative care program, and a community art gallery. Measurements: Participant meaning and purpose were assessed thr

ıced the abnormality of mitophagy and mitochondrial transfer also contributed to the fibrotic process. Therefore, with gaining the increasing knowledge of mitochondrial structure, function,

ersity is not changed significantly during the diet, but differences in both taxonomic and functional composition are detected. Relative abundance of bifidobacteria as well as E.

the calendar year of birth, maternal age, and parity. Results: Overall SMM rate among migrant women with insecure residency status was 21.5/1000 and 14.7/1000 among Swedish-born women. Compared

with real pediatric patients; the best regarded aspect of it was utility (93%). Differences were found between pre- and post-training scores: 2.72 +/- 0.84 versus 4.60 +/- 0

. A generalized linear model was used to determine the effect of image guidance on inflation-adjusted total cost and LOS. Results A total of 1,297 cases of TSPR were included

measures, and oral microbiota were identified using next generation sequencing (HOMINGS, Forsyth Institute, Cambridge, MA). Results: With the use of multi-dimensional scaling p

of incidents involved a previously-granted or sought PO, and none of which had explicitly mentioned firearm removal as a part of the PO. Conclusions: The majority of victims were killed w

of the proposed tests and their superiority over existing methods. The testing procedures are also illustrated by analyzing a data set of a metabolomics study that in

ico, North Dakota, Oklahoma, Rhode Island, South Carolina, South Dakota, Vermont, West Virginia, Wyoming. The Environmental influences on Child Health Outcomes (ECHO) research program's IDeA State

ations in the pediatric population affected by Severe Acute Respiratory Syndrome Coronavirus 2.

ng initiated and the child discharged after she started gaining weight. Early post-operation complication (anastomotic stricture) was noticed after 2 weeks; however, corrected with endoscopic ba

SRNS groups (p = 0.286). Concerning total biopsied cases and MCD (proven plus presumed) cases, no significant differences were found between those with positive and negative HpSA

ndred and three participants, including ophthalmologists, specialists/registrars, and optometrists from across Saudi Arabia, participated in this survey. Approximately 25% were classified as Group A. La

e care unit and managed with supportive care for 6 days where she died on the 6th day. Vital signs are simple but important and if overlooked can lead to a series of complicated ev

tomizable, and simple way to correct auricular deformities. Primary physicians/paediatricians should embed moulding into their practice, starting treatment as early as possible in the

utpatient opioid requirements for 102 patients (84.3%). For 15 (12.4%) patients, the algorithm over-estimated opioid need by an average of 0.38 OME/kg. Four (3.3%) patients required addit

notherapy, and corticosteroids. For this reason a restrictive calorie ketogenic diet, similar to that used in children to control drug resistant seizure activity, has been advanced as an alternat

d.

; with a similar age, sex, and body mass index were used for comparison. Left ventricular systolic dysfunction (LVSD) was defined as reduced 2D left ventricular ejection fractio

s for bench testing of innovative devices and lack of clinical validation in patients. The insights gained during the design, development, laboratory testing and clinical validat

C intake, total IPC sessions attended, overall IPC show rate, and IPC treatment attrition. Results: Of referred patients, 348 (84.9%) were encouraged to or scheduled an intake. Of thos

:re. Degree of facial paralysis before and after surgical intervention was measured using House-Brackmann scores. Results: Nine patients with a mean age of 8.6 (range: 5-15 years) years at time

n for over 28 days. Further, insulin can be easily recovered by dilution of these hydrogels for administration at the point of care. This supramolecular hydrogel system shows pr

ıeral T-cell lymphoma not otherwise specified is a rare subtype of PTLD and its association with EBV is even more rare. A few patients can achieve complete remission with standard chemothe

er a 16-year period (2004-2020), were included. Digital medical records, including ultrasound studies, were reviewed by a team of pediatric nephrologists. RESULTS: Thirteen patients were

: virus spreads through the brain tissue into the piriform cortex, basal ganglia, midbrain, and hypothalamus. Later, the substantia nigra of the midbrain, amygdala, hippocampus, and cerebellum be

an overall survival time was 16.9 months (95% Confidence Interval, 5.2-32.9 months) with an estimated 2- and 5-year overall survival of 41.9% +/- 9.6 and 27.9% +/- 9.2, respectively. Pat

:ing restrictive by 62% (138/219) of the respondents with 37% (80/216) reporting being able to visit less often than desired, 41% (78/191) reporting being unable to b

d by a subject-matter expert editor. Articles scoring higher than 99 percentiles by category-type were selected for full critique. Systematic differences between editors' and rev

ıs on its mucocutaneous manifestations.

with frequency and proportion for binary data. RESULTS: The prevalence of acute pediatric burns at the hospital was found to be 101 (29.71%) (24.85-34.57 at 95% Confidence Interval). The

of mental health service use. Results: Of 135 parents/caregivers interviewed, 38 (28.15%) sought mental health care from only complementary and alternative medicine (CAM) providers (tradit

ealth professionals working at the Butabika pediatric out-patient clinic while (N = 16, 44.4%) were pediatric health professionals working at the Mulago mental and neurological pediatric clinics. The m

frequent isolated bacteria. The resistance rates of E. coli isolates were increased against amikacin, ceftriaxone, ceftazidime, ciprofloxacin, cotrimoxazole and imipenem from 2005

ition 72-hour return rate. Results: The ED/UC network discharged 219,196 patients during the study, 12/2014-4/2016. The baseline 72-hour return rate was 3.5% before interventions. The te

CER1 syndrome. The patient underwent surgery followed by chemotherapy. Genetic analysis identified a germline DICER1 pathogenic variant (c.2062C>T, p.R688*)in the child and in 5 relative

lowest recorded core temperature, and perioperative complications. Patients were divided into those whose temperature decreased below 36 degrees C (Group H) and those who maintained a t

iodified Cormack-Lehane score at the trialed parameters. Vomiting and aspiration were our primary safety endpoints. Results: Eleven patients (6 women) with a mean age of 5.3 +/- 2.1 years

h a mean age of 24.3 +/- 6.5 years' old were enrolled in this study. Myocardial perfusion scan (MPS) was normal in all patients. The mean value of the measured left ventricular ejection fraction (LVEF

ind pulse oximetry (SpO2) were monitored before induction, at induction, and every 10 minutes during the surgery. In the postanesthetic care unit (PACU), pediatric anesthesia emergence delirium (

019 in the severely ill patients during the active phase and postinflammatory phase.

iificant. Conclusion A 12-month long 3:1 KD treatment exerts no deleterious effect on cardiac repolarization measures.

rint of neonates or older infants did not vary by degree of hypoxemia. There were 12 individual metabolites that differed between hypoxemic and non-hypoxemic neonates, including lower methylmalonic

e of no-touch venous grafting has not been reported. Based on the available data, routine use of arterial grafts cannot be recommended. Studies that show better pat

n other neurological diseases. But, inefficient connectivity and impaired ketogenic diet proposal limit ideal availability of this therapeutic option. Ketogenic diet in Italy is not yet

(+) T cell response in transgenic human HLA-A2 expressing mice. CD8(+) T cells from immunized mice exhibited antigen-specific proliferation and cancer cell cytotoxicity, highlighting the potential

nce [NGT]) and increasing (n = 47), and low (NGT) and flat (n = 8). There was high variability of 2hrGlu, but most patients belonged to a trajectory that increased over time. After contr

protocol for a multi-country matched cohort study designed to estimate the risk of long-term neurodevelopmental impairment (NDI), socioemotional behaviors, and economic outcomes

months. The primary outcomes were the sensitivity for ruling out pathology and the negative predictive value of the proposed criteria. Results: Of 915 total patients, 214 met the lo

or 3 months out of all DIPG patients identified. OS was estimated by the Kaplan-Meier method. Five DIPG patients (males, n = 3; median age 4.4 years; range, 2.5-15 years) meeting the

he most frequent cause of PHS were tumours (6/14), with the most representative neoplasm being neuroblastoma (4/14). Of the acquired cases (8/14), the most frequent cause was iat

from 6 weeks to 6 years. Results Total 50 patients with a wide variety of pathologies underwent analysis. Frontal sinus breach occurred in 21 patients. All were repaired by the descri

nfection with either HCV or HBV in seven cases. Of the nine patients, three underwent surgical resection and only one patient was alive at data abstraction. Conclusion: Intrahepat

temporary surgical management and prognosis.

ive Surgery. Statistical significance in response plurality was determined by nonoverlapping 99.9% confidence intervals (P < .001). Sum totals were reported as means with standard devi

of 168 encounters amongst 28 individuals were analyzed. Amongst both children and adults, ketone levels correlated with nutritional ketogenic ratio; however, the absolute ketone leve

s of cells in the pathogenesis of diseases is highlighted. Finally, the possible role of mechanosensitivity in mediating the physiotherapeutic effects is addressed.

nenting TECHS in a group, it is important to ensure participation is optional and to review expectations for confidentiality. The purpose of TECHS is to address the emotional needs of

nvironments. The focus is specifically on the choice of the optimal materials to simulate the ECM brain compartment and the biofabrication technologies needed to shape the cellular compone

el-forming formulation was used. In the group where in-situ gel forming formulation was used, re-epithelialization and normal corneal structure were observed. Conclusion: In-situ

establishment of a genetic etiology for these cases. Therefore, Silver syndrome should be included in the differential diagnosis if the initial presentation is a spastic paraparesis by urinary

S mean in lidocaine spray state, lidocaine gel state, and needle plate state was respectively 47.87, 51.31, and 49.43, which were significantly less than the control state with the VAS mean

patients. Future areas of research include new drug development, better imaging and localization techniques, and brain stimulation.

suggest clinicians are adopting these scores into their clinical workflows.

ody responses, whether measured by mean response magnitude or percent of responders. Although the mechanism(s) underlying this advantage is not yet clear, it is likely that both qu

Compared to WHO standards, Canadian children at birth had lower median WfLz (-0.73) and BMIz (-0.29), with more positive scores by 3 years (WfLz=BMIz=0.58). At both 1 and 3 years, formu

derived protein correction factor (1,000 RBCs: 0.011 g/L protein). Results: There were 437 traumatic LPs including 357 (82%) with pleocytosis and 4 (0.9%) with bacterial meningitis. Overall, f

loping PE, including those in women with CAH: chronic pyelonephritis; baseline mean AP above 95 mm Hg and diastolic AP above 80 mm Hg; body mass index over 30; family history of arterial hypertension

gold standard (n = 1,365) from the University of California, Los Angeles Health System's clinical data warehouse for patients 5 to 17 years old. Results were manually reviewed and predictive per

sub-Saharan African populations are found only in F* males of the Soliga, Irula and Kurumba may indicate a unique genetic connection between these Indian tribes and sub-Saharan

dosage regimens of VRCZ prophylaxis. Patients in the "high-dose" group received VRCZ at a dose of 9 mg/kg twice a day PO, or 8 mg/kg twice a day IV without a loading dose (children of 2-11 and adolescen

ed with adenoid hypertrophy (P < 0.0001), tonsil hypertrophy (P < 0.0001), sinusitis (P < 0.0001), posterior nostril polyps (P = 0.009), allergic rhinitis (P < 0.0001), recurr

tal stent migration is a known complication of coarctation stenting, often managed by expanding the stent in descending thoracic aorta where there are no major branches. Howev

l group. Hepatic, cardiac and pancreatic iron overload in cases were assessed by MRI T2*. Results: The mean age of our patients was 13.7 years with mean frequency of transfusion/year 12. Mean cardia

d the caretaker. Even though enuresis is proposed to have a significant effect on the child's psychology, it is still considered to be a condition that is outgrown with age. This

he tag was smooth, and the tag was almost immediately detected by the antenna. Wedge resection was successfully performed with the guiding signal from the tag. Conclusions: We have so far demonstrated

ars and a mean FEV1z-score of -0.33, of who 13 (32.5%) were classified as having uncontrolled asthma. Of the patients with uncontrolled asthma, 7 (53.8%) exhibited a decrease in the FEV1 after the EIB te

Setting/subjects: Inclusion criteria for the cases series consisted of children and their families who were aware that death may occur soon, were English spea

structure (parent-reported and observed mealtime structure at wave 1 and wave 2), mediated by parent factors (depressive symptoms, lax and overreactive parent disciplinary practices) were a

re and Mann-Whitney U tests to understand the factors contributing to exclusive breastfeeding rates. In a secondary analysis, breastfeeding rates and demographic patterns were examined using p-charts. Res

Colombia and their association with genetic ancestry and found that luminal B subtype was the most prevalent in Colombian women with BC. We found that African ancestry was associated with more aggr

nd variability selection, can transition from one energy state to another. We propose a different approach to brain cancer management that exploits the metabolic flexibility of normal c

ergy state to another. Mutations restrict genomic and metabolic flexibility thus making tumor cells more vulnerable to energy stress than normal cells. We propose an alternative approach to bra

grated anti-inflammatory, anti-angiogenic, and pro-apoptotic mechanisms. The approach focuses more on the genomic flexibility of normal cells than on the genomic defects of tumor cells a

"home-work interface." There was no significant correlation between "ethical intelligence" and "caring behaviours" and between nurses' "quality of work life" and "caring behavio

ted probably pathogenic using in-silico prediction tools. The variants include three missense (3/7 = 43%) (ITGA2B:c.1028 T > C, ITGA2B:c.1186G > A, ITGB3:c.1388G > C), two deletions (ITGA2B:c

(2ms, 7ms, 12ms, 17ms, and 22ms). The clinical sources included serum creatinine (SCr) and creatinine clearance (CrCl). First, the kidney was segmented through the RT-CAD system using a geometric de

660 differentially expressed genes and multiple enriched signaling pathways including p53 and TGF-beta. In support of these findings, HXP attenuated cardiac apoptosis and decreased p53 and

se, 27.8% had ulcerative colitis and 14.6% had IBD type unclassified. Mean follow-up time was 47.8 months. About 55.5% of the patients received a biologic medicatio

ne knowledge, attitude, and practices regarding complementary feeding. Statistical analysis was done using SPSS version 20. RESULTS: Two hundred and fifty mothers were interviewed. 151 (60.4%) mothers knew in

filtered EMG signal, time when muscles were active, and hip position (angles) were calculated. Compared to the Pavlik harness, infants exhibited similar adductor activity (but lower hamstr

of hsPDA. At the time of the evaluation by echo, hsPDA, and the diameter of the ductus higher than 1 mm were found significantly lower in the PPG (p=0.004 and p=0.013). Additionally, the repeated course o

olysubstance exposure has increased due to the number of prescribed and nonprescribed substances used by pregnant women and varying combinations of drugs may have differential effects on the outcome

ose. Results: Increases in CoPP and cerebral blood flow (CBF) were greater with vasopressin rescue than epinephrine rescue (CoPP: +8.16 [4.35, 12.06] mmHg vs. + 5.43 [1.56, 9.82] mmHg, p = 0.02; CBF: +14.58 [-0

o March 2016, 23,054 telehealth consultations were delivered for 37 pediatric clinical specialties. The most common service areas included child and youth mental health, neurology, bur

rted and the remaining patients had at least one heterozygous mutation. The following allele frequencies were observed for each mutation P30L-10%, I2G- 40%, 8bp-18.33%, I172N-3.33%, E6 cluster- 5%,

ıtions: We included review articles and primary studies that reported on stress, burnout, and depression in HCWs; that primarily focused on women; and that included the percentage or number of

treatment significantly reduced the rate of tumor growth and prolonged survival. Further, the KD reduced reactive oxygen species (ROS) production in tumor cells. Gene expression profiling d

ı the USA. Seven studies were in an adult population, while two studies were in a pediatric population. Results were overall inconsistent although some studies found a higher in-hospital

.9 +/- 2.9 years and 10.8 +/- 3.0 years at colonoscopy. Eight patients (72%) had upper gastrointestinal polyps, with adenomatous changes seen in seven of them on pathology. No patients had inva

ganisms was only 5.4% lower in FGC versus TGC. Adolescent age group (odds ratio [OR] = 8.83, 95% confidence interval [CI] = 1.47-53.11), uncircumcised males (OR = 4.52, 95% CI = 1.27-16.08),

patterns of interaction with at least one psychosocial factor. Functional annotation of the novel loci supports a major role for genes implicated in the immune response (PLCL2),

ps were more likely to receive/seek information from peers, media, and other sources (e.g., community centers). With the exception of lesbians, sexual minority subgroups were more likely to receive/s

ʒ FHH. Improving the integration of FHH into workflow, and providing decision support, as well as links and tools to help providers better utilize genetic counseling may improve p

ıot have two of the mutations used to characterize cluster #2, suggesting that these 14 cases of "undetermined source of infection" were not derived from cluster #2. Whole viral geno

;) and severe (23 patients) sequelae. All patients underwent blood tandem mass spectrometry (TMS on dried blood spots) and/or urine gas chromatography mass spectrometry (GCMS). Neuroimagi

; among women 40-41 years of age (8.6% vs. 5.9%). Peritonitis was the only recorded complication, with similar frequencies in the MIGLIS and DGC groups (MIGLIS two cases, DGC four cases). No cases became

embedded messages to promote intention to vaccinate. Parents and adolescents completed surveys before and after introducing the app in a pediatric primary care setting with low HPV vaccination rate

was completed 5 weeks later. Empathy scores were compared with a repeated measures MANOVA. The relationship between gender and empathy, and the effect of the age of the neurologi

mast cell density was quantified by semi-automated microscopy. Microarray data were utilized for functional and gene set enrichment analyses and to identify intrinsic subset (IS) assign

ntation of the optimal imaging protocol) using an unpaired image-to-image translation network. The segmentation is simultaneously generated by a supervised learning strategy. We evaluated our m

en their babies were six months old than the rates observed in case of the mothers in the non-risky age group. Mothers in the non-risky age group breastfed 14.8 times more in the first month and 5.4 times

leucocytes and granulocytes was observed in AZT group following treatment. An opposite dynamic was observed in placebo group; numbers of granulocytes significantly increased at POST interval. Al

for 31 patients and they were included in audiological analysis. Air-bone gap was maintained at preoperative levels or improve in all the patients during follow up and there were no incidences of

l modern self-medication only, 15.8% used traditional self-medication only and 33.3% used both types of self-medication. Paracetamol was the most commonly used drug in modern self-medicatio

was assessed on Conner's Abbreviated Rating Scale. Family interview for stress and coping, adapted for ADHD, was used to measure parental stress. Results: Total screen exposure time in preschool chi

345 children (0.25%), respectively. Disorders of creatine metabolism and organic acidurias were the most frequently established diagnoses in both time periods. No diagnoses were e

d in dose-dependent decreases in viability in response to glycolytic inhibitor 2-deoxy-D-glucose (2-DG), and ketone body Acetoacetate (AA), but not beta-hydroxybutyrate (betaHB). AA induced apoptosis was con

d to SLI in SSRD, and that comorbid chronic medical conditions, childhood trauma, current stress and comorbid depression and anxiety may be risk factors that account for some of the varianc

nd parents was measured with questionnaires. RESULTS: Three of 14 children referred to our hospital between 2010 and 2015 were included. Two patients completed the study, and one die

se as co-adjuvant and alternative therapeutic option for brain cancer, while any beneficial effect of KD on migraine remains unclear. KD could improve cognitive and social skills in a subs

d report feasibility and acceptability data from the perspectives of parents, children, and psychotherapists. We describe adaptation for international contexts and strategies for troub

hism were not associated with osteosarcoma risk. ERCC1 rs2298881 (C>A) gene polymorphism, ERCC1 rs3212986 (8092 C>A) gene polymorphism, ERCC1 rs11615 (19007 T>C) gene polymorphism, ERCC2 rs1799793 (Asp312Asn)

containing pBAF function. 37 of 69 mutations in the conserved scaffolding domains of ARID1 proteins observed in human cancer caused complex disassembly, partially explaining their mutation spectra. ARID1-less

3M), extreme gradient boosting] and determined 5-year ASCVD risk prediction, including with and without incorporation of additional EHR variables, and in Asian and Hispanic subgroups.

ıt reactions. The patient completed the remaining 2 months of therapy without reactions. Conclusion: This report outlines the first report of a successful desensitization to micafu

ıe home environment. Diagnosis is based on the co-occurrence of characteristic clinical presentation, radiographic and pulmonary function tests findings, and a history of exposure to a po

vith the use of the GeneBlazer cell-based fluorescence resonance energy transfer assay with and without cetrorelix, a GnRH antagonist. Main Outcome Measures: 1) GnRHR AAb activity in PCOS p

OR pathway and thereby triggered autophagic degradation of the oncogenic transcription factor TCF3. As a consequence, this led to a collapse of tonic B-cell receptor signaling, which is con

rhabdomyolysis to emicizumab. This report brings to light the possibility of symptomatic rhabdomyolysis as a potential side effect of emicizumab after moderate exertional activity.

crobiome may be one pathway by which insufficient sleep and circadian misalignment dysregulate metabolism.

hepatotoxicity in STEC-HUS.

based on national clinical practice guidelines was used to evaluate clinicians' competence in three domains: number and proportion of recommended (and essential) checklist (questions, exami

an score of the Trait Anxiety Scale for Children decreased in both intervention groups (p<0.01) and increased in the control group (p<0.05). In addition, the frequency of the consumption of mi

e therapy; (v) management in intensive care; (vi) prevention; and (vii) considerations in HIV-infected or HIVexposed, uninfected (HEU) children. Each subgroup reviewed the published evidence in their

TA-ECL1i radiotracer and PET/CT before and after treatment. We then compared the delivery efficiency of (64)Cu-CuNCs to DIPG tumor with and without FUS treatment and demonstrated the FUS-enhanced

kDa) as a model payload, while maintaining cell viabilities at >98%. The highly efficient intracellular delivery approach demonstrated in this work will facilitate translational studies ta

ion estimated factors associated with the use of sugammadex vs neostigmine. Results: A total of 27 094 pediatric clinical encounters were exposed to neuromuscular blocking agents (NMBAs), in which 21 8

ing down PGC-1alpha by siRNA impaired mitochondrial respiration, while upregulating PGC-1alpha by ZLN005 promoted mitochondrial biosynthesis and function by regulating the expression of d

Adult mice were implanted orthotopically with the malignant brain tumors and KetoCal was administered to the mice in either unrestricted amounts or in restricted amounts to reduce total c

eased accuracy to HBB mutation-specific strategies encompassing adult hemoglobin (HbA) restoration for a personalized treatment of hemoglobinopathies. Moreover, besides genome editing, m
